# Community-based models of care for adolescent and adult depression, suicidal behavior, anxiety, trauma, and substance use in Africa: a scoping review

**DOI:** 10.3389/fpsyg.2024.1241403

**Published:** 2024-02-09

**Authors:** Fabian Raeber, Maria-Inés Haldemann, Somidha Ray, Jacqueline Huber, Emmanuel Firima, Lucia Gonzalez Fernandez, Alain Amstutz, Felix Gerber, Niklaus D. Labhardt, Jennifer M. Belus

**Affiliations:** ^1^Division of Clinical Epidemiology, Department of Clinical Research, University and University Hospital Basel, Basel, Switzerland; ^2^Research Consultant, International Center for Research on Women, New Delhi, India; ^3^Swiss TPH Library, Swiss Tropical and Public Health Institute, Allschwil, Basel, Switzerland; ^4^Department of Medicine, Swiss Tropical and Public Health Institute, Allschwil, Switzerland

**Keywords:** community-based care, mental health, substance use, Africa, scoping review

## Abstract

**Background:**

Community-based care (CBC), where care is delivered outside of the traditional health facility setting, has been proposed to narrow the mental health (MH) and substance use (SU) treatment gap in Africa.

**Objective:**

This scoping review aims to comprehensively summarize CBC models addressing adolescent and adult MH (depression, anxiety, trauma, suicidal behavior) and (non-tobacco) SU problems in Africa.

**Methods:**

We searched PsycINFO, Embase, Scopus, CINAHL, and Medline Ovid. Studies and protocols were included if they reported on CBC intervention’s effects on MH or SU symptoms/ diagnoses, acceptability, feasibility, or patient engagement in care, regardless of whether the intervention itself was designed specifically for MH or SU.

**Results:**

Among 11,477 screened publications, 217 were eligible. Of the unique intervention studies (*n* = 206), CBC models were classified into the following approaches (non-mutually exclusive): psychotherapeutic (*n* = 144), social (*n* = 81), lifestyle/physical health (*n* = 55), economic (*n* = 26), and psychopharmacological (*n* = 2). While quantitative results suggest possible efficacy of CBC models, description of CBC location was often poor. Fewer interventions addressed suicidal behavior (*n* = 12), the needs of adolescents (*n* = 49), or used traditional healers or religious figures as providers (*n* = 3).

**Conclusion:**

Many CBC models have been tested on MH and SU in Africa and should be critically appraised and meta-analyzed in subsequent reviews, where possible.

## 1 Introduction

Globally, mental health (MH) and substance use (SU) problems account for 20% of years lived with disability. In low- and middle-income countries (LMICs), and Africa^1^ specifically, the MH burden^2^ is similar to the global burden (GBD 2019 Mental Disorders Collaborators, 2022). However, most individuals in low-resource settings lack access to MH care and over 75% of individuals needing MH support do not receive it (Williams et al., 2008; Rathod et al., 2016).

Evidence-based approaches are required to close the MH treatment gap in low-resource settings (Patel et al., 2018). Barriers to accessing MH care in low-resource settings include overburdened health facilities with inadequate preparation to provide services, transportation costs and time for patients to reach clinics, and MH stigma that leads to unwelcoming environments for patients (Hanlon et al., 2014; Badu et al., 2018; Muhorakeye and Biracyaza, 2021). To overcome the barrier of lack of specialized staff and to scale up MH care, task-shifting to non-specialist primary care providers was initially proposed as one strategy (World Health Organization, 2008a). In task-shifting, healthcare tasks are reallocated from highly qualified professionals to workers with less training, which optimizes the use of available human resources (World Health Organization, 2008b). However, task-shifting within traditional health care facilities does not address all the barriers, particularly those related to overburdened physical spaces and patient transportation costs and time.

Community-based care (CBC), where care is provided outside of these traditional healthcare facilities, might be a strategy to overcome such barriers. CBC has effectively been used for improving maternal and neonatal outcomes, and in the HIV epidemic for increasing access to HIV testing and treatment (Lassi and Bhutta, 2015; Geldsetzer et al., 2017; Labhardt et al., 2018; Fox et al., 2019). CBC can be provided in various locations, including patients’ homes, through telehealth, mobile treatment units, or other community settings, such as places of worship or schools. Interventions can even be delivered by a range of non-specialist *lay* providers, such as peers, teachers, or religious figures (Iheanacho et al., 2015; Tol et al., 2015; Reginald Fils-Aime et al., 2018; Fu et al., 2020; Bliznashka et al., 2021).

Systematic reviews and meta-analyses on CBC for MH problems in LMICs, such as home-based interventions for adults living with schizophrenia (Asher et al., 2017) and telehealth interventions addressing depression, anxiety, and SU (Fu et al., 2020; Carter et al., 2021), have indicated evidence of feasibility and effectiveness. However, these reviews focused on a specific type of CBC model and primarily analyzed studies originating from LMICs outside of Africa. In Africa, there is an epidemiological transition from infectious to non-communicable diseases, which challenges the existing health care systems (Gouda et al., 2019). Therefore, guiding the next steps on how to adapt the systems in this specific setting is crucial. To date, there is no general overview of CBC models for MH problems in Africa. The aim of this scoping review was to fill this gap and inform investigators and policymakers of the existing research on CBC models for MH in Africa. Specifically, the objectives of this scoping review were to: (1) compile, describe, and categorize types or models of CBC for MH (depression, anxiety, trauma, and suicidal behavior) and SU in adolescents (10–17 years old) and adults in Africa; (2) evaluate the described models of care in terms of relevant outcomes: effects on the targeted MH or SU symptoms or diagnoses, intervention acceptability and feasibility, and patient engagement in care; and (3) identify gaps in the literature concerning CBC models in Africa.

## 2 Methods

### 2.1 Study design and protocol registration

This review is part of the ComBaCaL (Community-Based Chronic Care Lesotho) project, a 5 year project addressing non-communicable diseases in Lesotho.^3^

This study followed the guidance on scoping reviews by the standardized approach described by Arksey and O’Malley (2005), which was further developed by Levac et al. (2010) and the Joanna Briggs Institute (Peters et al., 2020). The study protocol was registered on open science framework (OSF).^4^ We report our results according to the Preferred Reporting Items for Systematic reviews and Meta-Analyses extension for Scoping Reviews (PRISMA-ScR) guidelines (See Supplementary Table S1 for Checklist).

### 2.2 Eligibility criteria

We used the following inclusion criteria for studies: (1) participants at least 10 years old; (2) based in Africa; (3) addressed any of these most commonly assessed and observed MH problems in Africa (Greene et al., 2021): depression, anxiety, trauma, suicidal behavior, alcohol use or drug use; (4) used a CBC model, wherein a meaningful proportion of the care (defined by us as at least 50% of intervention duration/sessions) was provided outside of traditional healthcare facilities (such as hospitals, primary care clinics or private practices); healthcare facilities which were themselves based in community settings (such as in a church or school) were included; (5) reported or planned to report (for protocol papers) on at least one of the following outcomes related to the intervention: effects on the targeted MH or SU symptoms or diagnoses, intervention acceptability (provider or participant), intervention feasibility, or patient engagement in care; (6) peer-reviewed journal article with study design that described primary data collection (includes protocol papers). No publication date or language restrictions were employed for articles found in the search (See Table 1 for detailed inclusion criteria).

**Table 1:** Study inclusion and exclusion criteria.

Field	Inclusion criteria	Exclusion criteria
Population	Adolescents (of at least 10 years or older) or adults (18 years or older) Symptoms or a diagnosis of any of the following MH or SU problems: depression or suicidal behavior, anxiety, trauma, alcohol use, drug use	Children <10 years of age
Geographic region	Africa Including: Angola, Benin, Botswana, Burkina Faso, Burundi, Cameroon, Cape Verde, Central African Republic, Chad, Comoros, Congo, Cote d’Ivoire, Djibouti, Equatorial New Guinea, Eritrea, Ethiopia, Eswatini, Gabon, Gambia, Ghana, Guinea, Guinea-Bissau, Kenya, Lesotho, Liberia, Madagascar, Malawi, Mali, Mauritania, Mauritius, Mayotte, Mozambique, Namibia, Niger, Nigeria, La Réunion, Rwanda, Sao Tome and Principe, Senegal, Seychelles, Sierra Leone, Somalia, South Africa, South Sudan, Sudan, United Republic of Tanzania, Togo, Uganda, Zaire, Zambia, Zimbabwe	Outside of these specified countries
Intervention/model of care	Treatment for MH or SU problems (as defined under population), wherein a substantial proportion of the care is provided outside of healthcare facilities settings. Interventions that take place in healthcare facilities which are themselves based in community settings, such as in a church or a school, are eligible.	Report solely about interventions provided in typical healthcare facilities (e.g., standard clinic, hospital) Description provided does not describe all the characteristics needed to define the model
Comparators	If available, will compare to standard services delivered in healthcare facility settings	None
Outcomes	Reports one or more of the following outcomes: • intervention acceptability (defined as explicit use of the term by study author and includes a statement on how the outcome was evaluated) • intervention feasibility (defined as explicit use of the term by study author and includes a statement on how the outcome was evaluated) • patient engagement in care (either the explicit use of the term by study author or as seeking help for MH or SU care) • MH and SU symptoms or diagnoses (as defined by study authors)	None
Type of studies	Any peer-reviewed study that reports on planned or executed research on CBC models of care for MH or SU problems	Study designs that do not describe primary data collection, including reviews, treatment guidelines, mathematical models, editorials, or commentaries. Conference abstracts
Timing	No limits	None
Language	No limits	None

In our protocol, psychosocial outcomes (e.g., substance-related problems, quality of life) were an inclusion criterion. After abstract screening we chose to exclude psychosocial outcomes as an inclusion criterion to reduce the number of studies included in our review. However, we still extracted data on psychosocial outcomes from the studies that were ultimately included.

### 2.3 Search strategy

The search strategy was developed by two of the investigators (FR, JB) with the help of a professional medical librarian (JH) and peer-reviewed by a medical information specialist (CA). Using Embase Elsevier, a search string was created consisting of three components: MH problems, geographic location, and CBC. Suitable terms were also searched in Emtree and MeSH term synonym lists. To ensure an extensive coverage of CBC terms, the 100 most relevant abstracts as well as all review abstracts of a preliminary search were screened for eligible terms. The search string was translated, and abstracts were screened from the following databases: Embase, Medline Ovid, PsycINFO, Scopus, and CINAHL. The original search was conducted on September 2, 2021, with an updated search conducted on February 7, 2023. With EndNote X7, the results from the databases were pooled and duplicates removed. We refrained from further citation searching (i.e., forward and backward citation).^5^ An in-depth explanation of the search string development can be found in our protocol (see text footnote 4) and the final search strings are displayed in the Supplementary data.

### 2.4 Study selection

Titles and abstract screening and full-text screening were done with Covidence according to our eligibility criteria. Due to the large number of studies identified, we decided to deviate from the study protocol and refrain from including articles that solely reported on related psychosocial outcomes (e.g., a study that only looked at the effect of peer relationship quality after receiving a CBC intervention was excluded). To ensure a synchronized screening process, three reviewers (FR, M-IH, JB) independently evaluated batches of 50 abstracts until fewer than five discrepancies arose. Consequently, two reviewers (FR, M-IH) independently screened the abstracts and full-texts and discussed unsolved discrepancies with the third reviewer (JB) to reach consensus. Study authors were contacted in case of missing or unclear information.

### 2.5 Data extraction

After preliminary evaluation of the included studies, we defined intervention categories and components to categorize the CBC models used. The extraction template and process were also conducted within Covidence. Three reviewers (FR, M-IH, and SR) independently conducted initial data extraction from the studies’ full-texts; the accuracy of this information was then verified by a second reviewer. In case of discrepancies, the fourth reviewer (JB) was consulted. Information obtained from study authors was utilized to supplement or confirm data. Extracted data included: study authors, year of publication, journal, study design, sample size, study setting (country, urban/rural area), location of service delivery within the community, participant characteristics, MH problems addressed, MH inclusion or exclusion criteria, characteristics of intervention (and of comparator, if available), and outcomes reported (effects on MH symptoms or diagnoses, patient or provider acceptability, feasibility, patient engagement in care, or related psychosocial outcomes).

## 3 Results

### 3.1 Search results and description of study characteristics

#### 3.1.1 Search results

**Figure 1:**
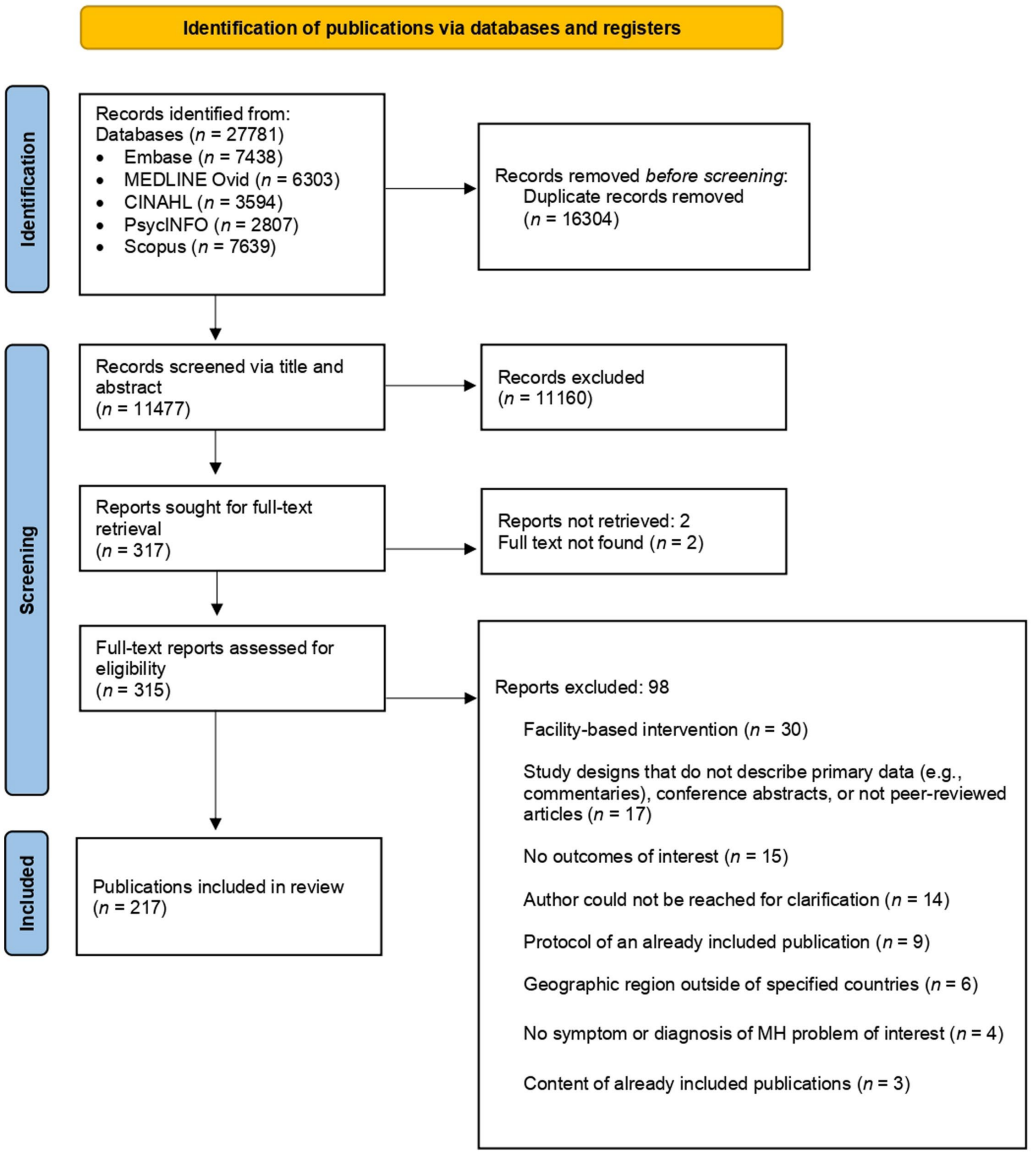
PRISMA flow diagram of identified publications.

Overall, 27,781 publications were identified, of which 11,477 remained after duplicate removal. Title and abstract screening yielded 315 retrievable publications for full-text review. During full-text review, we contacted 45 authors for clarification of unclear or missing information. Ninety-eight publications were excluded at this stage, primarily because they took place in traditional healthcare facilities (*n* = 30), did not describe primary data (*n* = 17), or did not report required outcomes (*n* = 15). Thus, a total of 217 publications were eligible for data extraction (See Figure 1 for PRISMA flow diagram). A table summarizing all the 217 publications can be found in Supplementary Tables S2, S3. Of these 217 studies, *n* = 5 reported on follow-up timepoints (Tomlinson et al., 2016, 2021; Rotheram-Borus et al., 2019; Stansert Katzen et al., 2020; Rossouw et al., 2022) and *n* = 6 reported on different outcomes of interventions that were already included (Chaudhury et al., 2016; Van de Water et al., 2018; Sherr et al., 2020; Giusto et al., 2021, 2022; Greene et al., 2022). As some publications reported on the same intervention that was tested within the same population, but evaluated different outcomes across the various publications, we considered an intervention unique if no other included publication reported on the same intervention within the same population. Meaning, a total of 206 unique interventions were identified across 217 publications. Thus, our totals differ when we analyze the models (*n* = 206) and the outcomes of these models (*n* = 217).

#### 3.1.2 Year of publication, study setting, and study designs

**Figure 2:**
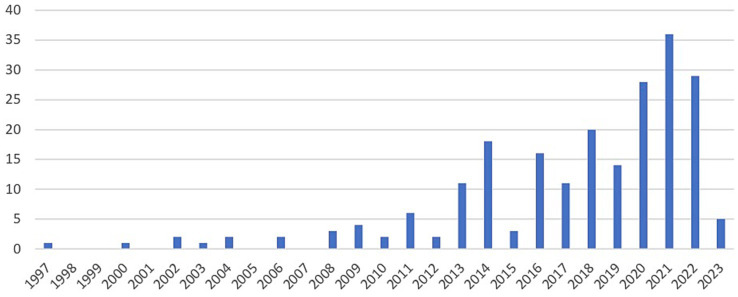
Number of identified publications per year (*n* = 217) until February 7, 2023.

**Figure 3:**
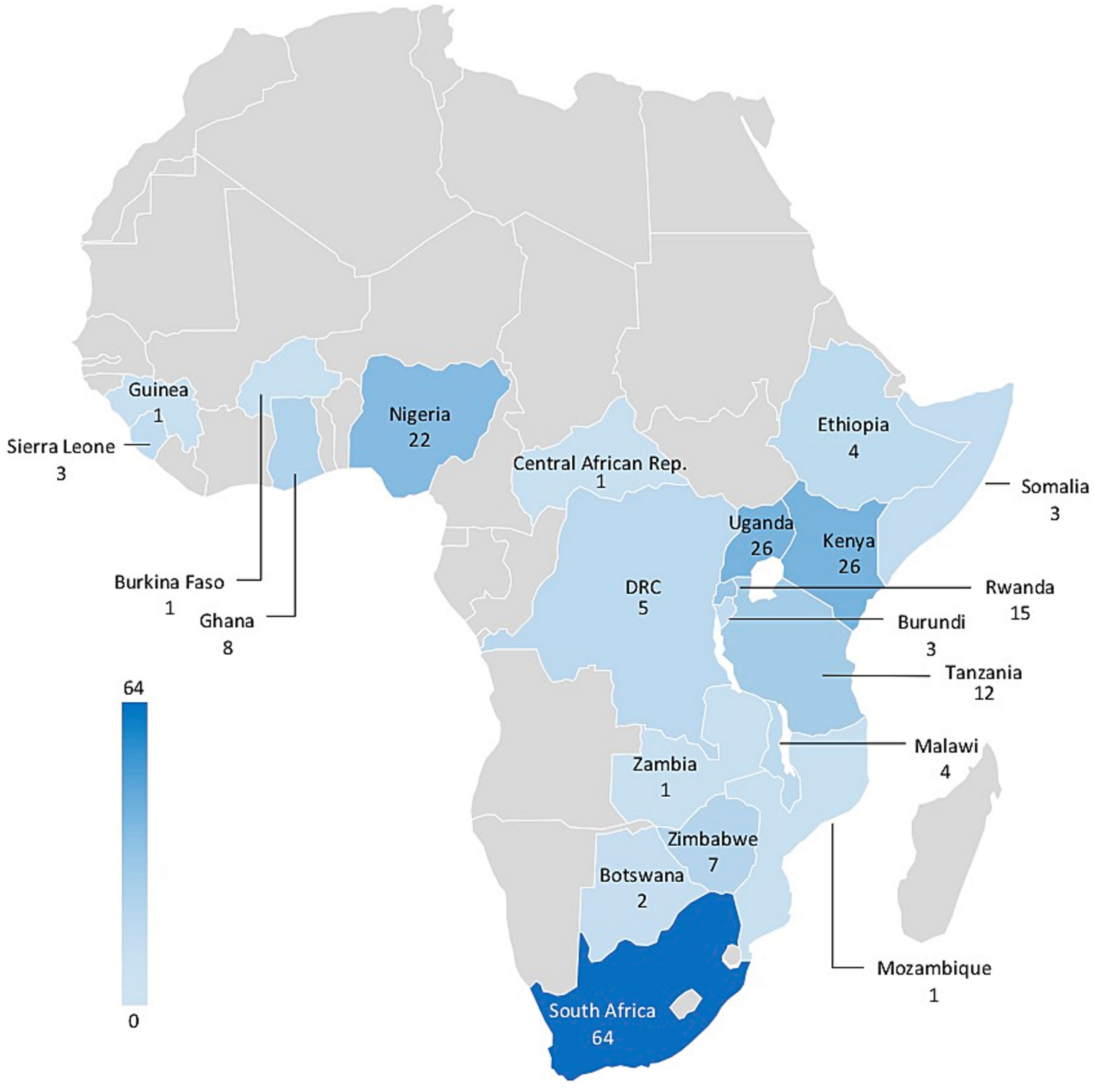
Geographic distribution of unique interventions in Africa (*n* = 206). Only countries with identified studies are labeled and Mauritius (*n* = 2) is not represented on this map.

Studies were published between 1997 and 2023, with increasing numbers in more recent years. Over half of studies (132/217) were published between 2018 and 2023 (see Figure 2). Geographically, approximately one third of the interventions were conducted in South Africa (64/206) and another third in the Great Victoria Lake area (Uganda, Kenya, and Tanzania; 64/206). Further countries with a considerable number of CBC interventions tested were Nigeria (22/206), Rwanda (15/206), Ghana (8/206), and Zimbabwe (7/206) (See Figure 3). More interventions were conducted in urban settings (89/206) than in rural/semi-rural settings (63/206) or in mixed urban/rural setting (16/206). No clear setting could be identified for 38/206 studies. Close to 10% of interventions were conducted in refugee camps (16/206).

Publications reported (or planned to report in the case of protocols) on quantitative data only (153/217), mixed methods (52/217), and on qualitative data only (12/217). Of the unique intervention studies, 61/206 were pilot or feasibility studies and 24/206 were protocols of ongoing or proposed studies. Of the unique intervention studies that used quantitative and mixed methods, 86/172 were randomized controlled trials (RCTs), 36/172 quasi-experimental/non-randomized comparative studies, and 50/172 single arm pre-post studies.

#### 3.1.3 Study target conditions and participants

The majority of interventions addressed depression (161/206), followed by anxiety (90/206), alcohol use (51/206), trauma (46/206), drug use (25/206), and suicidal behavior (12/206). A median of two targeted MH problems (range: 1 to 5) were addressed per intervention. However, 80/206 interventions required a minimum MH symptom threshold or diagnosis for study inclusion. Number of study participants ranged from 9 to 4,126. Most interventions (171/206) focused on a specific target group, including students (50/206), parents/caregivers (43/206), of which pregnant women were a relatively common subgroup (14/206), people living with HIV or people affected by HIV (e.g., family members of people living with HIV or having died of HIV-related causes; 26/206), refugees (20/206), survivors of war, genocide, torture, or child soldiers (14/206), orphans (11/206), and victims of gender-based violence (9/206).

Forty-six percent (95/206) of interventions targeted adults only, 24% (49/206) adolescents only, 28% (57/206) included both adults and adolescents, and 2% (5/206) were unclear. Only two studies focused on older adults aged at least 50 (Geffen et al., 2019; Lloyd-Sherlock et al., 2019). The presence of certain MH problems was an exclusion criterion in approximately a quarter (47/206) of studies, with severe MH disorder (36/206), suicidal behavior (25/206), or SU (9/206) as the most common. See Table 2 for a more detailed description of the study target conditions and participants.

**Table 2:** Targeted population.

Target group *n* (%)	MH problems addressed *n* (%)	Targeted age group *n* (%)	Community setting n (%)	MH problems as inclusion criteria *n* (%)	Excluded participants due to MH reasons *n* (%)
Total interventions *n* = 206 (100%)	Depression, *n* = 161 (78%) Anxiety, *n* = 90 (44%) Suicidal behavior, *n* = 12 (6%) Trauma, *n* = 46 (22%) Alcohol use, *n* = 51 (25%) Drug use, *n* = 25 (12%)	Adults, *n* = 95 (46%) Adolescents, *n* = 49 (24%) Mixed, *n* = 57 (28%) Unknown, *n* = 5 (2%)	Urban, *n* = 89 (43%) Rural, *n* = 63 (31%) Mixed, *n* = 16 (8%) Unknown, *n* = 38 (18%)	Depression/anxiety, *n* = 49 (24%) Alcohol/drug use, *n* = 17 (8%) Trauma, *n* = 21 (10%)	Suicidal behavior, *n* = 25 (12%) Alcohol use, *n* = 8 (4%) Drug use, *n* = 8 (4%) Severe MH, *n* = 36 (17%) Other^a^, *n* = 15 (7%)
Parents/caregivers *n* = 43 (21%)	Depression, *n* = 42 (98%) Anxiety, *n* = 21 (49%) Suicidal behavior, *n* = 4 (9%) Trauma, *n* = 4 (9%) Alcohol use, *n* = 8 (19%) Drug use, *n* = 4 (9%)	Adults, *n* = 27 (63%) Adolescents, *n* = 2 (5%) Mixed, *n* = 13 (30%) Unknown *n* = 1 (2%)	Urban, *n* = 18 (42%) Rural, *n* = 16 (37%) Mixed, *n* = 3 (7%) Unknown, *n* = 6 (14%)	Depression/anxiety, *n* = 8 (19%) Alcohol/drug use, *n* = 3 (7%) Trauma, *n* = 1 (2%)	Suicidal behavior, *n* = 6 (14%) Alcohol use, *n* = 2 (5%) Drug use, *n* = 1 (2%) Severe MH, *n* = 9 (21%) Other, *n* = 2 (5%)
Students *n* = 50 (24%)	Depression, *n* = 36 (72%) Anxiety, *n* = 24 (48%) Suicidal behavior, *n* = 3 (6%) Trauma, *n* = 7 (14%) Alcohol use, *n* = 10 (20%) Drug use, *n* = 7 (14%)	Adults, *n* = 5 (10%) Adolescents, *n* = 33 (66%) Mixed, *n* = 12 (24%)	Urban, *n* = 33 (66%) Rural, *n* = 4 (8%) Mixed, *n* = 1 (2%) Unknown, *n* = 12 (24%)	Depression/anxiety, *n* = 16 (32%) Alcohol/drug use, *n* = 2 (4%) Trauma, *n* = 6 (12%)	Suicidal behavior, *n* = 3 (6%) Alcohol use, *n* = 2 (4%) Drug use, *n* = 2 (4%) Severe MH, *n* = 5 (10%) Other, *n* = 6 (12%)
Affected by HIV/HIV+ *n* = 26 (13%)	Depression, *n* = 22 (85%) Anxiety, *n* = 12 (46%) Suicidal behavior, *n* = 1 (4%) Trauma, *n* = 1 (4%) Alcohol use, *n* = 5 (19%) Drug use, n = 3 (12%)	Adults, *n* = 16 (62%) Adolescents, *n* = 7 (27%) Mixed, *n* = 3 (12%)	Urban, *n* = 9 (35%) Rural, *n* = 11 (42%) Mixed, *n* = 4 (15%) Unknown, *n* = 2 (7%)	Depression/anxiety, *n* = 6 (23%) Alcohol/drug use, *n* = 2 (7%) Trauma, *n* = 0 (0%)	Suicidal behavior, *n* = 5 (19%) Alcohol use, *n* = 2 (7%) Drug use, *n* = 2 (7%) Severe MH, *n* = 4 (15%) Other, *n* = 3 (12%)
Refugees *n* = 20 (10%)	Depression, *n* = 16 (80%) Anxiety, *n* = 12 (60%) Suicidal behavior, *n* = 1 (5%) Trauma, *n* = 12 (60%) Alcohol use, *n* = 0 (0%) Drug use, *n* = 0 (0%)	Adults, *n* = 7 (35%) Adolescents, *n* = 4 (20%) Mixed, *n* = 9 (45%)	Urban, *n* = 6 (30%) Rural, *n* = 7 (35%) Mixed, *n* = 0 (0%) Unknown, *n* = 7 (35%)	Depression/anxiety, *n* = 7 (35%) Alcohol/drug use, *n* = 0 (0%) Trauma, *n* = 6 (30%)	Suicidal behavior, *n* = 6 (30%) Alcohol use, *n* = 2 (10%) Drug use, *n* = 2 (10%) Severe MH, *n* = 7 (35%) Other, *n* = 1 (5%)
Pregnant women^b^ *n* = 14 (7%)	Depression, *n* = 14 (100%) Anxiety, n = 6 (43%) Suicidal behavior, *n* = 1 (7%) Trauma, *n* = 0 (0%) Alcohol use, *n* = 3 (21%) Drug use, *n* = 1 (7%)	Adults, *n* = 9 (64%) Adolescents, *n* = 0 (0%) Mixed, *n* = 5 (36%)	Urban, *n* = 6 (43%) Rural, *n* = 6 (43%) Mixed, *n* = 1 (7%) Unknown, *n* = 1 (7%)	Depression/anxiety, *n* = 4 (29%) Alcohol/drug use, *n* = 0 (0%) Trauma, *n* = 0 (0%)	Suicidal behavior, *n* = 3 (21%) Alcohol use, *n* = 1 (7%) Drug use, *n* = 1 (7%) Severe MH, *n* = 4 (29%) Other, *n* = 1 (7%)
Survivors of war, genocide, torture, or child soldiers *n* = 14 (7%)	Depression, *n* = 11 (79%) Anxiety, *n* = 7 (50%) Suicidal behavior, *n* = 1 (7%) Trauma, *n* = 12 (86%) Alcohol use, *n* = 0 (0%) Drug use, *n* = 0 (0%)	Adults, *n* = 6 (43%) Adolescents, *n* = 3 (21%) Mixed, *n* = 3 (21%) Unknown, *n* = 2 (14%)	Urban, *n* = 3 (21%) Rural, *n* = 8 (57%) Mixed, *n* = 0 (0%) Unknown, *n* = 3 (21%)	Depression/anxiety, *n* = 4 (29%) Alcohol/drug use, *n* = 0 (0%) Trauma, *n* = 7 (50%)	Suicidal behavior, *n* = 0 (0%) Alcohol use, *n* = 1 (7%) Drug use, *n* = 1 (7%) Severe MH, *n* = 2 (14%) Other, *n* = 1 (%)
Orphans *n* = 11 (5%)	Depression, *n* = 9 (82%) Anxiety, *n* = 2 (18%) Suicidal behavior, *n* = 1 (9%) Trauma, *n* = 4 (36%) Alcohol use, *n* = 1 (9%) Drug use, n = 0 (0%)	Adults, *n* = 0 (0%) Adolescents, *n* = 9 (82%) Mixed, *n* = 2 (18%)	Urban, *n* = 2 (18%) Rural, *n* = 5 (45%) Mixed, *n* = 1 (9%) Unknown, *n* = 3 (27%)	Depression/anxiety, *n* = 0 (0%) Alcohol/drug use, *n* = 0 (0%) Trauma, *n* = 3 (27%)	Suicidal behavior, *n* = 0 (0%) Alcohol use, *n* = 0 (0%) Drug use, *n* = 0 (0%) Severe MH, *n* = 0 (0%) Other, *n* = 0 (0%)
Gender-based violence *n* = 9 (4%)	Depression, *n* = 9 (100%) Anxiety, *n* = 5 (56%) Suicidal behavior, *n* = 0 (0%) Trauma, *n* = 8 (89%) Alcohol use, *n* = 1 (11%) Drug use, *n* = 0 (0%)	Adults, *n* = 7 (78%) Adolescents, *n* = 1 (11%) Mixed, *n* = 1 (11%)	Urban, *n* = 4 (44%) Rural, *n* = 3 (33%) Mixed, *n* = 1 (11%) Unknown, *n* = 1 (11%)	Depression/anxiety, *n* = 5 (56%) Alcohol/drug use, *n* = 0 (0%) Trauma, *n* = 3 (33%)	Suicidal behavior, *n* = 5 (56%) Alcohol use, *n* = 0 (0%) Drug use, *n* = 0 (0%) Severe MH, *n* = 5 (56%) Other, *n* = 0 (0%)

^a^Other MH reasons include current or past psychological/psychopharmacological treatment, panic disorder, conduct disorder, acute intoxication. ^b^Pregnant women are also included in the Parent/Caregiver group.

### 3.2 Description of community-based care models

#### 3.2.1 Location of delivery

Location of CBC delivery was diverse, but often poorly reported and thus the most frequent reason for author contacts during clarification. The most common locations of intervention delivery were (not mutually exclusive): educational organizations (62/206), such as high schools or universities, homes (47/206), telehealth (36/206), faith-based gathering places, such as churches or mosques (13/206), non-governmental organization offices (9/206), and housing centers for vulnerable populations, such as women’s shelters or orphanages (6/206). Twenty-eight percent (57/206) were conducted in other community places, which comprised locations that were difficult to classify. This included for example, streets or outdoor areas, such as gardens or gathering places. Some authors stated that the intervention delivery location varied between communities within the same intervention, due to changes in location availability as well as participant accessibility and choice. The amount of care provided within the community varied; some interventions were solely delivered in such settings.

#### 3.2.2 Providers

Most CBC interventions engaged lay health workers (121/206), followed by specialist MH providers (60/206) and health care workers who did not specialize in MH (12/206). There were various types of lay health workers who were trained in providing CBC interventions, which were not mutually exclusive: community health workers (60/206), peers (40/206), teachers (15/206), and faith-based community members (3/206). Specialist MH workers were comprised of counselors or psychotherapists, including psychology students (38/206), social workers (11/206), psychiatric nurses (6/206), and psychiatrists (6/206). Health care workers who did not specialize in MH included general nurses (7/206), physicians (4/206), pharmacists (1/206), dieticians (1/206), laboratory technicians (1/206), and physical fitness counselors (1/206).

#### 3.2.3 Intervention approach categories

After preliminary evaluation of included publications, we developed a categorization for interventions, as follows: psychotherapeutic, social, lifestyle/physical health, economic, and psychopharmacological interventions. Table 3 provides a detailed overview of these intervention categories. Although these categories were not mutually exclusive, 120/206 interventions reported on an approach that was classified solely within one intervention category. The remaining 86/206 interventions were classified within multiple categories.

**Table 3:** Intervention categories.

Intervention category *n* (%)	MH problems addresed *n* (%)	Location delivery *n* (%)	Provider *n* (%)	Assessed quantitative outcomes *n* (%)	Assessed qualitative outcomes *n* (%)
Total studies *n* = 206^a^ (100%)	Depression, *n* = 161 (78%) Anxiety, *n* = 90 (44%) Suicidal behavior, *n* = 12 (6%) Trauma, *n* = 46 (22%) Alcohol use, *n* = 51 (25%) Drug use, *n* = 25 (12%)	Home-based, *n* = 47 (23%) Educational institutions, *n* = 62 (30%) Telehealth, *n* = 36 (17%) Orphanage, *n* = 3 (1%) Shelter, *n* = 3 (1%) NGO offices, *n* = 9 (4%) Faith-based, *n* = 13 (6%) Other community places, *n* = 57 (28%)	Specialized mental health, *n* = 60 (29%) Health care workers, *n* = 12 (6%) Lay provider, *n* = 122 (59%) Unknown/not reported, *n* = 21 (10%)	Effect on MH, *n* = 199 (92%) Acceptability, *n* = 40 (18%) Feasibility, *n* = 34 (16%) Engagement in care, *n* = 7 (3%) Psychosocial outcomes, *n* = 131 (60%)	Effect on MH, *n* = 11 (51%) Acceptability, *n* = 47 (22%) Feasibility, *n* = 24 (11%) Engagement in care, *n* = 0 (0%) Psychosocial outcomes, *n* = 12 (6%)
Psychotherapeutic *n* = 144 (70%)	Depression, *n* = 117 (81%) Anxiety, *n* = 72 (50%) Suicidal behavior, *n* = 9 (6%) Trauma, *n* = 41 (28%) Alcohol use, *n* = 30 (21%) Drug use, *n* = 15 (10%)	Home-based, *n* = 24 (17%) Educational institutions, *n* = 53 (37%) Telehealth, *n* = 24 (17%) Orphanage, *n* = 3 (2%) Shelter, *n* = 2 (1%) NGO offices, *n* = 6 (4%) Faith-based, *n* = 8 (6%) Other community places, *n* = 37 (26%)	Specialized mental health, *n* = 52 (36%) Health care workers, *n* = 8 (6%) Lay provider, *n* = 86 (60%) Unknown/not reported, *n* = 10 (7%)	Effect on MH, *n* = 136 (90%) Acceptability, *n* = 32 (21%) Feasibility, *n* = 28 (19%) Engagement in care, *n* = 7 (5%) Psychosocial outcomes, *n* = 91 (60%)	Effect on MH, *n* = 8 (5%) Acceptability, *n* = 41 (27%) Feasibility, *n* = 20 (13%) Engagement in care, *n* = 0 (0%) Psychosocial outcomes, *n* = 6 (4%)
Social *n* = 81 (39%)	Depression, *n* = 67 (83%) Anxiety, *n* = 32 (40%) Suicidal behavior, *n* = 6 (7%) Trauma, *n* = 18 (22%) Alcohol use, *n* = 20 (25%) Drug use, *n* = 13 (16%)	Home-based, *n* = 31 (38%) Educational institutions, *n* = 15 (19%) Telehealth, *n* = 6 (7%) Orphanage, *n* = 1 (1%) Shelter, *n* = 2 (2%) NGO offices, *n* = 5 (6%) Faith-based, *n* = 6 (7%) Other community places, *n* = 31 (38%)	Specialized mental health, *n* = 18 (22%) Health care workers, *n* = 2 (2%) Lay provider, *n* = 57 (70%) Unknown/not reported, *n* = 8 (10%)	Effect on MH, *n* = 83 (92%) Acceptability, *n* = 12 (13%) Feasibility, *n* = 10 (11%) Engagement in care, *n* = 2 (2%) Psychosocial outcomes, *n* = 62 (69%)	Effect on MH, *n* = 7 (8%) Acceptability, *n* = 18 (%) Feasibility, *n* = 8 (9%) Engagement in care, *n* = 0 (0%) Psychosocial outcomes, *n* = 10 (10%)
Lifestyle/physical health *n* = 55 (27%)	Depression, *n* = 29 (53%) Anxiety, *n* = 14 (25%) Suicidal behavior, *n* = 1 (2%) Trauma, *n* = 2 (4%) Alcohol use, *n* = 28 (51%) Drug use, *n* = 13 (24%)	Home-based, *n* = 15 (27%) Educational institutions, *n* = 11 (20%) Telehealth, *n* = 12 (22%) Orphanage, *n* = 0 (0%) Shelter, *n* = 1 (2%) NGO offices, *n* = 3 (5%) Faith-based, *n* = 2 (4%) Other community places, *n* = 15 (27%)	Specialized mental health, *n* = 11 (20%) Health care workers, *n* = 5 (9%) Lay provider, *n* = 35 (64%) Unknown/not reported, *n* = 6 (11%)	Effect on MH, *n* = 56 (95%) Acceptability, *n* = 8 (14%) Feasibility, *n* = 6 (10%) Engagement in care, *n* = 0 (%) Psychosocial outcomes, *n* = 40 (68%)	Effect on MH, *n* = 2 (3%) Acceptability, *n* = 9 (15%) Feasibility, *n* = 6 (10%) Engagement in care, *n* = 0 (0%) Psychosocial outcomes, *n* = 4 (7%)
Economic *n* = 26 (13%)	Depression, *n* = 23 (88%) Anxiety, *n* = 8 (31%) Suicidal behavior, *n* = 2 (7%) Trauma, *n* = 5 (19%) Alcohol use, *n* = 5 (19%) Drug use, *n* = 2 (7%)	Home-based, *n* = 11 (42%) Educational institutions, *n* = 3 (12%) Telehealth, *n* = 2 (7%) Orphanage, *n* = 0 (0%) Shelter, *n* = 0 (0%) NGO offices, *n* = 0 (0%) Faith-based, *n* = 3 (12%) Other community places, *n* = 8 (31%)	Specialized mental health, *n* = 4 (15%) Health care workers, *n* = 2 (8%) Lay provider, *n* = 15 (58%) Unknown/not reported, *n* = 5 (19%)	Effect on MH, *n* = 26 (100%) Acceptability, *n* = 1 (4%) Feasibility, *n* = 2 (7%) Engagement in care, *n* = 0 (0%) Psychosocial outcomes, *n* = 17 (63%)	Effect on MH, *n* = 1 (4%) Acceptability, *n* = 0 (0%) Feasibility, *n* = 0 (0%) Engagement in care, *n* = 0 (0%) Psychosocial outcomes, *n* = 1 (4%)

^a^For the quantitative and qualitative evaluation of outcomes, we were interested in all evaluations (i.e., not only in unique interventions). Therefore, the sample sizes for the last two columns are as follows: total studies, *n* = 217; psychotherapeutic, *n* = 151; social, *n* = 90; lifestyle/physical health, *n* = 59; economic, *n* = 27.

##### 3.2.3.1 Psychotherapeutic interventions

Psychotherapeutic interventions were defined as providing knowledge about MH problems (psychoeducation), teaching psychological skills to enhance resilience, supporting improved emotional or behavioral well-being, monitoring MH symptoms, and providing the necessary support to help patients reach their goals. This was the most common approach used (144/206). The psychotherapeutic interventions included (not mutually exclusive) were cognitive behavioral treatment (68/144), stress management (33/144), exposure therapy (21/144), supportive counseling (15/144), motivational interviewing (13/144), psychoeducation only (10/144), creative therapy (e.g., dancing, writing, or music creation; 9/144), and interpersonal therapy (7/144). More than 60% of psychotherapeutic interventions were held in groups (89/144) and the most common location used for psychotherapeutic interventions was educational organizations (53/144).

About 10% of psychotherapeutic interventions utilized a single-session intervention approach (14/144) (Stein et al., 1997; Igreja et al., 2004; Connolly and Sakai, 2011; Cubbins et al., 2012; Eller et al., 2013; Pengpid et al., 2013; Morojele et al., 2014; Lasebikan et al., 2017; De Fouchier and Kedia, 2018; Harder et al., 2020; Osborn et al., 2020; Akena et al., 2021; Wasil et al., 2021; Venturo-Conerly et al., 2022). A total of 10 studies quantitively assessed the effect of a single session intervention on MH or SU, of which eight studies indicated a statistically significant MH or SU reduction. Notably, a self-help component, including distribution of manuals or leaflets about psychoeducation, self-care strategies, problem-solving steps, or a drinking diary, was present in 75% of the single-session interventions that showed such a reduction (6/8) (Connolly and Sakai, 2011; Eller et al., 2013; Pengpid et al., 2013; Lasebikan et al., 2017; De Fouchier and Kedia, 2018; Osborn et al., 2020).

##### 3.2.3.2 Social interventions

Social interventions were the second most frequently described intervention category (81/206). These interventions focused on education about changing social norms (women’s, children’s, diverse gender, and sexual minority rights), teaching skills to prevent conflict emergence (interpersonal or gender-based violence), and strategies to resolve conflicts (peacebuilding exercises, reconciliation programs), strengthening or broadening social relationships (befriending), and providing the necessary support to achieve these goals (mentorship). Social interventions involved families (e.g., parenting programs), schools, and whole communities (such as community action groups) and most were delivered at home (31/81).

The most common intervention in this category consisted of parenting programs, comprising almost half (38/81) of the social interventions. A specific example of this type of intervention was implemented in a rural Ugandan setting and focused not only on promoting child development, but also on enhancing maternal mental well-being through child-care and mother-care sessions. The intervention was facilitated by trained non-professional community members and was conducted in groups in community places. The results showed improved child cognitive and language development, as well as fewer depression symptoms in mothers (Singla et al., 2015).

##### 3.2.3.3 Lifestyle/physical health interventions

Lifestyle/physical health interventions were reported in 55/206 interventions. These interventions consisted of education about physical chronic diseases and their prevention (HIV, tuberculosis, hypertension), measuring and monitoring physical health indicators (HIV testing, blood pressure measurements), providing material and capabilities to address risk factors (physical activity programs, risk reduction plans, condom/clean needle distribution, sex education), providing medical treatment for these physical diseases (e.g., delivery of medication for HIV or hypertension or referral to facility-based care), and providing support to reach these goals (mentors, adherence clubs). The most commonly addressed problem using this approach was depression (29/55), followed by SU (28/55). The specific group most frequently targeted with this approach for addressing SU were individuals affected by HIV (4/55) and students (4/55).

##### 3.2.3.4 Economic interventions

Economic interventions were found in 26/206 publications. Economic interventions aimed at enabling a better livelihood by providing financial or other means (cash transfers, animal stocks, farming equipment, payment for clothes/fees) or the education and skills (workshops on budgets, farming skills) or support (mentorship, saving and lending groups) to reach and maintain economic independence and self-reliance. Almost all economic interventions addressed depression (23/26). The primary focus of economic interventions was on families, with caregivers being the most targeted group (9/26). Economic interventions were frequently (18/26) combined with interventions from other categories, with 15 of the studies integrating a social intervention component.

##### 3.2.3.5 Psychopharmacological interventions

We defined psychopharmacological interventions as those that provide information and/or delivery of psychopharmacological medications (e.g., initiation, refill, dose adjustments), side effect monitoring, and medication adherence support (SMS-reminders, mentors). Only 2/206 interventions reported on a psychopharmacological intervention. One study targeted individuals with chronic psychotic disorders and concomitant use of khat (amphetamine-like plant, where excessive use can evoke psychotic symptoms). The treatment package included home delivery, adherence support, and side effect monitoring of low-dose neuroleptic medication (Odenwald et al., 2012). The other study reported on a mobile inpatient detoxification program for alcohol-dependent adults, which moved together with counselors and medical staff every 7 days to a different community in Northern Uganda (Ertl et al., 2021). The detoxification included medication delivery (benzodiazepines, neuroleptic medication, antidepressants, anticonvulsants) and monitoring and was complemented by psychotherapeutic content. This mobile detoxification center allowed for the inclusion of individuals with severe alcohol dependence, who had withdrawal symptoms and comorbidities, to receive such treatment.

#### 3.2.4 Elements of care

**Figure 4:**
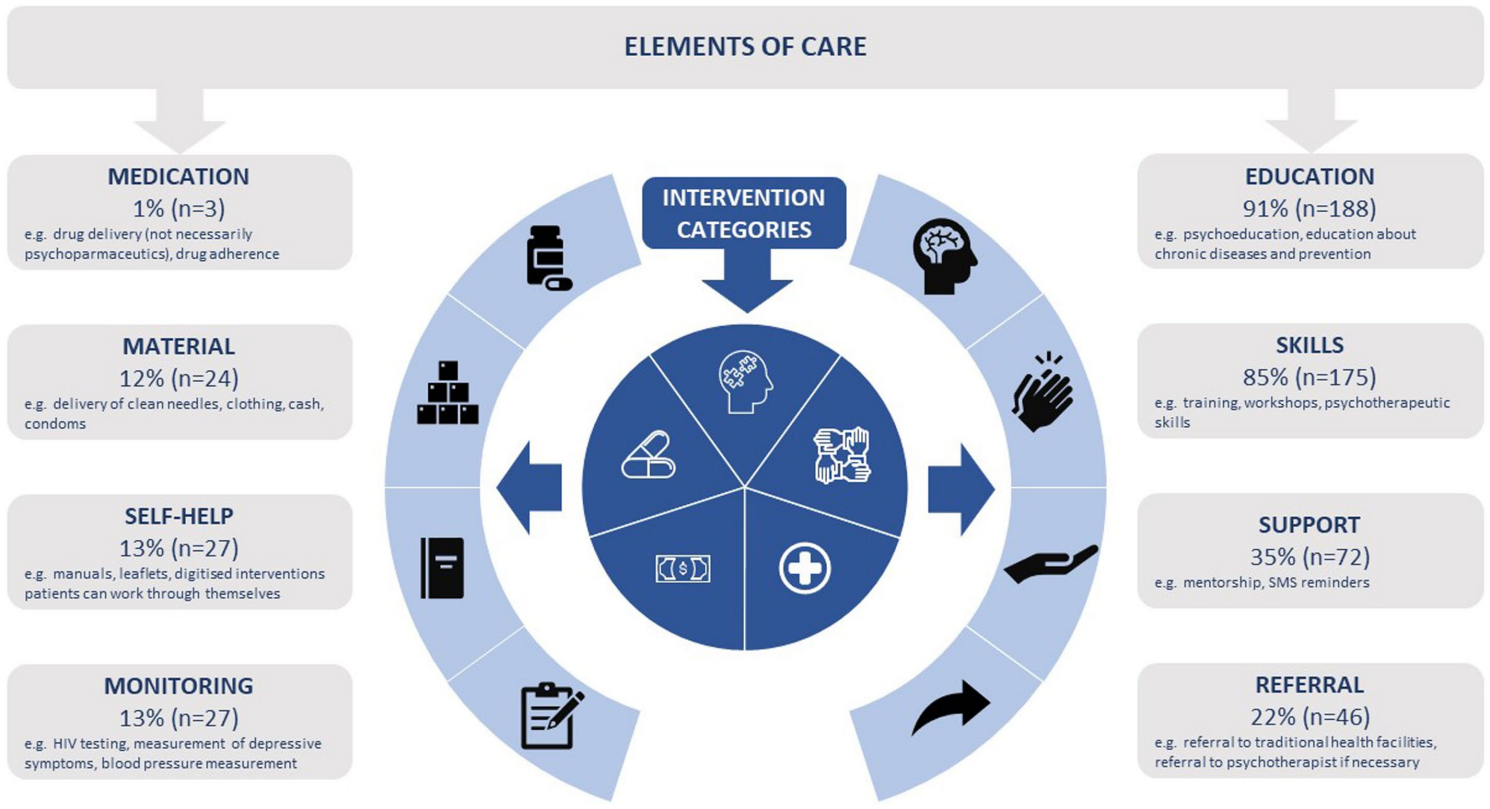
Elements of care and intervention approach categories (*n* = 206).

Given that the intervention approach categories shared common components of care, we further categorized these components and refer to these as *elements of care.* The elements of care were: education, skills, support to reach goals, self-help (e.g., leaflets, journals, digitized interventions), monitoring, medication delivery, material delivery (e.g., cash, condoms, farming equipment), and referral to facility-based care. Figure 4 provides a definition and visualization of the elements of care and the intervention approaches described in the previous section. The vast majority of interventions included education (188/206) or skills (175/206). Almost all interventions (190/206) implemented multiple elements. Four or more elements were applied in 20/206 interventions. Similar utilization of care components were found among lay providers and specialist MH care workers.

### 3.3 Outcomes

#### 3.3.1 Effects on MH problems

Overall, 198/217 publications quantitatively measured (or planned to measure in the case of protocol papers) the effect of the CBC intervention on the MH or SU problems of interest. Effects on symptoms or diagnoses of depression were measured in 157/217 publications, 84/217 on anxiety, 49/217 on alcohol use, 45/217 on traumatic stress, 21/217 on drug use, and 8/217 on suicidal behavior. We further examined the number of non-protocol publications reporting a significant reduction in any of the targeted MH or SU symptoms or diagnoses. In single arm studies, a significant reduction was defined as a statistically significant reduction within the intervention group itself; in multiple group studies (e.g., RCTs), a significant reduction was defined based on a comparative reduction between the groups. Of these publications, 136/175 found a significant reduction in at least one of the measured MH problems at one (or more) time points. Significant reductions were reported in 92/137 publications on depression, in 27/42 on traumatic stress, and in 45/71 on anxiety. Publications investigating the effects on SU had slightly fewer significant results, 22/40 for alcohol and 8/17 for drug use, though both had fewer studies conducted on the topic. Only 2/8 studies on suicidal behavior reported a significant reduction in this outcome.

#### 3.3.2 Acceptability, feasibility, and engagement in care

We included acceptability and feasibility as defined by the authors if they explained how they evaluated or measured these outcomes. Acceptability was assessed in 32% of publications (69/217) and feasibility in 24% of publications (51/217), with the majority of studies using qualitative methods. All studies found the CBC interventions to be acceptable and all but two studies found the CBC intervention to be feasible. Reasons reported to affect acceptability and feasibility from qualitative studies included provider training, a trustworthy patient-provider relationship, intervention usefulness, and intervention format (group, individual, or telehealth). The findings are summarized in Table 4.

**Table 4:** Acceptability and feasibility results.

Characteristics	Acceptability	Feasibility
Total number of publications	*n* = 69 (32%)	*n* = 51 (24%)
Publication designs	*n* = 28 (41%) qualitative *n* = 22 (32%) quantitative *n* = 19 (28%) mixed methods	*n* = 17 (33%) qualitative *n* = 27 (53%) quantitative *n* = 7 (14%) mixed methods
Primary findings (qualitative and quantitative designs)^a^	*n* = 52 (88%) acceptable *n* = 7 (12%) unclear^b^ no studies deemed unacceptable	*n* = 38 (86%) feasible *n* = 4 (9%) unclear^b^ *n* = 2 (5%) not feasible
Qualitative findings indicating negative outcomes and reasons		Healthy lifestyle group intervention conducted in church and delivered by church community members (Draper et al., 2019). Program fidelity was very poor. A suggested reason was that the unpaid providers did not support a longer training because they were not compensated for their time. Home-based counseling delivered by lay providers to pregnant women with common mental disorders or experience of violence (Abrahams et al., 2022). Lack of confidence among providers, supervisors, and patients in the lay workers’ abilities to provide the counseling.
Qualitative findings indicating a positive outcome^c^	For providers: • Adequate training with opportunities to practice skills (Murray et al., 2018) • Learning new skills during care provision or training that can be applied to other aspects of their life outside of intervention provision (Giusto et al., 2021; Musyoka et al., 2023) For patients: • Perception of intervention usefulness (Carney et al., 2019; Greene et al., 2019; Masulani-Mwale et al., 2019; Green et al., 2020) • Trustworthy patient-provider relationship (Fernando et al., 2021; Giusto et al., 2021) • Role of format: – group format: sense of shared accomplishment, feeling of support and exchange (Draper et al., 2019; Namy et al., 2021; Greene et al., 2022) – individual format: comfort and openness (Carney et al., 2020) – telehealth: anonymity of not having to talk face-to-face and feeling more open and less stigmatized (Green et al., 2020; Gericke et al., 2021; Dambi et al., 2022)	

^a^Protocols are not included. Therefore, the sample sizes are as follows: acceptability, *n* = 59; feasibility, *n* = 44. ^b^These publications did not clearly report on the acceptability or feasibility of results. As such, we were unable to determine the acceptability of the CBC intervention. ^c^For simplification, qualitative reasons for acceptability and feasibility were combined, as they frequently intersected.

Engagement in MH care was only reported in 7/217 publications. Only two publications (Green et al., 2020; Robjant et al., 2022) explicitly used the term engagement as an outcome measure. Green et al.’s (2020) publication described a telehealth intervention using an artificial intelligence app to provide psychosocial support to pregnant women with depression. In this publication, engagement was defined as frequency and duration of app usage. On the other hand, Robjant et al.’s (2022) publication described a trauma therapy intervention for postconflict communities, where engagement was defined as uptake of trauma-specific treatment after referral. The other five publications examined help-seeking as an outcome (Odenwald et al., 2012; Wright and Chiwandira, 2016; Akena et al., 2021; Atilola et al., 2022; Logie et al., 2022), three of which focused on mental health literacy, the ability to recognize the signs and symptoms of mental illness, understand their causes, and identify sources of help. The other two publications focused on treatment seeking behavior, such as seeking consultations with care providers (Odenwald et al., 2012; Wright and Chiwandira, 2016).

#### 3.3.3 Psychosocial outcomes

Sixty-two percent (135/217) of publications included a measure of at least one psychosocial outcome. The most frequently assessed psychosocial outcomes were functional impairment (30/217), social support (29/217), and family functioning (26/217), such as harsh punishment from parents or the quality of parent-infant relationship. Stigma (11/217) and quality of life (10/217), two outcomes that are hypothesized mechanisms for addressing barriers to care through CBC, were assessed in relatively few studies.

## 4 Discussion

The objective of this review was to systematically search, summarize, and categorize CBC models for MH problems for adolescents and adults in Africa, as well as to explore the outcomes evaluated and to identify gaps in the existing literature. The search yielded 206 unique interventions described in 217 publications, with 61% of these studies published since 2018. This suggests a rapidly growing field of research. The unexpected number of interventions and heterogeneity in the populations and conditions addressed limited our ability to provide an in-depth analysis, resulting in a primarily descriptive approach, which falls within the umbrella of a scoping review (Peters et al., 2020). Overall, we identified five major categories of CBC models and eight elements of care. The majority of interventions addressed depression (161/206), engaged lay health providers (122/206), were most commonly located in educational organizations (62/206) and could be classified as psychotherapeutic (144/206). Over 75% (136/175) of publications that measured quantitative outcomes reported a significant reduction in MH problems.

Depression and anxiety were the most frequently addressed conditions, which is unsurprising given the high frequency of their burden (GBD 2019 Mental Disorders Collaborators, 2022). However, only 12/206 of the interventions addressed suicidal behavior and overall 13% (26/206) explicitly excluded participants with suicidal behavior and/or severe MH issues. Given that the highest suicide rates globally occur in Africa (Ilic and Ilic, 2022), more evidence on the potential use of CBC models for treatment of suicidal behavior is urgently needed. The few identified studies examining the effect of CBC models on suicidal behavior showed mixed results (Ertl et al., 2011; Muriungi and Ndetei, 2013; Sherr et al., 2016; Mutamba et al., 2018; Lawrence and Falaye, 2020; Fernando et al., 2021). However, partial CBC models may have a role in reducing high-risk suicidality, alongside traditional health facilities. This could include acute crisis support inside the community (Matheson et al., 2014) or having peers take on certain tasks, such as gatekeeping (identifying signs of suicide risk and connecting individuals with support services) (Bowersox et al., 2021).

More than half (121/206) of the interventions employed lay health workers as part of the care team. Particularly in the case of psychotherapeutic interventions, lay health workers were prominently represented, with almost two thirds of these interventions relying on them to deliver the intervention. Within the cadre of lay health workers, we noted that 33% (40/121) of interventions used a peer provider, which is hypothesized to reduce MH stigma (Sun et al., 2022). This was especially common in interventions that targeted refugees, which used a peer provider, typically current or former refugees, in 40% (8/20) of interventions. However, recruitment and retention of peers can be challenging (Fine et al., 2021). Treatment provision by traditional healers or religious community leaders was very infrequently used, only in three studies (Verduin et al., 2014; Draper et al., 2019; Zoellner et al., 2021), yet in certain African settings these providers are the first point of contact for approximately 50% of patients with a MH or SU problem (Burns and Tomita, 2015). This cadre of provider represents an untapped resource that may be used to scale up evidence-based MH care within communities.

With regard to location, educational organizations such as schools or universities were the most common location of service delivery overall. This was mainly due to interventions addressing adolescents who were often targeted within schools. However, this approach could neglect a substantial proportion of this age group, given that an estimated 30–60% of adolescents ages of 12–17 in Africa are not in school (UNESCO, 2023). To extend the reach of MH interventions for this age group, alternative locations within the community setting must be evaluated. Overall however, we note that the location of service delivery was poorly described in many publications and several authors could not be reached for clarification. This may lead to an incorrect classification of interventions as CBC models or interventions may be missed because of missing location specification. We recommend that future researchers explicitly mention intervention locations, which is crucial for gathering high quality evidence on CBC models.

One identified gap in the literature based on our review is the use of psychopharmacological interventions. Only two interventions implemented such approaches (Odenwald et al., 2012; Ertl et al., 2021). This contrasts with the numerous community-based pharmacological interventions available for other chronic diseases, such as community-based ART-delivery and adherence counseling in HIV care (Geldsetzer et al., 2017; Labhardt et al., 2018; Fox et al., 2019). Although psychological treatment is considered frontline treatment for the majority of MH problems assessed in this review (National Institute for Health and Care Excellence, 2011; Cuijpers et al., 2020), a greater availability of depression medication in the community could help to better treat severe depression, where combined psychological and medication treatment is most effective (National Institute for Health and Care Excellence, 2022). Future research could consider adopting a CBC model similar to the inpatient alcohol detoxification clinic described by Ertl et al. (2021), where specialized staff moved from community to community or task-shifting the delivery of psychopharmacological treatment from physicians to trained nurses in the community, which has been adopted in high-income countries (American Psychiatric Nurse Association, 2019).

Overall, this study has a number of strengths and limitations. An important strength of this scoping review was the extensive search string we developed. CBC is a broad term and used in a variety of ways in the literature. The preliminary abstract review process, whereby we searched for relevant terms related to CBC, allowed us to include a broad range of keywords describing this concept. This is reflected in the number of included studies as well as the heterogeneity of identified models, ultimately leading to comprehensive search results. Similarly, we did not limit intervention selection to only psychotherapeutic or psychopharmacological approach, which allowed us to include studies where MH or SU outcomes were secondary or tertiary aims. This allowed for a broader picture of the models that may be used to improve MH or SU in various community settings.

Our review also has several limitations that must be noted. First, identified publications appeared to have differences in quality, although we did not appraise the methodological rigor of studies and thus cannot draw general conclusions on overall quality of evidence. Future systematic reviews and meta-analyses should therefore be employed to quantify the effect of identified models. Second, some CBC interventions may have been missed because we excluded the gray literature, only included studies in the abstract reviewing process that clearly stated or suggested CBC models, and refrained from forward and backward citation. However, the numerous studies found indicates that our overview of existing models is nevertheless comprehensive, Lastly, we did not extract all relevant data to describe the identified models, such as provider training or supervision, the assessment tools used for the outcome measures, or all implementation outcomes (e.g., adoption, fidelity, cost), which are important indicators for scaling up interventions (Proctor et al., 2011).

Overall, our review highlights that many studies have been conducted to evaluate delivery of MH and SU care in non-traditional health settings in Africa. This fast-growing area of research is not equally distributed, with many countries having no data on the topic. Nevertheless, the existing studies suggest that many CBC models are acceptable, feasible, and possibly effective. Gaps in the literature where future research is likely to be fertile include extension of reach for adolescents, evaluating faith-based leaders as providers, or delivery of psychopharmacological CBC models. Future research should build upon this review by focusing on a narrower topic to facilitate an in-depth analysis of the data available and provide evidence-based recommendations for the urgently required scaleup of MH care.

## Author contributions

FR, M-IH, JB, LF, EF, and NL conceptualized the scoping review. FR, M-IH, and SR performed references screening, data collection and summarization. JH led the literature search and deduplication of sources. FR, M-IH, and JB wrote a first draft of the manuscript and all authors provided critical feedback and gave their approval for the final version. AA and NL were responsible for securing funding for the ComBaCaL project. JB supervised the project. All authors contributed to the article and approved the submitted version.

## Footnotes

^1^In this review we refer to Africa as the entire continent excluding the Northern African region. Table 1 contains a detailed list of countries included in the search.

^2^For brevity reasons “MH” refers to “MH and SU” unless otherwise specified.

^3^www.combacal.org

^4^https://osf.io/2kuq3/

^5^We initially planned to do forward and backward citation searching, as specified in our protocol, because we expected to have relatively few studies that met inclusion criteria. However, given the very large number of studies that were included in full-text review, we did not expect that the additional forward and backward citation searching would yield data that would substantively change the findings of this scoping review.
