# Supplementary material for: Community-based models of care for adolescent and adult depression, suicidal behavior, anxiety, trauma, and substance use in Africa: a scoping review
Source: Front Psychol. 2024 Feb 9;15:1241403. doi: 10.3389/fpsyg.2024.1241403 (PMC10885163; doi:10.3389/fpsyg.2024.1241403)
Supplement: Supplementary file 1 [file Data_Sheet_1.PDF]

Supplementary Material

Community-based Models of Care for Adolescent and Adult Depression, Suicidal Behavior, Anxiety, Trauma, and Substance Use in Africa: A Scoping Review

Fabian Raeber, Maria-Inés Haldemann, Somidha Ray, Jacqueline Huber, Emmanuel Firima, Lucia Gonzalez Fernandez, Alain Amstutz, Felix Gerber, Niklaus D. Labhardt, & Jennifer M. Belus*

*** Correspondence:** Jennifer M. Belus, jennifer.belus@unibas.ch

1 Supplementary Data

Search Strings

For each database the complete search string is a combination of the three concepts (mental health and substance use problems, community-based care, and Africa) using the Boolean operator "AND". The following shows the search string parts for each database.

Search String PsychINFO

Concept 1: Mental health and substance use problems

('addiction'/de OR 'drug dependence'/exp OR 'drug dependence treatment'/exp OR 'methadone maintenance treatment'/exp OR 'cannabis use'/exp OR 'drug abuse'/exp OR 'substance abuse'/de OR 'alcohol consumption'/de OR 'drinking behavior'/de OR 'withdrawal syndrome'/exp OR 'anxiety disorder'/exp OR 'anxiety assessment'/exp OR 'shell shock'/de OR 'depression'/exp OR 'negative thinking'/exp OR 'suicidal behavior'/exp OR ((drug NEAR/3 (dehabituation OR rehabilitation)):ab,ti) OR addict:ab,ti OR addicts:ab,ti OR toxicomani*:ab,ti OR 'narcotism':ab,ti OR 'narcotic depression':ab,ti OR 'opi* crisis':ab,ti OR 'opi* epidemic':ab,ti OR 'physical dependence':ab,ti OR 'chemical dependence':ab,ti OR ((addictive NEXT/3 (behavior* OR behaviour* OR dependenc* OR disorder*)):ab,ti) OR (((polydrug OR multidrug OR multiple OR medication OR non-medical OR nonmedical OR medicine* OR prescription OR nonprescription OR prescribed) NEAR/3 (addiction OR abus* OR misus* OR mis-use OR overu* OR craving OR seeking)):ab,ti) OR NMUPD:ab,it OR 'non-medical use':ab,ti OR 'nonmedical use':ab,ti OR (((substance* OR drug* OR psychoactive* OR psychostimulant* OR benzodiazepine* OR morphin* OR morfin* OR opiate* OR narcotic* OR opioid* OR opium OR heroin* OR diamorphine OR diaphorin OR marihuana OR marijuana OR hashish OR cannabi* OR weed OR amphetamine* OR methamphetamine* OR methylamphetamine* OR extasy OR MDMA OR meth OR hallucinogen* OR cocaine* OR crack OR alcohol* OR ethanol OR drinking OR phencyclidine OR PCP OR 'angel dust' OR londrea OR killerweed OR 'peace pill' OR khat* OR qat* OR glue OR inhalant*) NEAR/3 (disorder* OR dependen* OR addict* OR abus* OR misus* OR mis-us* OR use OR overu* OR habit* OR intoxicat* OR problem* OR craving OR seeking OR sniffing OR overdose* OR psychos* OR abstain* OR abstinence* OR withdraw* OR

cessation)):ab,ti) OR (((marijuana OR marihuana OR hashish OR cannabis OR heroin OR opium) NEAR/3 (smoking OR inhalation)):ab,ti) OR (((harmful OR hazardous OR heavy OR problem* OR chronic OR binge) NEAR/3 (drink* OR alcohol*)):ab,ti) OR (((alcohol OR ethanol) NEAR/3 (intake OR use OR consumption)):ab,ti) OR (((alcohol OR ethanol OR controlled OR behaviour* OR behavior* OR habit* OR pattern* OR social) NEAR/3 drinking):ab,ti) OR alcoholism:ab,ti OR alcoholic*:ab,ti OR dipsomania*:ab,ti OR drunkenness*:ab,ti OR (((abstinence OR withdrawal OR cessation OR discontinuation) NEAR/3 (symptom* OR syndrome* OR seizure* OR hallucin* OR delirium)):ab,ti) OR 'rum fits':ab,ti OR 'delerium tremens':ab,ti OR 'delirium tremens':ab,ti OR ((alcohol* NEXT/3 deliri*):ab,ti) OR anxiet*:ab,ti OR anxious:ab,ti OR 'stress disorder*':ab,ti OR 'distress syndrome*':ab,ti OR 'dystress syndrome*':ab,ti OR 'psych* distress':ab,ti OR cothymia:ab,ti OR obsession:ab,ti OR (((obsessive OR compulsi*) NEAR/3 (disorder* OR disease* OR neurosis OR neuroses OR syndrome* OR reaction* OR behavior OR behaviour OR repetition*)):ab,ti) OR OCD:ab,ti OR 'repetitive behavior':ab,ti OR 'repetitive behaviour':ab,ti OR 'preoccupation neurosis':ab,ti OR 'preoccupation neuroses':ab,ti OR panic:ab,ti OR phobia*:ab,ti OR phobic*:ab,ti OR agoraphobi*:ab,ti OR acrophobi*:ab,ti OR 'fear of height*':ab,ti OR zoophobi*:ab,ti OR 'fear of animal*':ab,ti OR 'fear of snake*':ab,ti OR ophidiophobi*:ab,ti OR ophiophobi*:ab,ti OR 'fear of spider*':ab,ti OR arachnophobi*:ab,ti OR 'fear of blood':ab,ti OR haemophobi*:ab,ti OR hemophobi*:ab,ti OR ((fear NEAR/3 (needle* OR injection*)):ab,ti) OR trypanophobi*:ab,ti OR claustrophobi*:ab,ti OR sociophobi*:ab,ti OR (((posttrauma* OR 'post trauma*' OR combat OR war OR trauma* OR stressor) NEAR/3 (stress OR neurosis OR neuroses OR syndrome* OR disorder* OR psychos*)):ab,ti) OR ptsd:ab,ti OR 'shell shock':ab,ti OR 'combat fatigue':ab,ti OR 'battle fatigue':ab,ti OR traumatized:ab,ti OR traumatised:ab,ti OR ((sexual NEAR/3 trauma*):ab,ti) OR depress*:ab,ti OR melanchol*:ab,ti OR dysphori*:ab,ti OR dysfori*:ab,ti OR dysthymi*:ab,ti OR ((mental NEAR/3 fatigue):ab,ti) OR 'mourning syndrome*':ab,ti OR ((psychos* NEAR/3 involutional):ab,ti) OR 'negative thinking':ab,ti OR 'negative thought*':ab,ti OR 'thinking too much':ab,ti OR 'too many thoughts':ab,ti OR suicid*:ab,ti OR 'self killing':ab,ti OR 'self poisoning':ab,ti OR 'self immolat*':ab,ti OR 'self incineration':ab,ti OR 'selfkilling':ab,ti OR 'selfpoisoning':ab,ti OR 'selfimmolat*':ab,ti OR 'selfincineration':ab,ti OR parasuicid*:ab,ti OR 'fatal attempt*':ab,ti)

Concept 2: Community-based care

('community mental health service'/exp OR 'community mental health center'/exp OR 'school mental health service'/exp OR 'community mental health'/exp OR 'group medical visit'/exp OR (((community OR home OR domicil* OR domestic) NEXT/3 'mental health*'):ab,ti) OR 'deinstitutionalization'/exp OR 'decentralization'/exp OR 'telehealth'/exp OR 'telemetry'/exp OR telehealth:ab,ti OR 'tele health':ab,ti OR telemedic*:ab,ti OR 'tele medic*':ab,ti OR telemetry:ab,ti OR 'tele metry':ab,ti OR biotelemetry:ab,ti OR radiotelemetry:ab,ti OR teleradiometry:ab,ti OR telesensing:ab,ti OR 'tele sensing':ab,ti OR telemonitor*:ab,ti OR 'tele monitor*':ab,ti OR telenurs*:ab,ti OR 'tele nurs*':ab,ti OR teleconsultat*:ab,ti OR 'tele consultat*':ab,ti OR telerehabilitation*:ab,ti OR 'tele rehabilitation*':ab,ti OR (((virtual OR remote OR distance OR distant OR e) NEXT/3 (medic* OR monitor* OR nurs* OR consultat* OR rehabilitation* OR support* OR counsel* OR treatment* OR therap* OR sensing)):ab,ti) OR emedic*:ab,ti OR emonitor*:ab,ti OR enurs*:ab,ti OR econsultat*:ab,ti OR erehabilitation:ab,ti OR esupport*:ab,ti OR ecounsel*:ab,ti OR etreatment*:ab,ti OR etherap*:ab,ti OR esensing:ab,ti OR mhealth:ab,ti OR 'm health':ab,ti OR 'mobile health':ab,ti OR ehealth:ab,ti OR 'e health':ab,ti OR 'e-counseling'/exp OR 'e-mail'/exp OR 'hotline'/exp OR 'interactive voice response system'/exp OR 'internet'/de OR 'web-based intervention'/exp OR 'mobile phone'/exp OR 'telephone'/exp OR 'social media'/exp OR 'telecommunication'/exp OR 'text messaging'/exp OR

'videoconferencing'/exp OR 'web conferencing'/exp OR 'webcast'/exp OR 'podcast'/exp OR 'podcasting'/exp OR 'webinar'/exp OR 'mobile application'/exp OR 'website'/exp OR 'mobile technology'/exp OR 'e mail*':ab,ti OR 'email*':ab,ti OR ((electronic NEXT/1 (mail* OR messaging)):ab,ti) OR hotline*:ab,ti OR 'hot line*':ab,ti OR 'interactive voice response system*':ab,ti OR (((web OR online OR 'on line' OR internet OR video OR virtual OR tele) NEAR/3 (intervention* OR conferenc* OR communication OR seminar* OR information*)):ab,ti) OR internet:ab,ti OR 'world wide web':ab,ti OR www:ab,ti OR (((mobile OR cell OR smart OR portable) NEXT/1 (phone* OR telephone*)):ab,ti) OR 'social media*':ab,ti OR facebook:ab,ti OR twitter:ab,ti OR telecommunication*:ab,ti OR broadcasting:ab,ti OR 'text messag*':ab,ti OR texting:ab,ti OR teleconferenc*:ab,ti OR videoconferenc*:ab,ti OR webconferenc*:ab,ti OR webcast*:ab,ti OR podcast*:ab,ti OR webinar*:ab,ti OR sms:ab,ti OR telephone*:ab,ti OR smartphone*:ab,ti OR (((mobile OR portable OR tablet) NEXT/3 (app OR apps OR application*)):ab,ti) OR website*:ab,ti OR 'web site*':ab,ti OR homepage:ab,ti OR 'mobile technology':ab,ti OR 'ambulatory care'/exp OR decentrali*:ab,ti OR (((ambulatory OR dispensary OR extramural OR 'extra mural' OR outpatient OR 'outpatient health*' OR 'out patient' OR 'out-patient health*' OR 'out of office') NEXT/3 (care OR healthcare OR 'health care' OR service* OR setting* OR monitoring* OR treatment* OR therap*)):ab,ti) OR 'community care'/de OR 'community based rehabilitation'/exp OR 'community health nursing'/exp OR 'community integration'/exp OR 'community program'/exp OR 'community medicine'/exp OR 'community support'/exp OR 'community based distribution'/exp OR 'task shifting'/exp OR 'task sharing'/exp OR (((community OR 'community health*' OR district OR 'public health' OR 'nurse led' OR 'pharmacist led' OR 'nurse based' OR 'pharmacist based' OR 'social service' OR collaborative) NEXT/3 (care OR healthcare OR 'health care' OR nurs* OR integration OR service* OR program* OR medicine OR support* OR engagement OR intervention* OR deliver* OR outreach OR distribution OR network* OR 'participatory method*' OR rehabilitation* OR resource* OR strateg* OR project* OR approach* OR testing OR screening*)):ab,ti) OR 'task shar*':ab,ti OR 'task shift*':ab,ti OR 'home care'/exp OR 'home monitoring'/exp OR 'residential care'/exp OR 'adult day care'/exp OR 'group medical visit':ab,ti OR (((walkway OR weekend OR church) NEXT/3 clinic):ab,ti) OR (((adult OR home OR domicil* OR domestic OR visiting OR residential OR residence) NEAR/3 (care OR healthcare OR 'health care' OR nurs* OR help OR assistance OR service* OR treatment* OR therap* OR 'medication review*' OR pharmac* OR monitor* OR visit* OR agenc* OR testing OR screening* OR diagnos*)):ab,ti) OR homecare:ab,ti OR 'house call*':ab,ti OR 'home diagnostic test'/exp OR 'housebound patient*':ab,ti OR 'adult day care':ab,ti OR 'family service'/exp OR 'family service*':ab,ti OR 'family involve*':ab,ti OR 'family based':ab,ti OR 'family-based':ab,ti OR 'family health*':ab,ti OR 'outpatient care'/exp OR 'out-of-facilit*':ab,ti OR 'health center'/exp OR (((health OR 'health service' OR sanitary) NEXT/3 (center* OR centre* OR clinic* OR institute* OR unit* OR resort* OR facility OR facilities)):ab,ti) OR 'pharmacy (shop)'/de OR 'mail order pharmacy'/exp OR 'online pharmacy'/exp OR 'apothecary':ab,ti OR 'chemist shop*':ab,ti OR 'chemist`s shop*':ab,ti OR 'pharmacies':ab,ti OR 'pharmacy':ab,ti OR 'pharmaceutical service*':ab,ti OR 'rural health care'/exp OR 'school health service'/de OR 'school health nursing'/exp OR 'school based intervention'/exp OR (((rural OR village* OR 'informal settlement*' OR school* OR workplace OR worksite OR 'work place' OR 'work site') NEAR/3 (health* OR care OR medicine OR nurse OR nurses OR nursing OR intervention* OR program* OR approach* OR service* OR strateg*)):ab,ti) OR 'self care'/exp OR 'self monitoring'/exp OR ((self NEXT/3 (care* OR help OR management OR monitoring OR treatment* OR medication OR nurturance OR testing OR measur* OR observation)):ab,ti) OR 'selfcare':ab,ti OR 'selfhelp':ab,ti OR 'selfmanagement':ab,ti OR 'selfmonitor*':ab,ti OR 'selftreatment':ab,ti OR 'selfmedication*':ab,ti OR selftesting:ab,ti OR selfobservation:ab,ti OR 'social network'/exp OR 'social care'/exp OR (((social OR spiritual) NEXT/3 (care OR support OR network* OR service* OR work)):ab,ti) OR 'support group'/exp OR 'support group*':ab,ti OR 'support program*':ab,ti OR

paramedic*:ab,ti OR 'para medic*':ab,ti OR 'non health':ab,ti OR 'non healthcare':ab,ti OR 'non health care':ab,ti OR 'non medical':ab,ti OR (((health OR healthcare OR 'health care' OR medical) NEXT/3 ('support worker*' OR assistant* OR aid*)):ab,ti) OR (((community OR district OR 'public health' OR assistan* OR aid* OR staff* OR manpower OR personnel) NEAR/3 (nurse OR nurses OR nursing OR matron*)):ab,ti) OR 'midlevel health professional*':ab,ti OR 'midlevel health provider*':ab,ti OR 'paramedical personnel'/de OR 'pharmacy technician'/exp OR 'health practitioner'/exp OR 'health visitor'/exp OR 'nurse'/exp OR 'nursing assistant'/exp OR 'nursing staff'/exp OR 'paramedical profession'/exp OR 'pharmacist'/de OR 'community pharmacist'/exp OR 'health auxiliary'/exp OR (((community OR 'community based' OR village* OR rural OR 'informal settlement*' OR lay OR 'allied health' OR 'auxiliary health' OR 'health auxiliary') NEXT/3 (leader* OR worker* OR provider* OR personnel OR profession* OR staff OR aide* OR supporter* OR volunteer* OR advocate* OR team* OR group* OR pharmacist*)):ab,ti) OR 'barefoot doctor*':ab,ti OR 'local supervisor*':ab,ti OR 'caregiver'/exp OR 'caregiver*':ab,ti OR 'care-giver*':ab,ti OR 'peer group'/de OR 'peer education'/de OR 'peer counseling'/exp OR ((peer NEXT/3 (group* OR support OR relation* OR education OR tutoring OR counselling OR counseling OR consult* OR coach*)):ab,ti) OR 'participatory research'/exp OR 'patient participation'/exp OR 'patient empowerment'/exp OR 'patient engagement'/exp OR 'patient activation'/exp OR 'participatory research':ab,ti OR 'patient involvement':ab,ti OR 'patient participation':ab,ti OR 'patient empowerment':ab,ti OR 'patient activation':ab,ti OR 'patient engagement':ab,ti OR (('community'/exp OR 'religion'/exp OR 'church*':ab,ti OR 'workplace'/exp OR 'home'/exp OR 'home environment'/exp OR 'school'/exp OR 'school teacher'/exp) AND ('patient care'/exp OR 'health care'/exp OR 'disease management'/de OR 'drug therapy'/exp)))

Concept 3: Africa

('africa south of the sahara'/exp OR 'africa south of the sahara':ab,ti OR 'sub-sahara* africa*':ab,ti OR 'subsahara* africa*':ab,ti OR 'black africa*':ab,ti OR 'west africa'/exp OR 'west african'/exp OR 'west* africa*':ab,ti OR 'east african'/exp OR 'east* africa*':ab,ti OR 'southern african'/exp OR 'south* africa*':ab,ti OR 'central african'/exp OR 'central africa*':ab,ti OR 'equatorial africa*':ab,ti OR 'middle africa'/exp OR 'middle africa*':ab,ti OR angola*:ab,ti OR benin*:ab,ti OR dahomey:ab,ti OR botswana*:ab,ti OR bechuanaland:ab,ti OR 'burkina faso':ab,ti OR burkin*:ab,ti OR 'upper volta':ab,ti OR burundi*:ab,ti OR urundi:ab,ti OR cameroon*:ab,ti OR camerun:ab,ti OR kamerun:ab,ti OR cameroun:ab,ti OR 'cape verde*':ab,ti OR 'cabo verde':ab,ti OR centrafri*:ab,ti OR 'ubangi-shari':ab,ti OR 'oubangi-shari':ab,ti OR chad*:ab,ti OR tchad:ab,ti OR comoro*:ab,ti OR comores:ab,ti OR comoran:ab,ti OR comorian:ab,ti OR congo*:ab,ti OR kongo:ab,ti OR zaire:ab,ti OR 'cote d`ivoire':ab,ti OR 'ivory coast':ab,ti OR ivorian*:ab,ti OR djibouti*:ab,ti OR 'afars and issas':ab,ti OR eritrea*:ab,ti OR eswatini:ab,ti OR swazi*:ab,ti OR ethiopia*:ab,ti OR abyssinia:ab,ti OR gabon*:ab,ti OR gabun:ab,ti OR gambia*:ab,ti OR senegambia:ab,ti OR ghana*:ab,ti OR 'gold coast':ab,ti OR guinea*:ab,ti OR guinée:ab,ti OR guiné:ab,ti OR 'bissau-guinean':ab,ti OR equatoguinean:ab,ti OR kenya*:ab,ti OR lesotho*:ab,ti OR basutoland:ab,ti OR liberia*:ab,ti OR madagasca*:ab,ti OR malagasy*:ab,ti OR malawi*:ab,ti OR nyasaland:ab,ti OR mali:ab,ti OR malian*:ab,ti OR mauritania*:ab,ti OR mauritanie:ab,ti OR mauritius:ab,ti OR 'république de maurice':ab,ti OR mayotte:ab,ti OR mahoran*:ab,ti OR mozambi*:ab,ti OR mocambique:ab,ti OR namibia*:ab,ti OR niger*:ab,ti OR réunion:ab,ti OR réunionese:ab,ti OR rwanda*:ab,ti OR ruanda*:ab,ti OR senegal*:ab,ti OR 'seychellene'/exp OR seychell*:ab,ti OR 'sierra leon*':ab,ti OR somali*:ab,ti OR 'sudanese'/exp OR sudan*:ab,ti OR tanzania*:ab,ti OR tansania*:ab,ti OR tanganyika:ab,ti OR zanzibar:ab,ti OR togo*:ab,ti OR uganda*:ab,ti OR zambia*:ab,ti OR sambia*:ab,ti OR

zimbabwe*:ab,ti OR rhodesia:ab,ti OR luanda:ab,ti OR lubango:ab,ti OR cabinda:ab,ti OR 'porto-novo':ab,ti OR cotonou:ab,ti OR 'abomey-calavi':ab,ti OR gaborone:ab,ti OR ouagadougou:ab,ti OR 'bobo-dioulasso':ab,ti OR bujumbura:ab,ti OR yaounde:ab,ti OR douala:ab,ti OR praia:ab,ti OR bangui:ab,ti OR n`djamena:ab,ti OR ndjamena:ab,ti OR moroni:ab,ti OR brazzaville:ab,ti OR 'pointe-noire':ab,ti OR kinshasa:ab,ti OR 'mbuji-mayi':ab,ti OR lubumbashi:ab,ti OR kananga:ab,ti OR kisangani:ab,ti OR bukavu:ab,ti OR yamoussoukro:ab,ti OR abidjan:ab,ti OR malabo:ab,ti OR asmara:ab,ti OR mbabane:ab,ti OR 'addis ababa':ab,ti OR libreville:ab,ti OR banjul:ab,ti OR accra:ab,ti OR kumasi:ab,ti OR 'sekondi takoradi':ab,ti OR conakry:ab,ti OR bissau:ab,ti OR nairobi:ab,ti OR mombasa:ab,ti OR mombassa:ab,ti OR maseru:ab,ti OR monrovia:ab,ti OR antananarivo:ab,ti OR lilongwe:ab,ti OR 'blantyre-limbe':ab,ti OR bamako:ab,ti OR nouakchott:ab,ti OR 'port louis':ab,ti OR maputo:ab,ti OR matola:ab,ti OR nampula:ab,ti OR windhoek:ab,ti OR niamey:ab,ti OR abuja:ab,ti OR lagos:ab,ti OR kano:ab,ti OR ibadan:ab,ti OR 'port harcourt':ab,ti OR kigali:ab,ti OR 'sao tome*':ab,ti OR dakar:ab,ti OR freetown:ab,ti OR mogadishu:ab,ti OR hargeisa:ab,ti OR hargeysa:ab,ti OR pretoria:ab,ti OR 'cape town':ab,ti OR johannesburg:ab,ti OR soweto:ab,ti OR durban:ab,ti OR 'port elizabeth':ab,ti OR 'west rand':ab,ti OR juba:ab,ti OR khartoum:ab,ti OR nyala:ab,ti OR 'dar es salaam':ab,ti OR dodoma:ab,ti OR mwanza:ab,ti OR lome:ab,ti OR kampala:ab,ti OR lusaka:ab,ti OR harare:ab,ti) NOT (('animal'/de OR 'animal experiment'/exp OR 'nonhuman'/de) NOT ('human'/exp OR 'human experiment'/de)) NOT [conference abstract]/lim

Search String Embase

Concept 1: Mental health and substance use problems

('addiction'/de OR 'drug dependence'/exp OR 'drug dependence treatment'/exp OR 'methadone maintenance treatment'/exp OR 'cannabis use'/exp OR 'drug abuse'/exp OR 'substance abuse'/de OR 'alcohol consumption'/de OR 'drinking behavior'/de OR 'withdrawal syndrome'/exp OR 'anxiety disorder'/exp OR 'anxiety assessment'/exp OR 'shell shock'/de OR 'depression'/exp OR 'negative thinking'/exp OR 'suicidal behavior'/exp OR ((drug NEAR/3 (dehabituation OR rehabilitation)):ab,ti) OR addict:ab,ti OR addicts:ab,ti OR toxicomani*:ab,ti OR 'narcotism':ab,ti OR 'narcotic depression':ab,ti OR 'opi* crisis':ab,ti OR 'opi* epidemic':ab,ti OR 'physical dependence':ab,ti OR 'chemical dependence':ab,ti OR ((addictive NEXT/3 (behavior* OR behaviour* OR dependenc* OR disorder*)):ab,ti) OR (((polydrug OR multidrug OR multiple OR medication OR non-medical OR nonmedical OR medicine* OR prescription OR nonprescription OR prescribed) NEAR/3 (addiction OR abus* OR misus* OR mis-use OR overu* OR craving OR seeking)):ab,ti) OR NMUPD:ab,it OR 'non-medical use':ab,ti OR 'nonmedical use':ab,ti OR (((substance* OR drug* OR psychoactive* OR psychostimulant* OR benzodiazepine* OR morphin* OR morfin* OR opiate* OR narcotic* OR opioid* OR opium OR heroin* OR diamorphine OR diaphorin OR marihuana OR marijuana OR hashish OR cannabi* OR weed OR amphetamine* OR methamphetamine* OR methylamphetamine* OR extasy OR MDMA OR meth OR hallucinogen* OR cocaine* OR crack OR alcohol* OR ethanol OR drinking OR phencyclidine OR PCP OR 'angel dust' OR londrea OR killerweed OR 'peace pill' OR khat* OR qat* OR glue OR inhalant*) NEAR/3 (disorder* OR dependen* OR addict* OR abus* OR misus* OR mis-us* OR use OR overu* OR habit* OR intoxicat* OR problem* OR craving OR seeking OR sniffing OR overdose* OR psychos* OR abstain* OR abstinence* OR withdraw* OR cessation)):ab,ti) OR (((marijuana OR marihuana OR hashish OR cannabis OR heroin OR opium) NEAR/3 (smoking OR inhalation)):ab,ti) OR (((harmful OR hazardous OR heavy OR problem* OR chronic OR binge) NEAR/3 (drink* OR alcohol*)):ab,ti) OR (((alcohol OR ethanol) NEAR/3 (intake OR use OR consumption)):ab,ti) OR (((alcohol OR ethanol OR controlled OR behaviour* OR

behavior* OR habit* OR pattern* OR social) NEAR/3 drinking):ab,ti) OR alcoholism:ab,ti OR alcoholic*:ab,ti OR dipsomania*:ab,ti OR drunkenness*:ab,ti OR (((abstinence OR withdrawal OR cessation OR discontinuation) NEAR/3 (symptom* OR syndrome* OR seizure* OR hallucin* OR delirium)):ab,ti) OR 'rum fits':ab,ti OR 'delerium tremens':ab,ti OR 'delirium tremens':ab,ti OR ((alcohol* NEXT/3 deliri*):ab,ti) OR anxiet*:ab,ti OR anxious:ab,ti OR 'stress disorder*':ab,ti OR 'distress syndrome*':ab,ti OR 'dystress syndrome*':ab,ti OR 'psych* distress':ab,ti OR cothymia:ab,ti OR obsession:ab,ti OR (((obsessive OR compulsi*) NEAR/3 (disorder* OR disease* OR neurosis OR neuroses OR syndrome* OR reaction* OR behavior OR behaviour OR repetition*)):ab,ti) OR OCD:ab,ti OR 'repetitive behavior':ab,ti OR 'repetitive behaviour':ab,ti OR 'preoccupation neurosis':ab,ti OR 'preoccupation neuroses':ab,ti OR panic:ab,ti OR phobia*:ab,ti OR phobic*:ab,ti OR agoraphobi*:ab,ti OR acrophobi*:ab,ti OR 'fear of height*':ab,ti OR zoophobi*:ab,ti OR 'fear of animal*':ab,ti OR 'fear of snake*':ab,ti OR ophidiophobi*:ab,ti OR ophiophobi*:ab,ti OR 'fear of spider*':ab,ti OR arachnophobi*:ab,ti OR 'fear of blood':ab,ti OR haemophobi*:ab,ti OR hemophobi*:ab,ti OR ((fear NEAR/3 (needle* OR injection*)):ab,ti) OR trypanophobi*:ab,ti OR claustrophobi*:ab,ti OR sociophobi*:ab,ti OR (((posttrauma* OR 'post trauma*' OR combat OR war OR trauma* OR stressor) NEAR/3 (stress OR neurosis OR neuroses OR syndrome* OR disorder* OR psychos*)):ab,ti) OR ptsd:ab,ti OR 'shell shock':ab,ti OR 'combat fatigue':ab,ti OR 'battle fatigue':ab,ti OR traumatized:ab,ti OR traumatised:ab,ti OR ((sexual NEAR/3 trauma*):ab,ti) OR depress*:ab,ti OR melanchol*:ab,ti OR dysphori*:ab,ti OR dysfori*:ab,ti OR dysthymi*:ab,ti OR ((mental NEAR/3 fatigue):ab,ti) OR 'mourning syndrome*':ab,ti OR ((psychos* NEAR/3 involutional):ab,ti) OR 'negative thinking':ab,ti OR 'negative thought*':ab,ti OR 'thinking too much':ab,ti OR 'too many thoughts':ab,ti OR suicid*:ab,ti OR 'self killing':ab,ti OR 'self poisoning':ab,ti OR 'self immolat*':ab,ti OR 'self incineration':ab,ti OR 'selfkilling':ab,ti OR 'selfpoisoning':ab,ti OR 'selfimmolat*':ab,ti OR 'selfincineration':ab,ti OR parasuicid*:ab,ti OR 'fatal attempt*':ab,ti)

Concept 2: Community-based care

('community mental health service'/exp OR 'community mental health center'/exp OR 'school mental health service'/exp OR 'community mental health'/exp OR 'group medical visit'/exp OR (((community OR home OR domicil* OR domestic) NEXT/3 'mental health*'):ab,ti) OR 'deinstitutionalization'/exp OR 'decentralization'/exp OR 'telehealth'/exp OR 'telemetry'/exp OR telehealth:ab,ti OR 'tele health':ab,ti OR telemedic*:ab,ti OR 'tele medic*':ab,ti OR telemetry:ab,ti OR 'tele metry':ab,ti OR biotelemetry:ab,ti OR radiotelemetry:ab,ti OR teleradiometry:ab,ti OR telesensing:ab,ti OR 'tele sensing':ab,ti OR telemonitor*:ab,ti OR 'tele monitor*':ab,ti OR telenurs*:ab,ti OR 'tele nurs*':ab,ti OR teleconsultat*:ab,ti OR 'tele consultat*':ab,ti OR telerehabilitation*:ab,ti OR 'tele rehabilitation*':ab,ti OR (((virtual OR remote OR distance OR distant OR e) NEXT/3 (medic* OR monitor* OR nurs* OR consultat* OR rehabilitation* OR support* OR counsel* OR treatment* OR therap* OR sensing)):ab,ti) OR emedic*:ab,ti OR emonitor*:ab,ti OR enurs*:ab,ti OR econsultat*:ab,ti OR erehabilitation:ab,ti OR esupport*:ab,ti OR ecounsel*:ab,ti OR etreatment*:ab,ti OR etherap*:ab,ti OR esensing:ab,ti OR mhealth:ab,ti OR 'm health':ab,ti OR 'mobile health':ab,ti OR ehealth:ab,ti OR 'e health':ab,ti OR 'e-counseling'/exp OR 'e-mail'/exp OR 'hotline'/exp OR 'interactive voice response system'/exp OR 'internet'/de OR 'web-based intervention'/exp OR 'mobile phone'/exp OR 'telephone'/exp OR 'social media'/exp OR 'telecommunication'/exp OR 'text messaging'/exp OR 'videoconferencing'/exp OR 'web conferencing'/exp OR 'webcast'/exp OR 'podcast'/exp OR 'podcasting'/exp OR 'webinar'/exp OR 'mobile application'/exp OR 'website'/exp OR 'mobile technology'/exp OR 'e mail*':ab,ti OR 'email*':ab,ti OR ((electronic NEXT/1 (mail* OR messaging)):ab,ti) OR hotline*:ab,ti OR 'hot line*':ab,ti OR 'interactive voice response system*':ab,ti

OR (((web OR online OR 'on line' OR internet OR video OR virtual OR tele) NEAR/3 (intervention* OR conferenc* OR communication OR seminar* OR information*)):ab,ti) OR internet:ab,ti OR 'world wide web':ab,ti OR www:ab,ti OR (((mobile OR cell OR smart OR portable) NEXT/1 (phone* OR telephone*)):ab,ti) OR 'social media*':ab,ti OR facebook:ab,ti OR twitter:ab,ti OR telecommunication*:ab,ti OR broadcasting:ab,ti OR 'text messag*':ab,ti OR texting:ab,ti OR teleconferenc*:ab,ti OR videoconferenc*:ab,ti OR webconferenc*:ab,ti OR webcast*:ab,ti OR podcast*:ab,ti OR webinar*:ab,ti OR sms:ab,ti OR telephone*:ab,ti OR smartphone*:ab,ti OR (((mobile OR portable OR tablet) NEXT/3 (app OR apps OR application*)):ab,ti) OR website*:ab,ti OR 'web site*':ab,ti OR homepage:ab,ti OR 'mobile technology':ab,ti OR 'ambulatory care'/exp OR decentrali*:ab,ti OR (((ambulatory OR dispensary OR extramural OR 'extra mural' OR outpatient OR 'outpatient health*' OR 'out patient' OR 'out-patient health*' OR 'out of office') NEXT/3 (care OR healthcare OR 'health care' OR service* OR setting* OR monitoring* OR treatment* OR therap*)):ab,ti) OR 'community care'/de OR 'community based rehabilitation'/exp OR 'community health nursing'/exp OR 'community integration'/exp OR 'community program'/exp OR 'community medicine'/exp OR 'community support'/exp OR 'community based distribution'/exp OR 'task shifting'/exp OR 'task sharing'/exp OR (((community OR 'community health*' OR district OR 'public health' OR 'nurse led' OR 'pharmacist led' OR 'nurse based' OR 'pharmacist based' OR 'social service' OR collaborative) NEXT/3 (care OR healthcare OR 'health care' OR nurs* OR integration OR service* OR program* OR medicine OR support* OR engagement OR intervention* OR deliver* OR outreach OR distribution OR network* OR 'participatory method*' OR rehabilitation* OR resource* OR strateg* OR project* OR approach* OR testing OR screening*)):ab,ti) OR 'task shar*':ab,ti OR 'task shift*':ab,ti OR 'home care'/exp OR 'home monitoring'/exp OR 'residential care'/exp OR 'adult day care'/exp OR 'group medical visit':ab,ti OR (((walkway OR weekend OR church) NEXT/3 clinic):ab,ti) OR (((adult OR home OR domicil* OR domestic OR visiting OR residential OR residence) NEAR/3 (care OR healthcare OR 'health care' OR nurs* OR help OR assistance OR service* OR treatment* OR therap* OR 'medication review*' OR pharmac* OR monitor* OR visit* OR agenc* OR testing OR screening* OR diagnos*)):ab,ti) OR homecare:ab,ti OR 'house call*':ab,ti OR 'home diagnostic test'/exp OR 'housebound patient*':ab,ti OR 'adult day care':ab,ti OR 'family service'/exp OR 'family service*':ab,ti OR 'family involve*':ab,ti OR 'family based':ab,ti OR 'family-based':ab,ti OR 'family health*':ab,ti OR 'outpatient care'/exp OR 'out-of-facilit*':ab,ti OR 'health center'/exp OR (((health OR 'health service' OR sanitary) NEXT/3 (center* OR centre* OR clinic* OR institute* OR unit* OR resort* OR facility OR facilities)):ab,ti) OR 'pharmacy (shop)'/de OR 'mail order pharmacy'/exp OR 'online pharmacy'/exp OR 'apothecary':ab,ti OR 'chemist shop*':ab,ti OR 'chemist`s shop*':ab,ti OR 'pharmacies':ab,ti OR 'pharmacy':ab,ti OR 'pharmaceutical service*':ab,ti OR 'rural health care'/exp OR 'school health service'/de OR 'school health nursing'/exp OR 'school based intervention'/exp OR (((rural OR village* OR 'informal settlement*' OR school* OR workplace OR worksite OR 'work place' OR 'work site') NEAR/3 (health* OR care OR medicine OR nurse OR nurses OR nursing OR intervention* OR program* OR approach* OR service* OR strateg*)):ab,ti) OR 'self care'/exp OR 'self monitoring'/exp OR ((self NEXT/3 (care* OR help OR management OR monitoring OR treatment* OR medication OR nurturance OR testing OR measur* OR observation)):ab,ti) OR 'selfcare':ab,ti OR 'selfhelp':ab,ti OR 'selfmanagement':ab,ti OR 'selfmonitor*':ab,ti OR 'selftreatment':ab,ti OR 'selfmedication*':ab,ti OR selftesting:ab,ti OR selfobservation:ab,ti OR 'social network'/exp OR 'social care'/exp OR (((social OR spiritual) NEXT/3 (care OR support OR network* OR service* OR work)):ab,ti) OR 'support group'/exp OR 'support group*':ab,ti OR 'support program*':ab,ti OR paramedic*:ab,ti OR 'para medic*':ab,ti OR 'non health':ab,ti OR 'non healthcare':ab,ti OR 'non health care':ab,ti OR 'non medical':ab,ti OR (((health OR healthcare OR 'health care' OR medical) NEXT/3 ('support worker*' OR assistant* OR aid*)):ab,ti) OR (((community OR district OR 'public health' OR assistan* OR aid* OR staff* OR manpower OR personnel) NEAR/3 (nurse OR nurses OR nursing OR

matron*)):ab,ti) OR 'midlevel health professional*':ab,ti OR 'midlevel health provider*':ab,ti OR 'paramedical personnel'/de OR 'pharmacy technician'/exp OR 'health practitioner'/exp OR 'health visitor'/exp OR 'nurse'/exp OR 'nursing assistant'/exp OR 'nursing staff'/exp OR 'paramedical profession'/exp OR 'pharmacist'/de OR 'community pharmacist'/exp OR 'health auxiliary'/exp OR (((community OR 'community based' OR village* OR rural OR 'informal settlement*' OR lay OR 'allied health' OR 'auxiliary health' OR 'health auxiliary') NEXT/3 (leader* OR worker* OR provider* OR personnel OR profession* OR staff OR aide* OR supporter* OR volunteer* OR advocate* OR team* OR group* OR pharmacist*)):ab,ti) OR 'barefoot doctor*':ab,ti OR 'local supervisor*':ab,ti OR 'caregiver'/exp OR 'caregiver*':ab,ti OR 'care-giver*':ab,ti OR 'peer group'/de OR 'peer education'/de OR 'peer counseling'/exp OR ((peer NEXT/3 (group* OR support OR relation* OR education OR tutoring OR counselling OR counseling OR consult* OR coach*)):ab,ti) OR 'participatory research'/exp OR 'patient participation'/exp OR 'patient empowerment'/exp OR 'patient engagement'/exp OR 'patient activation'/exp OR 'participatory research':ab,ti OR 'patient involvement':ab,ti OR 'patient participation':ab,ti OR 'patient empowerment':ab,ti OR 'patient activation':ab,ti OR 'patient engagement':ab,ti OR (('community'/exp OR 'religion'/exp OR 'church*':ab,ti OR 'workplace'/exp OR 'home'/exp OR 'home environment'/exp OR 'school'/exp OR 'school teacher'/exp) AND ('patient care'/exp OR 'health care'/exp OR 'disease management'/de OR 'drug therapy'/exp)))

Concept 3: Africa

('africa south of the sahara'/exp OR 'africa south of the sahara':ab,ti OR 'sub-sahara* africa*':ab,ti OR 'subsahara* africa*':ab,ti OR 'black africa*':ab,ti OR 'west africa'/exp OR 'west african'/exp OR 'west* africa*':ab,ti OR 'east african'/exp OR 'east* africa*':ab,ti OR 'southern african'/exp OR 'south* africa*':ab,ti OR 'central african'/exp OR 'central africa*':ab,ti OR 'equatorial africa*':ab,ti OR 'middle africa'/exp OR 'middle africa*':ab,ti OR angola*:ab,ti OR benin*:ab,ti OR dahomey:ab,ti OR botswana*:ab,ti OR bechuanaland:ab,ti OR 'burkina faso':ab,ti OR burkin*:ab,ti OR 'upper volta':ab,ti OR burundi*:ab,ti OR urundi:ab,ti OR cameroon*:ab,ti OR camerun:ab,ti OR kamerun:ab,ti OR cameroun:ab,ti OR 'cape verde*':ab,ti OR 'cabo verde':ab,ti OR centrafri*:ab,ti OR 'ubangi-shari':ab,ti OR 'oubangi-shari':ab,ti OR chad*:ab,ti OR tchad:ab,ti OR comoro*:ab,ti OR comores:ab,ti OR comoran:ab,ti OR comorian:ab,ti OR congo*:ab,ti OR kongo:ab,ti OR zaire:ab,ti OR 'cote d`ivoire':ab,ti OR 'ivory coast':ab,ti OR ivorian*:ab,ti OR djibouti*:ab,ti OR 'afars and issas':ab,ti OR eritrea*:ab,ti OR eswatini:ab,ti OR swazi*:ab,ti OR ethiopia*:ab,ti OR abyssinia:ab,ti OR gabon*:ab,ti OR gabun:ab,ti OR gambia*:ab,ti OR senegambia:ab,ti OR ghana*:ab,ti OR 'gold coast':ab,ti OR guinea*:ab,ti OR guinée:ab,ti OR guiné:ab,ti OR 'bissau-guinean':ab,ti OR equatoguinean:ab,ti OR kenya*:ab,ti OR lesotho*:ab,ti OR basutoland:ab,ti OR liberia*:ab,ti OR madagasca*:ab,ti OR malagasy*:ab,ti OR malawi*:ab,ti OR nyasaland:ab,ti OR mali:ab,ti OR malian*:ab,ti OR mauritania*:ab,ti OR mauritanie:ab,ti OR mauritius:ab,ti OR 'république de maurice':ab,ti OR mayotte:ab,ti OR mahoran*:ab,ti OR mozambi*:ab,ti OR mocambique:ab,ti OR namibia*:ab,ti OR niger*:ab,ti OR réunion:ab,ti OR réunionese:ab,ti OR rwanda*:ab,ti OR ruanda*:ab,ti OR senegal*:ab,ti OR 'seychellene'/exp OR seychell*:ab,ti OR 'sierra leon*':ab,ti OR somali*:ab,ti OR 'sudanese'/exp OR sudan*:ab,ti OR tanzania*:ab,ti OR tansania*:ab,ti OR tanganyika:ab,ti OR zanzibar:ab,ti OR togo*:ab,ti OR uganda*:ab,ti OR zambia*:ab,ti OR sambia*:ab,ti OR zimbabwe*:ab,ti OR rhodesia:ab,ti OR luanda:ab,ti OR lubango:ab,ti OR cabinda:ab,ti OR 'porto-novo':ab,ti OR cotonou:ab,ti OR 'abomey-calavi':ab,ti OR gaborone:ab,ti OR ouagadougou:ab,ti OR 'bobo-dioulasso':ab,ti OR bujumbura:ab,ti OR yaounde:ab,ti OR douala:ab,ti OR praia:ab,ti OR bangui:ab,ti OR n`djamena:ab,ti OR ndjamena:ab,ti OR moroni:ab,ti OR brazzaville:ab,ti OR 'pointe-

noire':ab,ti OR kinshasa:ab,ti OR 'mbuji-mayi':ab,ti OR lubumbashi:ab,ti OR kananga:ab,ti OR kisangani:ab,ti OR bukavu:ab,ti OR yamoussoukro:ab,ti OR abidjan:ab,ti OR malabo:ab,ti OR asmara:ab,ti OR mbabane:ab,ti OR 'addis ababa':ab,ti OR libreville:ab,ti OR banjul:ab,ti OR accra:ab,ti OR kumasi:ab,ti OR 'sekondi takoradi':ab,ti OR conakry:ab,ti OR bissau:ab,ti OR nairobi:ab,ti OR mombasa:ab,ti OR mombassa:ab,ti OR maseru:ab,ti OR monrovia:ab,ti OR antananarivo:ab,ti OR lilongwe:ab,ti OR 'blantyre-limbe':ab,ti OR bamako:ab,ti OR nouakchott:ab,ti OR 'port louis':ab,ti OR maputo:ab,ti OR matola:ab,ti OR nampula:ab,ti OR windhoek:ab,ti OR niamey:ab,ti OR abuja:ab,ti OR lagos:ab,ti OR kano:ab,ti OR ibadan:ab,ti OR 'port harcourt':ab,ti OR kigali:ab,ti OR 'sao tome*':ab,ti OR dakar:ab,ti OR freetown:ab,ti OR mogadishu:ab,ti OR hargeisa:ab,ti OR hargeysa:ab,ti OR pretoria:ab,ti OR 'cape town':ab,ti OR johannesburg:ab,ti OR soweto:ab,ti OR durban:ab,ti OR 'port elizabeth':ab,ti OR 'west rand':ab,ti OR juba:ab,ti OR khartoum:ab,ti OR nyala:ab,ti OR 'dar es salaam':ab,ti OR dodoma:ab,ti OR mwanza:ab,ti OR lome:ab,ti OR kampala:ab,ti OR lusaka:ab,ti OR harare:ab,ti) NOT (('animal'/de OR 'animal experiment'/exp OR 'nonhuman'/de) NOT ('human'/exp OR 'human experiment'/de))
NOT [conference abstract]/lim

Search String Scopus

Concept 1: Mental health and substance use problems

((TITLE-ABS(drug) W/3 (dehabituation OR rehabilitation)) OR TITLE-ABS(addict) OR TITLE-ABS(addicts) OR TITLE-ABS(toxicomani*) OR TITLE-ABS(narcotism) OR TITLE-ABS("narcotic depression") OR TITLE-ABS("opi* crisis") OR TITLE-ABS("opi* epidemic") OR TITLE-ABS("physical dependence") OR TITLE-ABS("chemical dependence") OR (TITLE-ABS(addictive) Pre/3 TITLE-ABS(behavior* OR behaviour* OR dependenc* OR disorder*)) OR (TITLE-ABS(polydrug OR multidrug OR multiple OR medication OR non-medical OR nonmedical OR medicine* OR prescription OR nonprescription OR prescribed) W/3 (addiction OR abus* OR misus* OR mis-use OR overu* OR craving OR seeking)) OR TITLE-ABS(NMUPD) OR TITLE-ABS("non-medical 'use'" OR "nonmedical 'use'") OR (TITLE-ABS((substance* OR drug* OR psychoactive* OR psychostimulant* OR benzodiazepine* OR morphin* OR morfin* OR opiate* OR narcotic* OR opioid* OR opium OR heroin* OR diamorphine OR diaphorin OR marihuana OR marijuana OR hashish OR cannabi* OR weed OR amphetamine* OR methamphetamine* OR methylamphetamine* OR extasy OR MDMA OR meth OR hallucinogen* OR cocaine* OR crack OR alcohol* OR ethanol OR drinking OR phencyclidine OR PCP OR "angel dust" OR londrea OR killerweed OR "peace pill" OR khat* OR qat* OR glue OR inhalant*) W/3 (disorder* OR dependen* OR addict* OR abus* OR misus* OR mis-us* OR 'use' OR overu* OR habit* OR intoxicat* OR problem* OR craving OR seeking OR sniffing OR overdose* OR psychos* OR abstain* OR abstinence* OR withdraw* OR cessation))) OR (TITLE-ABS((marijuana OR marihuana OR hashish OR cannabis OR heroin OR opium) W/3 (smoking OR inhalation))) OR (TITLE-ABS((harmful OR hazardous OR heavy OR problem* OR chronic OR binge) W/3 (drink* OR alcohol*))) OR (TITLE-ABS((alcohol OR ethanol) W/3 (intake OR 'use' OR consumption))) OR (TITLE-ABS((alcohol OR ethanol OR controlled OR behaviour* OR behavior* OR habit* OR pattern* OR social) W/3 drinking)) OR TITLE-ABS(alcoholism) OR TITLE-ABS(alcoholic*) OR TITLE-ABS(dipsomania*) OR TITLE-ABS(drunkenness*) OR (TITLE-ABS((abstinence OR withdrawal OR cessation OR discontinuation) W/3 (symptom* OR syndrome* OR seizure* OR hallucin* OR delirium))) OR TITLE-ABS("rum fits") OR TITLE-ABS("delerium tremens") OR TITLE-ABS("delirium tremens") OR (TITLE-ABS(alcohol* Pre/3 deliri*)) OR TITLE-ABS(anxiet*) OR TITLE-ABS(anxious) OR TITLE-ABS("stress disorder*") OR TITLE-ABS("distress syndrome*") OR TITLE-ABS("dystress

syndrome*") OR TITLE-ABS("psych* distress") OR TITLE-ABS(cothymia) OR TITLE-ABS(obsession) OR (TITLE-ABS((obsessive OR compulsi*) W/3 (disorder* OR disease* OR neurosis OR neuroses OR syndrome* OR reaction* OR behavior OR behaviour OR repetition*))) OR TITLE-ABS(OCD) OR TITLE-ABS("repetitive behavior") OR TITLE-ABS("repetitive behaviour") OR TITLE-ABS("preoccupation neurosis") OR TITLE-ABS("preoccupation neuroses") OR TITLE-ABS(panic) OR TITLE-ABS(phobia*) OR TITLE-ABS(phobic*) OR TITLE-ABS(agoraphobi*) OR TITLE-ABS(acrophobi*) OR TITLE-ABS("fear of height*") OR TITLE-ABS(zoophobi*) OR TITLE-ABS("fear of animal*") OR TITLE-ABS("fear of snake*") OR TITLE-ABS(ophidiophobi*) OR TITLE-ABS(ophiophobi*) OR TITLE-ABS("fear of spider*") OR TITLE-ABS(arachnophobi*) OR TITLE-ABS("fear of blood") OR TITLE-ABS(haemophobi*) OR TITLE-ABS(hemophobi*) OR (TITLE-ABS(fear W/3 (needle* OR injection*))) OR TITLE-ABS(trypanophobi*) OR TITLE-ABS(claustrophobi*) OR TITLE-ABS(sociophobi*) OR (TITLE-ABS((posttrauma* OR "post trauma*" OR combat OR war OR trauma* OR stressor) W/3 (stress OR neurosis OR neuroses OR syndrome* OR disorder* OR psychos*))) OR TITLE-ABS(ptsd) OR TITLE-ABS("shell shock") OR TITLE-ABS("combat fatigue") OR TITLE-ABS("battle fatigue") OR TITLE-ABS(traumatized) OR TITLE-ABS(traumatised) OR (TITLE-ABS(sexual W/3 trauma*)) OR TITLE-ABS(depress*) OR TITLE-ABS(melanchol*) OR TITLE-ABS(dysphori*) OR TITLE-ABS(dysfori*) OR TITLE-ABS(dysthymi*) OR (TITLE-ABS(mental W/3 fatigue)) OR TITLE-ABS("mourning syndrome*") OR (TITLE-ABS(psychos* W/3 involutional)) OR TITLE-ABS("negative thinking") OR TITLE-ABS("negative thought*") OR TITLE-ABS("thinking too much") OR TITLE-ABS("too many thoughts") OR TITLE-ABS(suicid*) OR TITLE-ABS("self killing") OR TITLE-ABS("self poisoning") OR TITLE-ABS("self immolat*") OR TITLE-ABS("self incineration") OR TITLE-ABS(selfkilling) OR TITLE-ABS(selfpoisoning) OR TITLE-ABS(selfimmolat*) OR TITLE-ABS(selfincineration) OR TITLE-ABS(parasuicid*) OR TITLE-ABS("fatal attempt*"))

Concept 2: Community-based care

(TITLE-ABS((community OR home OR domicil* OR domestic) W/3 '"mental health*'") OR TITLE-ABS("Telehealth") OR TITLE-ABS("Tele-health") OR TITLE-ABS("Telemedic*") OR TITLE-ABS("Tele-medic*") OR TITLE-ABS("telemetry") OR TITLE-ABS("tele-metry") OR TITLE-ABS("biotelemetry") OR TITLE-ABS("radiotelemetry") OR TITLE-ABS("teleradiometry") OR TITLE-ABS("telesensing") OR TITLE-ABS("tele-sensing") OR TITLE-ABS("telemonitor*") OR TITLE-ABS("tele-monitor*") OR TITLE-ABS("telenurs*") OR TITLE-ABS("tele-nurs*") OR TITLE-ABS("teleconsultat*") OR TITLE-ABS("tele-consultat*") OR TITLE-ABS("telerehabilitation*") OR TITLE-ABS("tele-rehabilitation*") OR ((TITLE-ABS("virtual" OR "remote" OR "distance" OR "distant" OR "e")) W/3 (TITLE-ABS("medic*" OR "monitor*" OR "nurs*" OR "consultat*" OR "rehabilitation*" OR "support*" OR "counsel*" OR "treatment*" OR "therap*" OR "sensing"))) OR TITLE-ABS("emedic*") OR TITLE-ABS("emonitor*") OR TITLE-ABS("enurs*") OR TITLE-ABS("econsultat*") OR TITLE-ABS("erehabilitation") OR TITLE-ABS("esupport*") OR TITLE-ABS("ecounsel*") OR TITLE-ABS("etreatment*") OR TITLE-ABS("etherap*") OR TITLE-ABS("esensing") OR TITLE-ABS("Mhealth") OR TITLE-ABS("M-health") OR TITLE-ABS("mobile health") OR TITLE-ABS("Ehealth") OR TITLE-ABS("E-health") OR TITLE-ABS("e mail*") OR TITLE-ABS("email*") OR (TITLE-ABS("electronic") Pre/1 (TITLE-ABS("mail*" OR "messaging"))) OR TITLE-ABS("hotline*") OR TITLE-ABS("hot line*") OR TITLE-ABS("interactive voice response system*") OR ((TITLE-ABS("web" OR "online" OR "on-line" OR "internet" OR "video" OR "virtual" OR "tele")) W/3 (TITLE-ABS("intervention*" OR "conferenc*" OR "communication" OR "seminar*" OR "information*"))) OR TITLE-ABS("internet")

OR TITLE-ABS("world wide web") OR TITLE-ABS("www") OR ((TITLE-ABS("mobile" OR "cell" OR "smart" OR "portable")) Pre/1 (TITLE-ABS("phone*" OR "telephone*"))) OR TITLE-ABS("social media*") OR TITLE-ABS("facebook") OR TITLE-ABS("twitter") OR TITLE-ABS("telecommunication*") OR TITLE-ABS("broadcasting") OR TITLE-ABS("text messag*") OR TITLE-ABS("texting") OR TITLE-ABS("teleconferenc*") OR TITLE-ABS("videoconferenc*") OR TITLE-ABS("webconferenc*") OR TITLE-ABS("webcast*") OR TITLE-ABS("podcast*") OR TITLE-ABS("webinar*") OR TITLE-ABS("SMS") OR TITLE-ABS("telephone*") OR TITLE-ABS("smartphone*") OR ((TITLE-ABS("mobile" OR "portable" OR "tablet")) W/3 (TITLE-ABS("app" OR "apps" OR "application*"))) OR TITLE-ABS("website") OR TITLE-ABS("web site") OR TITLE-ABS("homepage") OR TITLE-ABS("mobile technology") OR TITLE-ABS("decentrali*") OR ((TITLE-ABS("ambulatory" OR "dispensary" OR "extramural" OR "extra-mural" OR "outpatient" OR "outpatient health*" OR "out-patient" OR "out-patient health*" OR "out-of-office")) W/3 (TITLE-ABS("care" OR "healthcare" OR "health care" OR "service*" OR "setting*" OR "monitoring*" OR "treatment*" OR "therap*"))) OR ((TITLE-ABS("community" OR "community health*" OR "district" OR "public health" OR "nurse-led" OR "pharmacist-led" OR "nurse-based" OR "pharmacist-based" OR "social service" OR "collaborative")) W/3 (TITLE-ABS("care" OR "healthcare" OR "health care" OR "nurs*" OR "integration" OR "service*" OR "program*" OR "medicine" OR "support*" OR "engagement" OR "intervention*" OR "deliver*" OR "outreach" OR "distribution" OR "network*" OR "participatory method*" OR "rehabilitation*" OR "resource*" OR "strateg*" OR "project*" OR "approach*" OR "testing" OR "screening*"))) OR TITLE-ABS("task shar*") OR TITLE-ABS("task shift*") OR TITLE-ABS ("group medical visit") OR ((TITLE-ABS("adult" OR "home" OR "domicil*" OR "domestic" OR "visiting" OR "residential" OR "residence")) W/3 (TITLE-ABS("care" OR "healthcare" OR ”health care" OR "nurs*" OR "help" OR "assistance" OR "service*" OR "treatment*" OR "therap*" OR "medication review*" OR "pharmac*" OR "monitor*" OR "visit*" OR "agenc*" OR "testing" OR "screening*" OR "diagnos*"))) OR TITLE-ABS("homecare") OR TITLE-ABS("house call*") OR TITLE-ABS("housebound patient*") OR TITLE-ABS("adult day care") OR TITLE-ABS("family service*") OR TITLE-ABS("family involve*") OR TITLE-ABS("family based") OR TITLE-ABS("family-based") OR TITLE-ABS("family health*") OR TITLE-ABS("out-of-facilit*") OR (TITLE-ABS("health" OR "health service" OR "sanitary") W/3 (TITLE-ABS("center*" OR "centre*" OR "clinic*" OR "institute*" OR "unit*" OR "resort*" OR "facility" OR "facilities"))) OR (TITLE-ABS((walkway OR weekend OR church) W/3 clinic)) OR TITLE-ABS("apothecary") OR TITLE-ABS("chemist shop*") OR TITLE-ABS("chemist`s shop*") OR TITLE-ABS("pharmacies") OR TITLE-ABS("pharmacy") OR TITLE-ABS("pharmaceutical service*") OR (TITLE-ABS(village* OR "informal settlement*" OR "rural" OR "school*" OR "workplace" OR "worksite" OR "work-place" OR "work-site") W/3 TITLE-ABS("health*" OR "care" OR "medicine" OR "nurse" OR "nurses" OR "nursing" OR "intervention*" OR "program*" OR "approach*" OR "service*" OR "strateg*")) OR (TITLE-ABS("self") W/3 (TITLE-ABS("care*" OR "help" OR "management" OR "monitoring" OR "treatment*" OR "medication" OR "nurturance" OR "testing" OR "measur*" OR "observation"))) OR TITLE-ABS("selfcare") OR TITLE-ABS("selfhelp") OR TITLE-ABS("selfmanagement") OR TITLE-ABS("selfmonitor*") OR TITLE-ABS("selftreatment") OR TITLE-ABS("selfmedication*") OR TITLE-ABS(selftesting) OR TITLE-ABS(selfobservation) OR (TITLE-ABS("social" OR "spiritual") W/3 (TITLE-ABS("care" OR "support" OR "network*" OR "service*" OR "work"))) OR TITLE-ABS("support group*") OR TITLE-ABS("support program*") OR TITLE-ABS("paramedic*") OR TITLE-ABS("para-medic*") OR TITLE-ABS("non-health") OR TITLE-ABS("non-healthcare") OR TITLE-ABS("non-health care") OR TITLE-ABS("non-medical") OR (TITLE-ABS("health" OR "healthcare" OR "health care" OR "medical") W/3 (TITLE-ABS("support worker*" OR "assistant*" OR "aid*"))) OR ((TITLE-ABS("community" OR "district" OR "public health" OR "assistan*" OR "aid*" OR "staff*" OR "manpower" OR "personnel")) W/4 (TITLE-

ABS("nurse" OR "nurses" OR "nursing" OR "matron*"))) OR TITLE-ABS("midlevel health professional*") OR TITLE-ABS("midlevel health provider*") OR (TITLE-ABS("community" OR "community based" OR "village*" OR "rural" OR "informal settlement*" OR "lay" OR "allied health" OR "auxiliary health" OR "health auxiliary") W/3 (TITLE-ABS("leader" OR "worker*" OR "provider*" OR "personnel" OR "profession*" OR "staff" OR "aide*" OR "supporter*" OR "volunteer*" OR "advocate*" OR "team*" OR "group*" OR "pharmacist*"))) OR TITLE-ABS("barefoot doctor*") OR TITLE-ABS("local supervisor*") OR TITLE-ABS("caregiver*") OR TITLE-ABS("care giver*") OR (TITLE-ABS("peer") W/3 (TITLE-ABS("group*" OR "support" OR "relation*" OR "education" OR "tutoring" OR "counselling" OR "counseling" OR "consult*" OR "coach*"))) OR TITLE-ABS("participatory research") OR TITLE-ABS("patient involvement") OR TITLE-ABS("patient participation") OR TITLE-ABS("patient empowerment") OR TITLE-ABS("patient activation") OR TITLE-ABS("patient engagement"))

Concept 3: Africa

(TITLE-ABS("africa south of the sahara") OR TITLE-ABS("sub-sahara* africa*") OR TITLE-ABS("subsahara* africa*") OR TITLE-ABS("black africa*") OR TITLE-ABS("west* africa*") OR TITLE-ABS("east* africa*") OR TITLE-ABS("south* africa*") OR TITLE-ABS("central africa*") OR TITLE-ABS("equatorial africa*") OR TITLE-ABS("middle africa*") OR TITLE-ABS("angola*") OR TITLE-ABS("benin*") OR TITLE-ABS("dahomey") OR TITLE-ABS("botswana*") OR TITLE-ABS("bechuanaland") OR TITLE-ABS("burkina faso") OR TITLE-ABS("burkin*") OR TITLE-ABS("upper volta") OR TITLE-ABS("burundi*") OR TITLE-ABS("urundi") OR TITLE-ABS("cameroon*") OR TITLE-ABS("camerun") OR TITLE-ABS("kamerun") OR TITLE-ABS("cameroun") OR TITLE-ABS("cape verde*") OR TITLE-ABS("cabo verde") OR TITLE-ABS("centrafri*") OR TITLE-ABS("ubangi-shari") OR TITLE-ABS("oubangi-shari") OR TITLE-ABS("chad*") OR TITLE-ABS("tchad") OR TITLE-ABS("comoro*") OR TITLE-ABS("comores") OR TITLE-ABS("comoran") OR TITLE-ABS("comorian") OR TITLE-ABS("congo*") OR TITLE-ABS("kongo") OR TITLE-ABS("zaire") OR TITLE-ABS("cote d`ivoire") OR TITLE-ABS("ivory coast") OR TITLE-ABS("ivorian*") OR TITLE-ABS("djibouti*") OR TITLE-ABS("afars and issas") OR TITLE-ABS("eritrea*") OR TITLE-ABS("eswatini") OR TITLE-ABS("swazi*") OR TITLE-ABS("ethiopia*") OR TITLE-ABS("abyssinia") OR TITLE-ABS("gabon*") OR TITLE-ABS("gabun") OR TITLE-ABS("gambia*") OR TITLE-ABS("senegambia") OR TITLE-ABS("ghana*") OR TITLE-ABS("gold coast") OR TITLE-ABS("guinea*") OR TITLE-ABS("guinee") OR TITLE-ABS("guine") OR TITLE-ABS("bissau-guinean") OR TITLE-ABS("equatoguinean") OR TITLE-ABS("kenya*") OR TITLE-ABS("lesotho*") OR TITLE-ABS("basutoland") OR TITLE-ABS("liberia*") OR TITLE-ABS("madagasca*") OR TITLE-ABS("malagasy*") OR TITLE-ABS("malawi*") OR TITLE-ABS("nyasaland") OR TITLE-ABS("mali") OR TITLE-ABS("malian*") OR TITLE-ABS("mauritania*") OR TITLE-ABS("mauritanie") OR TITLE-ABS("mauritius") OR TITLE-ABS("republique de maurice") OR TITLE-ABS("mayotte") OR TITLE-ABS("mahoran*") OR TITLE-ABS("mozambi*") OR TITLE-ABS("mocambique") OR TITLE-ABS("namibia*") OR TITLE-ABS("niger*") OR TITLE-ABS("reunion") OR TITLE-ABS("reunionese") OR TITLE-ABS("rwanda*") OR TITLE-ABS("ruanda*") OR TITLE-ABS("senegal*") OR TITLE-ABS("seychell*") OR TITLE-ABS("sierra leon*") OR TITLE-ABS("somali*") OR TITLE-ABS("sudan*") OR TITLE-ABS("tanzania*") OR TITLE-ABS("tansania*") OR TITLE-ABS("tanganyika") OR TITLE-ABS("zanzibar") OR TITLE-ABS("togo*") OR TITLE-ABS("uganda*") OR TITLE-ABS("zambia*") OR TITLE-ABS("sambia*") OR TITLE-ABS("zimbabwe*") OR TITLE-ABS("rhodesia") OR TITLE-ABS("luanda") OR TITLE-

ABS("lubango") OR TITLE-ABS("cabinda") OR TITLE-ABS("porto-novo") OR TITLE-ABS("cotonou") OR TITLE-ABS("abomey-calavi") OR TITLE-ABS("gaborone") OR TITLE-ABS("ouagadougou") OR TITLE-ABS("bobo-dioulasso") OR TITLE-ABS("bujumbura") OR TITLE-ABS("yaounde") OR TITLE-ABS("douala") OR TITLE-ABS("praia") OR TITLE-ABS("bangui") OR TITLE-ABS("n`djamena") OR TITLE-ABS("ndjamena") OR TITLE-ABS("moroni") OR TITLE-ABS("brazzaville") OR TITLE-ABS("pointe-noire") OR TITLE-ABS("kinshasa") OR TITLE-ABS("mbuji-mayi") OR TITLE-ABS("lubumbashi") OR TITLE-ABS("kananga") OR TITLE-ABS("kisangani") OR TITLE-ABS("bukavu") OR TITLE-ABS("yamoussoukro") OR TITLE-ABS("abidjan") OR TITLE-ABS("malabo") OR TITLE-ABS("asmara") OR TITLE-ABS("mbabane") OR TITLE-ABS("addis ababa") OR TITLE-ABS("libreville") OR TITLE-ABS("banjul") OR TITLE-ABS("accra") OR TITLE-ABS("kumasi") OR TITLE-ABS("sekondi takoradi") OR TITLE-ABS("conakry") OR TITLE-ABS("bissau") OR TITLE-ABS("nairobi") OR TITLE-ABS("mombasa") OR TITLE-ABS("mombassa") OR TITLE-ABS("maseru") OR TITLE-ABS("monrovia") OR TITLE-ABS("antananarivo") OR TITLE-ABS("lilongwe") OR TITLE-ABS("blantyre-limbe") OR TITLE-ABS("bamako") OR TITLE-ABS("nouakchott") OR TITLE-ABS("port louis") OR TITLE-ABS("maputo") OR TITLE-ABS("matola") OR TITLE-ABS("nampula") OR TITLE-ABS("windhoek") OR TITLE-ABS("niamey") OR TITLE-ABS("abuja") OR TITLE-ABS("lagos") OR TITLE-ABS("kano") OR TITLE-ABS("ibadan") OR TITLE-ABS("port harcourt") OR TITLE-ABS("kigali") OR TITLE-ABS("sao tome*") OR TITLE-ABS("dakar") OR TITLE-ABS("freetown") OR TITLE-ABS("mogadishu") OR TITLE-ABS("hargeisa") OR TITLE-ABS("hargeysa") OR TITLE-ABS("pretoria") OR TITLE-ABS("cape town") OR TITLE-ABS("johannesburg") OR TITLE-ABS("Soweto") OR TITLE-ABS("durban") OR TITLE-ABS("port elizabeth") OR TITLE-ABS("west rand") OR TITLE-ABS("juba") OR TITLE-ABS("khartoum") OR TITLE-ABS("nyala") OR TITLE-ABS("dar es salaam") OR TITLE-ABS("dodoma") OR TITLE-ABS("mwanza") OR TITLE-ABS("lome") OR TITLE-ABS("kampala") OR TITLE-ABS("lusaka") OR TITLE-ABS("harare"))

Search String CINAHL

Concept 1: Mental health and substance use problems

((MH "Behavior, Addictive") OR (MH "Substance Use Disorders+") OR (MH "Opioid Epidemic") OR (MH "Drug-Seeking Behavior+") OR (MH "Craving+") OR (MH "Drug Rehabilitation Programs+") OR (MH "Smoking Cessation") OR (MH "Drinking Behavior+") OR (MH "anxiety disorder+") OR (MH "Catastrophization+") OR ("Psychological Distress+") OR (MH "Compulsive Behavior") OR (MH "Separation Anxiety") OR (MH "Depression+") OR ("Psychomotor Agitation+") OR (MH "Suicide+") OR (((TI drug OR AB drug) N3 ((TI dehabituation OR AB dehabituation) OR (TI rehabilitation OR AB rehabilitation)))) OR (TI addict OR AB addict) OR (TI addicts OR AB addicts) OR (TI toxicomani* OR AB toxicomani*) OR (TI narcotism OR AB narcotism) OR (TI "narcotic depression" OR AB "narcotic depression") OR (TI "opi* crisis" OR AB "opi* crisis") OR (TI "opi* epidemic" OR AB "opi* epidemic") OR (TI "physical dependence" OR AB "physical dependence") OR (TI "chemical dependence" OR AB "chemical dependence") OR (((TI addictive OR AB addictive) W3 ((TI behavior* OR AB behaviour*) OR (TI behaviour* OR AB behaviour*) OR (TI dependenc* OR AB dependenc*) OR (TI disorder* OR AB disorder*)))) OR ((((TI polydrug OR AB polydrug) OR (TI multidrug OR AB multidrug) OR (TI multiple OR AB multiple) OR (TI medication OR AB medication) OR (TI non-medical OR AB non-medical) OR (TI nonmedical OR AB nonmedical) OR (TI medicine* OR AB medicine*) OR (TI prescription OR AB prescription) OR (TI nonprescription OR AB nonprescription) OR (TI prescribed OR AB prescribed)) N3 ((TI addiction OR AB addiction) OR (TI abus* OR AB abus*) OR (TI misus* OR AB misus*) OR (TI mis-use OR AB mis-use) OR

(TI overu* OR AB overu*) OR (TI craving OR AB craving) OR (TI seeking OR AB seeking)))) OR (TI NMUPD OR AB NMUPD) OR ((TI "non-medical 'use'" OR AB "non-medical 'use'") OR (TI "nonmedical 'use'" OR AB "nonmedical 'use'")) OR ((((TI substance* OR AB substance*) OR (TI drug* OR AB drug*) OR (TI psychoactive* OR AB psychoactive*) OR (TI psychostimulant* OR AB psychostimulant*) OR (TI benzodiazepine* OR AB benzodiazepine*) OR (TI morphin* OR AB morphin*) OR (TI morfin* OR AB morfin*) OR (TI opiate* OR AB opiate*) OR (TI narcotic* OR AB narcotic*) OR (TI opioid* OR AB opioid*) OR (TI opium OR AB opium) OR (TI heroin* OR AB heroin*) OR (TI diamorphine OR AB diamorphine) OR (TI diaphorin OR AB diaphorin) OR (TI marihuana OR AB marihuana) OR (TI marijuana OR AB marijuana) OR (TI hashish OR AB hashish) OR (TI cannabi* OR AB cannabi*) OR (TI weed OR AB weed) OR (TI amphetamine* OR AB amphetamine*) OR (TI methamphetamine* OR AB methamphetamine*) OR (TI methylamphetamine* OR AB methylamphetamine*) OR (TI extasy OR AB extasy) OR (TI MDMA OR AB MDMA) OR (TI meth OR AB meth) OR (TI hallucinogen* OR AB hallucinogen*) OR (TI cocaine* OR AB cocaine*) OR (TI crack OR AB crack) OR (TI alcohol* OR AB alcohol*) OR (TI ethanol OR AB ethanol) OR (TI drinking OR AB drinking) OR (TI phencyclidine OR AB phencyclidine) OR (TI PCP OR AB PCP) OR (TI "angel dust" OR AB "angel dust") OR (TI londrea OR AB londrea) OR (TI killerweed OR AB killerweed) OR (TI "peace pill" OR AB "peace pill") OR (TI khat* OR AB khat*) OR (TI qat* OR AB qat*) OR (TI glue OR AB glue) OR (TI inhalant* OR AB inhalant*)) N3 ((TI disorder* OR AB disorder*) OR (TI dependen* OR AB dependen*) OR (TI addict* OR AB addict*) OR (TI abus* OR AB abus*) OR (TI misus* OR AB misus*) OR (TI mis-us* OR AB mis-us*) OR (TI 'use' OR AB 'use') OR (TI overu* OR AB overu*) OR (TI habit* OR AB habit*) OR (TI intoxicat* OR AB intoxicat*) OR (TI problem* OR AB problem*) OR (TI craving OR AB craving) OR (TI seeking OR AB seeking) OR (TI sniffing OR AB sniffing) OR (TI overdose* OR AB overdose*) OR (TI psychos* OR AB psychos*) OR (TI abstain* OR AB abstain*) OR (TI abstinence* OR AB abstinence*) OR (TI withdraw* OR AB withdraw*) OR (TI cessation OR AB cessation)))) OR ((((TI marijuana OR AB marijuana) OR (TI marihuana OR AB marihuana) OR (TI hashish OR AB hashish) OR (TI cannabis OR AB cannabis) OR (TI heroin OR AB heroin) OR (TI opium OR AB opium)) N3 ((TI smoking OR AB smoking) OR (TI inhalation OR AB inhalation)))) OR ((((TI harmful OR AB harmful) OR (TI hazardous OR AB hazardous) OR (TI heavy OR AB heavy) OR (TI problem* OR AB problem*) OR (TI chronic OR AB chronic) OR (TI binge OR AB binge)) N3 ((TI drink* OR AB drink*) OR (TI alcohol* OR AB alcohol*)))) OR ((((TI alcohol OR AB alcohol) OR (TI ethanol OR AB ethanol)) N3 ((TI intake OR AB intake) OR (TI 'use' OR AB 'use') OR (TI consumption OR AB consumption)))) OR ((((TI alcohol OR AB alcohol) OR (TI ethanol OR AB ethanol) OR (TI controlled OR AB controlled) OR (TI behaviour* OR AB behaviour*) OR (TI behavior* OR AB behavior*) OR (TI habit* OR AB habit*) OR (TI pattern* OR AB pattern*) OR (TI social OR AB social)) N3 (TI drinking OR AB drinking))) OR (TI alcoholism OR AB alcoholism) OR (TI alcoholic* OR AB alcoholic*) OR (TI dipsomania* OR AB dipsomania*) OR (TI drunkenness* OR AB drunkenness*) OR ((((TI abstinence OR AB abstinence) OR (TI withdrawal OR AB withdrawal) OR (TI cessation OR AB cessation) OR (TI discontinuation OR AB discontinuation)) N3 ((TI symptom* OR AB symptom*) OR (TI syndrome* OR AB syndrome*) OR (TI seizure* OR AB seizure*) OR (TI hallucin* OR AB hallucin*) OR (TI delirium OR AB delirium)))) OR (TI "rum fits" OR AB "rum fits") OR (TI "delerium tremens" OR AB "delerium tremens") OR (TI "delirium tremens" OR AB "delirium tremens") OR (((TI alcohol* OR AB alcohol*) W3 (TI deliri* OR AB deliri*))) OR (TI anxiet* OR AB anxiet*) OR (TI anxious OR AB anxious) OR (TI "stress disorder*" OR AB "stress disorder*") OR (TI "distress syndrome*" OR AB "distress syndrome*") OR (TI "dystress syndrome*" OR AB "dystress syndrome*") OR (TI "psych* distress" OR AB "psych* distress") OR (TI cothymia OR AB cothymia) OR (TI obsession OR AB obsession) OR ((((TI obsessive OR AB obsessive) OR

(TI compulsi* OR AB compulsi*)) N3 ((TI disorder* OR AB disorder*) OR (TI disease* OR AB disease*) OR (TI neurosis OR AB neurosis) OR (TI neuroses OR AB neuroses) OR (TI syndrome* OR AB syndrome*) OR (TI reaction* OR AB reaction*) OR (TI behavior OR AB behavior) OR (TI behaviour OR AB behaviour) OR (TI repetition* OR AB repetition*)))) OR (TI OCD OR AB OCD) OR (TI "repetitive behavior" OR AB "repetitive behavior") OR (TI "repetitive behaviour" OR AB "repetitive behaviour") OR (TI "preoccupation neurosis" OR AB "preoccupation neurosis") OR (TI "preoccupation neuroses" OR AB "preoccupation neuroses") OR (TI panic OR AB panic) OR (TI phobia* OR AB phobia*) OR (TI phobic* OR AB phobic*) OR (TI agoraphobi* OR AB agoraphobi*) OR (TI acrophobi* OR AB acrophobi*) OR (TI "fear of height*" OR AB "fear of height*") OR (TI zoophobi* OR AB zoophobi*) OR (TI "fear of animal*" OR AB "fear of animal*") OR (TI "fear of snake*" OR AB "fear of snake*") OR (TI ophidiophobi* OR AB ophidiophobi*) OR (TI ophiophobi* OR AB ophiophobi*) OR (TI "fear of spider*" OR AB "fear of spider*") OR (TI arachnophobi* OR AB arachnophobi*) OR (TI "fear of blood" OR AB "fear of blood") OR (TI haemophobi* OR AB haemophobi*) OR (TI hemophobi* OR AB hemophobi*) OR (((TI fear OR AB fear) N3 ((TI needle* OR AB needle*) OR (TI injection* OR AB injection*)))) OR (TI trypanophobi* OR AB trypanophobi*) OR (TI claustrophobi* OR AB claustrophobi*) OR (TI sociophobi* OR AB sociophobi*) OR ((((TI posttrauma* OR AB posttrauma*) OR (TI "post trauma*" OR AB "post trauma*") OR (TI combat OR AB combat) OR (TI war OR AB war) OR (TI trauma* OR AB trauma*) OR (TI stressor OR AB stressor)) N3 ((TI stress OR AB stress) OR (TI neurosis OR AB neurosis) OR (TI neuroses OR AB neuroses) OR (TI syndrome* OR AB syndrome*) OR (TI disorder* OR AB disorder*) OR (TI psychos* OR AB psychos*)))) OR (TI ptsd OR AB ptsd) OR (TI "shell shock" OR AB "shell shock") OR (TI "combat fatigue" OR AB "combat fatigue") OR (TI "battle fatigue" OR AB "battle fatigue") OR (TI traumatized OR AB traumatized) OR (TI traumatised OR AB traumatised) OR (((TI sexual OR AB sexual) N3 (TI trauma* OR AB trauma*))) OR (TI depress* OR AB depress*) OR (TI melanchol* OR AB melanchol*) OR (TI dysphori* OR AB dysphori*) OR (TI dysfori* OR AB dysfori*) OR (TI dysthymi* OR AB dysthymi*) OR (((TI mental OR AB mental) N3 (TI fatigue OR AB fatigue))) OR (TI "mourning syndrome*" OR AB "mourning syndrome*") OR (((TI psychos* OR AB psychos*) N3 (TI involutional OR AB involutional))) OR (TI "negative thinking" OR AB "negative thinking") OR (TI "negative thought*" OR AB "negative thought*") OR (TI "thinking too much" OR AB "thinking too much") OR (TI "too many thoughts" OR AB "too many thoughts") OR (TI suicid* OR AB suicid*) OR (TI "self killing" OR AB "self killing") OR (TI "self poisoning" OR AB "self poisoning") OR (TI "self immolat*" OR AB "self immolat*") OR (TI "self incineration" OR AB "self incineration") OR (TI selfkilling OR AB selfkilling) OR (TI selfpoisoning OR AB selfpoisoning) OR (TI selfimmolat* OR AB selfimmolat*) OR (TI selfincineration OR AB selfincineration) OR (TI parasuicid* OR AB parasuicid*) OR (TI "fatal attempt*" OR AB "fatal attempt*"))

Concept 2: Community-based care

((MH "Community Mental Health Services+") OR (MH "School Mental Health Services+") OR (MH "Home Health Care+") OR (((TI community OR AB community) OR (TI home OR AB home) OR (TI domicil* OR AB domicil*) OR (TI domestic OR AB domestic)) W3 (TI "'mental health*'" OR AB "'mental health*'")) OR (MH "Deinstitutionalization") OR (MH "Decentralization") OR (MH "Telecommunications+") OR (MH "Telemetry") OR (TI Telehealth OR AB Telehealth) OR (TI Tele-health OR AB Tele-health) OR (TI Telemedic* OR AB Telemedic*) OR (TI Tele-medic* OR AB Tele-medic*) OR (TI telemetry OR AB telemetry) OR (TI tele-metry OR AB tele-metry) OR (TI biotelemetry OR AB biotelemetry) OR (TI radiotelemetry OR AB radiotelemetry) OR (TI teleradiometry OR AB teleradiometry) OR (TI telesensing OR AB telesensing) OR (TI tele-sensing

OR AB tele-sensing) OR (TI telemonitor* OR AB telemonitor*) OR (TI tele-monitor* OR AB tele-monitor*) OR (TI telenurs* OR AB telenurs*) OR (TI tele-nurs* OR AB tele-nurs*) OR (TI teleconsultat* OR AB teleconsultat*) OR (TI tele-consultat* OR AB tele-consultat*) OR (TI telerehabilitation* OR AB telerehabilitation*) OR (TI tele-rehabilitation* OR AB tele-rehabilitation*) OR (((TI virtual OR AB virtual) OR (TI remote OR AB remote) OR (TI distance OR AB distance) OR (TI distant OR AB distant) OR (TI e OR AB e)) W3 ((TI medic* OR AB medic*) OR (TI monitor* OR AB monitor*) OR (TI nurs* OR AB nurs*) OR (TI consultat* OR AB consultat*) OR (TI rehabilitation* OR AB rehabilitation*) OR (TI support* OR AB support*) OR (TI counsel* OR AB counsel*) OR (TI treatment* OR AB treatment*) OR (TI therap* OR AB therap*) OR (TI sensing OR AB sensing))) OR (TI emedic* OR AB emedic*) OR (TI emonitor* OR AB emonitor*) OR (TI enurs* OR AB enurs*) OR (TI econsultat* OR AB econsultat*) OR (TI erehabilitation OR AB erehabilitation) OR (TI esupport* OR AB esupport*) OR (TI ecounsel* OR AB ecounsel*) OR (TI etreatment* OR AB etreatment*) OR (TI etherap* OR AB etherap*) OR (TI esensing OR AB esensing) OR (TI MHealth OR AB MHealth) OR (TI M-health OR AB M-health) OR (TI "mobile health" OR AB "mobile health") OR (TI Ehealth OR AB Ehealth) OR (TI E-health OR AB E-health) OR (MH "Telephone Information Services") OR (MH "Mobile Applications") OR (TI "e mail*" OR AB "e mail*") OR (TI email* OR AB email*) OR ((TI electronic OR AB electronic) W1 ((TI mail* OR AB mail*) OR (TI messaging OR AB messaging))) OR (TI hotline* OR AB hotline*) OR (TI "hot line*" OR AB "hot line*") OR (TI "interactive voice response system*" OR AB "interactive voice response system*") OR (((TI web OR AB web) OR (TI online OR AB online) OR (TI on-line OR AB on-line) OR (TI internet OR AB internet) OR (TI video OR AB video) OR (TI virtual OR AB virtual) OR (TI tele OR AB tele)) N3 ((TI intervention* OR AB intervention*) OR (TI conferenc* OR AB conferenc*) OR (TI communication OR AB communication) OR (TI seminar* OR AB seminar*) OR (TI information* OR AB information*))) OR (TI internet OR AB internet) OR (TI "world wide web" OR AB "world wide web") OR (TI www OR AB www) OR (((TI mobile OR AB mobile) OR (TI cell OR AB cell) OR (TI smart OR AB smart) OR (TI portable OR AB portable)) W1 ((TI phone* OR AB phone*) OR (TI telephone* OR AB telephone*))) OR (TI "social media*" OR AB "social media*") OR (TI facebook OR AB facebook) OR (TI twitter OR AB twitter) OR (TI telecommunication* OR AB telecommunication*) OR (TI broadcasting OR AB broadcasting) OR (TI "text messag*" OR AB "text messag*") OR (TI texting OR AB texting) OR (TI teleconferenc* OR AB teleconferenc*) OR (TI videoconferenc* OR AB videoconferenc*) OR (TI webconferenc* OR AB webconferenc*) OR (TI webcast* OR AB webcast*) OR (TI podcast* OR AB podcast*) OR (TI webinar* OR AB webinar*) OR (TI SMS OR AB SMS) OR (TI telephone* OR AB telephone*) OR (TI smartphone* OR AB smartphone*) OR (((TI mobile OR AB mobile) OR (TI portable OR AB portable) OR (TI tablet OR AB tablet)) W3 ((TI app OR AB app) OR (TI apps OR AB apps) OR (TI application* OR AB application*))) OR (TI website OR AB website) OR (TI "web site" OR AB "web site") OR (TI homepage OR AB homepage) OR (TI "mobile technology" OR AB "mobile technology") OR (MH "ambulatory Care") OR (MH "ambulatory Care Information Systems") OR (MH "ambulatory Care Facilities") OR (MH "ambulatory Care Nursing") OR (TI decentrali* OR AB decentrali*) OR (((TI ambulatory OR AB ambulatory) OR (TI dispensary OR AB dispensary) OR (TI extramural OR AB extramural) OR (TI extra-mural OR AB extra-mural) OR (TI outpatient OR AB outpatient) OR (TI "outpatient health*" OR AB "outpatient health*") OR (TI out-patient OR AB out-patient) OR (TI "out-patient health*" OR AB "out-patient health*") OR (TI out-of-office OR AB out-of-office)) W3 ((TI care OR AB care) OR (TI healthcare OR AB healthcare) OR (TI health-care OR AB health-care) OR (TI service* OR AB service*) OR (TI setting* OR AB setting*) OR (TI monitoring* OR AB monitoring*) OR (TI treatment* OR AB treatment*) OR (TI therap* OR AB therap*))) OR (MH "Community Health Services+") OR (MH "community medicine") OR (MH "rehabilitation") OR (MH

"Community Programs") OR (MH "Uncompensated Care") OR (((TI community OR AB community) OR (TI "community health*" OR AB "community health*") OR (TI district OR AB district) OR (TI "public health" OR AB "public health") OR (TI nurse-led OR AB nurse-led) OR (TI pharmacist-led OR AB pharmacist-led) OR (TI nurse-based OR AB nurse-based) OR (TI pharmacist-based OR AB pharmacist-based) OR (TI "social service" OR AB "social service") OR (TI collaborative OR AB collaborative)) W3 ((TI care OR AB care) OR (TI healthcare OR AB healthcare) OR (TI health-care OR AB health-care) OR (TI nurs* OR AB nurs*) OR (TI integration OR AB integration) OR (TI service* OR AB service*) OR (TI program* OR AB program*) OR (TI medicine OR AB medicine) OR (TI support* OR AB support*) OR (TI engagement OR AB engagement) OR (TI intervention* OR AB intervention*) OR (TI deliver* OR AB deliver*) OR (TI outreach OR AB outreach) OR (TI distribution OR AB distribution) OR (TI network* OR AB network*) OR (TI "participatory method*" OR AB "participatory method*") OR (TI rehabilitation* OR AB rehabilitation*) OR (TI resource* OR AB resource*) OR (TI strateg* OR AB strateg*) OR (TI project* OR AB project*) OR (TI approach* OR AB approach*) OR (TI testing OR AB testing) OR (TI screening* OR AB screening*))) OR (TI "task shar*" OR AB "task shar*") OR (TI "task shift*" OR AB "task shift*") OR (MH "Day Care") OR (MH "Residential Care+") OR (TI "group medical visit" OR AB "group medical visit") OR (((TI adult OR AB adult) OR (TI home OR AB home) OR (TI domicil* OR AB domicil*) OR (TI domestic OR AB domestic) OR (TI visiting OR AB visiting) OR (TI residential OR AB residential) OR (TI residence OR AB residence)) N3 ((TI care OR AB care) OR (TI healthcare OR AB healthcare) OR (TI health-care OR AB health-care) OR (TI nurs* OR AB nurs*) OR (TI help OR AB help) OR (TI assistance OR AB assistance) OR (TI service* OR AB service*) OR (TI treatment* OR AB treatment*) OR (TI therap* OR AB therap*) OR (TI "medication review*" OR AB "medication review*") OR (TI pharmac* OR AB pharmac*) OR (TI monitor* OR AB monitor*) OR (TI visit* OR AB visit*) OR (TI agenc* OR AB agenc*) OR (TI testing OR AB testing) OR (TI screening* OR AB screening*) OR (TI diagnos* OR AB diagnos*))) OR (TI homecare OR AB homecare) OR (TI "house call*" OR AB "house call*") OR (MH "Diagnostic Tests, Routine") OR (TI "housebound patient*" OR AB "housebound patient*") OR (TI "adult day care" OR AB "adult day care") OR (TI "family service*" OR AB "family service*") OR (TI "family involve*" OR AB "family involve*") OR (TI "family based" OR AB "family based") OR (TI family-based OR AB family-based) OR (TI "family health*" OR AB "family health*") OR (TI out-of-facilit* OR AB out-of-facilit*) OR (MH "Community Health Centers+") OR (((TI health OR AB health) OR (TI "health service" OR AB "health service") OR (TI sanitary OR AB sanitary)) W3 ((TI center* OR AB center*) OR (TI centre* OR AB centre*) OR (TI clinic* OR AB clinic*) OR (TI institute* OR AB institute*) OR (TI unit* OR AB unit*) OR (TI resort* OR AB resort*) OR (TI facility OR AB facility) OR (TI facilities OR AB facilities))) OR (((TI walkway OR AB walkway) OR (TI weekend OR AB weekend) OR (TI church OR AB church)) N3 (TI clinic OR AB clinic)) OR (MH "Pharmacy, Retail") OR (TI apothecary OR AB apothecary) OR (TI "chemist shop*" OR AB "chemist shop*") OR (TI "chemist`s shop*" OR AB "chemist`s shop*") OR (TI pharmacies OR AB pharmacies) OR (TI pharmacy OR AB pharmacy) OR (TI "pharmaceutical service*" OR AB "pharmaceutical service*") OR (MH "Rural Health Services") OR (MH "Rural Health Centers") OR (MH "Rural Health Personnel") OR (MH "School Health Services+") OR (((TI rural OR AB rural) OR (TI village* OR AB village*) OR (TI "informal settlement*" OR AB "informal settlement*") OR (TI school* OR AB school*) OR (TI workplace OR AB workplace) OR (TI worksite OR AB worksite) OR (TI work-place OR AB work-place) OR (TI work-site OR AB work-site)) N3 ((TI health* OR AB health*) OR (TI care OR AB care) OR (TI medicine OR AB medicine) OR (TI nurse OR AB nurse) OR (TI nurses OR AB nurses) OR (TI nursing OR AB nursing) OR (TI intervention* OR AB intervention*) OR (TI program* OR AB program*) OR (TI approach* OR AB approach*) OR (TI service* OR AB service*) OR (TI strateg* OR AB strateg*))) OR (MH "self care+") OR ((TI self OR AB self) N3 ((TI care* OR AB care*) OR (TI help OR AB help) OR (TI

management OR AB management) OR (TI monitoring OR AB monitoring) OR (TI treatment* OR AB treatment*) OR (TI medication OR AB medication) OR (TI nurturance OR AB nurturance) OR (TI testing OR AB testing) OR (TI measur* OR AB measur*) OR (TI observation OR AB observation))) OR (TI selfcare OR AB selfcare) OR (TI selfhelp OR AB selfhelp) OR (TI selfmanagement OR AB selfmanagement) OR (TI selfmonitor* OR AB selfmonitor*) OR (TI selftreatment OR AB selftreatment) OR (TI selfmedication* OR AB selfmedication*) OR (TI selftesting OR AB selftesting) OR (TI selfobservation OR AB selfobservation) OR (MH "social networks") OR (MH "Social Work+") OR (((TI social OR AB social) OR (TI spiritual OR AB spiritual)) W3 ((TI care OR AB care) OR (TI support OR AB support) OR (TI network* OR AB network*) OR (TI service* OR AB service*) OR (TI work OR AB work))) OR (MH "Support Groups+") OR (TI "support group*" OR AB "support group*") OR (TI "support program*" OR AB "support program*") OR (TI paramedic* OR AB paramedic*) OR (TI para-medic* OR AB para-medic*) OR (TI non-health OR AB non-health) OR (TI non-healthcare OR AB non-healthcare) OR (TI non-health-care OR AB non-health-care) OR (TI non-medical OR AB non-medical) OR ((health OR healthcare OR (TI "health care" OR AB "health care") OR (TI medical OR AB medical))) W3 ("support worker*" OR assistant* OR aid*)) OR (((TI community OR AB community) OR (TI district OR AB district) OR (TI "public health" OR AB "public health") OR (TI assistan* OR AB assistan*) OR (TI aid* OR AB aid*) OR (TI staff* OR AB staff*) OR (TI manpower OR AB manpower) OR (TI personnel OR AB personnel)) N4 ((TI nurse OR AB nurse) OR (TI nurses OR AB nurses) OR (TI nursing OR AB nursing) OR (TI matron* OR AB matron*))) OR (TI "midlevel health professional*" OR AB "midlevel health professional*") OR (TI "midlevel health provider*" OR AB "midlevel health provider*") OR (MH "Allied Health Personnel") OR (MH "Pharmacy Technicians") OR (MH "Community Health Workers+") OR (MH "Home Health Aides") OR (MH "nurses+") OR (MH "Nursing Assistants") OR (MH "pharmacists") OR (MH "Allied Health Professions") OR (((TI community OR AB community) OR (TI "community based" OR AB "community based") OR (TI village* OR AB village*) OR (TI rural OR AB rural) OR (TI "informal settlement*" OR AB "informal settlement*") OR (TI lay OR AB lay) OR (TI "allied health" OR AB "allied health") OR (TI "auxiliary health" OR AB "auxiliary health") OR (TI "health auxiliary" OR AB "health auxiliary")) W3 ((TI leader* OR AB leader*) OR (TI worker* OR AB worker*) OR (TI provider* OR AB provider*) OR (TI personnel OR AB personnel) OR (TI profession* OR AB profession*) OR (TI staff OR AB staff) OR (TI aide* OR AB aide*) OR (TI supporter* OR AB supporter*) OR (TI volunteer* OR AB volunteer*) OR (TI advocate* OR AB advocate*) OR (TI team* OR AB team*) OR (TI group* OR AB group*) OR (TI pharmacist* OR AB pharmacist*))) OR (TI "barefoot doctor*" OR AB "barefoot doctor*") OR (TI "local supervisor*" OR AB "local supervisor*") OR (MH "Caregivers") OR (TI caregiver* OR AB caregiver*) OR (TI "care giver*" OR AB "care giver*") OR (MH "Peer Counseling") OR (MH "peer group") OR ((TI peer OR AB peer) W3 ((TI group* OR AB group*) OR (TI support OR AB support) OR (TI relation* OR AB relation*) OR (TI education OR AB education) OR (TI tutoring OR AB tutoring) OR (TI counselling OR AB counselling) OR (TI counseling OR AB counseling) OR (TI consult* OR AB consult*) OR (TI coach* OR AB coach*))) OR (MH "Community-Based Participatory Research+") OR (MH "Patient Participation+") OR (TI "participatory research" OR AB "participatory research") OR (TI "patient involvement" OR AB "patient involvement") OR (TI "patient participation" OR AB "patient participation") OR (TI "patient empowerment" OR AB "patient empowerment") OR (TI "patient activation" OR AB "patient activation") OR (TI "patient engagement" OR AB "patient engagement") OR (((MH "community networks") OR (MH "churches") OR (TI church* OR AB church*) OR (MH "work environment+") OR (MH "Home Environment") OR (MH "schools+") OR (MH "teachers")) AND ((MH "patient care+") OR (MH "drug therapy+"))))

Concept 3: Africa

((MH "africa south of the sahara+") OR (TI "africa south of the sahara" OR AB "africa south of the sahara") OR (TI "sub-sahara* africa*" OR AB "sub-sahara* africa*") OR (TI "subsahara* africa*" OR AB "subsahara* africa*") OR (TI "black africa*" OR AB "black africa*") OR (MH "Africa, Western+") OR (TI "west* africa*" OR AB "west* africa*") OR (TI "east* africa*" OR AB "east* africa*") OR (TI "south* africa*" OR AB "south* africa*") OR (TI "central africa*" OR AB "central africa*") OR (TI "equatorial africa*" OR AB "equatorial africa*") OR (TI "middle africa*" OR AB "middle africa*") OR (TI angola* OR AB angola*) OR (TI benin* OR AB benin*) OR (TI dahomey OR AB dahomey) OR (TI botswana* OR AB botswana*) OR (TI bechuanaland OR AB bechuanaland) OR (TI "burkina faso" OR AB "burkina faso") OR (TI burkin* OR AB burkin*) OR (TI "upper volta" OR AB "upper volta") OR (TI burundi* OR AB burundi*) OR (TI urundi OR AB urundi) OR (TI cameroon* OR AB cameroon*) OR (TI camerun OR AB camerun) OR (TI kamerun OR AB kamerun) OR (TI cameroun OR AB cameroun) OR (TI "cape verde*" OR AB "cape verde*") OR (TI "cabo verde" OR AB "cabo verde") OR (TI centrafri* OR AB centrafri*) OR (TI ubangi-shari OR AB ubangi-shari) OR (TI oubangi-shari OR AB oubangi-shari) OR (TI chad* OR AB chad*) OR (TI tchad OR AB tchad) OR (TI comoro* OR AB comoro*) OR (TI comores OR AB comores) OR (TI comoran OR AB comoran) OR (TI comorian OR AB comorian) OR (TI congo* OR AB congo*) OR (TI kongo OR AB kongo) OR (TI zaire OR AB zaire) OR (TI "cote d`ivoire" OR AB "cote d`ivoire") OR (TI "ivory coast" OR AB "ivory coast") OR (TI ivorian* OR AB ivorian*) OR (TI djibouti* OR AB djibouti*) OR (TI "afars and issas" OR AB "afars and issas") OR (TI eritrea* OR AB eritrea*) OR (TI eswatini OR AB eswatini) OR (TI swazi* OR AB swazi*) OR (TI ethiopia* OR AB ethiopia*) OR (TI abyssinia OR AB abyssinia) OR (TI gabon* OR AB gabon*) OR (TI gabun OR AB gabun) OR (TI gambia* OR AB gambia*) OR (TI senegambia OR AB senegambia) OR (TI ghana* OR AB ghana*) OR (TI "gold coast" OR AB "gold coast") OR (TI guinea* OR AB guinea*) OR (TI guinee OR AB guinee) OR (TI guine OR AB guine) OR (TI bissau-guinean OR AB bissau-guinean) OR (TI equatoguinean OR AB equatoguinean) OR (TI kenya* OR AB kenya*) OR (TI lesotho* OR AB lesotho*) OR (TI basutoland OR AB basutoland) OR (TI liberia* OR AB liberia*) OR (TI madagasca* OR AB madagasca*) OR (TI malagasy* OR AB malagasy*) OR (TI malawi* OR AB malawi*) OR (TI nyasaland OR AB nyasaland) OR (TI mali OR AB mali) OR (TI malian* OR AB malian*) OR (TI mauritania* OR AB mauritania*) OR (TI mauritanie OR AB mauritanie) OR (TI mauritius OR AB mauritius) OR (TI "republique de maurice" OR AB "republique de maurice") OR (TI mayotte OR AB mayotte) OR (TI mahoran* OR AB mahoran*) OR (TI mozambi* OR AB mozambi*) OR (TI mocambique OR AB mocambique) OR (TI namibia* OR AB namibia*) OR (TI niger* OR AB niger*) OR (TI reunion OR AB reunion) OR (TI reunionese OR AB reunionese) OR (TI rwanda* OR AB rwanda*) OR (TI ruanda* OR AB ruanda*) OR (TI senegal* OR AB senegal*) OR (TI seychell* OR AB seychell*) OR (TI "sierra leon*" OR AB "sierra leon*") OR (TI somali* OR AB somali*) OR (TI sudan* OR AB sudan*) OR (TI tanzania* OR AB tanzania*) OR (TI tansania* OR AB tansania*) OR (TI tanganyika OR AB tanganyika) OR (TI zanzibar OR AB zanzibar) OR (TI togo* OR AB togo*) OR (TI uganda* OR AB uganda*) OR (TI zambia* OR AB zambia*) OR (TI sambia* OR AB sambia*) OR (TI zimbabwe* OR AB zimbabwe*) OR (TI rhodesia OR AB rhodesia) OR (TI luanda OR AB luanda) OR (TI lubango OR AB lubango) OR (TI cabinda OR AB cabinda) OR (TI porto-novo OR AB porto-novo) OR (TI cotonou OR AB cotonou) OR (TI abomey-calavi OR AB abomey-calavi) OR (TI gaborone OR AB gaborone) OR (TI ouagadougou OR AB ouagadougou) OR (TI bobo-dioulasso OR AB bobo-dioulasso) OR (TI bujumbura OR AB bujumbura) OR (TI yaounde OR AB yaounde) OR (TI douala OR AB douala) OR (TI praia OR AB praia) OR (TI bangui OR AB bangui) OR (TI n`djamena OR AB n`djamena) OR (TI ndjamena OR AB ndjamena) OR (TI moroni OR AB moroni) OR (TI brazzaville OR AB brazzaville) OR (TI pointe-noire OR AB pointe-noire) OR (TI kinshasa OR AB

kinshasa) OR (TI mbuji-mayi OR AB mbuji-mayi) OR (TI lubumbashi OR AB lubumbashi) OR (TI kananga OR AB kananga) OR (TI kisangani OR AB kisangani) OR (TI bukavu OR AB bukavu) OR (TI yamoussoukro OR AB yamoussoukro) OR (TI abidjan OR AB abidjan) OR (TI malabo OR AB malabo) OR (TI asmara OR AB asmara) OR (TI mbabane OR AB mbabane) OR (TI "addis ababa" OR AB "addis ababa") OR (TI libreville OR AB libreville) OR (TI banjul OR AB banjul) OR (TI accra OR AB accra) OR (TI kumasi OR AB kumasi) OR (TI "sekondi takoradi" OR AB "sekondi takoradi") OR (TI conakry OR AB conakry) OR (TI bissau OR AB bissau) OR (TI nairobi OR AB nairobi) OR (TI mombasa OR AB mombasa) OR (TI mombassa OR AB mombassa) OR (TI maseru OR AB maseru) OR (TI monrovia OR AB monrovia) OR (TI antananarivo OR AB antananarivo) OR (TI lilongwe OR AB lilongwe) OR (TI blantyre-limbe OR AB blantyre-limbe) OR (TI bamako OR AB bamako) OR (TI nouakchott OR AB nouakchott) OR (TI "port louis" OR AB "port louis") OR (TI maputo OR AB maputo) OR (TI matola OR AB matola) OR (TI nampula OR AB nampula) OR (TI windhoek OR AB windhoek) OR (TI niamey OR AB niamey) OR (TI abuja OR AB abuja) OR (TI lagos OR AB lagos) OR (TI kano OR AB kano) OR (TI ibadan OR AB ibadan) OR (TI "port harcourt" OR AB "port harcourt") OR (TI kigali OR AB kigali) OR (TI "sao tome*" OR AB "sao tome*") OR (TI dakar OR AB dakar) OR (TI freetown OR AB freetown) OR (TI mogadishu OR AB mogadishu) OR (TI hargeisa OR AB hargeisa) OR (TI hargeysa OR AB hargeysa) OR (TI pretoria OR AB pretoria) OR (TI "cape town" OR AB "cape town") OR (TI johannesburg OR AB johannesburg) OR (TI Soweto OR AB Soweto) OR (TI durban OR AB durban) OR (TI "port elizabeth" OR AB "port elizabeth") OR (TI "west rand" OR AB "west rand") OR (TI juba OR AB juba) OR (TI khartoum OR AB khartoum) OR (TI nyala OR AB nyala) OR (TI "dar es salaam" OR AB "dar es salaam") OR (TI dodoma OR AB dodoma) OR (TI mwanza OR AB mwanza) OR (TI lome OR AB lome) OR (TI kampala OR AB kampala) OR (TI lusaka OR AB lusaka) OR (TI harare OR AB harare))

Search String Medline OVID

Concept 1: Mental health and substance use problems

(Behavior, Addictive/ OR exp Substance-Related Disorders/ OR Drug Misuse/ OR Prescription Drug Misuse/ OR Prescription Drug Overuse/ OR exp Opiate Substitution Treatment/ OR exp Drug-Seeking Behavior/ OR exp Smoking Cessation/ OR exp "Marijuana Use"/ OR exp Drinking Behavior/ OR exp Craving/ OR exp anxiety disorder/ OR exp Stress Disorders, Traumatic/ OR exp Catastrophization/ OR exp Psychological Distress/ OR Compulsive Behavior/ OR exp Obsessive Behavior/ OR exp Panic/ OR exp Depression/ OR exp Depressive Disorder/ OR Psychomotor Agitation/ OR exp Suicide/ OR ((drug ADJ3 (dehabituation OR rehabilitation)).ab,ti.) OR addict.ab,ti. OR addicts.ab,ti. OR toxicomani*.ab,ti. OR narcotism.ab,ti. OR narcotic depression.ab,ti. OR opi* crisis.ab,ti. OR opi* epidemic.ab,ti. OR physical dependence.ab,ti. OR chemical dependence.ab,ti. OR ((addictive ADJ3 (behavior* OR behaviour* OR dependenc* OR disorder*)).ab,ti.) OR (((polydrug OR multidrug OR multiple OR medication OR non-medical OR nonmedical OR medicine* OR prescription OR nonprescription OR prescribed) ADJ3 (addiction OR abus* OR misus* OR mis-use OR overu* OR craving OR seeking)).ab,ti.) OR NMUPD.ab,ti. OR (non-medical 'use' or nonmedical 'use').ab,ti. OR (((substance* OR drug* OR psychoactive* OR psychostimulant* OR benzodiazepine* OR morphin* OR morfin* OR opiate* OR narcotic* OR opioid* OR opium OR heroin* OR diamorphine OR diaphorin OR marihuana OR marijuana OR hashish OR cannabi* OR weed OR amphetamine* OR methamphetamine* OR methylamphetamine* OR extasy OR MDMA OR meth OR hallucinogen* OR cocaine* OR crack OR alcohol* OR ethanol OR drinking OR phencyclidine OR PCP OR angel dust OR londrea OR killerweed OR peace pill OR khat* OR qat* OR glue OR inhalant*) ADJ3 (disorder*

OR dependen* OR addict* OR abus* OR misus* OR mis-us* OR 'use' OR overu* OR habit* OR intoxicat* OR problem* OR craving OR seeking OR sniffing OR overdose* OR psychos* OR abstain* OR abstinence* OR withdraw* OR cessation)).ab,ti.) OR (((marijuana OR marihuana OR hashish OR cannabis OR heroin OR opium) ADJ3 (smoking OR inhalation)).ab,ti.) OR (((harmful OR hazardous OR heavy OR problem* OR chronic OR binge) ADJ3 (drink* OR alcohol*)).ab,ti.) OR (((alcohol OR ethanol) ADJ3 (intake OR 'use' OR consumption)).ab,ti.) OR (((alcohol OR ethanol OR controlled OR behaviour* OR behavior* OR habit* OR pattern* OR social) ADJ3 drinking).ab,ti.) OR alcoholism.ab,ti. OR alcoholic*.ab,ti. OR dipsomania*.ab,ti. OR drunkenness*.ab,ti. OR (((abstinence OR withdrawal OR cessation OR discontinuation) ADJ3 (symptom* OR syndrome* OR seizure* OR hallucin* OR delirium)).ab,ti.) OR rum fits.ab,ti. OR delerium tremens.ab,ti. OR delirium tremens.ab,ti. OR ((alcohol* ADJ3 deliri*).ab,ti.) OR anxiet*.ab,ti. OR anxious.ab,ti. OR stress disorder*.ab,ti. OR distress syndrome*.ab,ti. OR dystress syndrome*.ab,ti. OR psych* distress.ab,ti. OR cothymia.ab,ti. OR obsession.ab,ti. OR (((obsessive OR compulsi*) ADJ3 (disorder* OR disease* OR neurosis OR neuroses OR syndrome* OR reaction* OR behavior OR behaviour OR repetition*)).ab,ti.) OR OCD.ab,ti. OR repetitive behavior.ab,ti. OR repetitive behaviour.ab,ti. OR preoccupation neurosis.ab,ti. OR preoccupation neuroses.ab,ti. OR panic.ab,ti. OR phobia*.ab,ti. OR phobic*.ab,ti. OR agoraphobi*.ab,ti. OR acrophobi*.ab,ti. OR fear of height*.ab,ti. OR zoophobi*.ab,ti. OR fear of animal*.ab,ti. OR fear of snake*.ab,ti. OR ophidiophobi*.ab,ti. OR ophiophobi*.ab,ti. OR fear of spider*.ab,ti. OR arachnophobi*.ab,ti. OR fear of blood.ab,ti. OR haemophobi*.ab,ti. OR hemophobi*.ab,ti. OR ((fear ADJ3 (needle* OR injection*)).ab,ti.) OR trypanophobi*.ab,ti. OR claustrophobi*.ab,ti. OR sociophobi*.ab,ti. OR (((posttrauma* OR post trauma* OR combat OR war OR trauma* OR stressor) ADJ3 (stress OR neurosis OR neuroses OR syndrome* OR disorder* OR psychos*)).ab,ti.) OR ptsd.ab,ti. OR shell shock.ab,ti. OR combat fatigue.ab,ti. OR battle fatigue.ab,ti. OR traumatized.ab,ti. OR traumatised.ab,ti. OR ((sexual ADJ3 trauma*).ab,ti.) OR depress*.ab,ti. OR melanchol*.ab,ti. OR dysphori*.ab,ti. OR dysfori*.ab,ti. OR dysthymi*.ab,ti. OR ((mental ADJ3 fatigue).ab,ti.) OR mourning syndrome*.ab,ti. OR ((psychos* ADJ3 involutional).ab,ti.) OR negative thinking.ab,ti. OR negative thought*.ab,ti. OR thinking too much.ab,ti. OR too many thoughts.ab,ti. OR suicid*.ab,ti. OR self killing.ab,ti. OR self poisoning.ab,ti. OR self immolat*.ab,ti. OR self incineration.ab,ti. OR selfkilling.ab,ti. OR selfpoisoning.ab,ti. OR selfimmolat*.ab,ti. OR selfincineration.ab,ti. OR parasuicid*.ab,ti. OR fatal attempt*.ab,ti.)

Concept 2: Community-based Care

(exp Community Mental Health Services/ OR exp Community Mental Health Centers/ OR exp School Mental Health Services/ OR Community Psychiatry/ OR ((community OR home OR domicil* OR domestic) ADJ3 'mental health*').ab,ti. OR Deinstitutionalization/ OR exp Telecommunications/ OR exp Telenursing/ OR Telehealth.ab,ti. OR Tele-health.ab,ti. OR Telemedic*.ab,ti. OR Tele-medic*.ab,ti. OR telemetry.ab,ti. OR tele-metry.ab,ti. OR biotelemetry.ab,ti. OR radiotelemetry.ab,ti. OR teleradiometry.ab,ti. OR telesensing.ab,ti. OR tele-sensing.ab,ti. OR telemonitor*.ab,ti. OR tele-monitor*.ab,ti. OR telenurs*.ab,ti. OR tele-nurs*.ab,ti. OR teleconsultat*.ab,ti. OR tele-consultat*.ab,ti. OR telerehabilitation*.ab,ti. OR tele-rehabilitation*.ab,ti. OR ((virtual OR remote OR distance OR distant OR e) ADJ3 (medic* OR monitor* OR nurs* OR consultat* OR rehabilitation* OR support* OR counsel* OR treatment* OR therap* OR sensing)).ab,ti. OR (emedic* OR emonitor* OR enurs* OR econsultat* OR erehabilitation OR esupport* OR ecounsel* OR etreatment* OR etherap* OR esensing).ab,ti. OR Mhealth.ab,ti. OR M-health.ab,ti. OR mobile health.ab,ti. OR Ehealth.ab,ti. OR E-health.ab,ti. OR exp Distance Counseling/ OR exp Hotlines/ OR internet/ OR exp Internet-Based Intervention/ OR exp Social Media/ OR exp Mobile Applications/ OR e mail*.ab,ti. OR email*.ab,ti. OR (electronic ADJ1 (mail* OR messaging)).ab,ti. OR hotline*.ab,ti. OR hot

line*.ab,ti. OR interactive voice response system*.ab,ti. OR ((web OR online OR on-line OR internet OR video OR virtual OR tele) ADJ3 (intervention* OR conferenc* OR communication OR seminar* OR information*)).ab,ti. OR internet.ab,ti. OR world wide web.ab,ti. OR www.ab,ti. OR ((mobile OR cell OR smart OR portable) ADJ1 (phone* OR telephone*)).ab,ti. OR social media*.ab,ti. OR facebook.ab,ti. OR twitter.ab,ti. OR telecommunication*.ab,ti. OR broadcasting.ab,ti. OR text messag*.ab,ti. OR texting.ab,ti. OR teleconferenc*.ab,ti. OR videoconferenc*.ab,ti. OR webconferenc*.ab,ti. OR webcast*.ab,ti. OR podcast*.ab,ti. OR webinar*.ab,ti. OR SMS.ab,ti. OR telephone*.ab,ti. OR smartphone*.ab,ti. OR ((mobile OR portable OR tablet) ADJ3 (app OR apps OR application*)).ab,ti. OR website.ab,ti. OR web site*.ab,ti. OR homepage.ab,ti. OR mobile technology.ab,ti. OR exp ambulatory Care/ OR decentrali*.ab,ti. OR ((ambulatory OR dispensary OR extramural OR extra-mural OR outpatient OR outpatient health* OR out-patient OR out-patient health* OR out-of-office) ADJ3 (care OR healthcare OR health care OR service* OR setting* OR monitoring* OR treatment* OR therap*)).ab,ti. OR Community Health Services/ OR exp Community Health Nursing/ OR exp Psychiatric Nursing/ OR exp Community Pharmacy Services/ OR exp Community Integration/ OR exp Uncompensated Care/ OR exp community medicine/ OR rehabilitation/ OR ((community OR community health* OR district OR public health OR nurse-led OR pharmacist-led OR nurse-based OR pharmacist-based OR social service OR collaborative) ADJ3 (care OR healthcare OR health care OR nurs* OR integration OR service* OR program* OR medicine OR support* OR engagement OR intervention* OR deliver* OR outreach OR distribution OR network* OR participatory method* OR rehabilitation* OR resource* OR strateg* OR project* OR approach* OR testing OR screening*)).ab,ti. OR task shar*.ab,ti. OR task shift*.ab,ti. OR exp Home Care Services/ OR exp House Calls/ OR exp Adult Day Care Centers/ OR ((adult OR home OR domicil* OR domestic OR visiting OR residential OR residence) ADJ3 (care OR healthcare OR health care OR nurs* OR help OR assistance OR service* OR treatment* OR therap* OR medication review* OR pharmac* OR monitor* OR visit* OR agenc* OR testing OR screening* OR diagnos*)).ab,ti. OR homecare.ab,ti. OR house call*.ab,ti. OR exp Diagnostic Tests, Routine/ OR housebound patient*.ab,ti. OR adult day care.ab,ti.OR 'group medical visit'.ab,ti. OR family service*.ab,ti. OR family involve*.ab,ti. OR family based.ab,ti. OR family-based.ab,ti. OR family health*.ab,ti. OR exp Ambulatory Care/ OR out-of-facilit*.ab,ti. OR exp Community Health Centers/ OR ((health OR health service OR sanitary) ADJ3 (center* OR centre* OR clinic* OR institute* OR unit* OR resort* OR facility OR facilities)).ab,ti. OR ((walkway OR weekend OR church) ADJ3 clinic).ab,ti. OR exp Pharmacies/ OR exp Pharmaceutical Services, Online/ OR apothecary.ab,ti. OR chemist shop*.ab,ti. OR chemist`s shop*.ab,ti. OR pharmacies.ab,ti. OR pharmacy.ab,ti. OR pharmaceutical service*.ab,ti. OR exp Rural Health Services/ OR School Health Services/ OR exp School Nursing/ OR ((rural OR village* OR informal settlement* OR school* OR workplace OR worksite OR work-place OR work-site) ADJ3 (health* OR care OR medicine OR nurse OR nurses OR nursing OR intervention* OR program* OR approach* OR service* OR strateg*)).ab,ti. OR exp self care/ OR (self ADJ3 (care* OR help OR management OR monitoring OR treatment* OR medication OR nurturance OR testing OR measur* OR observation)).ab,ti. OR selfcare.ab,ti. OR selfhelp.ab,ti. OR selfmanagement.ab,ti. OR selfmonitor*.ab,ti. OR selftreatment.ab,ti. OR selfmedication*.ab,ti. OR selftesting.ab,ti. OR selfobservation.ab,ti. OR exp social network/ OR exp social care/ OR ((social OR spiritual) ADJ3 (care OR support OR network* OR service* OR work)).ab,ti. OR exp Social Work/ OR exp Self-Help Groups/ OR support group*.ab,ti. OR support program*.ab,ti. OR paramedic*.ab,ti. OR para-medic*.ab,ti. OR non-health.ab,ti. OR non-healthcare.ab,ti. OR non-health care.ab,ti. OR non-medical.ab,ti. OR ((health OR healthcare OR health care OR medical) ADJ3 (support worker* OR assistant* OR aid*)).ab,ti. OR ((community OR district OR public health OR assistan* OR aid* OR staff* OR manpower OR personnel) ADJ3 (nurse OR nurses OR nursing OR matron*)).ab,ti. OR

midlevel health professional*.ab,ti. OR midlevel health provider*.ab,ti. OR Allied Health Personnel/ OR exp Pharmacy Technicians/ OR exp Community Health Workers/ OR exp Home Health Aides/ OR exp Licensed Practical Nurses/ OR exp Nurses/ OR exp Nursing Assistants/ OR exp nursing staff/ OR exp pharmacists/ OR ((community OR community based OR village* OR rural OR informal settlement* OR lay OR allied health OR auxiliary health OR health auxiliary) ADJ3 (leader* OR worker* OR provider* OR personnel OR profession* OR staff OR aide* OR supporter* OR volunteer* OR advocate* OR team* OR group* OR pharmacist*)).ab,ti. OR barefoot doctor*.ab,ti. OR local supervisor*.ab,ti. OR exp Caregivers/ OR caregiver*.ab,ti. OR care giver*.ab,ti. OR exp peer group/ OR (peer ADJ3 (group* OR support OR relation* OR education OR tutoring OR counselling OR counseling OR consult* OR coach*)).ab,ti. OR exp Community-Based Participatory Research/ OR exp Patient Participation/ OR participatory research.ab,ti. OR patient involvement.ab,ti. OR patient participation.ab,ti. OR patient empowerment.ab,ti. OR patient activation.ab,ti. OR patient engagement.ab,ti. OR ((exp community networks/ OR exp religion/ OR church*.ab,ti. OR exp workplace/ OR exp schools/ OR exp school teachers/) AND (exp patient care/ OR disease management/ OR exp drug therapy/)))

Concept 3: Africa

(exp africa south of the sahara/ OR africa south of the sahara.ab,ti. OR sub-sahara* africa*.ab,ti. OR subsahara* africa*.ab,ti. OR black africa*.ab,ti. OR west* africa*.ab,ti. OR east* africa*.ab,ti. OR south* africa*.ab,ti. OR central africa*.ab,ti. OR equatorial africa*.ab,ti. OR middle africa*.ab,ti. OR angola*.ab,ti. OR benin*.ab,ti. OR dahomey.ab,ti. OR botswana*.ab,ti. OR bechuanaland.ab,ti. OR burkina faso.ab,ti. OR burkin*.ab,ti. OR upper volta.ab,ti. OR burundi*.ab,ti. OR urundi.ab,ti. OR cameroon*.ab,ti. OR camerun.ab,ti. OR kamerun.ab,ti. OR cameroun.ab,ti. OR cape verde*.ab,ti. OR cabo verde.ab,ti. OR centrafri*.ab,ti. OR ubangi-shari.ab,ti. OR oubangi-shari.ab,ti. OR chad*.ab,ti. OR tchad.ab,ti. OR comoro*.ab,ti. OR comores.ab,ti. OR comoran.ab,ti. OR comorian.ab,ti. OR congo*.ab,ti. OR kongo.ab,ti. OR zaire.ab,ti. OR cote d`ivoire.ab,ti. OR ivory coast.ab,ti. OR ivorian*.ab,ti. OR djibouti*.ab,ti. OR (afars and issas).ab,ti. OR eritrea*.ab,ti. OR eswatini.ab,ti. OR swazi*.ab,ti. OR ethiopia*.ab,ti. OR abyssinia.ab,ti. OR gabon*.ab,ti. OR gabun.ab,ti. OR gambia*.ab,ti. OR senegambia.ab,ti. OR ghana*.ab,ti. OR gold coast.ab,ti. OR guinea*.ab,ti. OR guinee.ab,ti. OR guine.ab,ti. OR bissau-guinean.ab,ti. OR equatoguinean.ab,ti. OR kenya*.ab,ti. OR lesotho*.ab,ti. OR basutoland.ab,ti. OR liberia*.ab,ti. OR madagasca*.ab,ti. OR malagasy*.ab,ti. OR malawi*.ab,ti. OR nyasaland.ab,ti. OR mali.ab,ti. OR malian*.ab,ti. OR mauritania*.ab,ti. OR mauritanie.ab,ti. OR mauritius.ab,ti. OR republique de maurice.ab,ti. OR mayotte.ab,ti. OR mahoran*.ab,ti. OR mozambi*.ab,ti. OR mocambique.ab,ti. OR namibia*.ab,ti. OR niger*.ab,ti. OR reunion.ab,ti. OR reunionese.ab,ti. OR rwanda*.ab,ti. OR ruanda*.ab,ti. OR senegal*.ab,ti. OR seychell*.ab,ti. OR sierra leon*.ab,ti. OR somali*.ab,ti. OR sudan*.ab,ti. OR tanzania*.ab,ti. OR tansania*.ab,ti. OR tanganyika.ab,ti. OR zanzibar.ab,ti. OR togo*.ab,ti. OR uganda*.ab,ti. OR zambia*.ab,ti. OR sambia*.ab,ti. OR zimbabwe*.ab,ti. OR rhodesia.ab,ti. OR luanda.ab,ti. OR lubango.ab,ti. OR cabinda.ab,ti. OR porto-novo.ab,ti. OR cotonou.ab,ti. OR abomey-calavi.ab,ti. OR gaborone.ab,ti. OR ouagadougou.ab,ti. OR bobo-dioulasso.ab,ti. OR bujumbura.ab,ti. OR yaounde.ab,ti. OR douala.ab,ti. OR praia.ab,ti. OR bangui.ab,ti. OR n`djamena.ab,ti. OR ndjamena.ab,ti. OR moroni.ab,ti. OR brazzaville.ab,ti. OR pointe-noire.ab,ti. OR kinshasa.ab,ti. OR mbuji-mayi.ab,ti. OR lubumbashi.ab,ti. OR kananga.ab,ti. OR kisangani.ab,ti. OR bukavu.ab,ti. OR yamoussoukro.ab,ti. OR abidjan.ab,ti. OR malabo.ab,ti. OR asmara.ab,ti. OR mbabane.ab,ti. OR addis ababa.ab,ti. OR libreville.ab,ti. OR banjul.ab,ti. OR accra.ab,ti. OR kumasi.ab,ti. OR sekondi takoradi.ab,ti. OR conakry.ab,ti. OR bissau.ab,ti. OR nairobi.ab,ti. OR mombasa.ab,ti. OR mombassa.ab,ti. OR maseru.ab,ti. OR monrovia.ab,ti. OR antananarivo.ab,ti. OR lilongwe.ab,ti. OR

blantyre-limbe.ab,ti. OR bamako.ab,ti. OR nouakchott.ab,ti. OR port louis.ab,ti. OR maputo.ab,ti. OR matola.ab,ti. OR nampula.ab,ti. OR windhoek.ab,ti. OR niamey.ab,ti. OR abuja.ab,ti. OR lagos.ab,ti. OR kano.ab,ti. OR ibadan.ab,ti. OR port harcourt.ab,ti. OR kigali.ab,ti. OR sao tome*.ab,ti. OR dakar.ab,ti. OR freetown.ab,ti. OR mogadishu.ab,ti. OR hargeisa.ab,ti. OR hargeysa.ab,ti. OR pretoria.ab,ti. OR cape town.ab,ti. OR johannesburg.ab,ti. OR Soweto.ab,ti. OR durban.ab,ti. OR port elizabeth.ab,ti. OR west rand.ab,ti. OR juba.ab,ti. OR khartoum.ab,ti. OR nyala.ab,ti. OR dar es salaam.ab,ti. OR dodoma.ab,ti. OR mwanza.ab,ti. OR lome.ab,ti. OR kampala.ab,ti. OR lusaka.ab,ti. OR harare.ab,ti.) NOT ((exp animals/ OR exp Animal Experimentation/) NOT humans/)

2 Supplementary Tables

Table S1. Preferred Reporting Items for Systematic Reviews and Meta-Analyses Extension for Scoping Reviews (PRISMA-ScR) Checklist

Section	Item	PRISMA-ScR Checklist item	Reported on page #
		Title	
Title	1	Identify the report as a scoping review.	1
		Abstract	
Structured summary	2	Provide a structured summary that includes (as applicable): background, objectives, eligibility criteria, sources of evidence, charting methods, results, and conclusions that relate to the review questions and objectives.	2
		Introduction	
Rationale	3	Describe the rationale for the review in the context of what is already known. Explain why the review questions/objectives lend themselves to a scoping review approach.	3-4
Objectives	4	Provide an explicit statement of the questions and objectives being addressed with reference to their key elements (e.g., population or participants, concepts, and context) or other relevant key elements used to conceptualize the review questions and/or objectives.	3-4
		Methods	
Protocol and registration	5	Indicate whether a review protocol exists; state if and where it can be accessed (e.g., a Web address); and if available, provide registration information, including the registration number.	4
Eligibility criteria	6	Specify characteristics of the sources of evidence used as eligibility criteria (e.g., years considered, language, and publication status), and provide a rationale.	4

Section	Item	PRISMA-ScR Checklist item	Reported on page #
Information sources*	7	Describe all information sources in the search (e.g., databases with dates of coverage and contact with authors to identify additional sources), as well as the date the most recent search was executed.	4
Search	8	Present the full electronic search strategy for at least 1 database, including any limits used, such that it could be repeated.	Supplementary Data
Selection of sources of evidence†	9	State the process for selecting sources of evidence (i.e., screening and eligibility) included in the scoping review.	5
Data charting process‡	10	Describe the methods of charting data from the included sources of evidence (e.g., calibrated forms or forms that have been tested by the team before their use, and whether data charting was done independently or in duplicate) and any processes for obtaining and confirming data from investigators.	5
Data items	11	List and define all variables for which data were sought and any assumptions and simplifications made.	5
Critical appraisal of individual sources of evidence§	12	If done, provide a rationale for conducting a critical appraisal of included sources of evidence; describe the methods used and how this information was used in any data synthesis (if appropriate).	Not done
Synthesis of results	13	Describe the methods of handling and summarizing the data that were charted.	5
		Results	
Selection of sources of evidence	14	Give numbers of sources of evidence screened, assessed for eligibility, and included in the review, with reasons for exclusions at each stage, ideally using a flow diagram.	5, Figure 1
Characteristics of sources of evidence	15	For each source of evidence, present characteristics for which data were charted and provide the citations.	Supplementary Table S2 & S3
Critical appraisal within sources of evidence	16	If done, present data on critical appraisal of included sources of evidence (see item 12).	Not done
Results of individual sources of evidence	17	For each included source of evidence, present the relevant data that were charted that relate to the review questions and objectives.	5-10
Synthesis of results	18	Summarize and/or present the charting results as they relate to the review questions and objectives.	5-10

Section	Item	PRISMA-ScR Checklist item	Reported on page #
		Discussion	
Summary of evidence	19	Summarize the main results (including an overview of concepts, themes, and types of evidence available), link to the review questions and objectives, and consider the relevance to key groups.	10-11
Limitations	20	Discuss the limitations of the scoping review process.	12
Conclusions	21	Provide a general interpretation of the results with respect to the review questions and objectives, as well as potential implications and/or next steps.	12
		Funding	
Funding	22	Describe sources of funding for the included sources of evidence, as well as sources of funding for the scoping review. Describe the role of the funders of the scoping review.	13

Note: From: Tricco AC, Lillie E, Zarin W, O'Brien KK, Colquhoun H, Levac D, et al. PRISMA Extension for Scoping Reviews (PRISMAScR): Checklist and Explanation. Ann Intern Med. 2018;169:467–473. doi: 10.7326/M18-0850.

Table S2. Summary of included studies: Description of interventions

Author and Year	Geographic location (Urban/Rural)	Age group	Specific targets	Location of service delivery	Provider	Broad intervention category	Elements	Short description of intervention
**(Abidemi et al., 2022)**	Nigeria (N/A)	Adults	Affected by HIV/HIV +	Telehealth (Phone calls)	N/A	Psychotherapeutic (SC); Lifestyle/Physical health	Education; Support	On-phone depression counselling for caregivers of HIV/AIDS patients alongside routine antiretroviral treatment: Psychoeducation about depression and its consequences; correction of erroneous beliefs; joint efforts that help the patient overcome recent challenges; and the helpfulness of family support.
**(Abrahams et al., 2010)**	South Africa (Mixed)	Mixed	Gender-based violence	Telehealth (Phone calls)	Specialist MH providers (counsellors/psychotherapists)	Psychotherapeutic (SC); Lifestyle/Physical health	Education; Self-help; Support; Referral	On-phone psychosocial support for rape victims in addition to a leaflet and adherence diary. Telephonic support from a counsellor throughout the 28-day period of taking post-exposure prophylaxis (PEP) consisted of basic counselling including listening and validating the traumatic events and encouragement of medication adherence, support seeking from family and friends, attention of formal counselling services (counsellor would assist in identifying a service and facilitate access), reading the pamphlet, to use the diary and to return to the clinic.
**(Abrahams et al., 2022)**	South Africa (Urban)	Adults	Pregnant women; Parents/care givers	Home	Lay-workers (CHW)	Psychotherapeutic (CBT)	Education; Skills	Health system strengthening (HSS) program for antenatal care facilities: The program consisted of three health facility delivered components: (1) health promotion and awareness; (2) detection of CMD and domestic violence; (3) referral of women with CMD and domestic violence for structured counselling; and one community-based component: delivery of structured counselling by lay-health workers in patients' homes, using a problem-solving approach.
**(Akena et al., 2021)**	Uganda (Rural/semi-rural)	Adults	Unspecified	Telehealth (Phone calls); Other community places	Lay-workers (CHW)	Psychotherapeutic (PE)	Education; Self-help; Support; Referral	Distribution of information and education communication material (IEC) aimed at improving mental health literacy by CHWs. The leaflets contain study members' phone numbers that participants can call if they need help.

Author and Year	Geographic location (Urban/Rural)	Age group	Specific targets	Location of service delivery	Provider	Broad intervention category	Elements	Short description of intervention
**(Akinsulore et al., 2022)**	Nigeria (Urban)	Adults	Other: Health care workers	Telehealth (Application)	Health care workers (physicians, nurses)	Psychotherapeutic (PE)	Education	MHealth-based psychological intervention (mPsyI): A subjectively managed and guided psychoeducation mobile-based treatment app that does not require the support of a therapist to ease the symptoms of psychological distress. The trained facilitators will provide weekly support to the end users to make sure that the intervention is being used appropriately.
**(Amoah et al., 2021)**	Ghana (N/A)	Mixed	Students	School	N/A	Lifestyle/Physical health	Education; Skills	School-based cardiovascular disease (CVD) risk factor reduction intervention consisting of health education and physical activity modules. The researchers provided education on CVDs, its risk factors, causes, development, and prevention. The physical activity module (aerobic and anaerobic exercises) was delivered by a physical education health instructor.
**(Angeles et al., 2019)**	Malawi (Rural/semi-rural)	Mixed	Unspecified	Other community places	N/A	Economic	Education; Material	Malawi Social Cash Transfer Program (SCTP): The SCTP provided unconditional income support to ultra-poor, labor-constrained households. The transfer was variable based on households' size and composition. There was messaging around use of the transfer to encourage beneficiaries to use it to invest in the human capital of children and for household basic needs.
**(Appiah et al., 2020)**	Ghana (Rural/semi-rural)	Adults	Unspecified	Other community places	Specialist MH providers (counsellors/psychotherapists)	Psychotherapeutic (CBT)	Education; Skills	Inspired Life Program (ILP): A multicomponent positive psychology intervention. Group sessions focused on building participants' skills in self-worth and self-acceptance, purposeful and meaningful living, positive relationships, self-efficacy, personal growth, kindness and compassion, coping skills, time management and goal setting skills, cognitive restructuring, and positive thinking.
**(Are et al., 2021)**	Nigeria (Urban)	Adolescents	Students	School	Lay-workers (teachers)	Psychotherapeutic (CBT, SM)	Education; Skills	Teacher-delivered CBT program for depressed adolescents in Nigeria. The sessions included psychoeducation, encouragement of positive self-talk, behavioral activation, relaxation strategies including deep breathing exercises, progressive muscle relaxation and positive imagery.

Author and Year	Geographic location (Urban/Rural)	Age group	Specific targets	Location of service delivery	Provider	Broad intervention category	Elements	Short description of intervention
**(Atilola et al., 2022)**	Nigeria (Mixed)	Mixed	Students	School	Specialist MH providers (counsellors/psychotherapists)	Psychotherapeutic (CBT)	Education; Skills	Break-Free from Depression: A depression awareness curriculum for staff and students that consisted of three main components: The staff training (teachers), parent events and student curriculum.
**(Augsburg et al., 2022)**	Ghana (N/A)	Unknown	Parents/caregivers	School	Lay-workers (peers, teachers)	Lifestyle/Physical health; Social	Education; Skills	Early childhood care and education (ECCE) program that consisted of: Play schemes (PS) for children attending kindergartent using discovery-based teaching methods; parenting courses for the women who run the PS; and training and supervision of kindergarten teachers.
**(Bain, 2014)**	South Africa (Urban)	Adults	Parents/caregivers; Other: Homeless mothers	Shelter	Specialist MH providers (counsellors/psychotherapists)	Social	Skills	New Beginnings Program: Parent-infant psychotherapy group program designed to improve the mother's ability to attune to her infant's needs and to enhance her ability to acknowledge her infant's separateness, to increase her range of contingent responses to her infant, and to increase the mother's capacity for mentalization.
**(Baird et al., 2013)**	Malawi (Mixed)	Mixed	Unspecified	Other: Cash transfer		Economic	Material	Intervention 1: Conditional cash transfer (CCT) on regular school attendance of school-going girls: Monthly school attendance of all the conditional cash transfer recipients was checked and payment for the following month was withheld for any student whose attendance was below 80 percent of the number of days school was in session for the previous month.<br><br>Intervention 2: Unconditional cash transfers (UCT): The offers were identical with one crucial difference: There was no requirement to attend school to receive the monthly cash transfers. Attendance was never checked for recipients in the unconditional arm, and they received their payments by simply presenting at the transfer locations each month.

Author and Year	Geographic location (Urban/Rural)	Age group	Specific targets	Location of service delivery	Provider	Broad intervention category	Elements	Short description of intervention
**(Barnhart et al., 2020)**	Rwanda (N/A)	Adults	Parents/care givers	Home	Lay-workers (CHW)	Economic; Lifestyle/Physical health; Social	Education; Skills; Support	Sugira Muryango: A strengths-based home-visiting intervention to promote child development and prevent violence among children aged 6-36 months, delivered to poor families. It used active coaching to promote responsive parenting, reduce family conflict, and increase caregivers' abilities to access and navigate available resources.
**(Bass et al., 2013)**	DRC (Rural/semi-rural)	Adults	Gender-based violence	Other community places	Specialist MH providers (paraprofessional psychosocial assistants)	Psychotherapeutic (CBT)	Education; Skills; Referral	Group cognitive processing therapy added to existing individual support provided by psychosocial assistants.
**(Baumgartner et al., 2021)**	Ghana (Rural/semi-rural)	Mixed	Pregnant women; Parents/care givers	Home; Other community places	Lay-workers (peers)	Psychotherapeutic (CBT); Social	Education; Skills; Support; Monitoring	Lay counselor-delivered, group-based Integrated Mothers and Babies Course (iMBC) and Early Childhood Development (ECD) in order to prevent postpartum depression. It is based on CBT and attachment theory. Implemented by model mothers (from the community). The key ECD messages contained information on the importance of antenatal care, breastfeeding and nutrition, seeking timely care for sick child. Model mothers checked on mothers' mood and encouraged support.
**(Bella-Awusah et al., 2016)**	Nigeria (Urban)	Adoles cents	Students	School	Specialist MH providers (psychiatrists)	Psychotherapeutic (CBT, SM)	Education; Skills	School-based CBT program: The sessions included psychoeducation, positive self-talk, behavioral activation, relaxation techniques (deep slow breathing exercises and positive imagery.
**(Beres et al., 2021)**	Uganda (Rural/semi-rural)	Adults	Unspecified	Telehealth (Application)		Lifestyle/Physical health	Education; Support; Monitoring	Ecological momentary assessment and intervention (EMAI): A mobile phone app gathered data about participants' health behaviors and delivered timely, personalized behavior change intervention. Participants received health-related messages responsive to the behavioral data submitted.

Author and Year	Geographic location (Urban/Rural)	Age group	Specific targets	Location of service delivery	Provider	Broad intervention category	Elements	Short description of intervention
**(Berger et al., 2018)**	Tanzania (N/A)	Adoles cents	Students	School	Lay-workers (teachers)	Psychotherapeutic (CBT, SM); Social	Education; Skills	ERSAE-Stress-Prosocial intervention (ESPS): A universal school-based resiliency program. It was divided into two sets of strategies: stress-reduction interventions and prosocial interventions (i.e., perspective-taking, empathy training, mindfulness and compassion-cultivating practices).
**(Berry et al., 2021)**	South Africa (Urban)	Mixed	Parents/care givers; Students	School	Lay-workers (Other)	Social	Education; Skills	School-based parenting intervention for school-going adolescents' parents to equip them to cope with the duality of parenthood and adolescence. It focused on developing positive parenting skills and supporting adolescent development for responsible adulthood.
**(Betancourt et al., 2014b)**	Rwanda (Rural/semi-rural)	Adoles cents	Affected by HIV/HIV +	Home	Specialist MH providers (counsellors/psychot herapists)	Lifestyle/Physical health; Social	Education; Skills; Support	Family-Strengthening Intervention for HIV-affected families (FSI-HIV): The intervention centered on the development of a family narrative to draw upon shared experiences, through four core intervention components: resilience, improved family communication on HIV transmission and status disclosure, and engagement of formal and informal supports.
**(Betancourt et al., 2014a)**	Sierra Leone (Urban)	Mixed	Other: youth Affected by war	Other community places	Specialist MH providers (social workers, other)	Psychotherapeutic (CBT, IPT, SM)	Education; Skills; Support	Youth Readiness Intervention (YRI): Group intervention for war-affected youth. Core components included: psychoeducation about trauma and its impact on interpersonal relationships; self-regulation and relaxation skills; cognitive restructuring; behavioral activation; communication and interpersonal skills; and sequential problem solving. Depending on group needs, it also includes community and family meetings where appropriate, to enhance engagement and social support.
**(Betancourt et al., 2017)**	Rwanda (Rural/semi-rural)	Adoles cents	Affected by HIV/HIV +	Home	Specialist MH providers (counsellors/psychot herapists)	Lifestyle/Physical health; Social	Education; Skills; Support; Referral	Family-Strengthening Intervention for HIV-affected families (FSI-HIV): The intervention centered on the development of a family narrative to draw upon shared experiences, through four core intervention components: resilience, improved family communication on HIV transmission and status disclosure, and engagement of formal and informal supports.

Author and Year	Geographic location (Urban/Rural)	Age group	Specific targets	Location of service delivery	Provider	Broad intervention category	Elements	Short description of intervention
**(Betancourt et al., 2020)**	Rwanda (Rural/semi-rural)	Adults	Parents/care givers	Home	Lay-workers (CHW)	Economic; Lifestyle/Physical health; Social	Education; Skills; Support; Referral	Family-Strengthening Intervention for HIV-affected families (FSI-HIV): The intervention centered on the development of a family narrative to draw upon shared experiences, through four core intervention components: resilience, improved family communication on HIV transmission and status disclosure, and engagement of formal and informal supports.
**(Bliznashka et al., 2021)**	Tanzania (Rural/semi-rural)	Mixed	Pregnant women; Parents/care givers	Home	Lay-workers (CHW)	Economic; Social	Education; Skills; Support; Material; Referral	CHW delivered health, nutrition and responsive stimulation intervention in combination with a conditional cash transfer (CCT) tied to antenatal care and routine child growth monitoring clinic attendance. CHWs conducted individualized, home visits to the women during which they demonstrated age-appropriate play and communication activities for the child, observed the women practicing the newly learned activities, and provided support applying the new activities. In addition, CHWs advised women on optimal health and nutrition practices for themselves and their young child, counselled them on parenting and problem-solving strategies, and made health facility referrals if necessary.
**(Boivin et al., 2013)**	Uganda (Rural/semi-rural)	Adults	Parents/care givers; Affected by HIV/HIV +	Home Study office on hospital ground	N/A	Social	Education; Skills	Meditational intervention for sensitizing caregivers (MISC): Through video feedback the MISC trainer taught the caregiver on how to enhance their children's cognitive development.
**(Boivin et al., 2017)**	Uganda (Rural/semi-rural)	Adults	Parents/care givers; Affected by HIV/HIV +	Home Study office on hospital ground	Specialist MH providers (counsellors/psychotherapists, social workers)	Social	Education; Skills	Meditational intervention for sensitizing caregivers (MISC): Through video feedback the MISC trainer taught the caregiver on how to enhance their children's cognitive development.
**(Bolton et al., 2003)**	Uganda (Rural/semi-rural)	Adults	Unspecified	Other community places	Lay-workers (Other)	Psychotherapeutic (IPT)	Education; Skills; Monitoring	Group interpersonal psychotherapy Uganda (IPT-G-U): A group interpersonal psychotherapy intervention.

Author and Year	Geographic location (Urban/Rural)	Age group	Specific targets	Location of service delivery	Provider	Broad intervention category	Elements	Short description of intervention
**(Brown et al., 2009)**	Rwanda (Rural/semi-rural)	Mixed	Orphans	Home	Lay-workers (peers)	Psychotherapeutic (CBT); Economic; Social	Skills; Support; Monitoring	Adult mentorship and support for youth-headed households: Mentors monitored youth wellbeing, provided them with love, attention, encouragement and guidance. They transferred life skills, helped to ensure their health and safety and performed advocacy on behalf of vulnerable youth, speaking in public forums and encouraging other community members to support them. The participants also received the basic needs program (provision of a range of goods and services).
**(Bryant et al., 2017)**	Kenya (Urban)	Adults	Gender-based violence	Home	Lay-workers (CHW)	Psychotherapeutic (CBT, MI, SM)	Education; Skills; Other: strengthening social Support	WHO Problem Management Plus (PM+) intervention consisting of four core intervention strategies taught by trained lay helpers. These strategies included managing stress through a slow breathing technique, a seven-step basic guide to managing practical problems, a behavioral activation strategy and strengthening social support. In addition, a psychoeducation component was included in the first PM+ session, where individuals were taught about common reactions to adversity, including experiences of CMDs.
**(Burgess et al., 2021)**	South Africa (Urban)	Adults	Other: Women who experience adversity	NGO offices	Specialist MH providers (counsellors/psychotherapists, social workers)	Psychotherapeutic (other); Social	Education; Skills	COURRAGE Plus: Group counselling intervention that combined collective narrative counselling with support and training in developing collective responses to economic and social hardships.
**(Carney et al., 2016)**	South Africa (Urban)	Mixed	Sex workers	Work locations Lifeline Drop-in centers	Lay-workers (CHW)	Psychotherapeutic (SC); Economic; Lifestyle/Physical health	Education; Skills; Monitoring; Referral; Other: Risk reduction plan	Inthubalethu Project: Community-based outreach and provision of HIV/AIDS and substance use education. Sessions contained the creation of a risk-reduction plan, HIV counselling and testing and necessary referrals to drug treatment, HIV/STI care and treatment, and other social services. Additional service was provision of counselling and personal growth courses, as well as skills development training in beading, sewing or hairdressing.

Author and Year	Geographic location (Urban/Rural)	Age group	Specific targets	Location of service delivery	Provider	Broad intervention category	Elements	Short description of intervention
**(Carney et al., 2019)**	South Africa (Urban)	Mixed	MSM; Other: Adolescent female school dropouts who use drugs and engage in risky sex	Other community places	Specialist MH providers (female interventionists)	Psychotherapeutic (PE); Lifestyle/Physical health; Social	Education; Skills	Young Women's Health CoOp: A woman-focused, behavioral intervention that addressed AOD use, gender-based violence, and sexual risk, with the primary goal of increasing skills and knowledge to reduce HIV risk. Additionally, HIV counselling and testing (HCT). It was an adapted version for young women.
**(Carney et al., 2020)**	South Africa (Urban)	Adolescents	Parents/care givers; Students	Other community places	Specialist MH providers (counsellors/psychotherapists)	Psychotherapeutic (CBT, MI);Social	Education; Skills; Self-help	Brief substance use intervention combining motivational interviewing with elements of CBT. It consisted of two intervention sessions for the participant (reduction of substance use, dealing with peer pressure, problem solving and readiness to change) and one separate intervention sessions for their primary caregiver (parenting skills, parent-child relationship, and information around substance use).
**(Carries et al., 2023)**	South Africa (Urban)	Adults	Parents/care givers; Affected by HIV/HIV +	Telehealth (SMS)		Economic	Support; Material	Economic incentive package: Caregivers of adolescents with HIV receive an economic incentive package comprising a monthly motivational SMS and cash to the value of ZAR 350 (23 USD) for three months. The SMS promote key behavioral economic principles (aspiration framing, loss aversion, altruism), aligned to social wellbeing dimensions that matter to people in this context.
**(Cetrone et al., 2021)**	Tanzania (Rural/semi-rural)	Adults	Parents/care givers; Other: farmers	Home	Lay-workers (peers)	Economic; Social	Education; Skills; Support; Material	Singida Nutrition and Agroecology Project: A participatory intervention that consisted of mentor farmers leading their village peers in participatory learning about sustainable farming, legume intensification, nutrition and women's empowerment. The mentor farmers facilitated learning exchanges during monthly community meetings and regular household visits. Additionally, each participating household received a mix of legume seeds.

Author and Year	Geographic location (Urban/Rural)	Age group	Specific targets	Location of service delivery	Provider	Broad intervention category	Elements	Short description of intervention
**(Chaudhury et al., 2016)**	Rwanda (Rural/semi-rural)	Mixed	Parents/care givers; Affected by HIV/HIV +	Home	Specialist MH providers (counsellors/psychot herapists)	Lifestyle/Physical health; Social	Education; Skills	Family-Strengthening Intervention for HIV-affected families (FSI-HIV): The intervention centered on the development of a family narrative to draw upon shared experiences, through four core intervention components: resilience, improved family communication on HIV transmission and status disclosure, and engagement of formal and informal supports.
**(Chipps et al., 2022)**	South Africa (Urban)	Adults	Students	Telehealth (Computer-based, WhatsApp)	N/A	Lifestyle/Physical health	Education; Skills; Self-help; Support; Material	eCOVID: Psychological first aid program which aimed to reduce COVID-19 related anxiety and concerns using the digital platforms of WhatsApp and the university learning management system. The online components of eCOVID were hosted on the university's learning management system and consisted of self-directed learning activities on COVID-19 and infection control. Stress management and support were provided through ongoing WhatsApp groups with messages delivered to the students' mobile phones.
**(Cilliers et al., 2016)**	Sierra Leone (Rural/semi-rural)	Adults	Other: Affected by war	Other community places	Lay-workers (CHW)	Social	Other: reconciliation /social bonding	Fambul Tok ("Family Talk" in Krio): As a part of its reconciliation program, committees composed of community members were trained in trauma healing and mediation and conducted outreach to encourage victims and perpetrators to participate in the truth-telling process. They also incorporated traditional rituals to promote community healing.
**(Cluver et al., 2018)**	South Africa (Mixed)	Mixed	Parents/care givers	Faith-based institution; Outdoors under trees	Specialist MH providers (social workers), Lay-workers (CHW)	Economic; Social	Education; Skills	Sinovuyo Teen: parenting program for adolescents and their caregivers. Session content was based on social learning principles and included praise and relationship building, managing stress and anger, family problem-solving, planning together to protect adolescents from community violence, monthly family budgeting, crises, saving and responding to crises.

Author and Year	Geographic location (Urban/Rural)	Age group	Specific targets	Location of service delivery	Provider	Broad intervention category	Elements	Short description of intervention
**(Coetzee et al., 2022)**	South Africa (Urban)	Adolescents	Students	School	Specialist MH providers (counsellors/psychotherapists)	Psychotherapeutic (CBT)	Education; Skills	4 Steps to my future (4STMF): A CBT based program during school lessons that will teach skills in 4 core steps: enhance self-esteem, promote helpful thinking, develop emotional regulation, and empower goal-focused action. It is designed to be active and engaging and uses a mix of whole group exercises, individual exercises, and different formats (speech, writing, reading of stories, role play, hand gestures, and visual posters).
**(Cohen et al., 2021)**	Uganda (N/A)	Adults	Refugees; Parents/caregivers	Home; Faith-based institution; Various community places	Lay-workers (Other)	Psychotherapeutic (PE); Social	Education; Skills	The Journey of Life (JoL) intervention focused on engaging caregivers in building awareness around child protection and fostered psychosocial support through reflection, dialogue, and action. The series of workshops included psychoeducation, self-care, positive parenting, understanding children's needs, identifying children who need help, and building on children's strengths.
**(Comrie-Thomson et al., 2022)**	Zimbabwe (Rural/semi-rural)	Mixed	Pregnant women; Parents/caregivers	Other community places	Health care workers (nurses), Lay-workers (CHW)	Psychotherapeutic (CBT); Economic; Lifestyle/Physical health	Education; Skills	The Mbereko+Men intervention included two gender-synchronized components implemented separately with women and men. The Mbereko component was delivered to women by trained local female VHWs who addressed mother and child health (MNCH) services and provided psychosocial support. The Mbereko component also included an internal saving and lending group for women. The +Men component was a discussion group for men led by a male nurse and focused on MNCH services and gender norms.
**(Connolly and Sakai, 2011)**	Rwanda (Urban)	Adults	Other: Survivors of genocide/war-Affected	Vacant rental home and back yard	Lay-workers (Other)	Psychotherapeutic (ET)	Skills; Self-help	Thought Field Therapy (TFT): Self-tapping of specific acupuncture points while recalling a traumatic event or cue. Each participant was provided with an individual TFT treatment session, administered by local community members.

Author and Year	Geographic location (Urban/Rural)	Age group	Specific targets	Location of service delivery	Provider	Broad intervention category	Elements	Short description of intervention
**(Constant et al., 2014)**	South Africa (Urban)	Adults	Other: Women undergoing medical abortion	Telehealth (SMS)		Lifestyle/Physical health	Education; Self-help; Support	Women undergoing medical abortion (MA) received timed, automated text messages delivered between clinic visits for mifepristone and follow-up of MA for "real-time" support and guidance. Thirteen timed text messages were sent including medication reminders as well as information on managing the bleeding, cramping and side effects while also highlighting potential problems, such as excess or no bleeding or fever in the days after the misoprostol.
**(Cooper et al., 2002)**	South Africa (Urban)	Adults	Pregnant women; Parents/care givers	Home	Lay-workers (CHW, peers)	Social	Education; Skills; Support	Mother-infant intervention: Home visits for pregnant women and mothers of newborns to encourage them in sensitive, responsive interactions with her infant and provide them with emotional support.
**(Cooper et al., 2009)**	South Africa (Urban)	Adults	Pregnant women; Parents/care givers	Home	Lay-workers (CHW, peers)	Social	Education; Skills; Support	Mother-infant intervention: Home visits for pregnant women and mothers of newborns to encourage them in sensitive, responsive interactions with her infant and provide them with emotional support.
**(Crombach and Siehl, 2018)**	Burundi (N/A)	Mixed	Refugees	Other community places	Specialist MH providers (counsellors/psychot herapists)	Psychotherapeutic (ET)	Skills	Narrative exposure therapy (NET) combines aspects of Testimony Therapy and Exposure Therapy in that patients were helped to re-enter their traumatic experiences while they recounted their autobiography to the therapist.
**(Cubbins et al., 2012)**	Zimbabwe (Rural/semi-rural)	Adults	Unspecified	Other community places	Lay-workers (peers)	Psychotherapeutic (PE); Lifestyle/Physical health	Education	Community Popular Opinion Leader (CPOL) intervention: Popular individuals or opinion leaders were used to spread culturally specific health-related messages to their friends and neighbors through casual conversations delivered in their own words: Primarily health-related messages involving reduction of risky sexual behaviors related to HIV (e.g., using a condom, practicing monogamy), but also not drinking alcohol excessively as a secondary message.
**(Dambi et al., 2022)**	Zimbabwe (N/A)	Adults	Unspecified	Telehealth (digital chat-based application)	Lay-workers (CHW)	Psychotherapeutic (CBT)	Education; Skills	Inuka intervention: A digital chat-based mental health application that consisted of problem-solving therapy sessions.

Author and Year	Geographic location (Urban/Rural)	Age group	Specific targets	Location of service delivery	Provider	Broad intervention category	Elements	Short description of intervention
**(Dawson et al., 2016)**	Kenya (Urban)	Adults	Gender-based violence; Other: women Affected by urban adversity	Home	Lay-workers (CHW)	Psychotherapeutic (CBT, SM)	Skills; Other: strengthening social Support	WHO Problem Management Plus (PM+) intervention consisting of four core intervention strategies taught by trained lay helpers. These strategies included managing stress through a slow breathing technique, a seven-step basic guide to managing practical problems, a behavioral activation strategy and strengthening social support. In addition, a psychoeducation component was included in the first PM+ session, where individuals were taught about common reactions to adversity, including experiences of CMDs.
**(de Fouchier and Kedia, 2018)**	Central African Republic (N/A)	Adults	Other: Humanitarian aid workers	NGO offices	Specialist MH providers	Psychotherapeutic (CBT, SM)	Education; Skills; Self-help	Stress management: Group intervention that comprised (1) psychoeducation on normal, chronic and traumatic stress and (2) strengthening of physical, cognitive, behavioral, spiritual and emotional coping strategies. Participants learned several techniques such as diaphragmatic breathing. A leaflet listing practical recommendations to cope with the symptoms of PTSD was also given to participants.
**(Draper et al., 2019)**	South Africa (Mixed)	Adults	Unspecified	Faith-based institution	Lay-workers (faith-based community member)	Lifestyle/Physical health	Education; Skills; Self-help	Impilo neZenkolo ('Health through Faith'): A group-based, free of charge, healthy lifestyle program with a strong Christian ethos, open to all; and recruited from church congregations, promoted by pastors/church leaders. Each session had an educational ('classroom') component covering diet, physical activity and weight loss, and behavior change techniques; and a practical group-based physical activity component.
**(Dunkle et al., 2020)**	Rwanda (N/A)	Adults	Gender-based violence; Other: couples	Other community places	Lay-workers (peers)	Economic; Social	Education; Skills	Indashyikirawa: The Couple's Curriculum incorporated the SASA! program's emphasis on positive and negative types and uses of power, critical personal reflection, and positive aspirations for healthy relationships. The curriculum also worked explicitly to address triggers of IPV (including disagreements about money, jealousy, and alcohol abuse) and emphasized skills building to create positive alternatives to violence.

Author and Year	Geographic location (Urban/Rural)	Age group	Specific targets	Location of service delivery	Provider	Broad intervention category	Elements	Short description of intervention
**(Duwell et al., 2013)**	South Africa (Urban)	Adults	Affected by HIV/HIV +	Home<br>Other community places	Lay-workers (Family member, peers)	Lifestyle/Physical health	Education; Skills; Support	Partial directly observed antiretroviral therapy (DOT-ART) by a chosen treatment supporter who were asked to observe and document at least one medication dose daily. Supporters and patients received ART adherence training. These sessions included training on how to fill pill boxes and complete the study adherence chart, as well as discussions on effective communication strategies, the provision of emotional support, and identification of barriers to adherence and potential solutions.
**(Edeh et al., 2022)**	Nigeria (Urban)	Mixed	Students; Other: business educators	Telehealth (Phone calls)	Specialist MH providers (counsellors/psychotherapists)	Psychotherapeutic (CBT, ET, SM)	Education; Skills	Trauma-focused CBT: Internet (Zoom) delivered intervention using exposure therapeutic procedures designed to assist participants in confronting dysfunctional thoughts, worries, fear, trauma and stress as well as high risk-stimuli caused by COVID-19. It involved systematic desensitization of pairing trauma-related memories and its reminders to muscle relaxation, as well as imaginal and vivo exposure.
**(Egbe et al., 2022)**	Kenya (N/A)	Adults	Other: men engaged in transactional sex	Telehealth (SMS)	N/A	Economic	Education; Skills; Support; Material	Akiba: Participant will receive assistance from study staff and local bank staff in Kenya in opening and using a savings account. In addition, they will receive an education session that emphasizes the importance of saving for the future, provides a tutorial on how to earn interest on savings, and addresses com-mon barriers to saving (lack of trust, lack of information, etc.). Participants will have a chance of winning monetary prizes each month based on the amount by which their account balance goes up during the month and will receive motivational SMS reminding the of their goals.
**(Egbegi et al., 2021)**	Nigeria (Urban)	Adolescents	Students	School	Specialist MH providers (counsellors/psychotherapists)	Psychotherapeutic (CBT, SM); Lifestyle/Physical health	Education; Skills	CBT strategies for insomnia: The group sessions focused on psychoeducation, sleep hygiene, restriction of day naps, relaxation techniques, and a cognitive strategy for managing bedtime worries.

Author and Year	Geographic location (Urban/Rural)	Age group	Specific targets	Location of service delivery	Provider	Broad intervention category	Elements	Short description of intervention
**(Eifediyi et al., 2018)**	Nigeria (Rural/semi-rural)	Mixed	Students	School	Specialist MH providers (counsellors/psychotherapists)	Psychotherapeutic (CBT)	Education; Skills	Rational emotive behavior therapy (REBT): An active, directive, solution-oriented therapy which focused on resolving emotional, cognitive, and behavioral problems in clients. The REBT entailed reconstruction of belief pattern of people from negative to positive.
**(Ekeke et al., 2022)**	Nigeria (N/A)	Adults	Other: leprosy/Buruli Ulcerosa patients	Other community places	Lay-workers (CHW)	Psychotherapeutic (SC)	Education; Support	The intervention consists of three components: 1) Engaging selected community members as lay counsellors to provide psychosocial support for persons affected by leprosy or BU with depression or general anxiety disorder. 2) Formation of self-help groups among persons affected by leprosy or BU with the aim to reduce stigma and improve quality of life through peer support. 3) Training of health-care workers in primary health care-units to screen for mental health problems and provide pharmacological and psychosocial treatment as well as referral.
**(Eller et al., 2013)**	South Africa; Puerto Rico, USA (N/A)	Adults	Affected by HIV/HIV +	Self-help with manual	Health care workers (nurses), Lay-workers (self-care)	Psychotherapeutic (other)	Education; Skills; Self-help	HIV/AIDS Symptom Management Manual: The participants were individually instructed in the use of the manual. The manual was divided into three sections. The ‘‘problem’’ section described depressive symptoms. The ‘‘treatment’’ section describes common treatments for depressive symptoms. The ‘‘self-care’’ section described self-care strategies for depressive symptoms.
**(Ertl et al., 2011)**	Uganda (N/A)	Mixed	Other: Child soldiers	Home	Lay-workers (CHW)	Psychotherapeutic (ET)	Education; Skills	Intervention arm 1: NET (kidNET): participant constructs a detailed chronological account of his or her own biography in cooperation with the therapist to reconstruct fragmented memories of traumatic events and to achieve habituation<br>academic catch-up program: 50% of the time English catch-up course, 50% of the time with elements of supportive counselling (psychoeducation, conducting discussions on coping with symptoms, and dealing with current problems)<br><br>Intervention arm 2: Academic catch up with supportive counselling. They also received psychoeducation on PTSD, sessions on coping with symptoms and dealing with current problems. In the last session, the participants received the English textbooks and exercise books they had been working on with their counselors.

Author and Year	Geographic location (Urban/Rural)	Age group	Specific targets	Location of service delivery	Provider	Broad intervention category	Elements	Short description of intervention
**(Ertl et al., 2021)**	Uganda (Urban)	Adults	Unspecified	Faith-based institution	Specialist MH providers (psychiatrists, counsellors/psychot herapists), Health care workers (laboratory technicians), Lay-workers (N/A)	Psychotherapeutic (CT, MI); Psychopharmacological	Education; Skills; Drug delivery; Support; Monitoring	Treatment Camp: One-week intensive inpatient detoxification program complemented by psycho-therapeutic content. It provided all necessary nutritional, medical and psychological care in one location for a predefined amount of time (usually 7 days). A key part of the treatment content was the introduction of the Alcoholics Anonymous philosophy and the strong recommendation to join AA meetings after the inpatient Camp phase.
**(Eseadi et al., 2022)**	Nigeria (Urban)	Mixed	Students	School	N/A	Psychotherapeutic (CBT)	Education; Skills	Religious rational emotive behavior therapy (RREBT): A faith-based mental health intervention created following the principles and practice of rational emotive behavior therapy. It integrated scriptural contents and other religious resources to address emotional and/or behavioral problems experienced by individuals.
**(Eze et al., 2021)**	Nigeria (Urban)	Adults	Other: Market traders	Telehealth (SMS); Other community places	Health care workers (physicians, dieticians, physical fitness counsellors)	Lifestyle/Physical health	Education; Skills; Self-help; Monitoring	Lifestyle/behavioral modification program: The program took place at the workplace (market) of participants (market traders) and consisted of health education on prevention, early detection, and control of hypertension through increased physical activity and dietary adjustment. At the end of the training, participants were given a health information leaflets. Participants also received weekly SMS with action points on prevention of hypertension and its risk factors. After training monthly visits to an outreach health post to check their blood pressure was offered.
**(Fabbri et al., 2021)**	Tanzania (Urban)	Adoles cents	Refugees; Students	School; Telehealth (SMS); Other community places	Lay-workers (peers)	Psychotherapeutic (CBT); Social	Education; Skills; Referral	EmpaTech: A classroom management and CBT-based intervention for groups of teachers to change negative thought and behavior pattern to corporal punishment. The intervention covered empathy building exercises and group work to learn and practice self-regulation techniques, positive disciplinary methods, and strategies to promote well-being. The teachers also received 2 SMS texts per week to reinforce aspects of the group sessions or homework.

Author and Year	Geographic location (Urban/Rural)	Age group	Specific targets	Location of service delivery	Provider	Broad intervention category	Elements	Short description of intervention
**(Fernando et al., 2021)**	Zimbabwe (Rural/semi-rural)	Adults	Pregnant women; Parents/care givers	Home	Lay-workers (CHW)	Psychotherapeutic (CBT)	Education; Skills; Referral	Friendship Bench intervention: VHWs conducted weekly home visits for 6 weeks, using a structured approach to identify problems and generate solutions (problem-solving therapy). VHWs were trained to identify 'red flags' indicating clinically significant depression or suicidal ideation. These women were referred to local clinics or discussed with the district mental health nurse.
**(Figge et al., 2022)**	Zambia (Urban)	Mixed	Unspecified	Telehealth (Phone calls)	Lay-workers (Other)	Psychotherapeutic (CBT)	Education; Skills	Telehealth-delivered Common Elements Treatment Approach (T-CETA): A transdiagnostic, multi-problem intervention that teaches CBT elements common to evidence-based treatments for trauma, anxiety, depression, and behavioral problems, and also how to combine these elements to treat different presenting problems and comorbidity. It involved delivery of varying elements and 'dose' of elements depending on client symptom and need and was adapted for telephone delivery.
**(Fine et al., 2021)**	Tanzania (N/A)	Mixed	Refugees; Parents/care givers	School; IRC wellness center in refugee camp	Lay-workers (peers)	Psychotherapeutic (CBT, SM); Social	Education; Skills	Early Adolescent Skills for Emotions (EASE) intervention: A brief, transdiagnostic group intervention to reduce internalizing symptoms. The sessions focused on evidence-based cognitive behavioral strategies including psychoeducation, stress management, behavioral activation, problem-solving, and relapse prevention. The intervention also included group sessions for caregivers, which focused on psychoeducation, active listening, slow breathing, positive parenting strategies, caregiver self-care, and relapse prevention.
**(Geffen et al., 2019)**	South Africa (Urban)	Adults	Other: older adults	Home; Telehealth (Phone calls)	Lay-workers (peers)	Lifestyle/Physical health; Social	Education; Support; Monitoring; Referral; Other: Wellness plan: goals and actions to achieve these goals	The AgeWell: Mentor/peer supporter concept that engaged older people to provide companionship to less-able older persons living in their communities. Peer supporters conducted home visits comprised of both social and wellness content.

Author and Year	Geographic location (Urban/Rural)	Age group	Specific targets	Location of service delivery	Provider	Broad intervention category	Elements	Short description of intervention
**(Gericke et al., 2021)**	South Africa (Urban)	Mixed	Students	School; Telehealth (Computer-based)	Specialist MH providers (counsellors/psychotherapists)	Psychotherapeutic (CBT)	Education; Skills; Self-help; Support	iCare: The iCBT intervention consisted of online sessions covering topics such as behavioral activation; reducing incongruence; overcoming difficulties and scheduling pleasant activities; psychoeducation; cognitive restructuring; problem-solving, exposure; and planning for the future. Students were responsible for working through the online material on their own and at their own pace, but anonymous eCoaches provided guidance and support using individualized structured feedback at the end of each session via email.
**(Giusto et al., 2020)**	Kenya (Urban)	Adults	Parents/caregivers	Neutral office space	Lay-workers (peers)	Psychotherapeutic (CBT, MI); Social	Education; Skills	Learn, Engage, Act, Dedicate (LEAD): The intervention addressed men's alcohol use, depressive symptoms, and co-occurring family problems using an approach based in motivational interviewing (MI), behavioral activation (BA), and masculinity discussion strategies and was delivered by peer-fathers.
**(Giusto et al., 2021)**	Kenya (Urban)	Adults	Parents/caregivers	Neutral office space	Lay-workers (peers)	Psychotherapeutic (CBT, MI); Social	Education; Skills	Learn, Engage, Act, Dedicate (LEAD): The intervention addressed men's alcohol use, depressive symptoms, and co-occurring family problems using an approach based in motivational interviewing (MI), behavioral activation (BA), and masculinity discussion strategies and was delivered by peer-fathers.
**(Giusto et al., 2022)**	Kenya (Urban)	Adults	Parents/caregivers	Neutral office space	Lay-workers (peers)	Psychotherapeutic (CBT, MI); Social	Education; Skills	Learn, Engage, Act, Dedicate (LEAD): The intervention addressed men's alcohol use, depressive symptoms, and co-occurring family problems using an approach based in motivational interviewing (MI), behavioral activation (BA), and masculinity discussion strategies and was delivered by peer-fathers.

Author and Year	Geographic location (Urban/Rural)	Age group	Specific targets	Location of service delivery	Provider	Broad intervention category	Elements	Short description of intervention
**(Glass et al., 2017)**	DRC (Rural/semi-rural)	Mixed	Unspecified	Home	Lay-workers (Other)	Economic	Skills; Support; Material	Pigs for Peace (PFP): A livestock productive asset transfer intervention, that gave female piglets to participants. Participants replayed the "credit" by transferring two piglets from the initial litter to other community members. Additionally, PFP provided practical skills training on managing nutrition and care of the livestock asset, biweekly home visits by trained staff, and basic health services by a local veterinarian technician.
**(Goodman et al., 2020)**	Kenya (Rural/semi-rural)	Adults	Unspecified	Other community places	Specialist MH providers (social workers)	Economic; Social	Education; Skills; Support	Kuja Pamoja/Come Together Intervention: A community-based intervention combining savings- and lending-groups, entrepreneurial training, and other skills training for families.
**(Green et al., 2016)**	Uganda (Rural/semi-rural)	Mixed	Unspecified	Other community places	N/A	Economic	Skills; Support; Material	Women's Income Generating Support (WINGS) program: The intervention consisted of business skills training, cash grants of approximately US$150, and ongoing follow-up support.
**(Green et al., 2019)**	Kenya (N/A)	Adoles cents	Orphans	School	Health care workers (nurses)	Economic; Lifestyle/Physical health	Education; Material; Monitoring	School support intervention consisting of three components: (a) payment of school tuition fees for secondary school; (b) provision of a school uniform in primary school and the first year of secondary school; and (c) nurse visits (nurses monitored school attendance, tracked the delivery of fees, and address health problems)
**(Green et al., 2020)**	Kenya (N/A)	Adults	Pregnant women; Parents/care givers	Telehealth (SMS)	Specialist MH providers (counsellors/psychot herapists)	Psychotherapeutic (CBT, SM)	Education; Skills; Self-help; Support; Monitoring; Referral	Healthy Moms: An artificial intelligence system named Zuri delivered automated psychological support via text messaging.

Author and Year	Geographic location (Urban/Rural)	Age group	Specific targets	Location of service delivery	Provider	Broad intervention category	Elements	Short description of intervention
**(Greene et al., 2019)**	Tanzania (Rural/semi-rural)	Adults	Refugees; Gender-based violence	NGO offices; Other community places	Lay-workers (Other)	Psychotherapeutic (CBT, SM); Social	Education; Skills; Referral	Nguvu: Single individual session of advocacy counselling followed by six group sessions focused on cognitive processing therapy and a final group session of advocacy counselling. Advocacy counselling focused on increasing autonomy, empowerment and strengthening linkages to community services by helping survivors process experiences of interpersonal violence and explore potential solutions.
**(Greene et al., 2021)**	Tanzania (Rural/semi-rural)	Adults	Refugees; Gender-based violence	Other community places	Lay-workers (CHW)	Psychotherapeutic (CBT, SC); Social	Education; Skills; Referral	The Nguvu intervention consisted of a single individual session of advocacy counselling followed by six group sessions focused on cognitive processing therapy and a final group session of advocacy counselling. Advocacy counselling focused on increasing autonomy, empowerment and strengthening linkages to community services by helping survivors process experiences of interpersonal violence and explore potential solutions.
**(Greene et al., 2022)**	Tanzania (Rural/semi-rural)	Adults	Refugees; Gender-based violence	NGO offices; Other community places	Lay-workers (Other)	Psychotherapeutic (CBT, SM); Social	Education; Skills; Referral	Nguvu: Single individual session of advocacy counselling followed by six group sessions focused on cognitive processing therapy and a final group session of advocacy counselling. Advocacy counselling focused on increasing autonomy, empowerment and strengthening linkages to community services by helping survivors process experiences of interpersonal violence and explore potential solutions.
**(Gupta and Zimmer, 2008)**	Sierra Leone (N/A)	Adoles cents	Refugees; Students	School	Lay-workers (teachers)	Psychotherapeutic (CT, ET)	Education; Skills	Rapid-Ed: Intervention that combined basic education with trauma healing activities. The activities focused on reducing the children’s levels of emotional distress and post-traumatic stress reactions such as difficulty concentrating, nightmares, flashbacks and hyper-vigilance. The children also participated in various recreational activities (i.e., jump rope, volleyball, athletics, football, ball tossing) that helped them release tension while providing respite from their bad memories and/or painful feelings.

Author and Year	Geographic location (Urban/Rural)	Age group	Specific targets	Location of service delivery	Provider	Broad intervention category	Elements	Short description of intervention
**(Habimana et al., 2021)**	Rwanda (N/A)	Adults	Other: genocide survivors	School; Other community places	Lay-workers (CHW)	Psychotherapeutic (SM)	Education; Skills	Community Resilience Model (CRM): The intervention helped genocide survivors to learn a set of self-regulation skills to increase their ability to respond to stress.
**(Harder et al., 2020)**	Kenya (Rural/semi-rural)	Adults	Unspecified	Telehealth (Phone calls)	Specialist MH providers (counsellors/psychotherapists, psychiatric nurses), Health care workers (physicians)	Psychotherapeutic (MI)	Education	Mobile motivational interviewing (MI): A mobile phone delivered motivational interview session delivered through a clinician.
**(Harding et al., 2019)**	Tanzania (Urban)	Adolescents	Orphans; Affected by HIV/HIV +	School; 5-day residential camp	Specialist MH providers (social workers), Health care workers (physicians)	Psychotherapeutic (ET)	Education; Skills	Memory Work Therapy (MWT): The intervention created a "safe space" in which the orphaned children could explore their life story and find and live out empowering parts of the stories that make up their lives. The intervention was conducted over a five-day residential camp during individual and group sessions.
**(Hatcher et al., 2020)**	Kenya (Rural/semi-rural)	Adults	Affected by HIV/HIV +; Other: farmers	Home; Faith-based institution	Lay-workers (Other)	Economic	Education; Skills; Material	Shamba Maisha: A multi-sectoral livelihood intervention that was comprised of three components: 1) a low-cost micro-irrigation water pump, which enabled farmers to irrigate their crops year-round, avoiding dependence on seasonal rainfall, 2) training on sustainable farming practices and financial management, and 3) loan program of vouchers (worth $150USD) to purchase the irrigation pump, seeds, fertilizers, and other farming implements.
**(Hermenau et al., 2011)**	Tanzania (Rural/semi-rural)	Adolescents	Orphans	Orphanage-based	Specialist MH providers (counsellors/psychotherapists)	Psychotherapeutic (ET); Social	Education; Skills	Instructional intervention and KIDNET, a child-friendly version of narrative exposure therapy (NET): The instructional system included training sessions for the caretakers that aimed for a better understanding towards the children and for a positive relationship between the two to reduce violent punishment and foster secure bonding. The KIDNET intervention was provided to children diagnosed with PTSD while the instructional interventions was aimed at all orphans living in the orphanage.

Author and Year	Geographic location (Urban/Rural)	Age group	Specific targets	Location of service delivery	Provider	Broad intervention category	Elements	Short description of intervention
**(Hoss et al., 2019)**	South Africa (Urban)	Adults	Gender-based violence	Local youth center	N/A	Psychotherapeutic (SM, other)	Education; Skills	Women empowerment community program: Intervention for women in systemically disadvantaged areas in South Africa and their experience of high levels of violence and traumatization. The sessions consisted of a blend of psychoeducation and person-centered group therapy using a narrative approach. The modules included a focus on self-enhancement, value clarification, building relationships, stress management and managing negative emotions, women's health, and gender-based violence and traumatization.
**(Igreja et al., 2004)**	Mozambique (Rural/semi-rural)	Adults	Other: war survivors	Home	Specialist MH providers (counsellors/psychotherapists)	Psychotherapeutic (ET)	Skills	Testimony method: A trauma exposure intervention for trauma survivors. The participants told the story of their traumatic experiences and, together with the interviewer, they created a narrative document of these experiences.
**(Im et al., 2018)**	Kenya (Urban)	Mixed	Refugees	Community-based organization	Lay-workers (peers)	Psychotherapeutic (PE)	Education	Trauma-informed psychoeducation (TIPE): The intervention included education on multifaceted impacts of trauma on the body, mind, social relationships, and spirituality, followed by psychosocial competencies, such as emotional coping and problem solving, community and support systems, and conflict management skills.
**(Ireri et al., 2019)**	Kenya (Urban)	Mixed	Other: Children with Autism Spectrum Disorder (ASD)	School	Specialist MH providers (counsellors/psychotherapists)	Psychotherapeutic (CBT)	Education; Skills; Support	Multimodal Anxiety and Social Skills Intervention (MASSI): The program was designed for adolescents, delivered across three modalities: individual therapy, group therapy (social skills training and practice) and family/school involvement. The parents and teachers were trained by observing and joining the child in reviewing their individual lessons and group practice.

Author and Year	Geographic location (Urban/Rural)	Age group	Specific targets	Location of service delivery	Provider	Broad intervention category	Elements	Short description of intervention
**(Ismayilova et al., 2018)**	Burkina Faso (Rural/semi-rural)	Adolescents	Unspecified	Home; Other community places	N/A	Economic; Social	Education; Skills; Support; Material	Trickle up plus: Combined economic strengthening and family-focused intervention. The intervention included four economic and livelihood strategies: 1) Savings group formation and training (weekly meetings), 2) Livelihood training and planning, 3) Seed capital grants, 4) Monthly one-on-one mentoring by field agents in participants homes. Additionally, a family coaching for all household members was conducted focusing on (1) schooling of children; (2) violence and abuse of children; (3) trafficking of children; (4) forced and early marriage; and (5) begging.
**(Jani et al., 2016)**	Ethiopia (Urban)	Adolescents	Other: adolescent rural-to-urban migrants	Service delivery program centers	Specialist MH providers (counsellors/psychotherapists)	Psychotherapeutic (CBT, CT); Lifestyle/Physical health	Education; Skills	Client-centered, psychosocial counselling intervention package. The intervention package was modelled on problem-solving therapy and comprised various counselling modalities, including individual, group and creative therapies, such as music, art and drama.
**(Jansen et al., 2022)**	Rwanda (N/A)	Unknown	Unspecified	Other community places	Lay-workers (CHW)	Psychotherapeutic (other); Social	Education; Skills	Community-Based Sociotherapy (CBS): A psychosocial community intervention aimed to enhance social bonding. Key elements were debates and the exchange of experiences and coping strategies among participants, exercises, games and mutual practical support.
**(Jewkes et al., 2008)**	South Africa (Rural/semi-rural)	Mixed	Unspecified	School	Lay-workers (CHW)	Lifestyle/Physical health; Social	Education; Skills	Stepping stones: Single sex group intervention that used participatory learning approaches. The sessions covered how we act and what shapes our actions; sex and love; conception and contraception; taking risks and sexual problems; unwanted pregnancy; sexually transmitted diseases and HIV; safer sex and condoms; gender-based violence; motivations for sexual behavior; dealing with grief and loss; and communication skills.
**(Jibunoh and Ani, 2021)**	Nigeria (Urban)	Adolescents	Students	School	Specialist MH providers (psychiatrists)	Psychotherapeutic (SM)	Education; Skills	Brief psychoeducation intervention: Three sessions of group-based psychoeducation about anxiety and relaxation techniques

Author and Year	Geographic location (Urban/Rural)	Age group	Specific targets	Location of service delivery	Provider	Broad intervention category	Elements	Short description of intervention
**(Jordans et al., 2013)**	Burundi (N/A)	Adoles cents	Students	Other community places	Lay-workers (CHW)	Social	Education	Brief parenting psychoeducation intervention for groups of parents that combined increasing awareness and understanding on psychosocial and mental health problems of children with information on problem management strategies, adapted from a manual for parents in helping children cope with the stresses of political violence.
**(Kagabo et al., 2023)**	Uganda; Rwanda (Urban)	Adults	Refugees	Other community places	Lay-workers (CHW)	Psychotherapeutic (other); Social	Education; Skills	Adapted form of Community-based sociotherapy (aCBS) for refugee settings: A psychosocial community intervention aimed to enhance social bonding. Key elements were debates and the exchange of experiences and coping strategies among participants, exercises, games, and mutual practical support.
**(Kalichman et al., 2019)**	South Africa (Urban)	Adults	Affected by HIV/HIV +	Telehealth (Phone calls)	Lay-workers (CHW)	Psychotherapeutic (CBT, MI); Lifestyle/Physical health	Education; Skills; Material; Referral	Phone-delivered behavioral self-regulation counselling: Client centered intervention to improve engagement in HIV care, that built on concepts from cognitive behavioral self-regulation delivered in a motivational interviewing framework.
**(Karnell et al., 2006)**	South Africa (Urban)	Adoles cents	Students	School	Lay-workers (peers)	Psychotherapeutic (CBT); Lifestyle/Physical health	Education; Skills	Our Times, Our Choices: An adapted American alcohol and HIV prevention curriculum for ninth grade students in South Africa that consisted of a series of monologues delivered by four fictional teenaged township characters. These performances served as the basis of class discussion and group assignments.
**(Khuzwayo et al., 2020)**	South Africa (Rural/semi-rural)	Mixed	Students	School	Lay-workers (CHW)	Psychotherapeutic (PE); Lifestyle/Physical health; Social	Education; Skills	Behavioral youth risk reduction intervention: School-based intervention, which took place during the life orientation (LO) periods. The sessions comprised the following topics: ‘knowing yourself’, ‘peer pressure’, ‘decision-making’, ‘healthy and unhealthy relationships’, ‘contraceptives’, ‘teenage pregnancy’, ‘condom use’, ‘HIV/ AIDS and STI prevention’, ‘alcohol and drug abuse’, ‘violence and gender-based violence’, ‘child support grant’, ‘human rights’ and ‘responsibilities in sexual health’.

Author and Year	Geographic location (Urban/Rural)	Age group	Specific targets	Location of service delivery	Provider	Broad intervention category	Elements	Short description of intervention
**(Kumakech et al., 2009)**	Uganda (Rural/semi-rural)	Adolescents	Orphans; Affected by HIV/HIV +	School	Lay-workers (teachers)	Psychotherapeutic (CBT); Lifestyle/Physical health; Social	Education; Skills; Drug; Support; Monitoring; Referral	A school-based peer-group support intervention combined with periodic somatic health checks and treatments for AIDS orphans. In the support interventions the participants were involved in exercises such as name games, blindfolded walk, sharing of past–present–future hopes, all of which were aimed at building trust within the group. Other topics discussed included approaches to problem solving, HIV/AIDS, fears about orphanhood and how to handle fear, and basic human needs and sources of satisfaction.
**(Kuo et al., 2020)**	South Africa (Urban)	Mixed	Parents/care givers	Other community places	Specialist MH providers (Other)	Psychotherapeutic (CBT); Lifestyle/Physical health; Social	Education; Skills	Our Family Our Future: A behavioral intervention for adolescent prevention of HIV and depression in combined adolescent-parent modules using role play, group activities, discussion and debate, and facilitation.
**(Lachman et al., 2020)**	Tanzania (Rural/semi-rural)	Mixed	Parents/care givers; Other: farmers	Other community places	N/A	Economic; Social	Education; Skills; Material	Skillful parenting: A group-based program that consisted of five sessions on parenting skills, two on child protection and five on family budgeting. It was delivered to farmer groups by Kiswahili-speaking professional trainers from Investing in Children and Societies, an international non-profit organization.<br><br>Agribusiness: A program that targeted food and income insecurity by providing small holder farmer groups access to drought-resistant seeds, credit for farm inputs, advice to improve farming techniques and market connections. The intervention was delivered to farmer group members by trained staff from a local economic enterprise initiative
**(Lasebikan et al., 2017)**	Nigeria (Rural/semi-rural)	Mixed	Unspecified	Other community places	Lay-workers (CHW)	Psychotherapeutic (MI)	Education; Self-help; Referral	Screening, brief intervention and referral to treatment (SBIRT): Community health extension workers screened participants for substance use and provided the patients with personalized feedback about their alcohol use. Lower risk clients received treatment as usual and were given feedback about their scores. Moderate risk clients received a 3-15 min brief intervention which comprised of giving feedback using simple motivational interviewing techniques. Clients were also given self-help strategies for cutting down or stopping alcohol use.

Author and Year	Geographic location (Urban/Rural)	Age group	Specific targets	Location of service delivery	Provider	Broad intervention category	Elements	Short description of intervention
**(Lawrence and Falaye, 2020)**	Nigeria (N/A)	Adolescents	Refugees; Students	School	Specialist MH providers (counsellors/psychotherapists)	Psychotherapeutic (CBT, ET)	Education; Skills	Experimental group 1: Trauma focused counselling to help clients to overcome trauma-related difficulties and enhance their psychosocial functioning ability, while group counselling technique was adopted. Experimental group 2: social effectiveness skill training was to help clients reduce social anxiety, increase interpersonal skills and improve social competence for psychological functioning in a group modality.
**(L'Engle et al., 2014)**	Kenya (Urban)	Adults	Sex workers	Drop-in centers	Specialist MH providers (psychiatric nurses)	Psychotherapeutic (MI); Lifestyle/Physical health	Education; Material; Referral	Brief alcohol intervention: Intervention based on the WHO Brief Intervention for Alcohol Use. Motivational interviewing techniques, a focus on goal setting and increasing self-efficacy for changing behavior, the provision of positive feedback and encouragement for change, and use of counselling notes and noting stage of change for alcohol reduction were essential intervention elements. Additionally, participants, were tested and counseled for HIV if they were negative at previous study visits, received a gynecological examination that included laboratory testing for STIs, and were provided condoms and relevant referrals and treatment if indicated.
**(le Roux et al., 2020)**	South Africa (Rural/semi-rural)	Mixed	Pregnant women; Parents/care givers	Home	Lay-workers (CHW)	Lifestyle/Physical health; Social	Education; Skills; Support; Referral	Philani program: In addition to access to clinic antenatal services, home visits were conducted. The CHWs aimed to reduce mother's risk of acquiring HIV; follow protocols to prevent maternal to child transmission (PMTCT); improve maternal and child health including tuberculosis and illness detection; reduce maternal alcohol use; improve infant and child nutrition; and foster children's growth and development. A critical aspect of the intervention was the peer support provided to mothers who were struggling by women from their own community, who have faced similar challenges.
**(Lloyd-Sherlock et al., 2019)**	Ghana (Urban)	Adults	Other: elderly	Day center for elderly	Specialist MH providers (psychiatrists)	Psychotherapeutic (other)	Education; Referral	SAGE screening algorithm: In a day center run by an NGO older people were screened for depression using SAGE and then reassessed by a psychiatrist (validity of screening). People with depression or mood or psychosocial problems received a group psychotherapy provided by a trained psychiatrist.

Author and Year	Geographic location (Urban/Rural)	Age group	Specific targets	Location of service delivery	Provider	Broad intervention category	Elements	Short description of intervention
**(Logie et al., 2021)**	Uganda (Urban)	Mixed	Refugees	Home; Telehealth (Phone calls)	Lay-workers (peers)	Lifestyle/Physical health	Education; Skills; Self-help; Support; Material; Referral	Experimental arm 1: HIV self-testing: Patients receive HIV self-testing kits and receive material such as booklets, condoms and lubricants and referral cards. They can also contact the peer navigator if they need additional information (via phone) or need support to go for confirmatory tests at local clinics. At follow-up visits, peer navigators will check in with participants about the HIV self-testing kit, distribute another HIV self-testing kit and condoms/lubricant, and screen for adverse events.<br><br>Experimental arm 2: HIV self-testing + mHealth strategies (supportive text messages). Same as experimental arm 1 + enrolled on a web-based SMS platform. They receive weekly supportive bidirectional text messages asking how they are doing.
**(Logie et al., 2022)**	Uganda (Urban)	Mixed	Refugees	NGO offices; Telehealth (SMS, Virtual reality)	Lay-workers (peers)	Psychotherapeutic (CBT, SM)	Education; Skills; Support; Monitoring; Referral	Tushirikiane4MH virtual reality (VR) and group problem management+ (GPM+): The intervention consists of an interactive 15-minute VR session. The VR scenario integrates mental health literacy, psychological first aid, and mindful self-compassion information and activities. GPM+, a WHO brief psychological transdiagnostic intervention employed evidence-based approaches to stress management, problem-solving, behavioral activation, and social support to reduce a range of mental health concerns. Additionally, mHealth support, including SMS bidirectional check-ins, SMS mental health literacy messages, and supportive WhatsApp conversations.
**(Lund et al., 2013)**	Kenya (Rural/semi-rural)	Adults	psychosis/schizophrenia; Other: having severe mental or neurological disorder	Home; Faith-based institution	Lay-workers (CHW)	Psychotherapeutic (SC); Economic	Education; Skills; Support; Referral	BasicNeeds' mental health and development program: The intervention included community engagement meeting to identify participants and to raise awareness in the community, and self-help support groups for people with mental illness and their carers. In the support groups participants were able to tell of their experience of illness and receive psychosocial support. The self-help groups also comprised livelihoods programs such as training in craft, agricultural or business skills, and the initiation of income-generating activities by participants.

Author and Year	Geographic location (Urban/Rural)	Age group	Specific targets	Location of service delivery	Provider	Broad intervention category	Elements	Short description of intervention
**(Lund et al., 2014)**	South Africa (Urban)	Adults	Pregnant women; Parents/care givers	Home (Participants can choose if home or clinic)	Lay-workers (CHW)	Psychotherapeutic (CBT, SM)	Education; Skills	Task-shared counselling intervention: Participants will receive six counselling sessions, which include aspects of psychoeducation, problem solving therapy, behavioral activation, cognitive reframing (healthy thinking), and relaxation training.
**(Marfo and Owusu-Daaku, 2016)**	Ghana (Urban)	Adults	Other: adults with high blood pressure/diab etes	Pharmacy	Health care workers (pharmacists)	Lifestyle/Physical health	Education; Self-help; Monitoring; Referral	Pharmacist-led hypertension preventative and detection intervention: Intervention strategies employed included screening (measuring BP and BMI), identification of other modifiable risk factors, health education, follow-up, and referral to a medical practitioner as necessary.
**(Masulani-Mwale et al., 2019)**	Malawi (Urban)	Adults	Parents/care givers	School	N/A	Psychotherapeutic (SC, other); Social	Education; Skills	Titukulane: intervention for training parents of children with intellectual disabilities.
**(Matsuba et al., 2020)**	Uganda (Rural/semi-rural)	Adoles cents	Students	School	Lay-workers (teachers)	Psychotherapeutic (SM)	Education; Skills	MindUp: Social and emotional learning mindfulness-based school program for children in post-conflict Northern Uganda that incorporated mindfulness practices with activity-based opportunities to learn about their brain, understand how thoughts and feelings affect actions, and strategies to become caring people.
**(McLea and Mayers, 2017)**	South Africa (Urban)	Adults	Unspecified	Faith-based institution	Specialist MH providers (social workers)	Psychotherapeutic (other)	Education; Skills; Referral	Grief and trauma project: Group intervention that provided psychological and spiritual support. Each session comprised of a scripture reading, prayer, a verbal expression of a feeling experienced, and a range of skills to manage the context they find themselves in. Exercises to enable participants to openly speak of emotional states were included in each session. Participants shared their stories.
**(McMullen and McMullen, 2018)**	Uganda (N/A)	Adoles cents	Students	School	Lay-workers (teachers)	Psychotherapeutic (CBT, other)	Education; Skills	Living well: A school-based, teacher-led, life skills-focused, and manualized intervention designed to support the social, emotional, and behavioral development of youth through promoting mental health and resilience, developing communication skills, supporting good choices, and learning life skills to help in their present and their future.

Author and Year	Geographic location (Urban/Rural)	Age group	Specific targets	Location of service delivery	Provider	Broad intervention category	Elements	Short description of intervention
**(Moffett et al., 2022)**	South Africa (Rural/semi-rural)	Mixed	Unspecified	Telehealth (Computer-based, SMS)	Lay-workers (peers)	Psychotherapeutic (CBT)	Education; Skills; Self-help; Support; Monitoring; Referral	Kuamsha program: The intervention arm will receive six modules of behavioral activation therapy via a smartphone application (the Kuamsha app) supported by trained peer mentors.
**(Monnapula-Mazabane and Petersen, 2022)**	South Africa (Urban)	Adults	Parents/care givers	Other community places	Specialist MH providers (counsellors/psychot herapists)	Psychotherapeutic (SM); Social	Education; Skills	The anti-stigma mental health intervention covered five critical topics for caregivers that addressed common misconceptions about mental illness: general mental health education, mental health stigma, communication and behavior management, caregiver well-being, and coping strategies.
**(Morojele et al., 2014)**	South Africa (Urban)	Adults	Other: Bar patrons	Other: Bar	Specialist MH providers (counsellors/psychot herapists), Lay-workers (peers)	Psychotherapeutic (MI); Lifestyle/Physical health	Education; Support; Material; Referral	Vuwani Program: (1) identification and training of suitable bar patrons to serve as peer interventionists or ‘change agents’ (Sisonke mentors); (2) deploying counsellors to the drinking venues to conduct screening and provide education, advice, and counselling, as well as referrals to formal counselling and treatment services; (3) assisting bar owners/managers to establish physical conditions that foster responsible drinking and risk reduction; (4) disseminating prevention messages and information via posters, leaflets, and other media; and 5) making affordable and free condoms consistently available at drinking venues.
**(Mpande et al., 2013)**	Zimbabwe (Rural/semi-rural)	Adults	Other: Torture survivors	Other community places; Other: Bar	Lay-workers (CHW)	Psychotherapeutic (ET)	Education; Skills; Support	Tree of Life trauma healing workshop (TOL): A group-based approach to the healing and empowerment of survivors of organized violence and torture. It was facilitated by survivors themselves and used the metaphor of the tree to provide a framework for understanding the trauma experience, and, through a series of inter-related processes, leads the survivor into an appreciation of his or her strength and the support of the community in surviving. It was carried out over a period of two to three days with a group living and sharing meals together.

Author and Year	Geographic location (Urban/Rural)	Age group	Specific targets	Location of service delivery	Provider	Broad intervention category	Elements	Short description of intervention
**(Muriungi and Ndetei, 2013)**	Kenya (Urban)	Adults	Students	School	Specialist MH providers (counsellors/psychot herapists)	Psychotherapeutic (PE)	Education	Psychoeducation: Psychoeducation sessions that comprised lectures, simulations, group discussions and role-plays. They incorporated predisposing/precipitating causes of depression, hopelessness, suicidality, anxiety, and substance abuse, as well as symptom recognition and stress-coping skills/ strategies.
**(Murray et al., 2018)**	Ethiopia (Rural/semi-rural)	Mixed	Refugees; Parents/care givers	Home; NGO offices; Confidential locations	Lay-workers (CHW)	Psychotherapeutic (CBT, ET, SM); Social	Education; Skills; Referral	Common elements treatment approach (CETA): An approach that teaches CBT elements common to evidence-based treatments for trauma, anxiety, depression, and behavioral problems, and also how to combine these elements to treat different presenting problems and comorbidity. It involved delivery of varying elements and 'dose' of elements depending on client symptom and need. Caregiver were also involved.
**(Musyoka et al., 2023)**	Kenya (Urban)	Mixed	Students	School Telehealth (Application)	Lay-workers (peers)	Psychotherapeutic (SC)	Education; Support; Referral	MHealth-based interventions: Peer mentors were trained to use the mHealth-based intervention tool, which was a digital android-based problem identification, targeted brief intervention, and referral tool. They screened at-risk students and provided brief interventions and referrals as necessary.
**(Mutamba et al., 2018)**	Uganda (Rural/semi-rural)	Adults	Parents/care givers Other: Parents' children with nodding syndrome	Other community places	Lay-workers (CHW)	Psychotherapeutic (IPT)	Education; Skills	Group interpersonal psychotherapy (IPT), a brief attachment-focused PT that was initially designed to treat depressive symptoms by improving interpersonal functioning and social support, was delivered in a group setting.
**(Namasaba et al., 2022)**	Uganda (Urban)	Adults	Parents/care givers	School; Telehealth (Phone calls)	Specialist MH providers (psychiatrists, other), Lay-workers (peers	Psychotherapeutic (CBT)	Education; Skills; Support	Multi-Modal CBT: Phase one: Group-based CBT for home and school caregivers delivered by clinical psychiatric officer and occupational therapist. Phase two: Phone-based CBT delivered by peer mentors.

Author and Year	Geographic location (Urban/Rural)	Age group	Specific targets	Location of service delivery	Provider	Broad intervention category	Elements	Short description of intervention
**(Namy et al., 2021)**	Uganda (Urban)	Mixed	Other: Human trafficking survivors	Shelter	Lay-workers (Other)	Psychotherapeutic (SM); Lifestyle/Physical health	Skills	Healing and Resilience after Trauma (HaRT) Yoga: Group-based intervention for women and girls who have experienced human trafficking that integrated physical yoga poses alongside breathwork, guided visualizations, mindfulness practices, and a 'closing circle' where participants shared their experiences, asked questions, and provided input into future sessions.
**(Negash et al., 2021)**	Ethiopia (Urban)	Adults	Students	School	Specialist MH providers (counsellors/psychotherapists)	Psychotherapeutic (IPT)	Education; Skills	Interpersonal psychotherapy: A culturally adapted talk therapy to treat clients with CMDs caused by loss, life transitions/role changes, and inter-personal conflicts, that included orientation to mental health in Ethiopia and interpersonal psychotherapy.
**(Nwobi et al., 2018)**	Nigeria (Mixed)	Adults	Affected by HIV/HIV +	Other community places	Specialist MH providers (counsellors/psychotherapists)	Psychotherapeutic (CBT, SM)	Education; Skills	Group-based cognitive behavioral stress management intervention: Group sessions combining relaxation training (e.g., imagery, progressive muscle relaxation, meditation) and stress management (e.g., cognitive restructuring, coping strategies, establishing a strong social network).
**(Nyongesa et al., 2022)**	Kenya (Urban)	Adults	Affected by HIV/HIV +	Telehealth (Phone calls)	Lay-workers (CHW)	Psychotherapeutic (CBT, SM)	Education; Skills	Problem management plus: By WHO developed intervention, that consists of four core intervention strategies taught by trained lay helpers. These strategies included managing stress through a slow breathing technique, a seven-step basic guide to managing practical problems, a behavioral activation strategy called 'Get Going, Keep Doing' and strengthening social support. In addition, a psychoeducation component was included in the first PM+ session, where individuals were taught about common reactions to adversity, including experiences of CMDs.
**(O'Callaghan et al., 2014)**	DRC (Rural/semi-rural)	Adolescents	Other: Affected by war	Faith-based institution	Lay-workers (CHW)	Psychotherapeutic (SM); Social	Education; Skills	Communities participative psychosocial intervention: Group intervention for participants and their caregivers covering psychoeducation, relaxation techniques, brainstorming major problems in families, improving interpersonal communication in families, major problems in the community, conflict resolution within the community, and effective parenting/youth contribution to the community.

Author and Year	Geographic location (Urban/Rural)	Age group	Specific targets	Location of service delivery	Provider	Broad intervention category	Elements	Short description of intervention
**(Odenwald et al., 2012)**	Somalia (N/A)	Adults	Parents/care givers; psychosis/schizophrenia	Home	Specialist MH providers (psychiatrists, social workers)	Psychotherapeutic (MI); Psychopharmacological	Education; Drug; Support; Monitoring; Referral	Home-based treatment package and relapse prevention: The intervention included in the acute treatment phase home visits for psychoeducation, diagnostic interviews, prescription, and adjustment of medication by an experienced psychiatrist. In the second phase a relapse prevention including monitoring of symptoms and side effects, repeated psychoeducation, motivation to adhere to the prescribed treatment, motivation to stop using khat, the discussion of current problems, active listening to the carers' and patients' problems and emotional support was conducted by local staff. Additionally, the patients received the prescribed drugs during home visits and the prescription regimen was explained and discussed in detail with both carers and patients.
**(O'Donnell et al., 2014)**	Tanzania (Mixed)	Adolescents	Orphans	Home; Other community places	Lay-workers (Other)	Psychotherapeutic (CBT, ET); Social	Education; Skills	The trauma-focused CBT for childhood traumatic grief: Group intervention (separate groups for guardians and children), that built a foundation for understanding how loss affects children, taught relaxation and coping skills, and delivery of trauma narrative, where the children talked about memories. Additionally, children and guardians were individually visited. In the guardian group parenting skills were taught.
**(Ofoegbu et al., 2020)**	Nigeria (N/A)	Mixed	Students	Telehealth (Phone calls, SMS, Computer-based, E-mail)	Specialist MH providers (counsellors/psychotherapists)	Psychotherapeutic (CBT, SC)	Education; Skills; Self-help	Guided internet-assisted intervention (GIAI) covering psychoeducation, interactive peer support, cognitive disputation, behavioral homework assignments, role-play, and depression management. The participants were asked to work independently with the program while the guidance therapists provided twice-a-week assistance within the portals and through email and telephone.
**(Ogum Alangea et al., 2020)**	Ghana (Rural/semi-rural)	Adults	Unspecified	Home; Other community places	Lay-workers (CHW)	Psychotherapeutic (SC); Social	Education; Skills; Support; Referral	Rural Response System (RRS) intervention: Community-based action teams were used to prevent intimate partner violence (IPV) by raising awareness during different opportunities (e.g., religious groupings, weddings) and supporting survivors (e.g., counselling for couples affected by IPV, referrals).

Author and Year	Geographic location (Urban/Rural)	Age group	Specific targets	Location of service delivery	Provider	Broad intervention category	Elements	Short description of intervention
**(Okube et al., 2022)**	Kenya (Urban)	Adults	Other: adults with metabolic syndrome	Other community places	Health care workers (nurses), Lay-workers (CHW)	Lifestyle/Physical health	Education; Skills; Referral	Community-based lifestyle intervention: The intervention included delivery of individualized health education, and recommendations and adherence counselling on lifestyle changes to reduce risk factors for cardiovascular diseases. Additionally, the participants who were not aware of diabetic/hypertensive status but found to have high BP and/or blood sugar were referred to health facilities.
**(Olowokere and Okanlawon, 2014)**	Nigeria (N/A)	Adoles cents	Students	School	Health care workers (nurses), Lay-workers (teachers)	Psychotherapeutic (other); Social	Education; Skills; Support	Resilience training: Intervention that focused on improving children knowledge and skills in the core resilience characteristics which included self-reliance, being oneself (existential aloneness), maintaining balance and harmony with self (equanimity), having a sense of purpose, and perseverance. Local songs that promoted resilience, lecture, discussions, brainstorming, craftwork, and assignments were used to teach resilience.<br><br>Peer support group: The support group was used to facilitate interaction between vulnerable children and to encourage sharing of feelings, ideas, and information on coping techniques in different situations. Basic life skills support was taught during the support group meetings using a standardized module that covered topics such as self-awareness, interpersonal communication, critical thinking, decision making, problem solving, coping with feelings, empathy, and creative thinking.<br>Experimental group 3 (E3): combination of interventions used by E1 and E2.

Author and Year	Geographic location (Urban/Rural)	Age group	Specific targets	Location of service delivery	Provider	Broad intervention category	Elements	Short description of intervention
**(Olowokere and Okanlawon, 2018) 12/12/2023 19:31:00**	Nigeria (N/A)	Adoles cents	Students	School	Lay-workers (teachers, Other)	Psychotherapeutic (other)	Education; Skills	Resilience training: Improving children knowledge and skills in the core resilience characteristics which included self-reliance, being oneself (existential aloneness), maintaining balance and harmony with self (equanimity), having a sense of purpose, and perseverance. Local songs that promoted resilience, lecture, discussions, brainstorming, craftwork, and assignments were used to teach resilience.<br><br>Peer support group used to facilitate interaction between vulnerable children and to encourage sharing of feelings, ideas, and information on coping techniques in different situations. Basic life skills support was taught during the support group meetings using a standardized module that covered topics such as self-awareness, interpersonal communication, critical thinking, decision making, problem solving, coping with feelings, empathy, and creative thinking.
**(Onyechi et al., 2016)**	Nigeria (Mixed)	Adults	Parents/care givers; Other: Cancer patients	Home	Specialist MH providers (counsellors/psychot herapists)	Psychotherapeutic (CBT)	Education; Skills; Monitoring	Rational emotive hospice care therapy (REHCT): Intervention for dying cancer patients and their care givers that addressed coping with death and avoiding self-defeating thoughts.
**(Onyut et al., 2004)**	Uganda (Rural/semi-rural)	Mixed	Refugees	Home; NGO offices	Lay-workers (CHW, peers)	Psychotherapeutic (ET)	Education; Skills	Nakivale camp project, Narrative Exposure Therapy (NET): NET combined aspects of testimony therapy and exposure therapy in that patients were helped to re-enter their traumatic experiences while they recounted their autobiography to the therapist who documented it.
**(Osafo and Andoh-Arthur, 2020)**	Ghana (N/A)	Adults	Unspecified	Telehealth (Phone calls, TV program)	N/A	Social	Education; Support	TV program organized to mark the World Suicide Prevention Day with interviews including a suicide attempt survivor. The audience was encouraged to ask questions, and in seeking clarification and counsel via the first author whose contact details were made public. Additionally, availability of lines to call in for help if the viewers' so desired. The contact details of the guests were also screened for the public to contact the experts for further consultation and help.

Author and Year	Geographic location (Urban/Rural)	Age group	Specific targets	Location of service delivery	Provider	Broad intervention category	Elements	Short description of intervention
**(Osborn et al., 2020a)**	Kenya (Urban)	Adoles cents	Students	School; Telehealth (Computer-based)	Lay-workers (peers)	Psychotherapeutic (other)	Education; Skills; Self-help	Shamiri-Digital: A psychological intervention that focused on positive human attributes rather than psychopathology. The adapted online version from the group-based Shamiri intervention consisted of three modules: growth mindset, gratitude, and value affirmation.
**(Osborn et al., 2020b)**	Kenya (Urban)	Adoles cents	Students	School	N/A	Psychotherapeutic (other)	Education; Skills	Shamiri: A psychological intervention that focused on positive human attributes rather than psychopathology. The intervention included 3 modules: growth mind- set (2 sessions), gratitude (1 session), and value affirmation (1 session).
**(Osborn et al., 2021)**	Kenya (Urban)	Adoles cents	Students	School	Lay-workers (peers)	Psychotherapeutic (other)	Education; Skills	Shamiri: A psychological intervention that focused on positive human attributes rather than psychopathology. The intervention included 3 modules: growth mind- set (2 sessions), gratitude (1 session), and value affirmation (1 session).
**(Osiki and Busari, 2002)**	Nigeria (Urban)	Adoles cents	Students	School	N/A	Psychotherapeutic (CBT)	Education;Skills	Self-statement monitoring technique to reduce test anxiety during a 8 week treatment period.
**(Pandya, 2018)**	South Africa; Zimbabwe; Ethiopia; India, China, Somalia, Botswana, Belarus, Czech Republic, Poland, Spain, US and Canada (N/A)	Adoles cents	Orphans	Orphanage-based	Lay-workers (spiritual trainers)	Psychotherapeutic (SM)	Skills	Spiritual intervention program: A week-long course on self-awareness, relationships, positive thinking, breath control and meditation. Conducted at an interval of 4 months (i.e. total three times) during the study period.

Author and Year	Geographic location (Urban/Rural)	Age group	Specific targets	Location of service delivery	Provider	Broad intervention category	Elements	Short description of intervention
**(Parry et al., 2017)**	South Africa (Mixed)	Mixed	Unspecified	Other community places	Lay-workers (peers)	Lifestyle/Physical health	Education; Skills; Material; Referral; Other: risk-reduction counselling	Local adaptation of the World Health Organization's Training guide for HIV prevention: Development of a risk-reduction plan together with the client, which consisted of non-injection drug use related risks, sex-related risks and plans for HIV counselling and testing. The intervention session which was then offered to the clients included education about HIV, condom demonstrations and the provision of referrals as needed. At the follow-up appointment, the risk- reduction plan was discussed again with the client to assess behavior change and revise risk-reduction plans.
**(Patel and Ross, 2020)**	South Africa (Urban)	Adults	Parents/care givers	School	Specialist MH providers (social workers)	Economic; Social	Education; Skills; Support	Cash transfer (Child Support Grant (CSG)) plus family strengthening intervention Sihleng'imizi program: The family intervention was designed to build on family strengths and addressed particular risk factors associated with low levels of child and caregiver well-being, with the purpose of improving positive parenting practices through equipping caregivers with appropriate knowledge, skills and information. Each family was paired with another family (Sihleng'imizi buddy) to support each other between sessions and after the groups terminated.
**(Pengpid et al., 2013)**	South Africa (Urban)	Adults	Students	School	Health care workers (nurses)	Psychotherapeutic (CBT, MI)	Education; Skills; Self-help	Brief Intervention: Participants received personalized feedback on their AUDIT results, a health education leaflet, simple advice plus brief counselling about reducing excessive drinking, during a one-time 20 min session.
**(Pengpid et al., 2014)**	South Africa (Rural/semi-rural)	Adults	Other: high blood pressure/diab etes	Faith-based institution	Lay-workers (CHW)	Lifestyle/Physical health	Education; Skills; Monitoring	lifestyle intervention: the program consists of six two-hour group sessions facilitated by trained lay health promoters. The group counselling sessions provide individuals with dietary and physical activity guidance based on the South African food-based dietary guidelines for adults and South African physical activity recommendations. The Health Action Process Approach (HAPA) model and self-regulation theory are used to set individual goals and motivate participants to progress from intention to actual behavior change to accomplish the diet and physical activity goals of the program.

Author and Year	Geographic location (Urban/Rural)	Age group	Specific targets	Location of service delivery	Provider	Broad intervention category	Elements	Short description of intervention
**(Pettifor et al., 2018)**	South Africa (Rural/semi-rural)	Adults	Unspecified	Home; Other community places Streets, bars	Lay-workers (Other)	Social	Education; Skills	Community mobilization (CM): Intervention activities comprised workshops, community activities (e.g., door-to-door visits, street soccer, discussion in bars, film screening, mural designs) and leadership engagement with workshops and activity content focused on seven areas; (1) gender, power, and health, (2) gender and violence, (3) alcohol abuse,(4) gender, HIV and AIDS, (5) healthy relationships, (6) human rights, and (7) taking action for change.
**(Prencipe et al., 2021)**	Tanzania (N/A)	Mixed	Unspecified	Other: Cash transfer		Economic	Material	Conditional cash transfers (CCT): The main element of the program was a monthly CTs, wherein a base household transfer, larger for households with children, was unconditional, while additional amounts were contingent on fulfilling requirements related to children's school attendance and health care visits. The CCT was complemented with a public works program (PWP), wherein temporary paid work is made available for one able-bodied adult per beneficiary household, during the lean season (up to 4 months).
**(Prinsloo et al., 2016)**	South Africa (Urban)	Adults	Affected by HIV/HIV +	Home; Faith-based institution; Other community places	Lay-workers (peers)	Social	Education; Skills; Self-help; Support	Community HIV stigma reduction hub network: People living with HIV and people living close to them shared their knowledge on HIV stigma reduction and mobilized and empowered their own community in HIV stigma reduction. Activities included workshops, door-to-door education, support groups and psychodrama group performances.
**(Puffer et al., 2020)**	Kenya (Urban)	Mixed	Parents/care givers	Home	Lay-workers (CHW)	Psychotherapeutic (CBT); Social	Education; Skills	Tuko Pamoja (TP): A family therapy intervention that aimed to create a positive family environment supportive of positive child and adult outcomes. The intervention was manualized and organized by modules. Core modules addressed problems in different family relationships: couples, parent–child, and overall family cohesion/organization.
**(Rabie et al., 2022)**	South Africa (Urban)	Adults	Unspecified	Other community places	Lay-workers (CHW)	Lifestyle/Physical health; Social	Education; Skills; Referral	Soccer League (SL): Men in the SL group received soccer training and matches over a 6-month period. At the start of each practice and match, participants practiced roleplaying situations, implementing prosocial relationships in a group format before playing soccer.

Author and Year	Geographic location (Urban/Rural)	Age group	Specific targets	Location of service delivery	Provider	Broad intervention category	Elements	Short description of intervention
**(Ramdhonee-Dowlot et al., 2021)**	Mauritius (Mixed)	Adolescents	Unspecified	Other: Residential care institutions (RCIs)	Specialist MH providers (counsellors/psychotherapists)	Psychotherapeutic (CBT)	Education; Skills	Super Skills for life (SSL): The intervention had five core principles: (1) it was based on a transdiagnostic approach that targets common core risk factors (e.g. low self-esteem, lack of social skills) of comorbid disorders; (2) it contained elements of CBT; (3) it used video feedback with cognitive preparation to help children enhance their self-perception; (4) it used the principle of behavioral activation by having children increase their activity levels and participate in positive and rewarding activities; and (5) finally, it taught children basic skills to use during social interactions.
**(Reeler et al., 2009)**	Zimbabwe (Rural/semi-rural)	Unknown	Other: Torture survivors	Other: natural setting	Lay-workers (peers)	Psychotherapeutic (ET, SM)	Education; Skills; Support	The Tree of Life: A group-based approach to the healing and empowerment of survivors of organized violence and torture, facilitated by survivors themselves and using the metaphor of the tree to provide a framework for understanding the trauma experience. Through a series of inter-related processes, it leads the survivor into an appreciation of his or her strength and the support of the community in surviving. It was carried out over a period of two to three days with a group living and sharing meals together.
**(Richards et al., 2014)**	Uganda (Urban)	Adolescents	Students	Other: central sports fields	Lay-workers (CHW)	Lifestyle/Physical health; Social	Education; Skills	Gum Marom Kids League (GMKL): A sports intervention that used sport as a vehicle to promote physical fitness and mental health as well as achieve peace-building objectives in the community.
**(Rivet-Duval et al., 2011)**	Mauritius (N/A)	Adolescents	Students	School	Lay-workers (teachers)	Psychotherapeutic (CBT, IPT)	Education; Skills	Resourceful adolescent program, adolescent version (RAP-A): A manualized group treatment program that included both CBT and interpersonal approaches covering topics such as building self-esteem, keeping calm, self-talk, thinking resourcefully, problem solving, identifying and accessing support networks, considering the perspective of others and keeping the peace.

Author and Year	Geographic location (Urban/Rural)	Age group	Specific targets	Location of service delivery	Provider	Broad intervention category	Elements	Short description of intervention
**(Robjant et al., 2022)**	DRC (Rural/semi-rural)	Unknown	Other: survivors of armed conflict	Faith-based institution	Specialist MH providers (counsellors/psychotherapists)	Psychotherapeutic (ET); Social	Education; Skills; Support; Referral	NETfacts health system with two components: (a) a screen-to-treat approach to identify and refer trauma survivors with clinically relevant symptoms of PTSD to therapists who are trained in Narrative Exposure Therapy (NET) through door-to-door visits. (b) NET-facts: A community intervention that disseminated and discussed facts derived from NET treatment by sharing of the avoided facts about violence in the community, unknown by many.
**(Rochat et al., 2015)**	South Africa (Rural/semi-rural)	Adults	Parents/care givers; Affected by HIV/HIV +	Home	Lay-workers (CHW)	Psychotherapeutic (SC, other); Social	Education; Skills;Support	Amagugu: Lay-counsellor delivered home-based HIV disclosure intervention for HIV positive mothers with children aged 6-10. The lay counsellors provided training and support to the mothers to communicate with her child independently, thus enabling skills transference and parent empowerment. After disclosure stage, the mothers take their children with them to HIV clinic visits.
**(Rochat et al., 2021)**	South Africa (Mixed)	Mixed	Pregnant women; Parents/care givers; Affected by HIV/HIV +	Home	Lay-workers (CHW)	Psychotherapeutic (CBT); Social	Education; Skills; Support	Insika Yomana: The intervention, integrating psychological treatment for maternal depression and a parenting intervention to enhance early childhood development, was based on two evidence-based interventions delivered at the home: (1) Behavioral activation, (2) WHO/UNICEF Care for Child Development (CCD) (a parenting intervention).
**(Rossouw et al., 2016)**	South Africa (Urban)	Adolescents	Students	School	Specialist MH providers (psychiatric nurses)	Psychotherapeutic (ET, SM)	Education; Skills	Prolonged exposure therapy for adolescents (PE-A): A trauma-focused, exposure-based intervention, that included psychoeducation on the treatment rationale and common reactions to trauma, relaxation techniques (breathing retraining), and in vivo and imaginal exposure (emotional processing of the traumatic memory through retelling).
**(Rossouw et al., 2018)**	South Africa (Urban)	Adolescents	Students	School	Specialist MH providers (psychiatric nurses)	Psychotherapeutic (ET, SM)	Education; Skills	Prolonged exposure therapy for adolescents (PE-A): A trauma-focused, exposure-based intervention, that included psychoeducation on the treatment rationale and common reactions to trauma, relaxation techniques (breathing retraining), and in vivo and imaginal exposure (emotional processing of the traumatic memory through retelling).

Author and Year	Geographic location (Urban/Rural)	Age group	Specific targets	Location of service delivery	Provider	Broad intervention category	Elements	Short description of intervention
**(Rossouw et al., 2021)**	South Africa (Urban)	Adults	Pregnant women; Parents/care givers	Home	Lay-workers (CHW)	Lifestyle/Physical health; Social	Education; Skills; Support; Material	Incentive-based and CHW package with two components: The first component of the intervention was a monthly visit by a CHW. The content ranged from promoting clinic attendance, discussing possible danger signs, nutrition (including smoking and drinking behavior), infant-feeding options, testing for infectious diseases such as HIV and tuberculosis, and bonding with the infant. The second component was the conditional incentive of a maternal starter-kit (Thula Baba Box), valued at approximately $27.8 in 2016 prices.
**(Rossouw et al., 2022)**	South Africa (Urban)	Mixed	Students	School	Specialist MH providers (psychiatric nurses)	Psychotherapeutic (ET, SM)	Education; Skills	Prolonged exposure therapy for adolescents (PE-A): A trauma-focused, exposure-based intervention, that included psychoeducation on the treatment rationale and common reactions to trauma, relaxation techniques (breathing retraining), and in vivo and imaginal exposure (emotional processing of the traumatic memory through retelling).
**(Rotheram-Borus et al., 2015)**	South Africa (Urban)	Adults	Pregnant women; Parents/care givers	Home	Lay-workers (CHW, peers)	Psychotherapeutic (CBT, other); Lifestyle/Physical health; Social	Education; Skills; Support; Monitoring; Referral	Philani Program: In addition to access to clinic antenatal services, home visits were conducted. The CHWs aimed to reduce mother's risk of acquiring HIV; follow protocols to prevent maternal to child transmission; improve maternal and child health including tuberculosis and illness detection; reduce maternal alcohol use; improve infant and child nutrition; and foster children's growth and development.
**(Rotheram-Borus et al., 2017)**	South Africa (Rural/semi-rural)	Adults	Pregnant women; Parents/care givers	Home; Telehealth (Phone calls)	Lay-workers (CHW, peers)	Lifestyle/Physical health; Social	Education; Skills; Support; Monitoring; Referral	Accountable Care condition (AC): Philani program with additional monitoring and accountability system.

Author and Year	Geographic location (Urban/Rural)	Age group	Specific targets	Location of service delivery	Provider	Broad intervention category	Elements	Short description of intervention
**(Rotheram-Borus et al., 2019)**	South Africa (Urban)	Adults	Pregnant women; Parents/care givers	Home	Lay-workers (CHW, peers)	Psychotherapeutic; Lifestyle/Physical health; Social	Education; Skills; Support; Monitoring; Referral	Philani Program: In addition to access to clinic antenatal services, home visits were conducted. The CHWs aimed to reduce mother's risk of acquiring HIV; follow protocols to prevent maternal to child transmission; improve maternal and child health including tuberculosis and illness detection; reduce maternal alcohol use; improve infant and child nutrition; and foster children's growth and development.
**(Sakai et al., 2010)**	Rwanda (Urban)	Adoles cents	Orphans; Students; Other: genocide survivors	School; Orphanage	Specialist MH providers (counsellors/psychot herapists, social workers)	Psychotherapeutic (SC, SM, other)	Skills; Support	Thought field therapy (TFT): Self-tapping of specific acupuncture points while recalling a traumatic event or cue. Each participant was provided with an individual TFT treatment session, a progressive relaxation technique teaching lesson with supportive counselling, and a diaphragmatic breathing technique teaching lesson with supportive counselling.
**(Schmitt et al., 2022)**	DRC (Rural/semi-rural)	Mixed	Other: survivors of armed conflict	Faith-based institution	Specialist MH providers (counsellors/psychot herapists)	Psychotherapeutic (ET); Social	Education; Skills; Support; Referral	NETfacts health system with two components: (a) a screen-to-treat approach to identify and refer trauma survivors with clinically relevant symptoms of PTSD to therapists who are trained in Narrative Exposure Therapy (NET) through door-to-door visits. (b) NET-facts: A community intervention that disseminated and discussed facts derived from NET treatment by sharing of the avoided facts about violence in the community, unknown by many.
**(Schneider et al., 2020)**	Uganda (Rural/semi-rural)	Adults	Refugees; Other: War survivors	Other community places	Lay-workers (CHW)	Psychotherapeutic (ET)	Education; Skills	Narrative exposure therapy (NET): The first therapy session included psychoeducation and lifeline exercise in order to get a chronological overview of the clients' biography. Beginning with the second session, the most severe traumatic experiences were treated by means of exposure therapy following the lifeline's chronological order. The clients were encouraged to share behavioral, physical, cognitive as well as emotional reactions and sensory impressions experienced during each traumatic event in a detailed manner.

Author and Year	Geographic location (Urban/Rural)	Age group	Specific targets	Location of service delivery	Provider	Broad intervention category	Elements	Short description of intervention
**(Schouw and Mash, 2020)**	South Africa (N/A)	Adults	Unspecified	Commercial powerplant	N/A	Lifestyle/Physical health	Education; Skills; Other: opportunities for physical activities, change to healthy food	Healthy Choices at Work (HCW) program: Workplace intervention that focused on four key areas: Catering and the provision of food, opportunities for physical activity, provision of health and wellness services and managerial buy-in and participation.
**(Searle and Skinner, 2000)**	South Africa (Urban)	Adoles cents	Refugees; Students	School	Specialist MH providers (other)	Psychotherapeutic (CT, other); Social	Education; Skills; Support	School program with two components: 1) Two teacher workshops with provision of practical skills in helping refugees cope in classroom and at home. 2) Eight group sessions with children aiming at enhancing the factors that make it possible to handle flight and violent experiences.
**(Sherr et al., 2016)**	South Africa (Mixed)	Adoles cents	Affected by HIV/HIV +	School; Home; Community-based organization	N/A	Economic; Lifestyle/Physical health; Social	Education; Skills; Support; Material; Referral	Community-based organization provision (CCC): Services provided included social grants or direct income support, food/nutrition, psychosocial and emotional support, home-based care, educational support, play supervision, early childhood development support, skills building and training, medical provision, or emergency support.
**(Sherr et al., 2020)**	South Africa (Mixed)	Adoles cents	Affected by HIV/HIV +	School	N/A	Economic; Social	Education; Skills; Support; Material; Referral	Community-based organization provision (CCC): Services provided included social grants or direct income support, food/nutrition, psychosocial and emotional support, home-based care, educational support, play supervision, early childhood development support, skills building and training, medical provision, or emergency support.
**(Singla et al., 2015)**	Uganda (Rural/semi-rural)	Adults	Parents/care givers	Home; Other community places	Lay-workers (CHW)	Psychotherapeutic (CBT); Lifestyle/Physical health; Social	Education; Skills	Parenting intervention: Group intervention for mothers and fathers that addressed five messages related to childcare (play, talk, diet, hygiene, and love and respect) and maternal wellbeing (e.g., increasing father involvement). Parents also received one or two home visits by a volunteer to review the five parenting messages, discuss their enactment, resolve barriers, and make other relevant observations.

Author and Year	Geographic location (Urban/Rural)	Age group	Specific targets	Location of service delivery	Provider	Broad intervention category	Elements	Short description of intervention
**(Smith et al., 2008)**	South Africa (Urban)	Adoles cents	Students	School	Lay-workers (teachers)	Psychotherapeutic (other); Lifestyle/Physical health	Education; Skills	HealthWise South Africa: A leisure, life skills, and sexuality education intervention for eighth and ninth grade students covering social-emotional skills, the positive use of free time and attitudes, knowledge, and skills about substance use and sexual risk.
**(Sonderegger et al., 2011)**	Uganda (N/A)	Mixed	Refugees	Other community places	Lay-workers (CHW)	Psychotherapeutic (CBT); Social	Education; Skills	EMPOWER program: The program combined an evidence-based CBT framework, in conjunction with culturally sensitive and culturally relevant knowledge and activities.<br>The first phase of the program focused on enhancing emotional resiliency and the second phase on reconciliation.
**(Stansert Katzen et al., 2020)**	South Africa (Rural/semi-rural)	Mixed	Pregnant women; Parents/care givers	Home	Lay-workers (CHW)	Lifestyle/Physical health; Social	Education; Skills; Support; Referral	Philani: In addition to access to clinic antenatal services, home visits were conducted. The CHWs aimed to reduce mother's risk of acquiring HIV; follow protocols to prevent maternal to child transmission; improve maternal and child health including tuberculosis and illness detection; reduce maternal alcohol use; improve infant and child nutrition; and foster children's growth and development.
**(Stein et al., 1997)**	South Africa (Mixed)	Mixed	Unspecified	Telehealth (Phone calls)	Specialist MH providers (psychiatric nurses)	Psychotherapeutic (PE)	Education; Self-help; Support; Referral	Mental Health Information Centre: The center attempted to publicize current information about psychiatric disorders in the media and provided a telephone helpline. Callers were provided with information about specific conditions. Interventions also included the mailing of pamphlets, referrals to general practitioners and mental health care professionals, and other forms of help.
**(Stepakoff et al., 2006)**	Guinea (N/A)	Mixed	Refugees	Home; Community mental health center	Lay-workers (peers)	Psychotherapeutic (CBT, CT, ET, IPT, SC, SM); Social	Education; Skills; Support; Monitoring	Center for Victims of Torture (CVT) program with three components:<br>1) Supportive group psychotherapy to support clients in developing safe. Additionally, regular visits of vulnerable clients in their homes, individual counselling, and family counselling. 2) Social activities (e.g., games, music, sports) to promote social interactions, build resilience and identify people who might benefit from supportive psychotherapy. 3) Raise community awareness about war trauma and mental health by conducting campaigns and events.

Author and Year	Geographic location (Urban/Rural)	Age group	Specific targets	Location of service delivery	Provider	Broad intervention category	Elements	Short description of intervention
**(Takahashi et al., 2018)**	Kenya (Rural/semi-rural)	Adults	Unspecified	School; Other community places	Lay-workers (CHW)	Psychotherapeutic (MI)	Education; Skills; Support	Alcohol brief intervention (ABI) + Motivational talks (MT): ABI: Brief individual alcohol counselling through CHWs consisting of three sessions, each lasting 4-20 minutes. MT: Two talks and group discussion with a former alcohol drinker.
**(Talbot et al., 2013)**	Rwanda (N/A)	Mixed	Orphans	Day center for orphans Camps	Specialist MH providers (counsellors/psychotherapists, psychiatric nurses), Lay workers (peers)	Psychotherapeutic (CBT, CT); Economic; Lifestyle/Physical health; Social	Education; Skills; Support; Referral	Uyisenga N'Manz's (UNM) HIV prevention + mental health intervention: Incorporated HIV prevention education into an existing UNM mental health intervention. The HIV educational program consisted of group discussion, didactic lectures, and formal presentations from staff members. Topics covered within the educational program included the basics on sexuality, HIV, and strategies to prevent its transmission. UNM's existing mental health intervention consisted of adult mentorship, solidarity camps, and counselling.
**(Thomson et al., 2014)**	Rwanda (Rural/semi-rural)	Adults	Affected by HIV/HIV +	Home	Lay-workers (CHW)	Economic; Lifestyle/Physical health; Social	Education; Support; Material; Monitoring	Community-based accompaniment program: In addition to receiving clinic-based care, individuals were visited daily in their homes by a CHW who provided social support, monitored for drug side effects and adverse events, identified potential barriers to adherence, and directly observed ingestion of medications. A monthly food ration based on a family of four was provided for the first 10 months of ART, and transportation support was provided for routine clinic visits.
**(Thurman et al., 2014)**	South Africa (Rural/semi-rural)	Mixed	Parents/caregivers; Affected by HIV/HIV +	Home	Lay-workers (other)	Psychotherapeutic (SC); Social	Support	Home visits that aimed to provide emotional, informational, and tangible support to both children and their caregivers.
**(Thurman et al., 2017b)**	South Africa (Rural/semi-rural)	Adolescents	Orphans; Affected by HIV/HIV +	School; Other community places	Specialist MH providers (social workers)	Psychotherapeutic (IPT)	Education; Skills	Interpersonal psychotherapy for groups: A group intervention that focused on four interpersonal areas that trigger depressive symptoms: grief, interpersonal disputes, role transitions, and relationship deficits.

Author and Year	Geographic location (Urban/Rural)	Age group	Specific targets	Location of service delivery	Provider	Broad intervention category	Elements	Short description of intervention
**(Thurman et al., 2017a)**	South Africa (Urban)	Adolescents	Students; Other: bereaved female adolescents	School	Lay-workers (CHW)	Psychotherapeutic (CBT, SM)	Education; Skills; Self-help	Abangane: A locally derived, curriculum-based support group focused on coping with loss. Support groups activities were guided by CBT principles and indigenous games and songs, contextually relevant stories and scenarios, as well as discussions about cultural rituals and traditions surrounding death. Each participant was provided with a journal to use for recording their progress and feelings.
**(Tol et al., 2014)**	Burundi (N/A)	Adolescents	Students; Other: Affected by war	School	Lay-workers (Other)	Psychotherapeutic (CBT, CT)	Education; Skills; Referral	Classroom-based intervention (CBI): School-based care package that included universal preventive activities and provision of mental health treatments (for example, psychosocial counselling and referral to mental health specialists). The manualized intervention consisted of CBT techniques and creative expressive elements (cooperative games, structured movement, music, drama, and dance) with groups of around 15 children.
**(Tol et al., 2018)**	Uganda (Rural/semi-rural)	Adults	Refugees	School; Other community places	Lay-workers (CHW)	Psychotherapeutic (CBT)	Education; Skills; Self-help	Self-Help Plus (SH+): A guided prerecorded psychoeducational audio course of five weekly 2-h sessions delivered in workshops with 20–30 participants. An illustration-based self-help book with minimal text covered key points from audio sessions. The course was based on acceptance and commitment therapy.
**(Tol et al., 2020)**	Uganda (Rural/semi-rural)	Adults	Refugees	School; Other community places	Lay-workers (CHW)	Psychotherapeutic (CBT)	Education; Skills; Self-help	Self-Help Plus (SH+): A guided prerecorded psychoeducational audio course of five weekly 2-h sessions delivered in workshops with 20–30 participants. An illustration-based self-help book with minimal text covered key points from audio sessions. The course was based on acceptance and commitment therapy.
**(Tomlinson et al., 2016)**	South Africa (Urban)	Adults	Pregnant women; Parents/caregivers	Home	Lay-workers (CHW, peers)	Psychotherapeutic (CBT, other); Lifestyle/Physical health; Social	Education; Skills; Support; Monitoring; Referral	Philani Intervention Program (PIP): In addition to access to clinic antenatal services, home visits were conducted. The CHWs aimed to reduce mother's risk of acquiring HIV; follow protocols to prevent maternal to child transmission; improve maternal and child health including tuberculosis and illness detection; reduce maternal alcohol use; improve infant and child nutrition; and foster children’s growth and development.

Author and Year	Geographic location (Urban/Rural)	Age group	Specific targets	Location of service delivery	Provider	Broad intervention category	Elements	Short description of intervention
**(Tomlinson et al., 2021)**	South Africa (Urban)	Adults	Pregnant women; Parents/care givers	Home	Lay-workers (CHW, peers)	Social	Education; Skills; Support; Monitoring; Referral	Thula Sana: A home-based early childhood maternal–infant attachment intervention across the perinatal period. During home visits the caregivers were sensitized to their infant's individual capacities and needs and in so doing improve infant–caregiver attachment security.
**(Unterhitzenberger and Rosner, 2014)**	Rwanda (Rural/semi-rural)	Adolescents	Orphans; Students	School	N/A	Psychotherapeutic (CT)	Skills	Emotional Writing (EW): Unstructured emotional writing session about the loss of a parent to reduce adaptation problems. The EW participants were asked to write about their deepest emotions concerning their loss of an attachment figure.
**(Van't Hof et al., 2011)**	South Africa (Mixed)	Adults	Unspecified	Telehealth (Phone calls, SMS)	Specialist MH providers (counsellors/psychotherapists), Lay workers (self-care)	Psychotherapeutic (CBT)	Education; Skills; Self-help; Support	Problem solving therapy (PST) in a booklet format: Self-help PST with a booklet by reading the weekly materials. In the individual format the subjects received weekly brief telephonic support. In the group format the previous week's content was discussed and the next week's chapter was given.
**(Vancampfort et al., 2021)**	Uganda (Rural/semi-rural)	Adults	Affected by HIV/HIV +	School; Home; NGO offices Other community places	Lay-workers (CHW)	Lifestyle/Physical health	Education; Skills	Physical activity counselling intervention: This counselling for inactive patients involved (a) building sustainable knowledge that supported informed choices, (b) encouraging choice and self-initiation; (c) providing patients with a menu of options and a variety of avenues for implementing physical activity in their daily life; (d) supporting a chosen activity; (e) encouraging patients to build and explore congruence between their values and goals in life, and their lifestyle and (f) giving informational positive feedback.
**(Van der Watt et al., 2018)**	South Africa (N/A)	Adults	Unspecified	Telehealth (Phone calls)	N/A	Psychotherapeutic (other)	Support; Monitoring	Telephonic mood monitoring: After discharge from a psychiatric hospital, patients' mood fluctuations were telephonically assessed for early identification of relapse, through detection of residual depressive symptoms and providing support.

Author and Year	Geographic location (Urban/Rural)	Age group	Specific targets	Location of service delivery	Provider	Broad intervention category	Elements	Short description of intervention
**(van de Water et al., 2018)**	South Africa (Urban)	Adoles cents	Students	School	Specialist MH providers (psychiatric nurses)	Psychotherapeutic (ET)	Education; Skills	Prolonged exposure therapy for adolescents (PE-A): A trauma-focused, exposure-based intervention, that included psychoeducation on the treatment rationale and common reactions to trauma, relaxation techniques (breathing retraining), and in vivo and imaginal exposure (emotional processing of the traumatic memory through retelling).
**(Vavani et al., 2019)**	Botswana (Mixed)	Adults	Affected by HIV/HIV +	Telehealth (Phone calls)	Specialist MH providers (counsellors/psychot herapists)	Psychotherapeutic (CBT, MI, SM)	Education; Skills; Self-help; Support; Monitoring	Booklet self-help with coaching: The content of the self-help program contained the following main components: activation, relaxation, changing maladaptive cognitions, and the attainment of new personal goals. This content used a combination of psychoeducation, assignments, and exercises. In weekly telephone calls a coach discussed with the participant the progress, problems encountered, and will offer support and provide motivation to continue the program.
**(Venturo-Conerly et al., 2021)**	Kenya (Urban)	Mixed	Students	School	Lay-workers (peers)	Psychotherapeutic (other)	Education; Skills	Shamiri: A simple psychological intervention that focused on positive human attributes rather than psychopathology. Five arms: (1) the Combined Shamiri Intervention (consisting of a growth element, a gratitude element, and a value affirmation element), (2) the Growth Intervention, which encompasses content related to growth mindset and strategies for growth, (3) the Gratitude Intervention, which incorporates content to build feelings of gratitude and expression of gratitude, (4) the Values Intervention, which incorporates content to help students identify, affirm, and better act on their personal values and sense of purpose. Each arm required 4-h-long sessions spanning 4 weeks.
**(Venturo-Conerly et al., 2022b)**	Kenya (Urban)	Adoles cents	Students	School	Lay-workers (peers)	Psychotherapeutic (other)	Education; Skills	Shamiri: A simple psychological intervention that focused on positive human attributes rather than psychopathology. Single-session group Shamiri version. Participants received one of three interventions: (1) the Growth Intervention, which encompassed content related to growth mindset and strategies for growth, (2) the Gratitude Intervention, which incorporated content to build feelings of gratitude and expression of gratitude, (3) the Values Intervention, which incorporated content to help students identify, affirm, and better act on their personal values and sense of purpose.

Author and Year	Geographic location (Urban/Rural)	Age group	Specific targets	Location of service delivery	Provider	Broad intervention category	Elements	Short description of intervention
**(Venturo-Conerly et al., 2022a)**	Kenya (Urban)	Mixed	Students	School	Lay-workers (peers)	Psychotherapeutic (other)	Education; Skills	Shamiri: A simple psychological intervention that focused on positive human attributes rather than psychopathology. Five arms: (1) the Combined Shamiri Intervention (consisting of a growth element, a gratitude element, and a value affirmation element), (2) the Growth Intervention, which encompasses content related to growth mindset and strategies for growth, (3) the Gratitude Intervention, which incorporates content to build feelings of gratitude and expression of gratitude, (4) the Values Intervention, which incorporates content to help students identify, affirm, and better act on their personal values and sense of purpose. Each arm required 4-h-long sessions spanning 4 weeks
**(Verduin et al., 2014)**	Rwanda (Mixed)	Mixed	Unspecified	Other community places	Lay-workers (faith-based community member, teachers, other)	Psychotherapeutic (PE); Social	Education; Skills; Support; Other: social bonding	Community-based sociotherapy: A psychosocial community intervention aimed to enhance social bonding. Key elements were debates and the exchange of experiences and coping strategies among participants, exercises, games and mutual practical support. Trauma symptoms were addressed through psychoeducation and advice.
**(Victor-Aigbodion et al., 2023)**	Nigeria (Urban)	Adults	Students	School	Specialist MH providers (counsellors/psychotherapists)	Psychotherapeutic (CBT)	Education; Skills	Rational emotive behavior therapy (REBT): Medical students' problematic beliefs were altered using REBT approaches focusing on unreasonable notions such as demandingness, self-deprecation, awfulizing, and a low tolerance for frustration.
**(Vigna-Taglianti et al., 2021)**	Nigeria (N/A)	Adolescents	Students	School	Lay-workers (teachers)	Psychotherapeutic (other); Social	Education; Skills	Unplugged curriculum: A combined social competence and social Influence universal school curriculum. The curriculum was taught using interactive techniques and aimed to develop and enhance personal and social skills with a specific focus on normative education. Sessions on information on the consequences of tobacco, alcohol and drug use were also part of the program.

Author and Year	Geographic location (Urban/Rural)	Age group	Specific targets	Location of service delivery	Provider	Broad intervention category	Elements	Short description of intervention
**(Wasil et al., 2021)**	Kenya (Urban)	Adolescents	Students	School; Telehealth (Computer-based)	N/A	Psychotherapeutic (other)	Education; Skills; Self-help	Intervention 1: Shamiri-Digital: A simple psychological intervention that focused on positive human attributes rather than psychopathology. Online (computer) single-session intervention with different exercises addressing growth mindset, gratitude, and value affirmations. Intervention 2: The Digital-CBT intervention will consist of three modules: cognitive restructuring, problem solving and behavioral activation.
**(Wechsberg et al., 2018)**	South Africa (Urban)	Mixed	Other: Adolescent female school dropouts who use drug and engage in risky sex	Other community places	Lay-workers (CHW, peers)	Lifestyle/Physical health; Social	Education; Skills; Monitoring; Referral; Other: risk-reduction action plan	Young Women's Health CoOp (YWHC): A woman-focused, behavioral intervention that addressed AOD use, gender-based violence (GBV), and sexual risk, with the primary goal of increasing skills and knowledge to reduce HIV risk. Additionally, HIV counseling and testing (HCT). It was an adapted version for young women.
**(Wechsberg et al., 2019)**	South Africa (Urban)	Mixed	Other: women who engage in risky sex	Telehealth (Phone calls); Other community places	Specialist MH providers (female interventionists)	Lifestyle/Physical health; Social	Education; Skills; Monitoring; Referral	Women's Health CoOp Plus: A woman-focused, behavioral intervention that addressed AOD use, gender-based violence (GBV), and sexual risk, with the primary goal of increasing skills and knowledge to reduce HIV risk. Additionally, HIV counselling and testing (HCT).
**(Williams et al., 2014)**	South Africa (Urban)	Mixed	MSM	NGO offices; Outreach services (mobile van); Clinics, clubs and areas frequented by MSM	Lay-workers (CHW, peers)	Lifestyle/Physical health; Social	Education; Skills; Support; Material; Monitoring; Referral; Other: risk-reduction counselling	Community-based outreach and provision of information on HIV/AIDS, substance use, and safer sex practices. Each participant developed a personalized HIV risk reduction plan. The plans were followed up and reassessed with the participants.
**(Williams et al., 2016)**	South Africa (Urban)	Mixed	MSM; Affected by HIV/HIV +	NGO offices; Other community places LGBTQIA pride events/clubs/areas frequented	Lay-workers (CHW, peers)	Lifestyle/Physical health; Social	Education; Skills; Support; Material; Monitoring; Referral; Other: risk-reduction counselling	Community-based outreach and provision of information on HIV/AIDS, substance use, and safer sex practices. Each participant developed a personalized HIV risk reduction plan. The plans were followed up and reassessed with the participants.

Author and Year	Geographic location (Urban/Rural)	Age group	Specific targets	Location of service delivery	Provider	Broad intervention category	Elements	Short description of intervention
**(Woollett et al., 2020)**	South Africa; USA (Urban)	Adolescents	Gender-based violence; Parents/care givers	Shelter	N/A	Psychotherapeutic (CBT, CT, SM); Social	Education; Skills; Support	Trauma-informed art and play therapy: A group intervention combining trauma focused CBT (verbal therapy method) with art and play therapy (non-verbal therapy methods).
**(Wright and Chiwandira, 2016)**	Malawi (Rural/semi-rural)	Unknown	Unspecified	Other community places	Lay-workers (CHW)	Psychotherapeutic (SC); Social	Education; Skills; Support; Referral; Other: risk reduction/suicide prevention plan	Mental Health in Zomba (MHiZ) Project: Health surveillance assistants provided individual psychological support to a number of vulnerable mentally ill people in their communities, especially through psychoeducation, and by reducing risk of harm to self or others. Additionally, the health surveillance assistants provided health promotion events to people withing their communities.
**(Zanoni et al., 2022)**	South Africa (Urban)	Adolescents	Affected by HIV/HIV +	Telehealth (WhatsApp Chat groups)	N/A	Lifestyle/Physical health; Social	Education; Skills; Support	Interactive Transition Support for Adolescents Living With HIV using Social Media (InTSHA): 10 weekly moderated WhatsApp group chats with adolescents and separately with their caregivers to develop peer support and improve communication between adolescents, their caregivers, and health care providers. Topics included: (1) web-based security, (2) HIV disclosure, (3) drug and substance abuse, (4) sexual and reproductive health, (5) gender roles and sexuality, (6) stigma, (7) HIV knowledge and health care navigation, (8) ART adherence and HIV resistance, (9) healthy relationships, and (10) career planning and future goals.
**(Zoellner et al., 2021)**	Somalia (Urban)	Adults	Other: trauma survivors	Faith-based institution	Lay-workers (faith-based community members)	Psychotherapeutic (CT, ET)	Education; Skills	Islamic Trauma Healing intervention: A group intervention developed within a Somali Muslim community that integrated evidence-based trauma-focused CBT principles with cultural and religious practices.
**(Zule et al., 2014)**	South Africa (Urban)	Adults	Affected by HIV/HIV +	Other community places	Lay-workers (peers)	Lifestyle/Physical health; Social	Education; Skills	Women's Health CoOp (WHC): A woman-focused, behavioral intervention that addressed AOD use, gender-based violence (GBV), and sexual risk, with the primary goal of increasing skills and knowledge to reduce HIV risk. Additionally, HIV counseling and testing (HCT).

Note: Abbreviations used for psychotherapeutic methods CBT = cognitive behavioral treatment; CT = creative therapy; ET = exposure therapy; IPT = interpersonal therapy; MI = motivational interviewing; PE = psychoeducation only; SC = supportive counselling; SM = stress management

Table S3. Summary of included studies: Descriptions of outcomes

Author and Year	Study design (Methodology)	Mental health problem(s) addressed	Assessed quantitative outcome(s)	Quant. acceptable	Quant. feasible	Significant reduction MH/SU	Assessed qualitative outcomes	Qual. feasible	Qual. acceptable
**(Abidemi et al., 2022)**	RCT (Quantitative)	Depression	Effects			Depression			
**(Abrahams et al., 2010)**	RCT (Quantitative)	Depression	Effects			None			
**(Abrahams et al., 2022)**	Pre-post (single group) (Mixed methods)	Depression; Anxiety					Accept. (part.); Accept. (prov.); Feasibility	Unclear	No
**(Akena et al., 2021)**	Protocol of Cluster RCT (Quantitative)	Depression; Anxiety; Trauma; Alcohol use; Drug use	Engagement						
**(Akinsulore et al., 2022)**	Protocol	Depression; Anxiety	Accept. (part.); Feasibility; Effects						
**(Amoah et al., 2021)**	Cluster RCT (Quantitative)	Alcohol use	Effects			Alcohol use			
**(Angeles et al., 2019)**	Cluster RCT (Quantitative)	Depression	Effects			Depression			
**(Appiah et al., 2020)**	Controlled Trial (quasi-experimental trial) (Quantitative)	Depression	Effects; Related (Self-concept, other)			Depression			

Author and Year	Study design (Methodology)	Mental health problem(s) addressed	Assessed quantitative outcome(s)	Quant. acceptable	Quant. feasible	Significant reduction MH/SU	Assessed qualitative outcomes	Qual. feasible	Qual. acceptable
**(Are et al., 2021)**	Controlled Trial (quasi-experimental trial) (Mixed methods)	Depression	Accept. (part.); Effects; Related (Self-concept)	Yes		Depression	Accept. (part.)	Yes	
**(Atilola et al., 2022)**	Pre-post (single group) (Quantitative)	Depression; Suicidal behavior	Accept. (part.); Engagement	Yes					
**(Augsburg et al., 2022)**	Protocol of Cluster RCT (Quantitative)	Depression; Anxiety	Effects; Related (Family functioning)						
**(Bain, 2014)**	Controlled Trial (quasi-experimental trial) (Quantitative)	Depression; Anxiety	Effects; Related (Family functioning)			None			
**(Baird et al., 2013)**	Cluster RCT (Quantitative)	Depression; Anxiety	Effects			Depression; Anxiety			
**(Barnhart et al., 2020)**	Cluster RCT (Quantitative)	Depression; Anxiety	Accept. (part.); Effects; Related (Family functioning)	Yes		None			
**(Bass et al., 2013)**	Cluster RCT (Quantitative)	Depression; Anxiety; Trauma	Effects; Related (Functional impairment)			Depression; Anxiety; Trauma			
**(Baumgartner et al., 2021)**	Cluster RCT (Mixed methods)	Depression	Accept. (part.); Effects	Yes		None	Accept. (part.); Accept. (prov.); Feasibility	Yes	Unclear
**(Bella-Awusah et al., 2016)**	Cluster RCT (Quantitative)	Depression	Accept. (part.); Effects	Yes		Depression			

Author and Year	Study design (Methodology)	Mental health problem(s) addressed	Assessed quantitative outcome(s)	Quant. acceptable	Quant. feasible	Significant reduction MH/SU	Assessed qualitative outcomes	Qual. feasible	Qual. acceptable
**(Beres et al., 2021)**	RCT (Quantitative)	Alcohol use	Feasibility; Effects; Related (Qol)		Yes	None			
**(Berger et al., 2018)**	Cluster RCT (Quantitative)	Anxiety	Effects; Related (Functional impairment, behavioral)			Anxiety			
**(Berry et al., 2021)**	Controlled Trial (quasi-experimental trial) (Quantitative)	Depression	Effects; Related (Family functioning, resilience, violence)			None			
**(Betancourt et al., 2014b)**	Pre-post (single group) (Mixed methods)	Depression; Anxiety	Accept. (part.); Feasibility; Effects; Related (Functional impairment, family functioning, social support, behavioral, violence, self-concept)	Yes	Yes	Depression; Anxiety	Accept. (part.); Feasibility	Yes	Yes
**(Betancourt et al., 2014a)**	RCT (Quantitative)	Depression; Anxiety; Trauma	Effects; Related (Functional impairment, emotional, social support, behavioral)			None			
**(Betancourt et al., 2017)**	RCT (Quantitative)	Depression	Accept. (part.); Feasibility; Effects; Related (Functional impairment, family functioning)	Yes	Yes	Depression			
**(Betancourt et al., 2020)**	Cluster RCT (Quantitative)	Depression; Anxiety	Effects; Related (Violence, family functioning)			Depression; Anxiety			
**(Bliznashka et al., 2021)**	Cluster RCT (Quantitative)	Depression; Anxiety	Effects			Depression; Anxiety			
**(Boivin et al., 2013)**	Cluster RCT (Quantitative)	Depression; Anxiety	Effects; Related (Family functioning)			Depression			
**(Boivin et al., 2017)**	Cluster RCT (Mixed methods)	Depression; Anxiety	Effects; Related (Functional impairment, family functioning)			None	Related (Functional impairment)		

Author and Year	Study design (Methodology)	Mental health problem(s) addressed	Assessed quantitative outcome(s)	Quant. acceptable	Quant. feasible	Significant reduction MH/SU	Assessed qualitative outcomes	Qual. feasible	Qual. acceptable
**(Bolton et al., 2003)**	Cluster RCT (Quantitative)	Depression	Effects; Related (Functional impairment)			Depression			
**(Brown et al., 2009)**	Controlled Trial (quasi-experimental trial) (Quantitative)	Depression	Effects; Related (Social support, violence)			Depression			
**(Bryant et al., 2017)**	RCT (Quantitative)	Depression; Anxiety; Trauma	Effects; Related (Functional impairment, violence, other			Depression; Anxiety; Trauma			
**(Burgess et al., 2021)**	Pre-post (single group) (Quantitative)	Depression	Effects			Depression			
**(Carney et al., 2016)**	Pre-post (single group) (Quantitative)	Alcohol use; Drug use	Effects; Related (Alcohol related)			Drug use			
**(Carney et al., 2019)**	Cluster RCT (Mixed methods)	Alcohol use; Drug use	Effects; Related (Alcohol related)			Drug use	Accept. (part.); Related (Alcohol related)	Yes	
**(Carney et al., 2020)**	Pre-post (single group) (Mixed methods)	Alcohol use; Drug use	Feasibility; Effects; Related (Alcohol related, behavioral)		Yes	Alcohol use; Drug use	Accept. (part.)	Yes	
**(Carries et al., 2023)**	Protocol of RCT (Mixed methods)	Depression	Feasibility; Effects; Related (Qol)						
**(Cetrone et al., 2021)**	Cluster RCT (Quantitative)	Depression	Effects			Depression			

Author and Year	Study design (Methodology)	Mental health problem(s) addressed	Assessed quantitative outcome(s)	Quant. acceptable	Quant. feasible	Significant reduction MH/SU	Assessed qualitative outcomes	Qual. feasible	Qual. acceptable
**(Chaudhury et al., 2016)**	RCT (Mixed methods)	Depression; Anxiety; Alcohol use	Effects; Related (Alcohol related, violence)			Depression; Alcohol use	Accept. (part.); Effects; Related (Alcohol related, violence)	Yes	
**(Chipps et al., 2022)**	Pre-post (single group) (Quantitative)	Anxiety	Effects			Anxiety			
**(Cilliers et al., 2016)**	Cluster RCT (Quantitative)	Depression; Anxiety; Trauma	Effects; Related (Emotional, social support)			None			
**(Cluver et al., 2018)**	Cluster RCT (Mixed methods)	Depression; Suicidal behavior; Alcohol use; Drug use	Effects; Related (Family functioning, violence, social support)			Depression; Alcohol use; Drug use			
**(Coetzee et al., 2022)**	Protocol of Cluster RCT (Mixed methods)	Depression; Anxiety	Accept. (part.); Feasibility; Effects; Related (emotional, violence, self-concept, other)				Accept. (part.)		
**(Cohen et al., 2021)**	Protocol of Controlled Trial (quasi-experimental) (Mixed methods)	Depression; Anxiety	Effects; Related (functional impairment, family functioning, social support, violence)				Accept. (part.); Accept. (prov.); Feasibility		
**(Comrie-Thomson et al., 2022)**	Cluster RCT (Quantitative)	Depression; Anxiety	Effects; Related (Family functioning, other)			None			
**(Connolly and Sakai, 2011)**	RCT (Quantitative)	Depression; Anxiety; Trauma	Effects; Related (Behavioral, self-concept)			Depression; Anxiety; Trauma			
**(Constant et al., 2014)**	RCT (Quantitative)	Depression; Anxiety	Accept. (part.); Effects; Related (Other)	Yes		Depression; Anxiety			

Author and Year	Study design (Methodology)	Mental health problem(s) addressed	Assessed quantitative outcome(s)	Quant. acceptable	Quant. feasible	Significant reduction MH/SU	Assessed qualitative outcomes	Qual. feasible	Qual. acceptable
**(Cooper et al., 2002)**	Controlled Trial (quasi-experimental trial) (Quantitative)	Depression	Accept. (part.); Effects; Related (Family functioning)	Yes		None			
**(Cooper et al., 2009)**	RCT (Quantitative)	Depression	Effects; Related (Family functioning)			None			
**(Crombach and Siehl, 2018)**	Controlled Trial (quasi-experimental trial) (Quantitative)	Depression; Trauma	Accept. (part.); Feasibility; Effects; Related (Stigma)	Yes	Yes	Depression; Trauma			
**(Cubbins et al., 2012)**	Cluster RCT (Quantitative)	Alcohol use	Effects			None			
**(Dambi et al., 2022)**	Controlled Trial (quasi-experimental trial) (Mixed methods)	Depression	Accept. (part.); Feasibility; Effects; Related (Qol, functional impairment, social support)	Yes	Yes	Depression	Accept. (part.): Accept. (prov.); Feasibility	Yes	Yes
**(Dawson et al., 2016)**	RCT (Quantitative)	Depression; Anxiety; Trauma	Accept. (part.); Feasibility; Effects; Related (Functional impairment, violence, other)	Yes	Yes	None			
**(de Fouchier and Kedia, 2018)**	Pre-post (single group) (Quantitative)	Depression; Anxiety; Trauma	Effects			Depression; Anxiety; Trauma			
**(Draper et al., 2019)**	Pre-post (single group) (Mixed methods)	Depression; Anxiety	Accept. (part.); Accept. (prov.); Effects; Related (Qol, self-concept)	Yes		None	Accept. (part.); Accept. (prov.); Feasibility	Yes	No
**(Dunkle et al., 2020)**	Cluster RCT (Quantitative)	Depression; Trauma; Alcohol use	Effects; Related (Violence, family functioning, other)			Depression; Trauma			

Author and Year	Study design (Methodology)	Mental health problem(s) addressed	Assessed quantitative outcome(s)	Quant. acceptable	Quant. feasible	Significant reduction MH/SU	Assessed qualitative outcomes	Qual. feasible	Qual. acceptable
**(Duwell et al., 2013)**	Qualitative analysis of RCT	Alcohol use; Drug use					Feasibility; Effects; Related (Other)		Yes
**(Edeh et al., 2022)**	RCT (Quantitative)	Depression; Anxiety; Trauma	Effects			Depression; Anxiety; Trauma			
**(Egbe et al., 2022)**	Protocol of RCT (Mixed methods)	Alcohol use	Effects; Related (Alcohol related)						
**(Egbegi et al., 2021)**	Controlled Trial (quasi-experimental trial) (Quantitative)	Depression	Accept. (part.); Effects	Yes		Depression			
**(Eifediyi et al., 2018)**	Controlled Trial (quasi-experimental trial) (Quantitative)	Anxiety	Effects			Anxiety			
**(Ekeke et al., 2022)**	Protocol of Cluster RCT (Quantitative)	Depression; Anxiety	Effects; Related (Qol, stigma)						
**(Eller et al., 2013)**	RCT (Quantitative)	Depression	Effects			Depression			
**(Ertl et al., 2011)**	RCT (Quantitative)	Depression; Suicidal behavior; Trauma	Effects; Related (Functional impairment, emotional, self-esteem)			Trauma			
**(Ertl et al., 2021)**	Pre-post (single group) (Mixed methods)	Depression; Trauma; Alcohol use	Effects; Related (Alcohol related, functional impairment)			Depression; Alcohol use	Accept. (part.); Feasibility	Yes	Yes
**(Eseadi et al., 2022)**	RCT (Quantitative)	Depression	Effects			Depression			

**Author and Year**	**Study design (Methodology)**	**Mental health problem(s) addressed**	**Assessed quantitative outcome(s)**	**Quant. acceptable**	**Quant. feasible**	**Significant reduction MH/SU**	**Assessed qualitative outcomes**	**Qual. feasible**	**Qual. acceptable**
**(Eze et al., 2021)**	Controlled Trial (quasi-experimental trial) (Quantitative)	Alcohol use	Effects			Alcohol use			
**(Fabbri et al., 2021)**	Cluster RCT (Quantitative)	Depression	Effects; Related (Violence)			None			
**(Fernando et al., 2021)**	Pre-post (single group) (Mixed methods)	Depression; Suicidal behavior; Anxiety	Effects; Related (Functional impairment)			Depression; Suicidal behavior; Anxiety	Accept. (part.)	Yes	
**(Figge et al., 2022)**	Protocol of RCT (Mixed methods)	Depression; Anxiety; Trauma; Alcohol use; Drug use	Accept. (part.); Accept. (prov.); Effects; Related (Qol, violence)				Accept. (part.); Accept. (prov.); Feasibility		
**(Fine et al., 2021)**	Cluster RCT (Mixed methods)	Depression; Anxiety; Trauma	Effects; Related (Functional impairment, family functioning)			Depression; Anxiety	Accept. (part.); Accept. (prov.); Feasibility	Yes	Yes
**(Geffen et al., 2019)**	Pre-post (single group) (Mixed methods)	Depression; Anxiety	Effects; Related (Qol, social support)			Depression; Anxiety	Accept. (prov.); Related (Social support)	Yes	
**(Gericke et al., 2021)**	Qualitative analysis of RCT	Depression; Anxiety	Effects			Depression	Accept. (part.); Effects	Yes	
**(Giusto et al., 2020)**	Pre-post (single group) (Quantitative)	Depression; Alcohol use	Effects; Related (Family functioning)			None			
**(Giusto et al., 2021)**	Pre-post (single group) (Mixed methods)	Depression; Alcohol use	Effects				Accept. (part.); Accept. (prov.); Feasibility	Yes	Yes

Author and Year	Study design (Methodology)	Mental health problem(s) addressed	Assessed quantitative outcome(s)	Quant. acceptable	Quant. feasible	Significant reduction MH/SU	Assessed qualitative outcomes	Qual. feasible	Qual. acceptable
**(Giusto et al., 2022)**	Pre-post (single group) (Mixed methods)	Depression; Alcohol use	Feasibility		Yes		Accept. (part.); Effects	Yes	
**(Glass et al., 2017)**	RCT (Quantitative)	Depression; Anxiety; Trauma	Effects; Related (Violence)			Anxiety; Trauma			
**(Goodman et al., 2020)**	Pre-post (single group) (Quantitative)	Depression	Effects; Related (Social support)			Depression			
**(Green et al., 2016)**	Cluster RCT (Quantitative)	Depression	Effects			None			
**(Green et al., 2019)**	Cluster RCT (Quantitative)	Depression	Effects			None			
**(Green et al., 2020)**	Pre-post (single group) (Mixed methods)	Depression	Accept. (part.); Feasibility; Engagement; Effects; Related (Alcohol related)	Yes	Yes	None	Accept. (part.); Feasibility	Yes	Yes
**(Greene et al., 2019)**	Pre-post (single group) (Mixed methods)	Depression; Anxiety; Trauma	Accept. (part.); Feasibility	Unclear	Yes		Accept. (part.)	Unclear	
**(Greene et al., 2021)**	Cluster RCT (Quantitative)	Depression; Anxiety; Trauma	Accept. (part.); Feasibility; Effects; Related (Functional impairment, violence)	Yes	Yes	None			
**(Greene et al., 2022)**	Qualitative analysis of RCT	Depression; Anxiety; Trauma					Accept. (part.); Accept. (prov.); Feasibility; Effects	Yes	Unclear
**(Gupta and Zimmer, 2008)**	Pre-post (single group) (Mixed methods)	Trauma	Effects			Trauma			

Author and Year	Study design (Methodology)	Mental health problem(s) addressed	Assessed quantitative outcome(s)	Quant. acceptable	Quant. feasible	Significant reduction MH/SU	Assessed qualitative outcomes	Qual. feasible	Qual. acceptable
**(Habimana et al., 2021)**	Controlled Trial (quasi-experimental trial) (Quantitative)	Depression; Trauma	Effects			Depression; Trauma			
**(Harder et al., 2020)**	RCT (Quantitative)	Alcohol use	Effects			Alcohol use			
**(Harding et al., 2019)**	RCT (Mixed methods)	Depression; Anxiety	Accept. (part.); Feasibility; Effects	Yes	Yes	Depression; Anxiety	Accept. (part.); Feasibility	Yes	Yes
**(Hatcher et al., 2020)**	Qualitative analysis of RCT	Depression; Anxiety					Effects		
**(Hermenau et al., 2011)**	Pre-post (single group) (Quantitative)	Depression; Suicidal behavior; Trauma	Effects; Related (Violence, behavioral)			Trauma			
**(Hoss et al., 2019)**	Controlled Trial (quasi-experimental trial) (Quantitative)	Depression; Trauma	Effects; Related (Functional impairment)			None			
**(Igreja et al., 2004)**	RCT (Quantitative)	Trauma	Effects			None			
**(Im et al., 2018)**	Pre-post (single group) (Quantitative)	Trauma	Effects; Related (Emotional, social support, violence)			Trauma			
**(Ireri et al., 2019)**	Cluster RCT (Quantitative)	Anxiety	Effects			Anxiety			
**(Ismayilova et al., 2018)**	Cluster RCT (Quantitative)	Depression; Trauma	Effects; Related (Self-concept)			Depression; Trauma			

Author and Year	Study design (Methodology)	Mental health problem(s) addressed	Assessed quantitative outcome(s)	Quant. acceptable	Quant. feasible	Significant reduction MH/SU	Assessed qualitative outcomes	Qual. feasible	Qual. acceptable
**(Jani et al., 2016)**	Pre-post (single group) (Quantitative)	Anxiety	Effects; Related (Behavioral)			Depression; Anxiety			
**(Jansen et al., 2022)**	Protocol of Cluster RCT (Quantitative)	Depression; Trauma	Effects; Related (Self-concept, social support)						
**(Jewkes et al., 2008)**	Cluster RCT (Quantitative)	Depression; Alcohol use; Drug use	Effects			Depression; Alcohol use; Drug use			
**(Jibunoh and Ani, 2021)**	Controlled Trial (quasi-experimental trial) (Quantitative)	Depression; Anxiety	Effects			None			
**(Jordans et al., 2013)**	Controlled Trial (quasi-experimental trial) (Quantitative)	Depression	Accept. (part.); Effects; Related (Family functioning, behavioral)	Yes		None			
**(Kagabo et al., 2023)**	Protocol of Cluster RCT (Mixed methods)	Depression; Anxiety; Trauma	Effects; Related (Social support)						
**(Kalichman et al., 2019)**	RCT (Quantitative)	Alcohol use	Related (Alcohol related, stigma)						
**(Karnell et al., 2006)**	RCT (Quantitative)	Alcohol use	Effects; Related (Alcohol related)			None			
**(Khuzwayo et al., 2020)**	Cluster RCT (Quantitative)	Alcohol use; Drug use	Effects; Related (Other)			None			
**(Kumakech et al., 2009)**	Cluster RCT (Quantitative)	Depression; Anxiety	Effects; Related (Self-concept)			Depression; Anxiety			

Author and Year	Study design (Methodology)	Mental health problem(s) addressed	Assessed quantitative outcome(s)	Quant. acceptable	Quant. feasible	Significant reduction MH/SU	Assessed qualitative outcomes	Qual. feasible	Qual. acceptable
**(Kuo et al., 2020)**	RCT (Mixed methods)	Depression	Accept. (part.); Feasibility; Effects; Related (Resilience)	Yes	Yes	None	Accept. (part.)	Unclear	
**(Lachman et al., 2020)**	Cluster RCT (Quantitative)	Depression	Effects; Related (Behavioral, family functioning, violence)			None			
**(Lasebikan et al., 2017)**	Pre-post (single group) (Quantitative)	Alcohol use	Effects			Alcohol use			
**(Lawrence and Falaye, 2020)**	Controlled Trial (quasi-experimental trial) (Quantitative)	Depression; Suicidal behavior; Anxiety	Effects; Related (Other)						
**(L'Engle et al., 2014)**	RCT (Quantitative)	Alcohol use	Effects; Related (Violence)			Alcohol use			
**(le Roux et al., 2020)**	Controlled Trial (quasi-experimental trial) (Quantitative)	Depression	Effects			Depression			
**(Lloyd-Sherlock et al., 2019)**	Pre-post (single group) (Mixed methods)	Depression	Effects			None	Accept. (part.)	Yes	
**(Logie et al., 2021)**	Protocol of Cluster RCT (Quantitative)	Depression	Effects; Related (Stigma, other)						
**(Logie et al., 2022)**	Protocol of RCT (Quantitative)	Depression	Engagement; Effects; Related (Functional impairment, stigma)						

Author and Year	Study design (Methodology)	Mental health problem(s) addressed	Assessed quantitative outcome(s)	Quant. acceptable	Quant. feasible	Significant reduction MH/SU	Assessed qualitative outcomes	Qual. feasible	Qual. acceptable
**(Lund et al., 2013)**	Pre-post (single group) (Quantitative)	Depression; Anxiety	Feasibility; Effects; Related (Functional impairment, social support, qol)		Yes	Depression; Anxiety			
**(Lund et al., 2014)**	Protocol of RCT (Mixed methods)	Depression; Alcohol use; Drug use	Effects; Related (Functional impairment, social support)						
**(Marfo and Owusu-Daaku, 2016)**	Pre-post (single group) (Mixed methods)	Alcohol use	Effects			Alcohol use	Accept. (part.); Accept. (prov.); Feasibility	Yes	Yes
**(Masulani-Mwale et al., 2019)**	Qualitative analysis of Pre-post (single group)	Depression; Suicidal behavior; Anxiety					Accept. (part.); Feasibility	Yes	Yes
**(Matsuba et al., 2020)**	Pre-post (single group) (Mixed methods)	Depression	Effects; Related (Emotional, behavioral)			Depression	Feasibility		Yes
**(McLea and Mayers, 2017)**	Pre-post (single group) (Mixed methods)	Depression; Anxiety	Effects			None	Accept. (part.); Related (Other)	Yes	
**(McMullen and McMullen, 2018)**	Controlled Trial (quasi-experimental trial) (Quantitative)	Depression; Anxiety	Effects; Related (Behavioral, self-concept, social support)			Depression; Anxiety			
**(Moffett et al., 2022)**	Protocol of RCT (Mixed methods)	Depression; Anxiety; Alcohol use; Drug use	Accept. (part.); Accept. (prov.); Effects; Related (Resilience); Feasibility				Accept. (part.)		
**(Monnapula-Mazabane and Petersen, 2022)**	Qualitative analysis of Pre-post (single group)	Depression					Accept. (part.); Related (Family functioning, stigma)	Yes	

Author and Year	Study design (Methodology)	Mental health problem(s) addressed	Assessed quantitative outcome(s)	Quant. acceptable	Quant. feasible	Significant reduction MH/SU	Assessed qualitative outcomes	Qual. feasible	Qual. acceptable
**(Morojele et al., 2014)**	Qualitative analysis of case	Alcohol use					Accept. (part.); Accept. (prov.); Feasibility	Yes	Yes
**(Mpande et al., 2013)**	Controlled Trial (quasi-experimental trial) (Quantitative)	Depression; Anxiety	Effects; Related (Other)			Depression; Anxiety			
**(Muriungi and Ndetei, 2013)**	Controlled Trial (quasi-experimental trial) (Quantitative)	Depression; Suicidal behavior; Anxiety; Alcohol use; Drug use	Effects			Depression; Anxiety; Drug use			
**(Murray et al., 2018)**	Pre-post (single group) (Mixed methods)	Depression; Anxiety; Trauma	Effects			Depression; Anxiety; Trauma	Accept. (part.); Accept. (prov.); Feasibility; Effects	Yes	Yes
**(Musyoka et al., 2023)**	Controlled Trial (quasi-experimental trial) (Mixed methods)	Alcohol use; Drug use	Accept. (prov.); Feasibility	Yes	Yes		Accept. (prov.)	Yes	
**(Mutamba et al., 2018)**	Controlled Trial (quasi-experimental trial) (Quantitative)	Depression; Suicidal behavior; Anxiety; Trauma; Alcohol use	Effects; Related (Functional impairment, social support, stigma)			Depression			
**(Namasaba et al., 2022)**	Cluster RCT (Quantitative)	Depression	Effects			Depression			
**(Namy et al., 2021)**	Pre-post (single group) (Mixed methods)	Depression	Effects; Related (Emotional)			Depression	Accept. (part.); Accept. (prov.); Feasibility	Yes	Yes

Author and Year	Study design (Methodology)	Mental health problem(s) addressed	Assessed quantitative outcome(s)	Quant. acceptable	Quant. feasible	Significant reduction MH/SU	Assessed qualitative outcomes	Qual. feasible	Qual. acceptable
**(Negash et al., 2021)**	Pre-post (single group) (Mixed methods)	Depression; Anxiety	Accept. (part.); Feasibility; Effects; Related (Functional impairment)	Yes	Yes	Depression; Anxiety	Accept. (part.)	Yes	
**(Nwobi et al., 2018)**	RCT (Quantitative)	Depression; Anxiety	Effects; Related (Other)			Depression; Anxiety			
**(Nyongesa et al., 2022)**	Controlled Trial (quasi-experimental trial) (Mixed methods)	Depression; Anxiety	Accept. (part.); Feasibility; Effects; Related (Social support, qol)	Yes	Yes	Depression; Anxiety	Accept. (part.); Feasibility	Yes	Yes
**(O'Callaghan et al., 2014)**	RCT (Mixed methods)	Depression; Anxiety; Trauma	Accept. (part.); Effects; Related (Behavioral)	Yes		Depression; Anxiety; Trauma			
**(Odenwald et al., 2012)**	Controlled Trial (quasi-experimental trial) (Quantitative)	Depression; Anxiety; Drug use	Accept. (part.); Feasibility; Engagement; Effects; Related (Functional impairment)	Yes	Yes	Drug use			
**(O'Donnell et al., 2014)**	Pre-post (single group) (Quantitative)	Depression; Trauma	Accept. (part.); Feasibility; Effects	Yes	Yes	Depression; Trauma			
**(Ofoegbu et al., 2020)**	RCT (Quantitative)	Depression	Effects			Depression			
**(Ogum Alangea et al., 2020)**	Cluster RCT (Quantitative)	Depression	Effects; Related (Behavioral, violence, other)			Depression			
**(Okube et al., 2022)**	RCT (Quantitative)	Alcohol use	Effects			Alcohol use			

Author and Year	Study design (Methodology)	Mental health problem(s) addressed	Assessed quantitative outcome(s)	Quant. acceptable	Quant. feasible	Significant reduction MH/SU	Assessed qualitative outcomes	Qual. feasible	Qual. acceptable
**(Olowokere and Okanlawon, 2014)**	Controlled Trial (quasi-experimental trial) (Quantitative)	Depression; Anxiety	Effects; Related (Self-concept, Resilience)			None			
**(Olowokere and Okanlawon, 2018) 12/12/2023 19:31:00**	Comparative prospective study (Quantitative)	Depression; Anxiety	Effects; Related (Self-concept, social support)			Anxiety			
**(Onyechi et al., 2016)**	Controlled Trial (quasi-experimental trial) (Quantitative)	Depression; Anxiety	Effects			Depression; Anxiety			
**(Onyut et al., 2004)**	RCT (Quantitative)	Depression; Trauma	Feasibility; Effects; Related (Functional impairment)		Yes	Trauma			
**(Osafo and Andoh-Arthur, 2020)**	Qualitative analysis of case	Suicidal behavior				None	Accept. (part.); Related (Stigma)	Unclear	
**(Osborn et al., 2020a)**	RCT (Quantitative)	Depression; Anxiety	Accept. (part.); Feasibility; Effects; Related (Social support)	Yes	Yes	Depression			
**(Osborn et al., 2020b)**	RCT (Mixed methods)	Depression; Anxiety	Accept. (part.); Feasibility; Effects; Related (Emotional)	Unclear	Unclear	Depression; Anxiety	Accept. (part.); Feasibility	Unclear	Unclear
**(Osborn et al., 2021)**	RCT (Quantitative)	Depression; Anxiety	Accept. (part.); Feasibility; Effects	Yes	Yes	Depression; Anxiety			
**(Osiki and Busari, 2002)**	Controlled Trial (quasi-experimental trial) (Quantitative)	Anxiety	Effects			Anxiety			

Author and Year	Study design (Methodology)	Mental health problem(s) addressed	Assessed quantitative outcome(s)	Quant. acceptable	Quant. feasible	Significant reduction MH/SU	Assessed qualitative outcomes	Qual. feasible	Qual. acceptable
**(Pandya, 2018)**	Pre-post (single group) (Quantitative)	Depression	Effects; Related (Resilience, self-concept, qol)			Depression			
**(Parry et al., 2017)**	Pre-post (single group) (Quantitative)	Alcohol use; Drug use	Effects; Related (Alcohol related)			Alcohol use			
**(Patel and Ross, 2020)**	Controlled Trial (quasi-experimental trial) (Mixed methods)	Depression	Effects			None	Related (Family functioning, social support)		
**(Pengpid et al., 2013)**	RCT (Quantitative)	Depression; Trauma; Alcohol use; Drug use	Effects			Alcohol use			
**(Pengpid et al., 2014)**	Protocol of Cluster RCT (Quantitative)	Alcohol use	Effects; Related (Other)						
**(Pettifor et al., 2018)**	Cluster RCT (Quantitative)	Alcohol use	Effects; Related (Violence, other)			None			
**(Prencipe et al., 2021)**	Cluster RCT (Quantitative)	Depression	Effects			Depression			
**(Prinsloo et al., 2016)**	Pre-post (single group) (Quantitative)	Depression	Effects			Depression			
**(Puffer et al., 2020)**	Pre-post (single group) (Mixed methods)	Depression; Anxiety; Alcohol use	Effects; Related (Alcohol related, family functioning, violence)			Depression; Anxiety; Alcohol use	Effects; Related (Family functioning)		
**(Rabie et al., 2022)**	Cluster RCT (Quantitative)	Depression; Alcohol use; Drug use	Effects; Related (Violence)			None			

Author and Year	Study design (Methodology)	Mental health problem(s) addressed	Assessed quantitative outcome(s)	Quant. acceptable	Quant. feasible	Significant reduction MH/SU	Assessed qualitative outcomes	Qual. feasible	Qual. acceptable
**(Ramdhonee-Dowlot et al., 2021)**	RCT (Quantitative)	Depression; Anxiety	Effects; Related (Emotional, self-concept)			Depression; Anxiety			
**(Reeler et al., 2009)**	Pre-post (single group) (Mixed methods)	Depression; Anxiety	Accept. (part.); Effects	Yes		Depression; Anxiety	Accept. (part.)	Yes	
**(Richards et al., 2014)**	RCT (Quantitative)	Depression; Anxiety	Effects			None			
**(Rivet-Duval et al., 2011)**	RCT (Quantitative)	Depression	Accept. (part.); Effects; Related (Self-concept, other)	Yes		Depression			
**(Robjant et al., 2022)**	RCT (Mixed methods)	Depression; Trauma	Engagement; Effects; Related (Stigma)			Depression	Effects		
**(Rochat et al., 2015)**	Pre-post (single group) (Quantitative)	Depression; Anxiety	Effects; Related (Behavioral, other)			Depression; Anxiety			
**(Rochat et al., 2021)**	Protocol of RCT (Mixed methods)	Depression; Anxiety	Effects; Related (Other)				Accept. (part.); Accept. (prov.)		
**(Rossouw et al., 2016)**	RCT (Quantitative)	Depression; Trauma	Feasibility; Effects		Yes	Depression; Trauma			
**(Rossouw et al., 2018)**	RCT (Mixed methods)	Depression; Trauma	Effects; Related (Behavioral, emotional, functional impairment, self-esteem)			Depression; Trauma			
**(Rossouw et al., 2021)**	RCT (Quantitative)	Depression; Trauma	Effects; Related (Functional impairment)			Depression; Trauma			
**(Rossouw et al., 2022)**	RCT (Quantitative)	Depression	Effects			Depression			

Author and Year	Study design (Methodology)	Mental health problem(s) addressed	Assessed quantitative outcome(s)	Quant. acceptable	Quant. feasible	Significant reduction MH/SU	Assessed qualitative outcomes	Qual. feasible	Qual. acceptable
**(Rotheram-Borus et al., 2015)**	Cluster RCT (Quantitative)	Depression; Anxiety; Alcohol use	Effects; Related (Violence)			Depression			
**(Rotheram-Borus et al., 2017)**	Protocol of RCT (Quantitative)	Depression; Alcohol use	Effects						
**(Rotheram-Borus et al., 2019)**	Cluster RCT (Quantitative)	Alcohol use	Effects; Related (Behavioral)			Alcohol use			
**(Sakai et al., 2010)**	Pre-post (single group) (Quantitative)	Trauma	Effects; Related (Other)			Trauma			
**(Schmitt et al., 2022)**	Pre-post (single group) (Quantitative)	Depression; Trauma	Feasibility; Effects		Yes	Trauma			
**(Schneider et al., 2020)**	Pre-post (single group) (Quantitative)	Trauma	Effects			Trauma			
**(Schouw and Mash, 2020)**	Pre-post (single group) (Quantitative)	Alcohol use	Feasibility; Effects		Yes	Alcohol use			
**(Searle and Skinner, 2000)**	Qualitative analysis of case	Anxiety	Effects; Related (Behavioral)				Effects; Related (Behavioral)		
**(Sherr et al., 2016)**	Comparison of two longitudinal studies (Quantitative)	Depression; Suicidal behavior; Trauma	Effects; Related (Behavioral, stigma, violence)			Depression; Suicidal behavior			

Author and Year	Study design (Methodology)	Mental health problem(s) addressed	Assessed quantitative outcome(s)	Quant. acceptable	Quant. feasible	Significant reduction MH/SU	Assessed qualitative outcomes	Qual. feasible	Qual. acceptable
**(Sherr et al., 2020)**	Comparison of two longitudinal studies (Quantitative)	Depression	Effects			Depression			
**(Singla et al., 2015)**	Cluster RCT (Quantitative)	Depression	Effects; Related (Family functioning, other)			Depression			
**(Smith et al., 2008)**	Controlled Trial (quasi-experimental trial) (Quantitative)	Alcohol use; Drug use	Effects; Related (Behavioral)			Alcohol use			
**(Sonderegger et al., 2011)**	Controlled Trial (quasi-experimental trial) (Quantitative)	Depression; Anxiety	Effects; Related (Behavioral)			Depression; Anxiety			
**(Stansert Katzen et al., 2020)**	Controlled Trial (quasi-experimental trial) (Quantitative)	Depression	Effects			Depression			
**(Stein et al., 1997)**	Qualitative analysis of case	Depression; Anxiety					Accept. (part.)	Yes	
**(Stepakoff et al., 2006)**	Pre-post (single group) (Quantitative)	Depression; Anxiety; Trauma	Effects; Related (Functional impairment, social support)			Trauma			
**(Takahashi et al., 2018)**	Controlled Trial (quasi-experimental trial) (Quantitative)	Alcohol use	Effects			Alcohol use			

Author and Year	Study design (Methodology)	Mental health problem(s) addressed	Assessed quantitative outcome(s)	Quant. acceptable	Quant. feasible	Significant reduction MH/SU	Assessed qualitative outcomes	Qual. feasible	Qual. acceptable
**(Talbot et al., 2013)**	Pre-post (single group) (Quantitative)	Trauma; Alcohol use	Effects; Related (Social support)			Trauma			
**(Thomson et al., 2014)**	Controlled Trial (quasi-experimental trial) (Quantitative)	Depression	Effects; Related (Social support, qol)			Depression			
**(Thurman et al., 2014)**	Controlled Trial (quasi-experimental trial) (Quantitative)	Depression	Effects; Related (Behavioral, Family functioning)			None			
**(Thurman et al., 2017b)**	Cluster RCT (Quantitative)	Depression	Effects			None			
**(Thurman et al., 2017a)**	RCT (Quantitative)	Depression	Effects; Related (Behavioral, emotional, family functioning, social support)			Depression			
**(Tol et al., 2014)**	Cluster RCT (Quantitative)	Depression; Anxiety; Trauma	Effects; Related (Emotional, functional impairment)			None			
**(Tol et al., 2018)**	Pre-post (single group) (Mixed methods)	Depression; Anxiety	Effects; Related (Functional impairment, resilience)			Depression	Accept. (part.); Accept. (prov.); Feasibility	Yes	Yes
**(Tol et al., 2020)**	Cluster RCT (Quantitative)	Depression; Anxiety; Trauma	Effects; Related (Emotional, functional impairment, resilience, other)			Depression; Anxiety; Trauma			
**(Tomlinson et al., 2016)**	Cluster RCT (Quantitative)	Depression; Alcohol use	Effects; Related (Family functioning)			Depression			
**(Tomlinson et al., 2021)**	RCT (Quantitative)	Depression; Anxiety	Effects			Depression			

Author and Year	Study design (Methodology)	Mental health problem(s) addressed	Assessed quantitative outcome(s)	Quant. acceptable	Quant. feasible	Significant reduction MH/SU	Assessed qualitative outcomes	Qual. feasible	Qual. acceptable
**(Unterhitzenberger and Rosner, 2014)**	RCT (Quantitative)	Depression	Effects; Related (Emotional)			None			
**(Van't Hof et al., 2011)**	Pre-post (single group) (Quantitative)	Depression; Anxiety	Accept. (part.); Feasibility; Effects	Yes (group based)	Yes (group based)	Depression; Anxiety			
**(Vancampfort et al., 2021)**	Pre-post (single group)	Depression; Anxiety	Effects; Related (Functional impairment, other)			Depression; Anxiety			
**(Van der Watt et al., 2018)**	Qualitative analysis of RCT	Depression; Anxiety					Accept. (part.); Feasibility; Effects	Unclear	Unclear
**(van de Water et al., 2018)**	Qualitative analysis of RCT	Trauma					Accept. (part.)	Unclear	
**(Vavani et al., 2019)**	Protocol of RCT (Quantitative)	Depression; Anxiety; Alcohol use	Effects; Related (Self-concept, social support)				Accept. (part.)		
**(Venturo-Conerly et al., 2021)**	Protocol of RCT (Mixed methods)	Depression; Anxiety	Accept. (part.); Effects; Related (Social support)				Accept. (part.)		
**(Venturo-Conerly et al., 2022b)**	Cluster RCT (Quantitative)	Depression; Anxiety	Accept. (part.); Effects	Yes		Anxiety			
**(Venturo-Conerly et al., 2022a)**	Protocol of Follow-up XX (Quantitative)	Depression; Anxiety; Alcohol use	Effects; Related (Social support, violence)						

Author and Year	Study design (Methodology)	Mental health problem(s) addressed	Assessed quantitative outcome(s)	Quant. acceptable	Quant. feasible	Significant reduction MH/SU	Assessed qualitative outcomes	Qual. feasible	Qual. acceptable
**(Verduin et al., 2014)**	Controlled Trial (quasi-experimental trial) (Quantitative)	Depression; Anxiety	Effects; Related (Social support)			Depression; Anxiety			
**(Victor-Aigbodion et al., 2023)**	RCT (Quantitative)	Depression	Effects			Depression			
**(Vigna-Taglianti et al., 2021)**	Cluster RCT (Quantitative)	Alcohol use; Drug use	Effects			Alcohol use			
**(Wasil et al., 2021)**	Protocol of RCT (Quantitative)	Depression; Anxiety	Accept. (part.); Effects						
**(Wechsberg et al., 2018)**	Protocol of Cluster RCT (Quantitative)	Alcohol use; Drug use	Effects; Related (Alcohol related)						
**(Wechsberg et al., 2019)**	Cluster RCT (Quantitative)	Alcohol use; Drug use	Effects; Related (Alcohol related, violence)			Alcohol use			
**(Williams et al., 2014)**	Pre-post (single group) (Quantitative)	Alcohol use; Drug use	Effects; Related (Alcohol related)						
**(Williams et al., 2016)**	Pre-post (single group) (Quantitative)	Alcohol use; Drug use	Effects; Related (Alcohol related)			Drug use			
**(Woollett et al., 2020)**	Pre-post (single group) (Mixed methods)	Depression; Trauma	Effects			Depression	Related (Behavioral, emotional)		
**(Wright and Chiwandira, 2016)**	Pre-post (single group) (Quantitative)	Depression; Suicidal behavior; Anxiety; Alcohol use; Drug use	Engagement						

Author and Year	Study design (Methodology)	Mental health problem(s) addressed	Assessed quantitative outcome(s)	Quant. acceptable	Quant. feasible	Significant reduction MH/SU	Assessed qualitative outcomes	Qual. feasible	Qual. acceptable
**(Zanoni et al., 2022)**	Protocol of RCT (Quantitative)	Depression	Accept. (part.); Feasibility; Effects; Related (Self-concept, stigma)						
**(Zoellner et al., 2021)**	Pre-post (single group) (Mixed methods)	Depression; Trauma	Accept. (part.); Feasibility; Effects	Yes	Yes	Depression; Trauma	Accept. (part.)	Yes	
**(Zule et al., 2014)**	RCT (Quantitative)	Alcohol use; Drug use	Effects			Alcohol use			

Note: Abbreviations used for quantitative and qualitative assessed outcomes accept. = acceptability, part. = participants, prov. = providers, related = related psychosocial outcomes, qol = quality of life, effects = effects on symptoms/diagnosis of mental health/substance use problem

References Tables S2 & S3

Abidemi, O.O., Oluwafunmilola, O.A., Babalola, B.T., 2022. Effects of Care Givers Counselling on Depression among People Living With HIV/AIDs. J Biostat Epidemiol 8, 102–114.

Abrahams, N., Jewkes, R., Lombard, C., Mathews, S., Campbell, J., Meel, B., 2010. Impact of telephonic psycho-social support on adherence to post-exposure prophylaxis (PEP) after rape. AIDS Care - Psychol. Socio-Med. Asp. AIDSHIV 22, 1173–1181. https://doi.org/10.1080/09540121003692185

Abrahams, Z., Jacobs, Y., Mohlamonyane, M., Boisits, S., Schneider, M., Honikman, S., Seward, N., Lund, C., 2022. Implementation outcomes of a health systems strengthening intervention for perinatal women with common mental disorders and experiences of domestic violence in South Africa: Pilot feasibility and acceptability study. BMC Health Serv. Res. 22, 641. https://doi.org/10.1186/s12913-022-08050-x

Akena, D., Kiguba, R., Muhwezi, W.W., Kwesiga, B., Kigozi, G., Nakasujja, N., Lukwata, H., 2021. The effectiveness of a psycho-education intervention on mental health literacy in communities affected by the COVID-19 pandemic—a cluster randomized trial of 24 villages in central Uganda—a research protocol. Trials 22. https://doi.org/10.1186/s13063-021-05391-6

Akinsulore, A., Aloba, O., Oginni, O., Oloniniyi, I., Ibigbami, O., Seun-Fadipe, C.T., Opakunle, T., Owojuyigbe, A.M., Olibamoyo, O., Mapayi, B., Okorie, V.O., Adewuya, A.O., 2022. Developing an mHealth Intervention to Reduce COVID-19-Associated Psychological Distress Among Health Care Workers in Nigeria: Protocol for a Design and Feasibility Study. JMIR Res Protoc 11, e36174. https://doi.org/10.2196/36174

Amoah, J., Said, S., Rampal, L., Manaf, R., Ibrahim, N., Owusu-Agyei, S., Asante, K.P., 2021. Effects of a school-based intervention to reduce cardiovascular disease risk factors among secondary school students: A cluster-randomized, controlled trial. PLoS ONE 16. https://doi.org/10.1371/journal.pone.0259581

Angeles, G., de Hoop, J., Handa, S., Kilburn, K., Milazzo, A., Peterman, A., Abdoulayi, S., Barrington, C., Brugh, K., Molotsky, A., Otchere, F., Zietz, S., Mvula, P., Tsoka, M., Palermo, T., 2019. Government of Malawi's unconditional cash transfer improves youth mental health. Soc. Sci. Med. 225, 108–119. https://doi.org/10.1016/j.socscimed.2019.01.037

Appiah, R., Wilson-Fadiji, A., Schutte, L., Wissing, M.P., 2020. Effects of a Community-Based Multicomponent Positive Psychology Intervention on Mental Health of Rural Adults in Ghana. Appl. Psychol. Health Well-Being 12, 828–862. https://doi.org/10.1111/aphw.12212

Are, A., Olisah, V., Bella-Awusah, T., Ani, C., 2021. Controlled clinical trial of teacher-delivered Cognitive Behavioural Therapy (CBT) for adolescents with clinically diagnosed depressive disorder in Nigeria. Int. J. Ment. Health. https://doi.org/10.1080/00207411.2021.1891361

Atilola, O., Ayinde, O., Obialo, F.-K., Adeyemo, S.O., Adegbaju, D., Anthony, R., 2022. Towards school-based mental health programs in Nigeria: the immediate impact of a depression-literacy program among school-going adolescents and their teachers. Child Adolesc. Psychiatry Ment. Health 16, 70. https://doi.org/10.1186/s13034-022-00503-9

Augsburg, B., Attanasio, O.P., Dreibelbis, R., Nketiah-Amponsah, E., Phimister, A., Wolf, S., Krutikova, S., 2022. Lively Minds: improving health and development through play-a randomised controlled trial evaluation of a comprehensive ECCE programme at scale in Ghana. BMJ Open 12. https://doi.org/10.1136/bmjopen-2022-061571

Bain, K., 2014. “New beginnings” in South African shelters for the homeless: Piloting of a group psychotherapy intervention for high-risk mother-infant dyads. Infant Ment. Health J. 35, 591–603. https://doi.org/10.1002/imhj.21457

Baird, S., de Hoop, J., Özler, B., 2013. Income shocks and adolescent mental health. J. Hum. Resour. 48, 370–403. https://doi.org/10.3368/jhr.48.2.370

Barnhart, D.A., Farrar, J., Murray, S.M., Brennan, R.T., Antonaccio, C.M., Sezibera, V., Ingabire, C., Godfroid, K., Bazubagira, S., Uwimana, O., Kamurase, A., Wilson, B., Rawlings, L.B., Yousafzai, A., Betancourt, T.S., 143660652. Language: English. Entry Date: 20200611. Revision Date: 20210701. Publication Type: Article, 2020. Lay-worker Delivered Home Visiting Promotes Early Childhood Development and Reduces Violence in Rwanda: A Randomized Pilot. J. Child Fam. Stud. 29, 1804–1817. https://doi.org/10.1007/s10826-020-01709-1

Bass, J.K., Annan, J., Murray, S.M., Kaysen, D., Griffiths, S., Cetinoglu, T., Wachter, K., Murray, L.K., Bolton, P.A., 2013. Controlled trial of psychotherapy for congolese survivors of sexual violence. N. Engl. J. Med. 368, 2182–2191. https://doi.org/10.1056/NEJMoa1211853

Baumgartner, J.N., Ali, M., Gallis, J.A., Lillie, M., Owusu, R., Abubakr-Bibilazu, S., Adam, H., Aborigo, R., McEwan, E., Zhou, Y., Kim, E.T., Mackness, J., Williams, J.K.A., Hembling, J., 2021. Effect of a lay counselor-delivered integrated maternal mental health and early childhood development group-based intervention in Northern Ghana: A cluster-randomized controlled trial. Glob. Ment. Health. https://doi.org/10.1017/gmh.2021.15

Bella-Awusah, T., Ani, C., Ajuwon, A., Omigbodun, O., 2016. Effectiveness of brief school-based, group cognitive behavioural therapy for depressed adolescents in south west Nigeria. Child Adolesc. Ment. Health 21, 44–50. https://doi.org/10.1111/camh.12104

Beres, L.K., Mbabali, I., Anok, A., Katabalwa, C., Mulamba, J., Thomas, A.G., Bugos, E., Nakigozi, G., Grabowski, M.K., Chang, L.W., 34283027, 2021. Mobile Ecological Momentary Assessment and Intervention and Health Behavior Change Among Adults in Rakai, Uganda: Pilot Randomized Controlled Trial. JMIR Form. Res. 5, e22693. https://doi.org/10.2196/22693

Berger, R., Benatov, J., Cuadros, R., VanNattan, J., Gelkopf, M., 2018. Enhancing resiliency and promoting prosocial behavior among Tanzanian primary-school students: A school-based intervention. Transcult. Psychiatry 55, 821–845. https://doi.org/10.1177/1363461518793749

Berry, L., Mathews, S., Reis, R., Crone, M., 2021. Mental health effects on adolescent parents of young children: Reflections on outcomes of an adolescent parenting programme in south africa. Vulnerable Child. Youth Stud. No Pagination Specified. https://doi.org/10.1080/17450128.2021.1954737

Betancourt, T.S., Jensen, S.K.G., Barnhart, D.A., Brennan, R.T., Murray, S.M., Yousafzai, A.K., Farrar, J., Godfroid, K., Bazubagira, S.M., Rawlings, L.B., Wilson, B., Sezibera, V., Kamurase, A., 2020. Promoting parent-child relationships and preventing violence via home-visiting: a pre-post cluster randomised trial among Rwandan families linked to social

protection programmes. BMC Public Health 20, 621. https://doi.org/10.1186/s12889-020-08693-7

Betancourt, T.S., McBain, R., Newnham, E.A., Akinsulure-Smith, A.M., Brennan, R.T., Weisz, J.R., Hansen, N.B., 2014a. A behavioral intervention for war-affected youth in Sierra Leone: A randomized controlled trial. J. Am. Acad. Child Adolesc. Psychiatry 53, 1288–1297. https://doi.org/10.1016/j.jaac.2014.09.011

Betancourt, T.S., Ng, L.C., Kirk, C.M., Brennan, R.T., Beardslee, W.R., Stulac, S., Mushashi, C., Nduwimana, E., Mukunzi, S., Nyirandagijimana, B., Kalisa, G., Rwabukwisi, C.F., Sezibera, V., 2017. Family-based promotion of mental health in children affected by HIV: a pilot randomized controlled trial. J. Child Psychol. Psychiatry 58, 922–930. https://doi.org/10.1111/jcpp.12729

Betancourt, T.S., Ng, L.C., Kirk, C.M., Munyanah, M., Mushashi, C., Ingabire, C., Teta, S., Beardslee, W.R., Brennan, R.T., Zahn, I., Stulac, S., Cyamatare, F.R., Sezibera, V., 2014b. Family-based prevention of mental health problems in children affected by HIV and AIDS: An open trial. AIDS 28, S359–S368. https://doi.org/10.1097/QAD.0000000000000336

Bliznashka, L., Yousafzai, A.K., Asheri, G., Masanja, H., Sudfeld, C.R., 2021. Effects of a community health worker delivered intervention on maternal depressive symptoms in rural Tanzania. Health Policy Plan. 36, 473–483. https://doi.org/10.1093/heapol/czaa170

Boivin, M.J., Bangirana, P., Nakasujja, N., Page, C.F., Shohet, C., Givon, D., Bass, J.K., Opoka, R.O., Klein, P.S., 2013. A year-long caregiver training program improves cognition in preschool ugandan children with human immunodeficiency virus. J. Pediatr. 163, 1409-1416.e5. https://doi.org/10.1016/j.jpeds.2013.06.055

Boivin, M.J., Nakasujja, N., Familiar-Lopez, I., Murray, S.M., Sikorskii, A., Awadu, J., Shohet, C., Givon, D., Ruiseñor-Escudero, H., Schut, E.E., Opoka, R.O., Bass, J.K., 2017. Effect of Caregiver Training on the Neurodevelopment of HIV-Exposed Uninfected Children and Caregiver Mental Health: A Ugandan Cluster-Randomized Controlled Trial. J. Dev. Behav. Pediatr. JDBP 38, 753–764. https://doi.org/10.1097/DBP.0000000000000510

Bolton, P., Bass, J., Neugebauer, R., Verdeli, H., Clougherty, K.F., Wickramaratne, P., Speelman, L., Ndogoni, L., Weissman, M., 2003. Group Interpersonal Psychotherapy for Depression in Rural Uganda: A Randomized Controlled Trial. J. Am. Med. Assoc. 289, 3117–3124. https://doi.org/10.1001/jama.289.23.3117

Brown, L., Thurman, T., Rice, J., Boris, N., Ntaganira, J., Nyirazinyoye, L., De Dieu, J., Snider, L., 2009. Impact of a mentoring program on psychosocial wellbeing of youth in Rwanda: Results of a quasi-experimental study. Vulnerable Child. Youth Stud. 4, 288–299. https://doi.org/10.1080/17450120903193915

Bryant, R.A., Schafer, A., Dawson, K.S., Anjuri, D., Mulili, C., Ndogoni, L., Koyiet, P., Sijbrandij, M., Ulate, J., Harper Shehadeh, M., Hadzi-Pavlovic, D., van Ommeren, M., 2017. Effectiveness of a brief behavioural intervention on psychological distress among women with a history of gender-based violence in urban Kenya: A randomised clinical trial. PLoS Med. 14. https://doi.org/10.1371/journal.pmed.1002371

Burgess, R.A., Jeske, N., Rasool, S., Ahmad, A., Kydd, A., Ncube Mlilo, N., 2021. Exploring the impact of a complex intervention for women with depression in contexts of adversity: A pilot feasibility study of COURRAGE-plus in South Africa. Int. J. Soc. Psychiatry 207640211010203. https://doi.org/10.1177/00207640211010203

Carney, T., Browne, F.A., Myers, B., Kline, T.L., Howard, B., Wechsberg, W.M., 2019. Adolescent female school dropouts who use drugs and engage in risky sex: effects of a brief pilot intervention in Cape Town, South Africa. AIDS Care - Psychol. Socio-Med. Asp. AIDSHIV 31, 77–84. https://doi.org/10.1080/09540121.2018.1500008

Carney, T., Johnson, K., Carrico, A., Myers, B., 2020. Acceptability and feasibility of a brief substance use intervention for adolescents in Cape Town, South Africa: A pilot study. Int. J. Psychol. J. Int. Psychol. 55, 1016–1025. https://doi.org/10.1002/ijop.12668

Carney, T., Petersen Williams, P.M., Parry, C.D.H., 2016. Ithubalethu-Intervention to Address Drug Use and Sexual HIV Risk Patterns among Female Commercial Sex Workers in Durban, South Africa. J. Psychoactive Drugs 48, 303–309. https://doi.org/10.1080/02791072.2016.1208855

Carries, S., Mkhwanazi, Z., Sigwadhi, L., Moshabela, M., Nyirenda, M., Goudge, J., Govindasamy, D., 2023. An economic incentive package to support the wellbeing of caregivers of adolescents living with HIV during the COVID-19 pandemic in South Africa: a feasibility study protocol for a pilot randomised trial. Pilot Feasibility Stud. 9. https://doi.org/10.1186/s40814-023-01237-x

Cetrone, H.M., Santoso, M.V., Bezner Kerr, R., Petito, L., Blacker, L., Nonga, T., Martin, H.D., Kassim, N., Mtinda, E., Young, S.L., 2021. Food security mediates the decrease in women's depressive symptoms in a participatory nutrition-sensitive agroecology intervention in rural Tanzania. Public Health Nutr. 1–29. https://doi.org/10.1017/S1368980021001014

Chaudhury, S., Brown, F.L., Kirk, C.M., Mukunzi, S., Nyirandagijimana, B., Mukandanga, J., Ukundineza, C., Godfrey, K., Ng, L.C., Brennan, R.T., Betancourt, T.S., 2016. Exploring the potential of a family-based prevention intervention to reduce alcohol use and violence within HIV-affected families in Rwanda. AIDS Care - Psychol. Socio-Med. Asp. AIDSHIV 28, 118–129. https://doi.org/10.1080/09540121.2016.1176686

Chipps, J., Penelope, M., Jeffrey, H., Margaret, W., Olivia, B., Fiona, W., Jarvis, M.A., 2022. Evaluation of a digital programme for final year nursing students during COVID-19. Int. J. Afr. Nurs. Sci. 17. https://doi.org/10.1016/j.ijans.2022.100455

Cilliers, J., Dube, O., Siddiqi, B., 2016. Psychology: Reconciling after civil conflict increases social capital but decreases individual well-being. Science 352, 787–794. https://doi.org/10.1126/science.aad9682

Cluver, L.D., Meinck, F., Steinert, J.I., Shenderovich, Y., Doubt, J., Herrero Romero, R., Lombard, C.J., Redfern, A., Ward, C.L., Tsoanyane, S., Nzima, D., Sibanda, N., Wittesaele, C., De Stone, S., Boyes, M.E., Catanho, R., Lachman, J.M., Salah, N., Nocuza, M., Gardner, F., 29564157, 2018. Parenting for Lifelong Health: a pragmatic cluster randomised controlled trial of a non-commercialised parenting programme for adolescents and their families in South Africa. BMJ Glob. Health 3, e000539. https://doi.org/10.1136/bmjgh-2017-000539

Coetzee, B.J., Loades, M.E., Human, S., Gericke, H., Loxton, H., Laning, G., Myburgh, N., Stallard, P., 2022. 4 Steps To My Future (4STMF): protocol for a universal school-based pilot and feasibility study of a CBT-based psychoeducational intervention to support psychological well-being amongst young adolescents in the Western Cape, South Africa. Pilot Feasibility Stud. 8. https://doi.org/10.1186/s40814-022-01035-x

Cohen, F., Hermosilla, S., Knox, J., Agaba, G.S., Obalim, G., Kajungu, R., Mangen, P.O., Stark, L., 2021. Protocol for a caregiver psychosocial support intervention for populations affected by

displacement in Uganda. BMC Public Health 21, 932. https://doi.org/10.1186/s12889-021-10921-7

Comrie-Thomson, L., Webb, K., Patel, D., Wata, P., Kapamurandu, Z., Mushavi, A., Nicholas, M.A., Agius, P.A., Davis, J., Luchters, S., 2022. Engaging women and men in the gender-synchronised, community-based Mbereko+Men intervention to improve maternal mental health and perinatal care-seeking in Manicaland, Zimbabwe: A cluster-randomised controlled pragmatic trial. J. Glob. Health 12, 04042. https://doi.org/10.7189/jogh.12.04042

Connolly, S., Sakai, C., 2011. Brief trauma intervention with Rwandan genocide-survivors using thought field therapy. Int. J. Emerg. Ment. Health 13, 161–172.

Constant, D., De Tolly, K., Harries, J., Myer, L., 2014. Mobile phone messages to provide support to women during the home phase of medical abortion in South Africa: A randomised controlled trial. Contraception 90, 226–233. https://doi.org/10.1016/j.contraception.2014.04.009

Cooper, P.J., Landman, M., Tomlinson, M., Molteno, C., Swartz, L., Murray, L., 2002. Impact of a mother-infant intervention in an indigent peri-urban South African context. Pilot study. Br. J. Psychiatry 180, 76–81. https://doi.org/10.1192/bjp.180.1.76

Cooper, P.J., Tomlinson, M., Swartz, L., Landman, M., Molteno, C., Stein, A., McPherson, K., Murray, L., 2009. Improving quality of mother-infant relationship and infant attachment in socioeconomically deprived community in South Africa: randomised controlled trial. BMJ 338, b974. https://doi.org/10.1136/bmj.b974

Crombach, A., Siehl, S., 2018. Impact and cultural acceptance of the Narrative Exposure Therapy in the aftermath of a natural disaster in Burundi. BMC Psychiatry 18. https://doi.org/10.1186/s12888-018-1799-3

Cubbins, L.A., Kasprzyk, D., Montano, D., Jordan, L.P., Woelk, G., 2012. Alcohol use and abuse among rural Zimbabwean adults: A test of a community-level intervention. Drug Alcohol Depend. 124, 333–339. https://doi.org/10.1016/j.drugalcdep.2012.02.002

Dambi, J., Norman, C., Doukani, A., Potgieter, S., Turner, J., Musesengwa, R., Verhey, R., Chibanda, D., 2022. A Digital Mental Health Intervention (Inuka) for Common Mental Health Disorders in Zimbabwean Adults in Response to the COVID-19 Pandemic: Feasibility and Acceptability Pilot Study. JMIR Ment. Health 9. https://doi.org/10.2196/37968

Dawson, K.S., Schafer, A., Anjuri, D., Ndogoni, L., Musyoki, C., Sijbrandij, M., van Ommeren, M., Bryant, R.A., 2016. Feasibility trial of a scalable psychological intervention for women affected by urban adversity and gender-based violence in Nairobi. BMC Psychiatry 16. https://doi.org/10.1186/s12888-016-1117-x

de Fouchier, C., Kedia, M.S., 2018. Trauma-related mental health problems and effectiveness of a stress management group in national humanitarian workers in the Central African Republic. Interv. 15718883 16, 103–109. https://doi.org/10.4103/INTV.INTV_9_18

Draper, C.E., Tomaz, S.A., Zihindula, G., Bunn, C., Gray, C.M., Hunt, K., Micklesfield, L.K., Wyke, S., 2019. Development, feasibility, acceptability and potential effectiveness of a healthy lifestyle programme delivered in churches in urban and rural South Africa. PLoS ONE 14. https://doi.org/10.1371/journal.pone.0219787

Dunkle, K., Stern, E., Chatterji, S., Heise, L., 2020. Effective prevention of intimate partner violence through couples training: a randomised controlled trial of Indashyikirwa in Rwanda. BMJ Glob. Health 5, 12. https://doi.org/10.1136/bmjgh-2020-002439

Duwell, M.M., Knowlton, A.R., Nachega, J.B., Efron, A., Goliath, R., Morroni, C., Maartens, G., Chaisson, R.E., 2013. Patient-nominated, community-based HIV Treatment Supporters: Patient perspectives, feasibility, challenges, and factors for success in HIV-Infected South African Adults. AIDS Patient Care STDs 27, 96–102. https://doi.org/10.1089/apc.2012.0348

Edeh, N.I., Ugwoke, E.O., Anaele, E.N., Madusaba, B.M., Naboth-Odums, A., Isiwu, E.A., Olinya, T.O., Enyi, C., David, A.O., Yumma, D.C., Yeldim, T.P., Odunukwe, M.C., Victor, S.N., Abubakar, A., 2022. Supporting business educators and students against COVID-19 trauma using trauma-focused cognitive behavioral therapy. Med. U. S. 101. https://doi.org/10.1097/MD.0000000000029133

Egbe, T.I., Omollo, O.D., Wesonga, J.O., Bair, E.F., Chakrabarti, A., Putt, M.E., Celum, C.L., Camlin, C.S., Napierala, S., Agot, K., Thirumurthy, H., 2022. A savings intervention to reduce men’s engagement in HIV risk behaviors: study protocol for a randomized controlled trial. Trials 23. https://doi.org/10.1186/s13063-022-06927-0

Egbegi, D.R., Bella-Awusah, T., Omigbodun, O., Ani, C., 2021. A controlled trial of Cognitive Behavioural Therapy-based strategies for insomnia among in-school adolescents in southern Nigeria. Child Adolesc. Psychiatry Ment. Health 15. https://doi.org/10.1186/s13034-021-00406-1

Eifediyi, G., Ojugo, A.I., Aluede, O., 2018. Effectiveness of rational emotive behaviour therapy in the reduction of examination Anxiety among secondary school students in Edo State, Nigeria. Asia Pac. J. Couns. Psychother. 9, 61–76. https://doi.org/10.1080/21507686.2017.1412329

Ekeke, N., Ossai, E.N., Kreibich, S., Onyima, A., Chukwu, J., Nwafor, C., Meka, A., Murphy-Okpala, N., Henry, P., Eze, C., 2022. A cluster randomized trial for improving mental health and well-being of persons affected by leprosy or buruli ulcer in Nigeria: A study protocol. Int. J. Mycobacteriology 11, 133–138. https://doi.org/10.4103/ijmy.ijmy_247_21

Eller, L.S., Kirksey, K.M., Nicholas, P.K., Corless, I.B., Holzemer, W.L., Wantland, D.J., Willard, S.S., Robinson, L., Hamilton, M.J., Sefcik, E.F., Moezzi, S., Mendez, M.R., Rosa, M., Human, S., 22880943, 2013. A randomized controlled trial of an HIV/AIDS Symptom Management Manual for depressive symptoms. AIDS Care 25, 391–399. https://doi.org/10.1080/09540121.2012.712662

Ertl, V., Groß, M., Mwaka, S.O., Neuner, F., 2021. Treating alcohol use disorder in the absence of specialized services – evaluation of the moving inpatient Treatment Camp approach in Uganda. BMC Psychiatry 21. https://doi.org/10.1186/s12888-021-03593-5

Ertl, V., Pfeiffer, A., Schauer, E., Elbert, T., Neuner, F., 2011. Community-implemented trauma therapy for former child soldiers in Northern Uganda: A randomized controlled trial. JAMA - J. Am. Med. Assoc. 306, 503–512. https://doi.org/10.1001/jama.2011.1060

Eseadi, C., Ilechukwu, L.C., Victor-Aigbodion, V., Sewagegn, A.A., Amedu, A.N., 2022. Intervention for depression among undergraduate religious education students: A randomized controlled trial. Med. U. S. 101, E31034. https://doi.org/10.1097/MD.0000000000031034

Eze, I.I., Mbachu, C.O., Azuogu, B.N., Ossai, E., Unah, A.I., Akamike, I.C., Onwasigwe, C.N., 2021. Effect of on-site behavioural modification intervention on lifestyle risk factors of hypertension among adult market traders in Abakaliki, Nigeria. Int. J. Health Promot. Educ. 59, 35–49. https://doi.org/10.1080/14635240.2020.1713188

Fabbri, C., Rodrigues, K., Leurent, B., Allen, E., Qiu, M., Zuakulu, M., Nombo, D., Kaemingk, M., De Filippo, A., Torrats-Espinosa, G., Shayo, E., Barongo, V., Greco, G., Tol, W., Devries, K.M., 2021. The EmpaTeach intervention for reducing physical violence from teachers to students in Nyarugusu Refugee Camp: A clusterrandomised controlled trial. PLoS Med. 18. https://doi.org/10.1371/journal.pmed.1003808

Fernando, S., Brown, T., Datta, K., Chidhanguro, D., Tavengwa, N.V., Chandna, J., Munetsi, E., Dzapasi, L., Nyachowe, C., Mutasa, B., Chasekwa, B., Ntozini, R., Chibanda, D., Prendergast, A.J., 2021. The Friendship Bench as a brief psychological intervention with peer support in rural Zimbabwean women: a mixed methods pilot evaluation. Glob Ment Health Camb 8, e31. https://doi.org/10.1017/gmh.2021.32

Figge, C.J., Kane, J.C., Skavenski, S., Haroz, E., Mwenge, M., Mulemba, S., Aldridge, L.R., Vinikoor, M.J., Sharma, A., Inoue, S., Paul, R., Simenda, F., Metz, K., Bolton, C., Kemp, C., Bosomprah, S., Sikazwe, I., Murray, L.K., 2022. Comparative effectiveness of in-person vs. remote delivery of the Common Elements Treatment Approach for addressing mental and behavioral health problems among adolescents and young adults in Zambia: protocol of a three-arm randomized controlled trial. Trials 23. https://doi.org/10.1186/s13063-022-06319-4

Fine, S.L., Malik, A., Guimond, M.F., Nemiro, A., Temu, G., Likindikoki, S., Annan, J., Tol, W.A., 2021. Improving mental health in low-resource settings: A feasibility randomized controlled trial of a transdiagnostic psychological intervention among Burundian refugee adolescents and their caregivers. Behav. Res. Ther. 145. https://doi.org/10.1016/j.brat.2021.103944

Geffen, L.N., Kelly, G., Morris, J.N., Howard, E.P., 2019. Peer-to-peer support model to improve quality of life among highly vulnerable, low-income older adults in Cape Town, South Africa. BMC Geriatr. 19, 279. https://doi.org/10.1186/s12877-019-1310-0

Gericke, F., Ebert, D.D., Breet, E., Auerbach, R.P., Bantjes, J., 2021. A qualitative study of university students' experience of internet-based cbt for depression. Couns. Psychother. Res. No Pagination Specified. https://doi.org/10.1002/capr.12465

Giusto, A., Ayuku, D., Puffer, E.S., 2022. Learn, Engage, Act, Dedicate (LEAD): development and feasibility testing of a task-shifted intervention to improve alcohol use, depression and family engagement for fathers. Int. J. Ment. Health Syst. 16. https://doi.org/10.1186/s13033-022-00522-1

Giusto, A., Green, E.P., Simmons, R.A., Ayuku, D., Patel, P., Puffer, E.S., 2020. A multiple baseline study of a brief alcohol reduction and family engagement intervention for fathers in Kenya. J. Consult. Clin. Psychol. 88, 708–725. https://doi.org/10.1037/ccp0000559

Giusto, A., Johnson, S.L., Lovero, K.L., Wainberg, M.L., Rono, W., Ayuku, D., Puffer, E.S., 2021. Building community-based helping practices by training peer-father counselors: A novel intervention to reduce drinking and depressive symptoms among fathers through an expanded masculinity lens. Int. J. Drug Policy 95. https://doi.org/10.1016/j.drugpo.2021.103291

Glass, N., Perrin, N.A., Kohli, A., Campbell, J., Remy, M.M., 2017. Randomised controlled trial of a livestock productive asset transfer programme to improve economic and health outcomes and reduce intimate partner violence in a postconflict setting. BMJ Glob. Health 2, e000165. https://doi.org/10.1136/bmjgh-2016-000165

Goodman, M.L., Elliott, A.J., Gitari, S., Keiser, P., Onwuegbuchu, E., Michael, N., Seidel, S., 2020. Come Together to Decrease Depression: Women's mental health, social capital, and

participation in a Kenyan combined microfinance program. Int. J. Soc. Psychiatry 20764020966014. https://doi.org/10.1177/0020764020966014

Green, E.P., Blattman, C., Jamison, J., Annan, J., 2016. Does poverty alleviation decrease depression symptoms in post-conflict settings? A cluster-randomized trial of microenterprise assistance in Northern Uganda. Glob. Ment. Health 3, e7. https://doi.org/10.1017/gmh.2015.28

Green, E.P., Cho, H., Gallis, J., Puffer, E.S., 2019. The impact of school support on depression among adolescent orphans: a cluster-randomized trial in Kenya. J. Child Psychol. Psychiatry 60, 54–62. https://doi.org/10.1111/jcpp.12955

Green, E.P., Lai, Y., Pearson, N., Rajasekharan, S., Rauws, M., Joerin, A., Kwobah, E., Musyimi, C., Jones, R.M., Bhat, C., Mulinge, A., Puffer, E.S., 33016883, 2020. Expanding Access to Perinatal Depression Treatment in Kenya Through Automated Psychological Support: Development and Usability Study. JMIR Form. Res. 4, e17895. https://doi.org/10.2196/17895

Greene, M.C., Likindikoki, S., Rees, S., Bonz, A., Kaysen, D., Misinzo, L., Njau, T., Kiluwa, S., Turner, R., Ventevogel, P., Mbwambo, J.K.K., Tol, W.A., 2021. Evaluation of an integrated intervention to reduce psychological distress and intimate partner violence in refugees: Results from the Nguvu cluster randomized feasibility trial. PLoS ONE 16. https://doi.org/10.1371/journal.pone.0252982

Greene, M.C., Rees, S., Likindikoki, S., Bonz, A.G., Joscelyne, A., Kaysen, D., Nixon, R.D.V., Njau, T., Tankink, M.T.A., Tiwari, A., Ventevogel, P., Mbwambo, J.K.K., Tol, W.A., 31428190, 2019. Developing an integrated intervention to address intimate partner violence and psychological distress in Congolese refugee women in Tanzania. Confl. Health Electron. Resour. 13, 38. https://doi.org/10.1186/s13031-019-0222-0

Greene, M.C., Scognamiglio, T., Likindikoki, S.L., Misinzo, L., Njau, T., Bonz, A., Ventevogel, P., Mbwambo, J.K.K., Tol, W.A., 2022. Examining implementation of an intervention to reduce psychological distress and intimate partner violence in a refugee camp setting. Glob. Public Health 17, 2868–2882. https://doi.org/10.1080/17441692.2022.2029926

Gupta, L., Zimmer, C., 2008. Psychosocial intervention for war-affected children in Sierra Leone. Br. J. Psychiatry 192, 212–216. https://doi.org/10.1192/bjp.bp.107.038182

Habimana, S., Biracyaza, E., Habumugisha, E., Museka, E., Mutabaruka, J., Montgomery, S.B., 2021. Role of Community Resiliency Model Skills Trainings in Trauma Healing Among 1994 Tutsi Genocide Survivors in Rwanda. Psychol. Res. Behav. Manag. 14, 1139–1148. https://doi.org/10.2147/PRBM.S319057

Harder, V.S., Musau, A.M., Musyimi, C.W., Ndetei, D.M., Mutiso, V.N., 2020. A randomized clinical trial of mobile phone motivational interviewing for alcohol use problems in Kenya. Addict. Abingdon Engl. 115, 1050–1060. https://doi.org/10.1111/add.14903

Harding, R., Wei, G., Gwyther, L., Miti, E., 2019. Improving psychological outcomes for orphans living with HIV in Tanzania through a novel intervention to improve resilience: findings from a pilot RCT. AIDS Care 31, 340–348. https://doi.org/10.1080/09540121.2018.1533630

Hatcher, A.M., Lemus Hufstedler, E., Doria, K., Dworkin, S.L., Weke, E., Conroy, A., Bukusi, E.A., Cohen, C.R., Weiser, S.D., 2020. Mechanisms and perceived mental health changes after a livelihood intervention for HIV-positive Kenyans: Longitudinal, qualitative findings. Transcult. Psychiatry 57, 124–139. https://doi.org/10.1177/1363461519858446

Hermenau, K., Hecker, T., Ruf, M., Schauer, E., Elbert, T., Schauer, M., 2011. Childhood adversity, mental ill-health and aggressive behavior in an African orphanage: Changes in response to trauma-focused therapy and the implementation of a new instructional system. Child Adolesc. Psychiatry Ment. Health 5. https://doi.org/10.1186/1753-2000-5-29

Hoss, J.M., Blokland, L.E., Weierstall, R., 2019. Questioning the mental health effects of community-based interventions: Evaluation of a women empowerment programme. South Afr. J. Psychol. 49, 282–292. https://doi.org/10.1177/0081246318789539

Igreja, V., Kleijn, W.C., Schreuder, B.J.N., Van Dijk, J.A., Verschuur, M., 2004. Testimony method to ameliorate post-traumatic stress symptoms: Community-based intervention study with Mozambican civil war survivors. Br. J. Psychiatry 184, 251–257. https://doi.org/10.1192/bjp.184.3.251

Im, H., Jettner, J.F., Warsame, A.H., Isse, M.M., Khoury, D., Ross, A.I., 2018. Trauma-Informed Psychoeducation for Somali Refugee Youth in Urban Kenya: Effects on PTSD and Psychosocial Outcomes. J. Child Adolesc. Trauma 11, 431–441. https://doi.org/10.1007/s40653-017-0200-x

Ireri, N.W., White, S.W., Mbwayo, A.W., 2019. Treating anxiety and social deficits in children with autism spectrum disorder in two schools in Nairobi, Kenya. J. Autism Dev. Disord. 49, 3309–3315. https://doi.org/10.1007/s10803-019-04045-6

Ismayilova, L., Karimli, L., Sanson, J., Gaveras, E., Nanema, R., Tô-Camier, A., Chaffin, J., 2018. Improving mental health among ultra-poor children: Two-year outcomes of a cluster-randomized trial in Burkina Faso. Soc. Sci. Med. 208, 180–189. https://doi.org/10.1016/j.socscimed.2018.04.022

Jani, N., Vu, L., Kay, L., Habtamu, K., Kalibala, S., 2016. Reducing HIV-related risk and mental health problems through a client-centred psychosocial intervention for vulnerable adolescents in Addis Ababa, Ethiopia. J. Int. AIDS Soc. 19. https://doi.org/10.7448/IAS.19.5.20832

Jansen, S., Niyonsenga, J., Ingabire, C.M., Jansen, A., Nzabonimpa, E., Ingabire, N., Kangabe, J., Sarabwe, E., Richters, A., Rutayisire, T., Nsabimana, E., 2022. Evaluating the impact of Community-Based Sociotherapy on social dignity in post-genocide Rwanda: study protocol for a cluster randomized controlled trial. Trials 23. https://doi.org/10.1186/s13063-022-06994-3

Jewkes, R., Nduna, M., Levin, J., Jama, N., Dunkle, K., Puren, A., Duvvury, N., 2008. Impact of Stepping Stones on incidence of HIV and HSV-2 and sexual behaviour in rural South Africa: Cluster randomised controlled trial. BMJ 337, 391–395. https://doi.org/10.1136/bmj.a506

Jibunoh, O., Ani, C., 2021. A controlled clinical trial of a brief psycho-educational intervention for anxiety among in-school adolescents in nigeria. Int. J. Ment. Health No Pagination Specified. https://doi.org/10.1080/00207411.2021.1891362

Jordans, M.J., Tol, W.A., Ndayisaba, A., Komproe, I.H., 2013. A controlled evaluation of a brief parenting psychoeducation intervention in Burundi. Soc. Psychiatry Psychiatr. Epidemiol. 48, 1851–1859. https://doi.org/10.1007/s00127-012-0630-6

Kagabo, D.M., Bangirana, P., Burnside, G., Chiumento, A., Duarte, R., Gishoma, D., Girvan, M., Jansen, A., Jansen, S., Kasujja, R., Lubunga, R., Nevitt, S., Nzaramba, L., Sarabwe, E., Jackson, C., Rahman, A., Richters, A., Robinson, J., Rutayisire, T., Ventevogel, P., White, R.G., 2023. Community based sociotherapy for depressive symptomatology of Congolese

refugees in Rwanda and Uganda (CoSTAR): a protocol for a cluster randomised controlled trial. Eur J Psychotraumatology 14. https://doi.org/10.1080/20008066.2022.2151281

Kalichman, S.C., Mathews, C., Banas, E., Kalichman, M.O., 2019. Stigma management intervention to improve antiretroviral therapy adherence: Phase-I test of concept trial, Cape Town South Africa. Glob. Public Health 14, 1059–1074. https://doi.org/10.1080/17441692.2018.1552307

Karnell, A.P., Cupp, P.K., Zimmerman, R.S., Feist-Price, S., Bennie, T., 2006. Efficacy of an American alcohol and HIV prevention curriculum adapted for use in South Africa: Results of a pilot study in five township schools. AIDS Educ. Prev. 18, 295–310. https://doi.org/10.1521/aeap.2006.18.4.295

Khuzwayo, N., Taylor, M., Connolly, C., 2020. Changing youth behaviour in South Africa. Health SA Gesondheid 25, 1–7. https://doi.org/10.4102/hsag.v25i0.1031

Kumakech, E., Cantor-Graae, E., Maling, S., Bajunirwe, F., 2009. Peer-group support intervention improves the psychosocial well-being of AIDS orphans: Cluster randomized trial. Soc. Sci. Med. 68, 1038–1043. https://doi.org/10.1016/j.socscimed.2008.10.033

Kuo, C., Mathews, C., Giovenco, D., Atujuna, M., Beardslee, W., Hoare, J., Stein, D.J., Brown, L.K., 2020. Acceptability, feasibility, and preliminary efficacy of a resilience-oriented family intervention to prevent adolescent HIV and depression: A pilot randomized controlled trial. AIDS Educ. Prev. 32, 67–81. https://doi.org/10.1521/aeap.2020.32.1.67

Lachman, J., Wamoyi, J., Spreckelsen, T., Wight, D., Maganga, J., Gardner, F., 2020. Combining parenting and economic strengthening programmes to reduce violence against children: a cluster randomised controlled trial with predominantly male caregivers in rural Tanzania. BMJ Glob. Health 5, 07. https://doi.org/10.1136/bmjgh-2020-002349

Lasebikan, V., Ola, B.A., Ayinde, O.O., 2017. Effectiveness of alcohol, smoking, and substance involvement screening test-linked brief intervention on harmful and hazardous alcohol use in Nigerian semirural communities: A non-randomized intervention study. Front. Psychiatry 8. https://doi.org/10.3389/fpsyt.2017.00050

Lawrence, K.C., Falaye, A.O., 2020. Trauma-focused counselling and social effectiveness skills training interventions on impaired psychological functioning of internally displaced adolescents in Nigeria. J. Community Appl. Soc. Psychol. 30, 616–627. https://doi.org/10.1002/casp.2477

le Roux, K.W., Almirol, E., Rezvan, P.H., le Roux, I.M., Mbewu, N., Dippenaar, E., Stansert-Katzen, L., Baker, V., Tomlinson, M., Rotheram-Borus, M.J., 2020. Community health workers impact on maternal and child health outcomes in rural South Africa - a non-randomized two-group comparison study. BMC Public Health 20, 1404. https://doi.org/10.1186/s12889-020-09468-w

L’Engle, K.L., Mwarogo, P., Kingola, N., Sinkele, W., Weiner, D.H., 2014. A randomized controlled trial of a brief intervention to reduce alcohol use among female sex workers in Mombasa, Kenya. J. Acquir. Immune Defic. Syndr. JAIDS 67, 446–453. https://doi.org/10.1097/QAI.0000000000000335

Lloyd-Sherlock, P., Agrawal, S., Amoakoh-Coleman, M., Adom, S., Adjetey-Sorsey, E., Rocco, I., Minicuci, N., 2019. Old age and depression in Ghana: assessing and addressing diagnosis and

treatment gaps. Glob. Health Action 12, 1678282. https://doi.org/10.1080/16549716.2019.1678282

Logie, C., Okumu, M., Hakiza, R., Kibuuka Musoke, D., Berry, I., Mwima, S., Kyambadde, P., Kiera, U.M., Loutet, M., Neema, S., Newby, K., McNamee, C., Baral, S.D., Lester, R., Musinguzi, J., Mbuagbaw, L., 33528378, 2021. Mobile Health-Supported HIV Self-Testing Strategy Among Urban Refugee and Displaced Youth in Kampala, Uganda: Protocol for a Cluster Randomized Trial (Tushirikiane, Supporting Each Other). JMIR Res. Protoc. 10, e26192. https://doi.org/10.2196/26192

Logie, C., Okumu, M., Kortenaar, J.L., Gittings, L., Khan, N., Hakiza, R., Kibuuka Musoke, D., Nakitende, A., Katisi, B., Kyambadde, P., Khan, T., Lester, R., Mbuagbaw, L., 2022. Mobile Health-Supported Virtual Reality and Group Problem Management Plus: Protocol for a Cluster Randomized Trial Among Urban Refugee and Displaced Youth in Kampala, Uganda (Tushirikiane4MH, Supporting Each Other for Mental Health). JMIR Res Protoc 11, e42342. https://doi.org/10.2196/42342

Lund, C., Schneider, M., Davies, T., Nyatsanza, M., Honikman, S., Bhana, A., Bass, J., Bolton, P., Dewey, M., Joska, J., Kagee, A., Myer, L., Petersen, I., Prince, M., Stein, D.J., Thornicroft, G., Tomlinson, M., Alem, A., Susser, E., 2014. Task sharing of a psychological intervention for maternal depression in Khayelitsha, South Africa: Study protocol for a randomized controlled trial. Trials 15. https://doi.org/10.1186/1745-6215-15-457

Lund, C., Waruguru, M., Kingori, J., Kippen-Wood, S., Breuer, E., Mannarathd, S., Raja, S., 2013. Outcomes of the mental health and development model in rural Kenya: A 2-year prospective cohort intervention study. Int. Health 5, 43–50. https://doi.org/10.1093/inthealth/ihs037

Marfo, A.F.A., Owusu-Daaku, F.T., 2016. Evaluation of a pharmacist-led hypertension preventative and detection service in the Ghanaian community pharmacy: an exploratory study. Int. J. Pharm. Pract. 24, 341–348. https://doi.org/10.1111/ijpp.12263

Masulani-Mwale, C., Kauye, F., Gladstone, M., Mathanga, D., 2019. Development of a psycho-social intervention for reducing psychological distress among parents of children with intellectual disabilities in Malawi. PLoS ONE 14. https://doi.org/10.1371/journal.pone.0210855

Matsuba, M., Schonert-Reichl, K.A., McElroy, T., Katahoire, A., 2020. Effectiveness of a sel/mindfulness program on northern ugandan children. Int. J. Sch. Educ. Psychol. No Pagination Specified. https://doi.org/10.1080/21683603.2020.1760977

McLea, H.F., Mayers, P., 2017. The grief and trauma project: A group work approach to restoring emotional and spiritual health to women in bereaved and traumatised indigent communities in the Western Cape, South Africa. Soc. Work South Afr. 53, 423–444. https://doi.org/10.15270/52-2-590

McMullen, J.D., McMullen, N., 2018. Evaluation of a teacher-led, life-skills intervention for secondary school students in Uganda. Soc. Sci. Med. 217, 10–17. https://doi.org/10.1016/j.socscimed.2018.09.041

Moffett, B.D., Pozuelo, J.R., van Heerden, A., O'Mahen, H.A., Craske, M., Sodi, T., Lund, C., Orkin, K., Kilford, E.J., Blakemore, S.J., Mahmud, M., Musenge, E., Davis, M., Makhanya, Z., Baloyi, T., Mahlangu, D., Chierchia, G., Fielmann, S.L., Gómez-Olivé, F.X., Valodia, I., Tollman, S., Kahn, K., Stein, A., 2022. Digital delivery of behavioural activation therapy to overcome depression and facilitate social and economic transitions of adolescents in South

Africa (the DoBAt study): protocol for a pilot randomised controlled trial. BMJ Open 12, e065977. https://doi.org/10.1136/bmjopen-2022-065977

Monnapula-Mazabane, P., Petersen, I., 2022. Feasibility and acceptability of a mental health stigma intervention for low-income South African caregivers: A qualitative investigation. South Afr. J. Psychiatry 28. https://doi.org/10.4102/sajpsychiatry.v28i0.1824

Morojele, N.K., Kitleli, N., Ngako, K., Kekwaletswe, C.T., Nkosi, S., Fritz, K., Parry, C.D., 2014. Feasibility and acceptability of a bar-based sexual risk reduction intervention for bar patrons in Tshwane, South Africa. SAHARA J J. Soc. Asp. HIVAIDS Res. Alliance SAHARA Hum. Sci. Res. Counc. 11, 1–9. https://doi.org/10.1080/17290376.2014.890123

Mpande, E., Higson-Smith, C., Chimatira, R.J., Kadaira, A., Mashonganyika, J., Ncube, Q.M., Ngwenya, S., Vinson, G., Wild, R., Ziwoni, N., 2013-16201-004, 2013. Community intervention during ongoing political violence: What is possible? What works? Peace Confl. J. Peace Psychol. 19, 196–208. https://doi.org/10.1037/a0032529

Muriungi, S.K., Ndetei, D.M., 2013. Effectiveness of psycho-education on depression, hopelessness, suicidality, anxiety and substance use among basic diploma students at Kenya Medical Training College. South Afr. J. Psychiatry 19, 41–50. https://doi.org/10.7196/SAJP.401

Murray, L.K., Hall, B.J., Dorsey, S., Ugueto, A.M., Puffer, E.S., Sim, A., Ismael, A., Bass, J., Akiba, C., Lucid, L., Harrison, J., Erikson, A., Bolton, P.A., 2018. An evaluation of a common elements treatment approach for youth in Somali refugee camps. Glob. Ment. Health 5. https://doi.org/10.1017/gmh.2018.7

Musyoka, C.M., Mbwayo, A., Donovan, D.M., Mathai, M., 2023. Student peer mentoring: Feasibility and acceptability of mHealth-based tool for alcohol and substance abuse prevention by peer mentors at a university in Kenya. PLoS Digit. Health 1, 1–22. https://doi.org/10.1371/journal.pdig.0000177

Mutamba, B.B., Kane, J.C., de Jong, J.T.V.M., Okello, J., Musisi, S., Kohrt, B.A., 2018. Psychological treatments delivered by community health workers in low-resource government health systems: effectiveness of group interpersonal psychotherapy for caregivers of children affected by nodding syndrome in Uganda. Psychol. Med. 48, 2573–2583. https://doi.org/10.1017/S0033291718000193

Namasaba, M., Nabunje, S., Baguwemu, A.A., 2022. Effectiveness of multi-modal cognitive behavioural therapy in improving mental well-being among caregivers of children with disabilities in urban Uganda: A cluster-randomized controlled trial. J. Glob. Health 12, 04102. https://doi.org/10.7189/jogh.12.04102

Namy, S., Carlson, C., Morgan, K., Nkwanzi, V., Neese, J., 2021. Healing and resilience after trauma (hart) yoga: Programming with survivors of human trafficking in uganda. J. Soc. Work Pract. No Pagination Specified. https://doi.org/10.1080/02650533.2021.1934819

Negash, A., Khan, M.A., Medhin, G., Wondimagegn, D., Pain, C., Araya, M., 2021. Feasibility and acceptability of brief individual interpersonal psychotherapy among university students with mental distress in Ethiopia. BMC Psychol. 9, 64. https://doi.org/10.1186/s40359-021-00570-1

Nwobi, U.A., Eseadi, C., Obetta, K.C., Ekwealor, N., Ogbonnaya, K.A., Oboegbulem, A.I., Chinweuba, N.H., Mbagwu, F., Agundu, U.V., Okpoko, C., Ololo, K.O., Ohia, N.C., Nwankwor, P.P., Osilike, C., Okechukwu, E., Umoke, P.C.I., 30383633, 2018. A stress

management intervention for adults living with HIV in Nigerian community settings: An effects study. Medicine (Baltimore) 97, e12801. https://doi.org/10.1097/MD.0000000000012801

Nyongesa, M.K., Mwangome, E., Mwangi, P., Nasambu, C., Mbuthia, J.W., Koot, H.M., Cuijpers, P., Newton, C.R.J.C., Abubakar, A., 2022. Adaptation, acceptability and feasibility of Problem Management Plus (PM+) intervention to promote the mental health of young people living with HIV in Kenya: Formative mixed-methods research. BJPsych Open 8. https://doi.org/10.1192/bjo.2022.564

O'Callaghan, P., Branham, L., Shannon, C., Betancourt, T.S., Dempster, M., McMullen, J., 2014. A pilot study of a family focused, psychosocial intervention with war-exposed youth at risk of attack and abduction in north-eastern Democratic Republic of Congo. Child Abuse Negl. 38, 1197–1207. https://doi.org/10.1016/j.chiabu.2014.02.004

Odenwald, M., Lingenfelder, B., Peschel, W., Haibe, F.A., Warsame, A.M., Omer, A., Stöckel, J., Maedl, A., Elbert, T., 2012. A pilot study on community-based outpatient treatment for patients with chronic psychotic disorders in Somalia: Change in symptoms, functioning and co-morbid khat use. Int. J. Ment. Health Syst. 6. https://doi.org/10.1186/1752-4458-6-8

O'Donnell, K., Dorsey, S., Gong, W., Ostermann, J., Whetten, R., Cohen, J.A., Itemba, D., Manongi, R., Whetten, K., 2014. Treating maladaptive grief and posttraumatic stress symptoms in orphaned children in Tanzania: group-based trauma-focused cognitive-behavioral therapy. J. Trauma. Stress 27, 664–671. https://doi.org/10.1002/jts.21970

Ofoegbu, T.O., Asogwa, U., Otu, M.S., Ibenegbu, C., Muhammed, A., Eze, B., 2020. Efficacy of guided internet-assisted intervention on depression reduction among educational technology students of Nigerian universities. Med. U. S. 99. https://doi.org/10.1097/MD.0000000000018774

Ogum Alangea, D., Addo-Lartey, A.A., Chirwa, E.D., Sikweyiya, Y., Coker-Appiah, D., Jewkes, R., Adanu, R.M.K., 2020. Evaluation of the rural response system intervention to prevent violence against women: findings from a community-randomised controlled trial in the Central Region of Ghana. Glob. Health Action 13, 1711336. https://doi.org/10.1080/16549716.2019.1711336

Okube, O.T., Kimani, S., Mirie, W., 2022. Community-based lifestyle intervention improves metabolic syndrome and related markers among Kenyan adults. J. Diabetes Metab. Disord. 21, 607–621. https://doi.org/10.1007/s40200-022-01023-1

Olowokere, A.E., Okanlawon, F.A., 2018. Improving vulnerable school children's psychosocial health outcomes through resilience-based training and peer-support activities: a comparative prospective study. Vulnerable Child. Youth Stud. 13, 291–304. https://doi.org/10.1080/17450128.2018.1499988

Olowokere, A.E., Okanlawon, F.A., 2014. The effects of a school-based psychosocial intervention on resilience and health outcomes among vulnerable children. J. Sch. Nurs. Off. Publ. Natl. Assoc. Sch. Nurses 30, 206–215. https://doi.org/10.1177/1059840513501557

Onyechi, K.C.N., Onuigbo, L.N., Eseadi, C., Ikechukwu-Ilomuanya, A.B., Nwaubani, O.O., Umoke, P.C.I., Agu, F.U., Otu, M.S., Utoh-Ofong, A.N., 2016. Effects of rational-emotive hospice care therapy on problematic assumptions, death anxiety, and psychological distress in a sample of cancer patients and their family caregivers in Nigeria. Int. J. Environ. Res. Public. Health 13. https://doi.org/10.3390/ijerph13090929

Onyut, L.P., Neuner, F., Schauer, E., Ertl, V., Odenwald, M., Schauer, M., Elbert, T., 2005-01751-002, 2004. The Nakivale Camp Mental Health Project: Building local competency for psychological assistance to traumatised refugees. Interv. Int. J. Ment. Health Psychosoc. Work Couns. Areas Armed Confl. 2, 90–107.

Osafo, J., Andoh-Arthur, J., 2020. Engaging television for public education and counselling on suicide: A case example from ghana. Int. J. Adv. Couns. No Pagination Specified. https://doi.org/10.1007/s10447-020-09398-7

Osborn, T.L., Rodriguez, M., Wasil, A.R., Venturo-Conerly, K.E., Gan, J., Alemu, R.G., Roe, E., Arango G, S., Otieno, B.H., Wasanga, C.M., Shingleton, R., Weisz, J.R., 2020a. Single-Session digital intervention for adolescent depression, anxiety, and well-being: Outcomes of a randomized controlled trial with Kenyan adolescents. J. Consult. Clin. Psychol. 88, 657–668. https://doi.org/10.1037/ccp0000505

Osborn, T.L., Venturo-Conerly, K.E., Arango G, S., Roe, E., Rodriguez, M., Alemu, R.G., Gan, J., Wasil, A.R., Otieno, B.H., Rusch, T., Ndetei, D.M., Wasanga, C., Schleider, J.L., Weisz, J.R., 2021. Effect of Shamiri Layperson-Provided Intervention vs Study Skills Control Intervention for Depression and Anxiety Symptoms in Adolescents in Kenya: A Randomized Clinical Trial. JAMA Psychiatry 78, 829–837. https://doi.org/10.1001/jamapsychiatry.2021.1129

Osborn, T.L., Wasil, A.R., Venturo-Conerly, K.E., Schleider, J.L., Weisz, J.R., 2020b. Group Intervention for Adolescent Anxiety and Depression: Outcomes of a Randomized Trial with Adolescents in Kenya. Behav. Ther. 51, 601–615. https://doi.org/10.1016/j.beth.2019.09.005

Osiki, J., Busari, A., 2002. Effects of self-statements monitoring techniques in the reduction of test anxiety among adolescent underachievers in Ibadan Metropolis, Nigeria. Niger. J. Guid. Couns. 8, 133–141.

Pandya, S.P., 2018. Spirituality for Wellbeing of Bereaved Children in Residential Care: Insights for Spiritually Sensitive Child-Centred Social Work Across Country Contexts. Child Adolesc. Soc. Work J. 35, 181–195. https://doi.org/10.1007/s10560-017-0509-1

Parry, C.D.H., Carney, T., Petersen Williams, P., 2017. Reducing substance use and risky sexual behaviour among drug users in Durban, South Africa: Assessing the impact of community-level risk-reduction interventions. SAHARA J J. Soc. Asp. HIVAIDS Res. Alliance 14, 110–117. https://doi.org/10.1080/17290376.2017.1381640

Patel, L., Ross, E., 2020. Connecting cash transfers with care for better child and family well-being: Evidence from a qualitative evaluation in south africa. Child Adolesc. Soc. Work J. No Pagination Specified. https://doi.org/10.1007/s10560-020-00714-z

Pengpid, S., Peltzer, K., Skaal, L., 2014. Efficacy of a church-based lifestyle intervention programme to control high normal blood pressure and/or high normal blood glucose in church members: A randomized controlled trial in Pretoria, South Africa. BMC Public Health 14. https://doi.org/10.1186/1471-2458-14-568

Pengpid, S., Peltzer, K., Van der Heever, H., Skaal, L., 2013. Screening and brief interventions for hazardous and harmful alcohol use among university students in South Africa: Results from a randomized controlled trial. Int. J. Environ. Res. Public. Health 10, 2043–2057. https://doi.org/10.3390/ijerph10052043

Pettifor, A., Lippman, S.A., Gottert, A., Suchindran, C.M., Selin, A., Peacock, D., Maman, S., Rebombo, D., Twine, R., Gomez-Olive, F.X., Tollman, S., Kahn, K., MacPhail, C., 29972287, 2018. Community mobilization to modify harmful gender norms and reduce HIV risk: results from a community cluster randomized trial in South Africa. J. Int. AIDS Soc. 21, e25134. https://doi.org/10.1002/jia2.25134

Prencipe, L., Houweling, T.A.J., van Lenthe, F.J., Palermo, T., 2021. Do Conditional Cash Transfers Improve Mental Health? Evidence From Tanzania's Governmental Social Protection Program. J. Adolesc. Health. https://doi.org/10.1016/j.jadohealth.2021.04.033

Prinsloo, C.D., Greeff, M., Kruger, A., Ellis, S., 2016. Psychosocial well-being of people living with HIV and the community before and after a HIV stigma-reduction community "hub" network intervention. Afr. J. AIDS Res. 15, 261–271. https://doi.org/10.2989/16085906.2016.1200640

Puffer, E.S., Healy, E.F., Green, E.P., Giusto, A.M., Kaiser, B.N., Patel, P., Ayuku, D., 2020. Family Functioning and Mental Health Changes Following a Family Therapy Intervention in Kenya: a Pilot Trial. J. Child Fam. Stud. 29, 3493–3508. https://doi.org/10.1007/s10826-020-01816-z

Rabie, S., Tomlinson, M., Almirol, E., Stewart, J., Skiti, Z., Weiss, R.E., Vogel, L., Rotheram-Borus, M.J., 2022. Utilizing Soccer for Delivery of HIV and Substance Use Prevention for Young South African Men: 6-Month Outcomes of a Cluster Randomized Controlled Trial. AIDS Behav. https://doi.org/10.1007/s10461-022-03819-x

Ramdhonee-Dowlot, K., Balloo, K., Essau, C.A., 2021. Effectiveness of the Super Skills for Life programme in enhancing the emotional wellbeing of children and adolescents in residential care institutions in a low- and middle-income country: A randomised waitlist-controlled trial. J. Affect. Disord. 278, 327–338. https://doi.org/10.1016/j.jad.2020.09.053

Reeler, T., Chitsike, K., Maizva, F., Reeler, B., 2009. The Tree of Life: a community approach to empowering and healing the survivors of torture in Zimbabwe. Torture Q. J. Rehabil. Torture Vict. Prev. Torture 19, 180–193.

Richards, J., Foster, C., Townsend, N., Bauman, A., 2014. Physical fitness and mental health impact of a sport-for-development intervention in a post-conflict setting: randomised controlled trial nested within an observational study of adolescents in Gulu, Uganda. BMC Public Health 14, 619. https://doi.org/10.1186/1471-2458-14-619

Rivet-Duval, E., Heriot, S., Hunt, C., 2011. Preventing Adolescent Depression in Mauritius: A Universal School-Based Program. Child Adolesc. Ment. Health 16, 86–91. https://doi.org/10.1111/j.1475-3588.2010.00584.x

Robjant, K., Schmitt, S., Carleial, S., Elbert, T., Abreu, L., Chibashimba, A., Hinkel, H., Hoeffler, A., Rukundo Zeller, A.C., Rockstroh, B., Koebach, A., 2022. NETfacts: An integrated intervention at the individual and collective level to treat communities affected by organized violence. Proc. Natl. Acad. Sci. 119, e2204698119. https://doi.org/10.1073/pnas.2204698119

Rochat, T.J., Arteche, A.X., Stein, A., Mitchell, J., Bland, R.M., 2015. Maternal and child psychological outcomes of HIV disclosure to young children in rural South Africa: The Amagugu intervention. AIDS 29, S67–S79. https://doi.org/10.1097/QAD.0000000000000668

Rochat, T.J., Dube, S., Herbst, K., Hoegfeldt, C.A., Redinger, S., Khoza, T., Bland, R.M., Richter, L., Linsell, L., Desmond, C., Yousafzai, A.K., Craske, M., Juszczak, E., Abas, M., Edwards, T., Ekers, D., Stein, A., 2021. An evaluation of a combined psychological and parenting intervention for HIV-positive women depressed in the perinatal period, to enhance child

development and reduce maternal depression: study protocol for the Insika Yomama cluster randomised controlled. Trials 22, 914. https://doi.org/10.1186/s13063-021-05672-0

Rossouw, J., Yadin, E., Alexander, D., Mbanga, I., Jacobs, T., Seedat, S., 2016. A pilot and feasibility randomised controlled study of Prolonged Exposure Treatment and supportive counselling for post-traumatic stress disorder in adolescents: A third world, task-shifting, community-based sample. Trials 17. https://doi.org/10.1186/s13063-016-1677-6

Rossouw, J., Yadin, E., Alexander, D., Seedat, S., 2022. Long-term follow-up of a randomised controlled trial of prolonged exposure therapy and supportive counselling for post-traumatic stress disorder in adolescents: a task-shifted intervention. Psychol. Med. 52, 1022–1030. https://doi.org/10.1017/S0033291720002731

Rossouw, J., Yadin, E., Alexander, D., Seedat, S., 2018. Prolonged exposure therapy and supportive counselling for post-traumatic stress disorder in adolescents: task-shifting randomised controlled trial. Br. J. Psychiatry 213, 587–594. https://doi.org/10.1192/bjp.2018.130

Rossouw, L., Burger, R.P., Burger, R., 2021. Testing an Incentive-Based and Community Health Worker Package Intervention to Improve Maternal Health and Nutrition Outcomes: A Pilot Randomized Controlled Trial. Matern. Child Health J. 25, 1913–1922. https://doi.org/10.1007/s10995-021-03229-w

Rotheram-Borus, M.J., Arfer, K.B., Christodoulou, J., Comulada, W.S., Stewart, J., Tubert, J.E., Tomlinson, M., 2019. The association of maternal alcohol use and paraprofessional home visiting with children’s health: A randomized controlled trial. J. Consult. Clin. Psychol. 87, 551–562. https://doi.org/10.1037/ccp0000408

Rotheram-Borus, M.J., Le Roux, K., Le Roux, I.M., Christodoulou, J., Laurenzi, C., Mbewu, N., Tomlinson, M., 2017. To evaluate if increased supervision and support of South African Government health workers’ home visits improves maternal and child outcomes: Study protocol for a randomized control trial. Trials 18. https://doi.org/10.1186/s13063-017-2074-5

Rotheram-Borus, M.J., Tomlinson, M., Roux, I.L., Stein, J.A., 2015. Alcohol Use, Partner Violence, and Depression: A Cluster Randomized Controlled Trial Among Urban South African Mothers Over 3 Years. Am. J. Prev. Med. 49, 715–725. https://doi.org/10.1016/j.amepre.2015.05.004

Sakai, C.E., Connolly, S.M., Oas, P., 2010. Treatment of PTSD in Rwandan child genocide survivors using thought field therapy. Int. J. Emerg. Ment. Health 12, 41–49.

Schmitt, S., Robjant, K., Elbert, T., Carleial, S., Hoeffler, A., Chibashimba, A., Hinkel, H., Koebach, A., 2022. Breaking the cycles of violence with narrative exposure: Development and feasibility of NETfacts, a community-based intervention for populations living under continuous threat. PLoS ONE 17. https://doi.org/10.1371/journal.pone.0275421

Schneider, A., Pfeiffer, A., Conrad, D., Elbert, T., Kolassa, I.T., Wilker, S., 2020. Does cumulative exposure to traumatic stressors predict treatment outcome of community-implemented exposure-based therapy for PTSD? Eur. J. Psychotraumatology 11, 1789323. https://doi.org/10.1080/20008198.2020.1789323

Schouw, D.D., Mash, R., 2020. Cost and consequence analysis of the Healthy Choices at Work programme to prevent non-communicable diseases in a commercial power plant, South

Africa. Afr. J. Prim. Health Care Fam. Med. 12, e1–e8. https://doi.org/10.4102/phcfm.v12i1.2217

Searle, C., Skinner, D., 2000. A school-based intervention programme for refugee children. Soc. Work 36, 237–246.

Sherr, L., Yakubovich, A.R., Skeen, S., Cluver, L.D., Hensels, I.S., Macedo, A., Tomlinson, M., 2016. How effective is help on the doorstep? A longitudinal evaluation of community-based organisation support. PLoS ONE 11. https://doi.org/10.1371/journal.pone.0151305

Sherr, L., Yakubovich, A.R., Skeen, S., Tomlinson, M., Cluver, L.D., Roberts, K.J., Macedo, A., 2020. Depressive symptoms among children attending community based support in South Africa - pathways for disrupting risk factors. Clin. Child Psychol. Psychiatry 25, 984–1001. https://doi.org/10.1177/1359104520935502

Singla, D.R., Kumbakumba, E., Aboud, F.E., 2015. Effects of a parenting intervention to address maternal psychological wellbeing and child development and growth in rural Uganda: A community-based, cluster-randomised trial. Lancet Glob. Health 3. https://doi.org/10.1016/S2214-109X(15)00099-6

Smith, E.A., Palen, L.A., Caldwell, L.L., Flisher, A.J., Graham, J.W., Mathews, C., Wegner, L., Vergnani, T., 2008. Substance use and sexual risk prevention in Cape Town, South Africa: An evaluation of the HealthWise program. Prev. Sci. 9, 311–321. https://doi.org/10.1007/s11121-008-0103-z

Sonderegger, R., Rombouts, S., Ocen, B., McKeever, R.S., 2011. Trauma rehabilitation for war-affected persons in northern Uganda: a pilot evaluation of the EMPOWER programme. Br. J. Clin. Psychol. Br. Psychol. Soc. 50, 234–249.

Stansert Katzen, L., le Roux, K.W., Almirol, E., Hayati Rezvan, P., le Roux, I.M., Mbewu, N., Dippenaar, E., Baker, V., Tomlinson, M., Rotheram-Borus, M.J., 2020. Community health worker home visiting in deeply rural South Africa: 12-month outcomes. Glob. Public Health. https://doi.org/10.1080/17441692.2020.1833960

Stein, D.J., Wessels, C., Van Kradenberg, J., Emsley, R.A., 1997. The Mental Health Information Centre of South Africa: a report of the first 500 calls. Cent. Afr. J. Med. 43, 244–246.

Stepakoff, S., Hubbard, J., Katoh, M., Falk, E., Mikulu, J.B., Nkhoma, P., Omagwa, Y., 2006. Trauma healing in refugee camps in Guinea: A psychosocial program for Liberian and Sierra Leonean survivors of torture and war. Am. Psychol. 61, 921–932. https://doi.org/10.1037/0003-066X.61.8.921

Takahashi, R., Wilunda, C., Magutah, K., Mwaura-Tenambergen, W., Atwoli, L., Perngparn, U., 2018. Evaluation of alcohol screening and community-based brief interventions in rural western Kenya: A quasi-experimental study. Alcohol Alcohol 53, 121–128. https://doi.org/10.1093/alcalc/agx083

Talbot, A., Uwihoreye, C., Kamen, C., Grant, P., McGlynn, L., Mugabe, I., Nshimyumukiza, M., Dongier, P., Slamowitz, D., Padilla, C., Uvamahoro, J., Musayidire, I., Mukarubuga, A., Zolopa, A., 2013. Treating psychological trauma among Rwandan orphans is associated with a reduction in HIV risk-taking behaviors: A pilot study. AIDS Educ. Prev. Off. Publ. Int. Soc. AIDS Educ. 25, 468–479.

Thomson, D.R., Rich, M.L., Kaigamba, F., Socci, A.R., Hakizamungu, M., Bagiruwigize, E., Binagwaho, A., Franke, M.F., 2014. Community-based accompaniment and psychosocial

health outcomes in HIV-infected adults in Rwanda: a prospective study. AIDS Behav. 18, 368–380. https://doi.org/10.1007/s10461-013-0431-2

Thurman, T.R., Kidman, R., Taylor, T.M., 2014. Does investment in home visitors lead to better psychological health for HIV-affected families? Results from a quasi-experimental evaluation in South Africa. AIDS Care - Psychol. Socio-Med. Asp. AIDSHIV 26, S2–S10. https://doi.org/10.1080/09540121.2014.906555

Thurman, T.R., Luckett, B.G., Nice, J., Spyrelis, A., Taylor, T.M., 2017a. Effect of a bereavement support group on female adolescents' psychological health: a randomised controlled trial in South Africa. Lancet Glob. Health 5, e604–e614. https://doi.org/10.1016/S2214-109X(17)30146-8

Thurman, T.R., Nice, J., Taylor, T.M., Luckett, B., 2017b. Mitigating depression among orphaned and vulnerable adolescents: a randomized controlled trial of interpersonal psychotherapy for groups in South Africa. Child Adolesc. Ment. Health 22, 224–231. https://doi.org/10.1111/camh.12241

Tol, W.A., Augustinavicius, J., Carswell, K., Brown, F.L., Adaku, A., Leku, M.R., Garcia-Moreno, C., Ventevogel, P., White, R.G., van Ommeren, M., 30128161, 2018. Translation, adaptation, and pilot of a guided self-help intervention to reduce psychological distress in South Sudanese refugees in Uganda. Glob. Ment. Health 5, e25. https://doi.org/10.1017/gmh.2018.14

Tol, W.A., Komproe, I.H., Jordans, M.J.D., Ndayisaba, A., Ntamutumba, P., Sipsma, H., Smallegange, E.S., Macy, R.D., de Jong, J.T.V.M., 2014. School-based mental health intervention for children in war-affected Burundi: A cluster randomized trial. BMC Med. 12. https://doi.org/10.1186/1741-7015-12-56

Tol, W.A., Leku, M.R., Lakin, D.P., Carswell, K., Augustinavicius, J., Adaku, A., Au, T.M., Brown, F.L., Bryant, R.A., Garcia-Moreno, C., Musci, R.J., Ventevogel, P., White, R.G., van Ommeren, M., 2020. Guided self-help to reduce psychological distress in South Sudanese female refugees in Uganda: a cluster randomised trial. Lancet Glob. Health 8, e254–e263. https://doi.org/10.1016/S2214-109X(19)30504-2

Tomlinson, M., Rotheram-Borus, M.J., le Roux, I.M., Youssef, M., Nelson, S.H., Scheffler, A., Weiss, R.E., O'Connor, M., Worthman, C.M., 2016. Thirty-Six-Month Outcomes of a Generalist Paraprofessional Perinatal Home Visiting Intervention in South Africa on Maternal Health and Child Health and Development. Prev. Sci. Off. J. Soc. Prev. Res. 17, 937–948.

Tomlinson, M., Skeen, S., Melendez-Torres, G.J., Hunt, X., Desmond, C., Morgan, B., Murray, L., Cooper, P.J., Rathod, S.D., Marlow, M., Fearon, P., 2021. First 1,000 days: enough for mothers but not for children? Long-term outcomes of an early intervention on maternal depressed mood and child cognitive development: follow-up of a randomised controlled trial. J. Child Psychol. Psychiatry. https://doi.org/10.1111/jcpp.13482

Unterhitzenberger, J., Rosner, R., 2014. Lessons from writing sessions: a school-based randomized trial with adolescent orphans in Rwanda. Eur. J. Psychotraumatology 5, 24917. https://doi.org/10.3402/ejpt.v5.24917

van de Water, T., Rossouw, J., van der Watt, A.S.J., Yadin, E., Seedat, S., 2018. Adolescents' Experience of Stigma When Accessing School-Based PTSD Interventions. Qual. Health Res. 28, 1088–1098. https://doi.org/10.1177/1049732318761365

Van der Watt, A.S.J., Roos, T., Beyer, C., Seedat, S., 2018. Participants' perspectives of weekly telephonic mood monitoring in South Africa: A feasibility study. Pilot Feasibility Stud. 4. https://doi.org/10.1186/s40814-018-0245-0

Vancampfort, D., Byansi, P.K., Namutebi, H., Kinyanda, E., Bbosa, R.S., Ward, P.B., Lukwata, H., Mugisha, J., 2021. The efficacy of a lay health workers–led physical activity counselling program in patients with HIV and mental health problems: a real-world intervention from Uganda. AIDS Care - Psychol. Socio-Med. Asp. AIDSHIV. https://doi.org/10.1080/09540121.2021.1874268

Van't Hof, E., Stein, D.J., Marks, I., Tomlinson, M., Cuijpers, P., 2011. The effectiveness of problem solving therapy in deprived South African communities: Results from a pilot study. BMC Psychiatry 11. https://doi.org/10.1186/1471-244X-11-156

Vavani, B., Kraaij, V., Spinhoven, P., Garnefski, N., 2019. A booklet self-help intervention to reduce depressive symptoms among people living with HIV in Botswana: Study protocol for a randomized controlled trial. Trials 20. https://doi.org/10.1186/s13063-019-3584-0

Venturo-Conerly, K.E., Johnson, N.E., Osborn, T.L., Puffer, E.S., Rusch, T., Ndetei, D.M., Wasanga, C.M., Mutiso, V., Musyimi, C., Weisz, J.R., 2022a. Long-term health outcomes of adolescent character strength interventions: 3- to 4-year outcomes of three randomized controlled trials of the Shamiri program. Trials 23. https://doi.org/10.1186/s13063-022-06394-7

Venturo-Conerly, K.E., Osborn, T.L., Alemu, R., Roe, E., Rodriguez, M., Gan, J., Arango, S., Wasil, A., Wasanga, C., Weisz, J.R., 2022b. Single-session interventions for adolescent anxiety and depression symptoms in Kenya: A cluster-randomized controlled trial. Behav. Res. Ther. 151. https://doi.org/10.1016/j.brat.2022.104040

Venturo-Conerly, K.E., Osborn, T.L., Wasil, A.R., Le, H., Corrigan, E., Wasanga, C., Weisz, J.R., 2021. Testing the effects of the Shamiri Intervention and its components on anxiety, depression, wellbeing, and academic functioning in Kenyan adolescents: study protocol for a five-arm randomized controlled trial. Trials 22. https://doi.org/10.1186/s13063-021-05736-1

Verduin, F., Smid, G.E., Wind, T.R., Scholte, W.F., 2014. In search of links between social capital, mental health and sociotherapy: a longitudinal study in Rwanda. Soc. Sci. Med. 121, 1–9. https://doi.org/10.1016/j.socscimed.2014.09.054

Victor-Aigbodion, V., Eseadi, C., Ardi, Z., Sewagegn, A.A., Ololo, K., Abonor, L.B., Aloh, H.E., Falade, T.A., Effanga, O.A., 2023. Effectiveness of rational emotive behavior therapy in reducing depression among undergraduate medical students. Med. U. S. 102, E32724. https://doi.org/10.1097/MD.0000000000032724

Vigna-Taglianti, F., Mehanović, E., Alesina, M., Damjanović, L., Ibanga, A., Pwajok, J., Prichard, G., van der Kreeft, P., Virk, H.K., 2021. Effects of the "Unplugged" school-based substance use prevention program in Nigeria: A cluster randomized controlled trial. Drug Alcohol Depend. 228. https://doi.org/10.1016/j.drugalcdep.2021.108966

Wasil, A.R., Osborn, T.L., Weisz, J.R., DeRubeis, R.J., 2021. Online single-session interventions for Kenyan adolescents: study protocol for a comparative effectiveness randomised controlled trial. Gen. Psychiatry 34, e100446. https://doi.org/10.1136/gpsych-2020-100446

Wechsberg, W.M., Bonner, C.P., Zule, W.A., van der Horst, C., Ndirangu, J., Browne, F.A., Kline, T.L., Howard, B.N., Rodman, N.F., 2019. Addressing the nexus of risk: Biobehavioral outcomes from a cluster randomized trial of the Women's Health CoOp Plus in Pretoria,

South Africa. Drug Alcohol Depend. 195, 16–26. https://doi.org/10.1016/j.drugalcdep.2018.10.036

Wechsberg, W.M., Browne, F.A., Carney, T., Myers, B., Minnis, A., MacDonald, R., Ndirangu, J.W., Turner, L.B., Howard, B.N., Rodman, N., 2018. The Young Women’s Health CoOp in Cape Town, South Africa: Study protocol for a cluster-randomised trial for adolescent women at risk for HIV. BMC Public Health 18, 859. https://doi.org/10.1186/s12889-018-5665-5

Williams, P.P., Carney, T., Parry, C.D.H., 2016. Reducing substance use and sexual risk behaviour among men who have sex with men in South Africa. South Afr. J. Sci. 112. https://doi.org/10.17159/sajs.2016/20150425

Williams, P.P., Carney, T., Plüddemann, A., Parry, C.D.H., 2014. Intervening to identify and reduce drug use and sexual HIV risk patterns among men who have sex with men in three provinces in South Africa. J. Subst. Use 19, 141–146. https://doi.org/10.3109/14659891.2012.760009

Woollett, N., Bandeira, M., Hatcher, A., 2020. Trauma-informed art and play therapy: Pilot study outcomes for children and mothers in domestic violence shelters in the United States and South Africa. Child Abuse Negl. 107. https://doi.org/10.1016/j.chiabu.2020.104564

Wright, J., Chiwandira, C., 2016. Building capacity for community mental health care in rural Malawi: Findings from a district-wide task-sharing intervention with village-based health workers. Int. J. Soc. Psychiatry 62, 589–596. https://doi.org/10.1177/0020764016657112

Zanoni, B.C., Archary, M., Sibaya, T., Goldstein, M., Bergam, S., Denton, D., Cordero, V., Peng, C., Psaros, C., Marconi, V.C., Haberer, J.E., 2022. Mobile Phone-Based Intervention Among Adolescents Living With Perinatally Acquired HIV Transitioning from Pediatric to Adult Care: Protocol for the Interactive Transition Support for Adolescents Living With HIV using Social Media (InTSHA) Study. JMIR Res Protoc 11, e35455. https://doi.org/10.2196/35455

Zoellner, L.A., Bentley, J.A., Feeny, N.C., Klein, A.B., Dolezal, M.L., Angula, D.A., Egeh, M.H., 2021. Reaching the Unreached: Bridging Islam and Science to Treat the Mental Wounds of War. Front. Psychiatry 12. https://doi.org/10.3389/fpsyt.2021.599293

Zule, W., Myers, B., Carney, T., Novak, S.P., McCormick, K., Wechsberg, W.M., 2014. Alcohol and drug use outcomes among vulnerable women living with HIV: results from the Western Cape Women’s Health CoOp. AIDS Care 26, 1494–1499. https://doi.org/10.1080/09540121.2014.933769
